# Supplementary material for: The Gut Bacteria Dysbiosis Contributes to Chronic Graft-Versus-Host Disease Associated With a Treg/Th1 Ratio Imbalance
Source: Front Microbiol. 2022 Sep 8;13:813576. doi: 10.3389/fmicb.2022.813576 (PMC9493085; doi:10.3389/fmicb.2022.813576)
Supplement: Supplementary file 1 [file Data_Sheet_1.zip › P101SC18090073-01-B1-3-4_result/05.WebShow/taxa_charts_html/pie_charts.html]

Taxa Summaries


|  |  |
| --- | --- |
|  | |
| Taxonomy Summary. Current Level: | |
| View Figure (.pdf)  View Legend (.pdf) |  |
|  |  |

|  |  |  |
| --- | --- | --- |
| Count | Pct | Taxonomy |
| 5.28373526335 | 52.84% | k\_\_Bacteria;p\_\_Bacteroidetes |
| 3.8134205988 | 38.13% | k\_\_Bacteria;p\_\_Firmicutes |
| 0.691159424387 | 6.91% | k\_\_Bacteria;p\_\_Proteobacteria |
| 0.135746798225 | 1.36% | k\_\_Bacteria;p\_\_Verrucomicrobia |
| 0.0450934381273 | 0.45% | k\_\_Bacteria;p\_\_Tenericutes |
| 0.00845325266461 | 0.08% | k\_\_Bacteria;Other |
| 0.00606428995505 | 0.06% | k\_\_Bacteria;p\_\_unidentified\_Bacteria |
| 0.00463656667892 | 0.05% | k\_\_Bacteria;p\_\_Melainabacteria |
| 0.00435384919849 | 0.04% | k\_\_Bacteria;p\_\_Actinobacteria |
| 0.00400045234797 | 0.04% | k\_\_Bacteria;p\_\_Oxyphotobacteria |
| 0.00295439767041 | 0.03% | k\_\_Bacteria;p\_\_Fusobacteria |
| 0.00025444573238 | 0.00% | k\_\_Bacteria;p\_\_Acidobacteria |
| 7.06793701055e-05 | 0.00% | k\_\_Archaea;p\_\_Euryarchaeota |
| 2.82717480422e-05 | 0.00% | k\_\_Bacteria;p\_\_Deinococcus-Thermus |
| 2.82717480422e-05 | 0.00% | k\_\_Bacteria;p\_\_Armatimonadetes |

|  |  |
| --- | --- |
|  | |
| Taxonomy Summary. Current Level: \_MD1 | |
| View Figure (.pdf)  View Legend (.pdf) |  |
|  |  |

|  |  |  |
| --- | --- | --- |
| Count | Pct | Taxonomy |
| 0.47739673744 | 47.74% | k\_\_Bacteria;p\_\_Firmicutes |
| 0.430168782336 | 43.02% | k\_\_Bacteria;p\_\_Bacteroidetes |
| 0.0887167453564 | 8.87% | k\_\_Bacteria;p\_\_Proteobacteria |
| 0.00166803313449 | 0.17% | k\_\_Bacteria;p\_\_Verrucomicrobia |
| 0.000819880693223 | 0.08% | k\_\_Bacteria;p\_\_Tenericutes |
| 0.000353396850527 | 0.04% | k\_\_Bacteria;Other |
| 0.000296853354443 | 0.03% | k\_\_Bacteria;p\_\_Fusobacteria |
| 0.000282717480422 | 0.03% | k\_\_Bacteria;p\_\_Actinobacteria |
| 0.000169630488253 | 0.02% | k\_\_Bacteria;p\_\_unidentified\_Bacteria |
| 9.89511181476e-05 | 0.01% | k\_\_Bacteria;p\_\_Melainabacteria |
| 2.82717480422e-05 | 0.00% | k\_\_Bacteria;p\_\_Oxyphotobacteria |

|  |  |
| --- | --- |
|  | |
| Taxonomy Summary. Current Level: \_MD2 | |
| View Figure (.pdf)  View Legend (.pdf) |  |
|  |  |

|  |  |  |
| --- | --- | --- |
| Count | Pct | Taxonomy |
| 0.474880551865 | 47.49% | k\_\_Bacteria;p\_\_Bacteroidetes |
| 0.410138248848 | 41.01% | k\_\_Bacteria;p\_\_Firmicutes |
| 0.10476096237 | 10.48% | k\_\_Bacteria;p\_\_Proteobacteria |
| 0.00407113171807 | 0.41% | k\_\_Bacteria;p\_\_Verrucomicrobia |
| 0.00190834299285 | 0.19% | k\_\_Bacteria;p\_\_Oxyphotobacteria |
| 0.00171044075655 | 0.17% | k\_\_Bacteria;Other |
| 0.00135704390602 | 0.14% | k\_\_Bacteria;p\_\_Tenericutes |
| 0.000395804472591 | 0.04% | k\_\_Bacteria;p\_\_unidentified\_Bacteria |
| 0.000353396850527 | 0.04% | k\_\_Bacteria;p\_\_Actinobacteria |
| 0.00025444573238 | 0.03% | k\_\_Bacteria;p\_\_Fusobacteria |
| 0.000169630488253 | 0.02% | k\_\_Bacteria;p\_\_Melainabacteria |

|  |  |
| --- | --- |
|  | |
| Taxonomy Summary. Current Level: \_MD3 | |
| View Figure (.pdf)  View Legend (.pdf) |  |
|  |  |

|  |  |  |
| --- | --- | --- |
| Count | Pct | Taxonomy |
| 0.47162930084 | 47.16% | k\_\_Bacteria;p\_\_Bacteroidetes |
| 0.414025614204 | 41.40% | k\_\_Bacteria;p\_\_Firmicutes |
| 0.107036838088 | 10.70% | k\_\_Bacteria;p\_\_Proteobacteria |
| 0.00337847389104 | 0.34% | k\_\_Bacteria;p\_\_Verrucomicrobia |
| 0.000932967685392 | 0.09% | k\_\_Bacteria;Other |
| 0.000819880693223 | 0.08% | k\_\_Bacteria;p\_\_Tenericutes |
| 0.000819880693223 | 0.08% | k\_\_Bacteria;p\_\_Oxyphotobacteria |
| 0.000579570834865 | 0.06% | k\_\_Bacteria;p\_\_Actinobacteria |
| 0.000310989228464 | 0.03% | k\_\_Bacteria;p\_\_unidentified\_Bacteria |
| 0.000212038110316 | 0.02% | k\_\_Bacteria;p\_\_Fusobacteria |
| 0.000169630488253 | 0.02% | k\_\_Bacteria;p\_\_Acidobacteria |
| 8.48152441265e-05 | 0.01% | k\_\_Bacteria;p\_\_Melainabacteria |

|  |  |
| --- | --- |
|  | |
| Taxonomy Summary. Current Level: \_MD4 | |
| View Figure (.pdf)  View Legend (.pdf) |  |
|  |  |

|  |  |  |
| --- | --- | --- |
| Count | Pct | Taxonomy |
| 0.682932345707 | 68.29% | k\_\_Bacteria;p\_\_Bacteroidetes |
| 0.220533770603 | 22.05% | k\_\_Bacteria;p\_\_Firmicutes |
| 0.0842356732917 | 8.42% | k\_\_Bacteria;p\_\_Proteobacteria |
| 0.0103333239094 | 1.03% | k\_\_Bacteria;p\_\_Verrucomicrobia |
| 0.000720929575076 | 0.07% | k\_\_Bacteria;p\_\_unidentified\_Bacteria |
| 0.000325125102485 | 0.03% | k\_\_Bacteria;p\_\_Fusobacteria |
| 0.000296853354443 | 0.03% | k\_\_Bacteria;p\_\_Melainabacteria |
| 0.000282717480422 | 0.03% | k\_\_Bacteria;p\_\_Tenericutes |
| 0.000268581606401 | 0.03% | k\_\_Bacteria;p\_\_Actinobacteria |
| 7.06793701055e-05 | 0.01% | k\_\_Bacteria;p\_\_Oxyphotobacteria |

|  |  |
| --- | --- |
|  | |
| Taxonomy Summary. Current Level: \_MD5 | |
| View Figure (.pdf)  View Legend (.pdf) |  |
|  |  |

|  |  |  |
| --- | --- | --- |
| Count | Pct | Taxonomy |
| 0.500395804473 | 50.04% | k\_\_Bacteria;p\_\_Bacteroidetes |
| 0.459938933024 | 45.99% | k\_\_Bacteria;p\_\_Firmicutes |
| 0.0337706030364 | 3.38% | k\_\_Bacteria;p\_\_Proteobacteria |
| 0.0041559469622 | 0.42% | k\_\_Bacteria;p\_\_Tenericutes |
| 0.000508891464759 | 0.05% | k\_\_Bacteria;p\_\_unidentified\_Bacteria |
| 0.000395804472591 | 0.04% | k\_\_Bacteria;p\_\_Verrucomicrobia |
| 0.000268581606401 | 0.03% | k\_\_Bacteria;p\_\_Fusobacteria |
| 0.000268581606401 | 0.03% | k\_\_Bacteria;Other |
| 0.000197902236295 | 0.02% | k\_\_Bacteria;p\_\_Actinobacteria |
| 8.48152441265e-05 | 0.01% | k\_\_Bacteria;p\_\_Melainabacteria |
| 1.41358740211e-05 | 0.00% | k\_\_Bacteria;p\_\_Oxyphotobacteria |

|  |  |
| --- | --- |
|  | |
| Taxonomy Summary. Current Level: \_MC1 | |
| View Figure (.pdf)  View Legend (.pdf) |  |
|  |  |

|  |  |  |
| --- | --- | --- |
| Count | Pct | Taxonomy |
| 0.563569025473 | 56.36% | k\_\_Bacteria;p\_\_Bacteroidetes |
| 0.28425829069 | 28.43% | k\_\_Bacteria;p\_\_Firmicutes |
| 0.0868084023635 | 8.68% | k\_\_Bacteria;p\_\_Verrucomicrobia |
| 0.0345763478556 | 3.46% | k\_\_Bacteria;p\_\_Proteobacteria |
| 0.0251901275056 | 2.52% | k\_\_Bacteria;p\_\_Tenericutes |
| 0.00166803313449 | 0.17% | k\_\_Bacteria;p\_\_Melainabacteria |
| 0.0012722286619 | 0.13% | k\_\_Bacteria;Other |
| 0.00104605467756 | 0.10% | k\_\_Bacteria;p\_\_Oxyphotobacteria |
| 0.00065025020497 | 0.07% | k\_\_Bacteria;p\_\_unidentified\_Bacteria |
| 0.000537163212801 | 0.05% | k\_\_Bacteria;p\_\_Actinobacteria |
| 0.000395804472591 | 0.04% | k\_\_Bacteria;p\_\_Fusobacteria |
| 2.82717480422e-05 | 0.00% | k\_\_Bacteria;p\_\_Armatimonadetes |

|  |  |
| --- | --- |
|  | |
| Taxonomy Summary. Current Level: \_MC2 | |
| View Figure (.pdf)  View Legend (.pdf) |  |
|  |  |

|  |  |  |
| --- | --- | --- |
| Count | Pct | Taxonomy |
| 0.644723078228 | 64.47% | k\_\_Bacteria;p\_\_Bacteroidetes |
| 0.240027140878 | 24.00% | k\_\_Bacteria;p\_\_Firmicutes |
| 0.0963642532018 | 9.64% | k\_\_Bacteria;p\_\_Proteobacteria |
| 0.0124678408866 | 1.25% | k\_\_Bacteria;p\_\_Verrucomicrobia |
| 0.00385909360776 | 0.39% | k\_\_Bacteria;p\_\_Tenericutes |
| 0.00114500579571 | 0.11% | k\_\_Bacteria;p\_\_unidentified\_Bacteria |
| 0.000537163212801 | 0.05% | k\_\_Bacteria;p\_\_Actinobacteria |
| 0.000310989228464 | 0.03% | k\_\_Bacteria;p\_\_Melainabacteria |
| 0.000282717480422 | 0.03% | k\_\_Bacteria;p\_\_Fusobacteria |
| 0.00025444573238 | 0.03% | k\_\_Bacteria;Other |
| 1.41358740211e-05 | 0.00% | k\_\_Bacteria;p\_\_Oxyphotobacteria |
| 1.41358740211e-05 | 0.00% | k\_\_Archaea;p\_\_Euryarchaeota |

|  |  |
| --- | --- |
|  | |
| Taxonomy Summary. Current Level: \_MC3 | |
| View Figure (.pdf)  View Legend (.pdf) |  |
|  |  |

|  |  |  |
| --- | --- | --- |
| Count | Pct | Taxonomy |
| 0.614557123067 | 61.46% | k\_\_Bacteria;p\_\_Bacteroidetes |
| 0.315993327867 | 31.60% | k\_\_Bacteria;p\_\_Firmicutes |
| 0.0625088349213 | 6.25% | k\_\_Bacteria;p\_\_Proteobacteria |
| 0.00318057165475 | 0.32% | k\_\_Bacteria;p\_\_Tenericutes |
| 0.00114500579571 | 0.11% | k\_\_Bacteria;p\_\_Verrucomicrobia |
| 0.000735065449097 | 0.07% | k\_\_Bacteria;p\_\_unidentified\_Bacteria |
| 0.000706793701055 | 0.07% | k\_\_Bacteria;Other |
| 0.000593706708886 | 0.06% | k\_\_Bacteria;p\_\_Actinobacteria |
| 0.000268581606401 | 0.03% | k\_\_Bacteria;p\_\_Fusobacteria |
| 0.000155494614232 | 0.02% | k\_\_Bacteria;p\_\_Melainabacteria |
| 5.65434960844e-05 | 0.01% | k\_\_Bacteria;p\_\_Oxyphotobacteria |
| 5.65434960844e-05 | 0.01% | k\_\_Archaea;p\_\_Euryarchaeota |
| 2.82717480422e-05 | 0.00% | k\_\_Bacteria;p\_\_Acidobacteria |
| 1.41358740211e-05 | 0.00% | k\_\_Bacteria;p\_\_Deinococcus-Thermus |

|  |  |
| --- | --- |
|  | |
| Taxonomy Summary. Current Level: \_MC4 | |
| View Figure (.pdf)  View Legend (.pdf) |  |
|  |  |

|  |  |  |
| --- | --- | --- |
| Count | Pct | Taxonomy |
| 0.586921489356 | 58.69% | k\_\_Bacteria;p\_\_Firmicutes |
| 0.387238132934 | 38.72% | k\_\_Bacteria;p\_\_Bacteroidetes |
| 0.0202567074722 | 2.03% | k\_\_Bacteria;p\_\_Proteobacteria |
| 0.00188007124481 | 0.19% | k\_\_Bacteria;Other |
| 0.00130050040994 | 0.13% | k\_\_Bacteria;p\_\_Verrucomicrobia |
| 0.000819880693223 | 0.08% | k\_\_Bacteria;p\_\_Tenericutes |
| 0.000565434960844 | 0.06% | k\_\_Bacteria;p\_\_unidentified\_Bacteria |
| 0.000339260976506 | 0.03% | k\_\_Bacteria;p\_\_Actinobacteria |
| 0.000325125102485 | 0.03% | k\_\_Bacteria;p\_\_Melainabacteria |
| 0.000310989228464 | 0.03% | k\_\_Bacteria;p\_\_Fusobacteria |
| 4.24076220633e-05 | 0.00% | k\_\_Bacteria;p\_\_Oxyphotobacteria |

|  |  |
| --- | --- |
|  | |
| Taxonomy Summary. Current Level: \_MC5 | |
| View Figure (.pdf)  View Legend (.pdf) |  |
|  |  |

|  |  |  |
| --- | --- | --- |
| Count | Pct | Taxonomy |
| 0.51364111843 | 51.36% | k\_\_Bacteria;p\_\_Bacteroidetes |
| 0.404187045885 | 40.42% | k\_\_Bacteria;p\_\_Firmicutes |
| 0.0589324587939 | 5.89% | k\_\_Bacteria;p\_\_Proteobacteria |
| 0.0141782816432 | 1.42% | k\_\_Bacteria;p\_\_Verrucomicrobia |
| 0.00460829493088 | 0.46% | k\_\_Bacteria;p\_\_Tenericutes |
| 0.00144185915015 | 0.14% | k\_\_Bacteria;p\_\_Melainabacteria |
| 0.0010743264256 | 0.11% | k\_\_Bacteria;Other |
| 0.000862288315287 | 0.09% | k\_\_Bacteria;p\_\_unidentified\_Bacteria |
| 0.000664386078991 | 0.07% | k\_\_Bacteria;p\_\_Actinobacteria |
| 0.000339260976506 | 0.03% | k\_\_Bacteria;p\_\_Fusobacteria |
| 5.65434960844e-05 | 0.01% | k\_\_Bacteria;p\_\_Acidobacteria |
| 1.41358740211e-05 | 0.00% | k\_\_Bacteria;p\_\_Deinococcus-Thermus |

|  |  |
| --- | --- |
|  | |
| Taxonomy Summary. Current Level: | |
| View Figure (.pdf)  View Legend (.pdf) |  |
|  |  |
| View Figure (.pdf)  View Legend (.pdf) |  |
|  |  |
|  |  |

|  |  |  |
| --- | --- | --- |
| Count | Pct | Taxonomy |
| 5.28373526335 | 52.84% | k\_\_Bacteria;p\_\_Bacteroidetes;c\_\_Bacteroidia |
| 3.66468293235 | 36.65% | k\_\_Bacteria;p\_\_Firmicutes;c\_\_Clostridia |
| 0.578213790959 | 5.78% | k\_\_Bacteria;p\_\_Proteobacteria;c\_\_Gammaproteobacteria |
| 0.135746798225 | 1.36% | k\_\_Bacteria;p\_\_Verrucomicrobia;c\_\_Verrucomicrobiae |
| 0.1115461819 | 1.12% | k\_\_Bacteria;p\_\_Proteobacteria;c\_\_Deltaproteobacteria |
| 0.0999406293291 | 1.00% | k\_\_Bacteria;p\_\_Firmicutes;c\_\_Bacilli |
| 0.0450934381273 | 0.45% | k\_\_Bacteria;p\_\_Tenericutes;c\_\_Mollicutes |
| 0.0404427355743 | 0.40% | k\_\_Bacteria;p\_\_Firmicutes;c\_\_Erysipelotrichia |
| 0.00845325266461 | 0.08% | k\_\_Bacteria;Other;Other |
| 0.00606428995505 | 0.06% | k\_\_Bacteria;p\_\_unidentified\_Bacteria;c\_\_unidentified\_Bacteria |
| 0.00487687653728 | 0.05% | k\_\_Bacteria;p\_\_Firmicutes;c\_\_Negativicutes |
| 0.00463656667892 | 0.05% | k\_\_Bacteria;p\_\_Melainabacteria;Other |
| 0.00400045234797 | 0.04% | k\_\_Bacteria;p\_\_Oxyphotobacteria;c\_\_unidentified\_Oxyphotobacteria |
| 0.00374600661559 | 0.04% | k\_\_Bacteria;p\_\_Actinobacteria;c\_\_unidentified\_Actinobacteria |
| 0.00347742500919 | 0.03% | k\_\_Bacteria;p\_\_Firmicutes;Other |
| 0.00295439767041 | 0.03% | k\_\_Bacteria;p\_\_Fusobacteria;c\_\_Fusobacteriia |
| 0.00135704390602 | 0.01% | k\_\_Bacteria;p\_\_Proteobacteria;c\_\_Alphaproteobacteria |
| 0.000240309858359 | 0.00% | k\_\_Bacteria;p\_\_Actinobacteria;c\_\_Thermoleophilia |
| 0.000212038110316 | 0.00% | k\_\_Bacteria;p\_\_Actinobacteria;c\_\_Rubrobacteria |
| 0.000169630488253 | 0.00% | k\_\_Bacteria;p\_\_Acidobacteria;c\_\_unidentified\_Acidobacteria |
| 8.48152441266e-05 | 0.00% | k\_\_Bacteria;p\_\_Acidobacteria;c\_\_Holophagae |
| 8.48152441265e-05 | 0.00% | k\_\_Bacteria;p\_\_Actinobacteria;c\_\_Acidimicrobiia |
| 7.06793701055e-05 | 0.00% | k\_\_Bacteria;p\_\_Actinobacteria;c\_\_Coriobacteriia |
| 7.06793701055e-05 | 0.00% | k\_\_Archaea;p\_\_Euryarchaeota;c\_\_Methanobacteria |
| 4.24076220633e-05 | 0.00% | k\_\_Bacteria;p\_\_Proteobacteria;Other |
| 2.82717480422e-05 | 0.00% | k\_\_Bacteria;p\_\_Deinococcus-Thermus;c\_\_Deinococci |
| 2.82717480422e-05 | 0.00% | k\_\_Bacteria;p\_\_Armatimonadetes;c\_\_Fimbriimonadia |

|  |  |
| --- | --- |
|  | |
| Taxonomy Summary. Current Level: \_MD1 | |
| View Figure (.pdf)  View Legend (.pdf) |  |
|  |  |

|  |  |  |
| --- | --- | --- |
| Count | Pct | Taxonomy |
| 0.468717310791 | 46.87% | k\_\_Bacteria;p\_\_Firmicutes;c\_\_Clostridia |
| 0.430168782336 | 43.02% | k\_\_Bacteria;p\_\_Bacteroidetes;c\_\_Bacteroidia |
| 0.0787085465494 | 7.87% | k\_\_Bacteria;p\_\_Proteobacteria;c\_\_Gammaproteobacteria |
| 0.00993751943683 | 0.99% | k\_\_Bacteria;p\_\_Proteobacteria;c\_\_Deltaproteobacteria |
| 0.00561194198637 | 0.56% | k\_\_Bacteria;p\_\_Firmicutes;c\_\_Bacilli |
| 0.00244550620565 | 0.24% | k\_\_Bacteria;p\_\_Firmicutes;c\_\_Erysipelotrichia |
| 0.00166803313449 | 0.17% | k\_\_Bacteria;p\_\_Verrucomicrobia;c\_\_Verrucomicrobiae |
| 0.000819880693223 | 0.08% | k\_\_Bacteria;p\_\_Tenericutes;c\_\_Mollicutes |
| 0.000409940346612 | 0.04% | k\_\_Bacteria;p\_\_Firmicutes;c\_\_Negativicutes |
| 0.000353396850527 | 0.04% | k\_\_Bacteria;Other;Other |
| 0.000296853354443 | 0.03% | k\_\_Bacteria;p\_\_Fusobacteria;c\_\_Fusobacteriia |
| 0.000268581606401 | 0.03% | k\_\_Bacteria;p\_\_Actinobacteria;c\_\_unidentified\_Actinobacteria |
| 0.000212038110316 | 0.02% | k\_\_Bacteria;p\_\_Firmicutes;Other |
| 0.000169630488253 | 0.02% | k\_\_Bacteria;p\_\_unidentified\_Bacteria;c\_\_unidentified\_Bacteria |
| 9.89511181476e-05 | 0.01% | k\_\_Bacteria;p\_\_Melainabacteria;Other |
| 7.06793701055e-05 | 0.01% | k\_\_Bacteria;p\_\_Proteobacteria;c\_\_Alphaproteobacteria |
| 2.82717480422e-05 | 0.00% | k\_\_Bacteria;p\_\_Oxyphotobacteria;c\_\_unidentified\_Oxyphotobacteria |
| 1.41358740211e-05 | 0.00% | k\_\_Bacteria;p\_\_Actinobacteria;c\_\_Thermoleophilia |

|  |  |
| --- | --- |
|  | |
| Taxonomy Summary. Current Level: \_MD2 | |
| View Figure (.pdf)  View Legend (.pdf) |  |
|  |  |

|  |  |  |
| --- | --- | --- |
| Count | Pct | Taxonomy |
| 0.474880551865 | 47.49% | k\_\_Bacteria;p\_\_Bacteroidetes;c\_\_Bacteroidia |
| 0.399239489978 | 39.92% | k\_\_Bacteria;p\_\_Firmicutes;c\_\_Clostridia |
| 0.0929150999406 | 9.29% | k\_\_Bacteria;p\_\_Proteobacteria;c\_\_Gammaproteobacteria |
| 0.0117751830596 | 1.18% | k\_\_Bacteria;p\_\_Proteobacteria;c\_\_Deltaproteobacteria |
| 0.00735065449097 | 0.74% | k\_\_Bacteria;p\_\_Firmicutes;c\_\_Bacilli |
| 0.00407113171807 | 0.41% | k\_\_Bacteria;p\_\_Verrucomicrobia;c\_\_Verrucomicrobiae |
| 0.00296853354443 | 0.30% | k\_\_Bacteria;p\_\_Firmicutes;c\_\_Erysipelotrichia |
| 0.00190834299285 | 0.19% | k\_\_Bacteria;p\_\_Oxyphotobacteria;c\_\_unidentified\_Oxyphotobacteria |
| 0.00171044075655 | 0.17% | k\_\_Bacteria;Other;Other |
| 0.00135704390602 | 0.14% | k\_\_Bacteria;p\_\_Tenericutes;c\_\_Mollicutes |
| 0.000395804472591 | 0.04% | k\_\_Bacteria;p\_\_unidentified\_Bacteria;c\_\_unidentified\_Bacteria |
| 0.000325125102485 | 0.03% | k\_\_Bacteria;p\_\_Firmicutes;c\_\_Negativicutes |
| 0.000310989228464 | 0.03% | k\_\_Bacteria;p\_\_Actinobacteria;c\_\_unidentified\_Actinobacteria |
| 0.00025444573238 | 0.03% | k\_\_Bacteria;p\_\_Fusobacteria;c\_\_Fusobacteriia |
| 0.00025444573238 | 0.03% | k\_\_Bacteria;p\_\_Firmicutes;Other |
| 0.000169630488253 | 0.02% | k\_\_Bacteria;p\_\_Melainabacteria;Other |
| 7.06793701055e-05 | 0.01% | k\_\_Bacteria;p\_\_Proteobacteria;c\_\_Alphaproteobacteria |
| 4.24076220633e-05 | 0.00% | k\_\_Bacteria;p\_\_Actinobacteria;c\_\_Coriobacteriia |

|  |  |
| --- | --- |
|  | |
| Taxonomy Summary. Current Level: \_MD3 | |
| View Figure (.pdf)  View Legend (.pdf) |  |
|  |  |
| View Figure (.pdf)  View Legend (.pdf) |  |
|  |  |
|  |  |

|  |  |  |
| --- | --- | --- |
| Count | Pct | Taxonomy |
| 0.47162930084 | 47.16% | k\_\_Bacteria;p\_\_Bacteroidetes;c\_\_Bacteroidia |
| 0.401091289474 | 40.11% | k\_\_Bacteria;p\_\_Firmicutes;c\_\_Clostridia |
| 0.0932402250431 | 9.32% | k\_\_Bacteria;p\_\_Proteobacteria;c\_\_Gammaproteobacteria |
| 0.01342908032 | 1.34% | k\_\_Bacteria;p\_\_Proteobacteria;c\_\_Deltaproteobacteria |
| 0.00759096434933 | 0.76% | k\_\_Bacteria;p\_\_Firmicutes;c\_\_Bacilli |
| 0.00460829493088 | 0.46% | k\_\_Bacteria;p\_\_Firmicutes;c\_\_Erysipelotrichia |
| 0.00337847389104 | 0.34% | k\_\_Bacteria;p\_\_Verrucomicrobia;c\_\_Verrucomicrobiae |
| 0.000932967685392 | 0.09% | k\_\_Bacteria;Other;Other |
| 0.000819880693223 | 0.08% | k\_\_Bacteria;p\_\_Tenericutes;c\_\_Mollicutes |
| 0.000819880693223 | 0.08% | k\_\_Bacteria;p\_\_Oxyphotobacteria;c\_\_unidentified\_Oxyphotobacteria |
| 0.000438212094654 | 0.04% | k\_\_Bacteria;p\_\_Firmicutes;c\_\_Negativicutes |
| 0.000367532724548 | 0.04% | k\_\_Bacteria;p\_\_Proteobacteria;c\_\_Alphaproteobacteria |
| 0.000310989228464 | 0.03% | k\_\_Bacteria;p\_\_unidentified\_Bacteria;c\_\_unidentified\_Bacteria |
| 0.000296853354443 | 0.03% | k\_\_Bacteria;p\_\_Firmicutes;Other |
| 0.00025444573238 | 0.03% | k\_\_Bacteria;p\_\_Actinobacteria;c\_\_unidentified\_Actinobacteria |
| 0.000212038110316 | 0.02% | k\_\_Bacteria;p\_\_Fusobacteria;c\_\_Fusobacteriia |
| 0.000155494614232 | 0.02% | k\_\_Bacteria;p\_\_Actinobacteria;c\_\_Rubrobacteria |
| 0.000113086992169 | 0.01% | k\_\_Bacteria;p\_\_Acidobacteria;c\_\_unidentified\_Acidobacteria |
| 8.48152441265e-05 | 0.01% | k\_\_Bacteria;p\_\_Melainabacteria;Other |
| 8.48152441265e-05 | 0.01% | k\_\_Bacteria;p\_\_Actinobacteria;c\_\_Thermoleophilia |
| 8.48152441265e-05 | 0.01% | k\_\_Bacteria;p\_\_Actinobacteria;c\_\_Acidimicrobiia |
| 5.65434960844e-05 | 0.01% | k\_\_Bacteria;p\_\_Acidobacteria;c\_\_Holophagae |

|  |  |
| --- | --- |
|  | |
| Taxonomy Summary. Current Level: \_MD4 | |
| View Figure (.pdf)  View Legend (.pdf) |  |
|  |  |

|  |  |  |
| --- | --- | --- |
| Count | Pct | Taxonomy |
| 0.682932345707 | 68.29% | k\_\_Bacteria;p\_\_Bacteroidetes;c\_\_Bacteroidia |
| 0.208617228803 | 20.86% | k\_\_Bacteria;p\_\_Firmicutes;c\_\_Clostridia |
| 0.0761216816036 | 7.61% | k\_\_Bacteria;p\_\_Proteobacteria;c\_\_Gammaproteobacteria |
| 0.0103333239094 | 1.03% | k\_\_Bacteria;p\_\_Verrucomicrobia;c\_\_Verrucomicrobiae |
| 0.00989511181476 | 0.99% | k\_\_Bacteria;p\_\_Firmicutes;c\_\_Bacilli |
| 0.00802917644398 | 0.80% | k\_\_Bacteria;p\_\_Proteobacteria;c\_\_Deltaproteobacteria |
| 0.0016114896384 | 0.16% | k\_\_Bacteria;p\_\_Firmicutes;c\_\_Erysipelotrichia |
| 0.000720929575076 | 0.07% | k\_\_Bacteria;p\_\_unidentified\_Bacteria;c\_\_unidentified\_Bacteria |
| 0.000339260976506 | 0.03% | k\_\_Bacteria;p\_\_Firmicutes;c\_\_Negativicutes |
| 0.000325125102485 | 0.03% | k\_\_Bacteria;p\_\_Fusobacteria;c\_\_Fusobacteriia |
| 0.000296853354443 | 0.03% | k\_\_Bacteria;p\_\_Melainabacteria;Other |
| 0.000282717480422 | 0.03% | k\_\_Bacteria;p\_\_Tenericutes;c\_\_Mollicutes |
| 0.000268581606401 | 0.03% | k\_\_Bacteria;p\_\_Actinobacteria;c\_\_unidentified\_Actinobacteria |
| 8.48152441265e-05 | 0.01% | k\_\_Bacteria;p\_\_Proteobacteria;c\_\_Alphaproteobacteria |
| 7.06793701055e-05 | 0.01% | k\_\_Bacteria;p\_\_Oxyphotobacteria;c\_\_unidentified\_Oxyphotobacteria |
| 7.06793701055e-05 | 0.01% | k\_\_Bacteria;p\_\_Firmicutes;Other |

|  |  |
| --- | --- |
|  | |
| Taxonomy Summary. Current Level: \_MD5 | |
| View Figure (.pdf)  View Legend (.pdf) |  |
|  |  |

|  |  |  |
| --- | --- | --- |
| Count | Pct | Taxonomy |
| 0.500395804473 | 50.04% | k\_\_Bacteria;p\_\_Bacteroidetes;c\_\_Bacteroidia |
| 0.434678126149 | 43.47% | k\_\_Bacteria;p\_\_Firmicutes;c\_\_Clostridia |
| 0.022758757174 | 2.28% | k\_\_Bacteria;p\_\_Firmicutes;c\_\_Bacilli |
| 0.0199881258658 | 2.00% | k\_\_Bacteria;p\_\_Proteobacteria;c\_\_Gammaproteobacteria |
| 0.0136128466823 | 1.36% | k\_\_Bacteria;p\_\_Proteobacteria;c\_\_Deltaproteobacteria |
| 0.0041559469622 | 0.42% | k\_\_Bacteria;p\_\_Tenericutes;c\_\_Mollicutes |
| 0.00117327754375 | 0.12% | k\_\_Bacteria;p\_\_Firmicutes;c\_\_Erysipelotrichia |
| 0.000735065449097 | 0.07% | k\_\_Bacteria;p\_\_Firmicutes;c\_\_Negativicutes |
| 0.000593706708886 | 0.06% | k\_\_Bacteria;p\_\_Firmicutes;Other |
| 0.000508891464759 | 0.05% | k\_\_Bacteria;p\_\_unidentified\_Bacteria;c\_\_unidentified\_Bacteria |
| 0.000395804472591 | 0.04% | k\_\_Bacteria;p\_\_Verrucomicrobia;c\_\_Verrucomicrobiae |
| 0.000268581606401 | 0.03% | k\_\_Bacteria;p\_\_Fusobacteria;c\_\_Fusobacteriia |
| 0.000268581606401 | 0.03% | k\_\_Bacteria;Other;Other |
| 0.000183766362274 | 0.02% | k\_\_Bacteria;p\_\_Actinobacteria;c\_\_unidentified\_Actinobacteria |
| 0.00012722286619 | 0.01% | k\_\_Bacteria;p\_\_Proteobacteria;c\_\_Alphaproteobacteria |
| 8.48152441265e-05 | 0.01% | k\_\_Bacteria;p\_\_Melainabacteria;Other |
| 4.24076220633e-05 | 0.00% | k\_\_Bacteria;p\_\_Proteobacteria;Other |
| 1.41358740211e-05 | 0.00% | k\_\_Bacteria;p\_\_Oxyphotobacteria;c\_\_unidentified\_Oxyphotobacteria |
| 1.41358740211e-05 | 0.00% | k\_\_Bacteria;p\_\_Actinobacteria;c\_\_Coriobacteriia |

|  |  |
| --- | --- |
|  | |
| Taxonomy Summary. Current Level: \_MC1 | |
| View Figure (.pdf)  View Legend (.pdf) |  |
|  |  |

|  |  |  |
| --- | --- | --- |
| Count | Pct | Taxonomy |
| 0.563569025473 | 56.36% | k\_\_Bacteria;p\_\_Bacteroidetes;c\_\_Bacteroidia |
| 0.266079556699 | 26.61% | k\_\_Bacteria;p\_\_Firmicutes;c\_\_Clostridia |
| 0.0868084023635 | 8.68% | k\_\_Bacteria;p\_\_Verrucomicrobia;c\_\_Verrucomicrobiae |
| 0.0298691018066 | 2.99% | k\_\_Bacteria;p\_\_Proteobacteria;c\_\_Gammaproteobacteria |
| 0.0251901275056 | 2.52% | k\_\_Bacteria;p\_\_Tenericutes;c\_\_Mollicutes |
| 0.0103474597834 | 1.03% | k\_\_Bacteria;p\_\_Firmicutes;c\_\_Erysipelotrichia |
| 0.0072375674988 | 0.72% | k\_\_Bacteria;p\_\_Firmicutes;c\_\_Bacilli |
| 0.00453761556077 | 0.45% | k\_\_Bacteria;p\_\_Proteobacteria;c\_\_Deltaproteobacteria |
| 0.00166803313449 | 0.17% | k\_\_Bacteria;p\_\_Melainabacteria;Other |
| 0.0012722286619 | 0.13% | k\_\_Bacteria;Other;Other |
| 0.00104605467756 | 0.10% | k\_\_Bacteria;p\_\_Oxyphotobacteria;c\_\_unidentified\_Oxyphotobacteria |
| 0.00065025020497 | 0.07% | k\_\_Bacteria;p\_\_unidentified\_Bacteria;c\_\_unidentified\_Bacteria |
| 0.000537163212801 | 0.05% | k\_\_Bacteria;p\_\_Actinobacteria;c\_\_unidentified\_Actinobacteria |
| 0.000409940346612 | 0.04% | k\_\_Bacteria;p\_\_Firmicutes;c\_\_Negativicutes |
| 0.000395804472591 | 0.04% | k\_\_Bacteria;p\_\_Fusobacteria;c\_\_Fusobacteriia |
| 0.000183766362274 | 0.02% | k\_\_Bacteria;p\_\_Firmicutes;Other |
| 0.000169630488253 | 0.02% | k\_\_Bacteria;p\_\_Proteobacteria;c\_\_Alphaproteobacteria |
| 2.82717480422e-05 | 0.00% | k\_\_Bacteria;p\_\_Armatimonadetes;c\_\_Fimbriimonadia |

|  |  |
| --- | --- |
|  | |
| Taxonomy Summary. Current Level: \_MC2 | |
| View Figure (.pdf)  View Legend (.pdf) |  |
|  |  |

|  |  |  |
| --- | --- | --- |
| Count | Pct | Taxonomy |
| 0.644723078228 | 64.47% | k\_\_Bacteria;p\_\_Bacteroidetes;c\_\_Bacteroidia |
| 0.219968335642 | 22.00% | k\_\_Bacteria;p\_\_Firmicutes;c\_\_Clostridia |
| 0.0834440643465 | 8.34% | k\_\_Bacteria;p\_\_Proteobacteria;c\_\_Gammaproteobacteria |
| 0.0138107489186 | 1.38% | k\_\_Bacteria;p\_\_Firmicutes;c\_\_Bacilli |
| 0.0128636453592 | 1.29% | k\_\_Bacteria;p\_\_Proteobacteria;c\_\_Deltaproteobacteria |
| 0.0124678408866 | 1.25% | k\_\_Bacteria;p\_\_Verrucomicrobia;c\_\_Verrucomicrobiae |
| 0.00568262135648 | 0.57% | k\_\_Bacteria;p\_\_Firmicutes;c\_\_Erysipelotrichia |
| 0.00385909360776 | 0.39% | k\_\_Bacteria;p\_\_Tenericutes;c\_\_Mollicutes |
| 0.00114500579571 | 0.11% | k\_\_Bacteria;p\_\_unidentified\_Bacteria;c\_\_unidentified\_Bacteria |
| 0.00052302733878 | 0.05% | k\_\_Bacteria;p\_\_Actinobacteria;c\_\_unidentified\_Actinobacteria |
| 0.000325125102485 | 0.03% | k\_\_Bacteria;p\_\_Firmicutes;c\_\_Negativicutes |
| 0.000310989228464 | 0.03% | k\_\_Bacteria;p\_\_Melainabacteria;Other |
| 0.000282717480422 | 0.03% | k\_\_Bacteria;p\_\_Fusobacteria;c\_\_Fusobacteriia |
| 0.00025444573238 | 0.03% | k\_\_Bacteria;Other;Other |
| 0.000240309858359 | 0.02% | k\_\_Bacteria;p\_\_Firmicutes;Other |
| 5.65434960844e-05 | 0.01% | k\_\_Bacteria;p\_\_Proteobacteria;c\_\_Alphaproteobacteria |
| 1.41358740211e-05 | 0.00% | k\_\_Bacteria;p\_\_Oxyphotobacteria;c\_\_unidentified\_Oxyphotobacteria |
| 1.41358740211e-05 | 0.00% | k\_\_Bacteria;p\_\_Actinobacteria;c\_\_Rubrobacteria |
| 1.41358740211e-05 | 0.00% | k\_\_Archaea;p\_\_Euryarchaeota;c\_\_Methanobacteria |

|  |  |
| --- | --- |
|  | |
| Taxonomy Summary. Current Level: \_MC3 | |
| View Figure (.pdf)  View Legend (.pdf) |  |
|  |  |
| View Figure (.pdf)  View Legend (.pdf) |  |
|  |  |
|  |  |

|  |  |  |
| --- | --- | --- |
| Count | Pct | Taxonomy |
| 0.614557123067 | 61.46% | k\_\_Bacteria;p\_\_Bacteroidetes;c\_\_Bacteroidia |
| 0.295241864805 | 29.52% | k\_\_Bacteria;p\_\_Firmicutes;c\_\_Clostridia |
| 0.0434960843629 | 4.35% | k\_\_Bacteria;p\_\_Proteobacteria;c\_\_Gammaproteobacteria |
| 0.018786576574 | 1.88% | k\_\_Bacteria;p\_\_Proteobacteria;c\_\_Deltaproteobacteria |
| 0.0128919171072 | 1.29% | k\_\_Bacteria;p\_\_Firmicutes;c\_\_Bacilli |
| 0.00689830652229 | 0.69% | k\_\_Bacteria;p\_\_Firmicutes;c\_\_Erysipelotrichia |
| 0.00318057165475 | 0.32% | k\_\_Bacteria;p\_\_Tenericutes;c\_\_Mollicutes |
| 0.00114500579571 | 0.11% | k\_\_Bacteria;p\_\_Verrucomicrobia;c\_\_Verrucomicrobiae |
| 0.000735065449097 | 0.07% | k\_\_Bacteria;p\_\_unidentified\_Bacteria;c\_\_unidentified\_Bacteria |
| 0.000706793701055 | 0.07% | k\_\_Bacteria;Other;Other |
| 0.000508891464759 | 0.05% | k\_\_Bacteria;p\_\_Firmicutes;c\_\_Negativicutes |
| 0.000452347968675 | 0.05% | k\_\_Bacteria;p\_\_Firmicutes;Other |
| 0.000395804472591 | 0.04% | k\_\_Bacteria;p\_\_Actinobacteria;c\_\_unidentified\_Actinobacteria |
| 0.000268581606401 | 0.03% | k\_\_Bacteria;p\_\_Fusobacteria;c\_\_Fusobacteriia |
| 0.000226173984337 | 0.02% | k\_\_Bacteria;p\_\_Proteobacteria;c\_\_Alphaproteobacteria |
| 0.000155494614232 | 0.02% | k\_\_Bacteria;p\_\_Melainabacteria;Other |
| 0.000141358740211 | 0.01% | k\_\_Bacteria;p\_\_Actinobacteria;c\_\_Thermoleophilia |
| 5.65434960844e-05 | 0.01% | k\_\_Bacteria;p\_\_Oxyphotobacteria;c\_\_unidentified\_Oxyphotobacteria |
| 5.65434960844e-05 | 0.01% | k\_\_Archaea;p\_\_Euryarchaeota;c\_\_Methanobacteria |
| 4.24076220633e-05 | 0.00% | k\_\_Bacteria;p\_\_Actinobacteria;c\_\_Rubrobacteria |
| 2.82717480422e-05 | 0.00% | k\_\_Bacteria;p\_\_Acidobacteria;c\_\_unidentified\_Acidobacteria |
| 1.41358740211e-05 | 0.00% | k\_\_Bacteria;p\_\_Deinococcus-Thermus;c\_\_Deinococci |
| 1.41358740211e-05 | 0.00% | k\_\_Bacteria;p\_\_Actinobacteria;c\_\_Coriobacteriia |

|  |  |
| --- | --- |
|  | |
| Taxonomy Summary. Current Level: \_MC4 | |
| View Figure (.pdf)  View Legend (.pdf) |  |
|  |  |

|  |  |  |
| --- | --- | --- |
| Count | Pct | Taxonomy |
| 0.577733171242 | 57.77% | k\_\_Bacteria;p\_\_Firmicutes;c\_\_Clostridia |
| 0.387238132934 | 38.72% | k\_\_Bacteria;p\_\_Bacteroidetes;c\_\_Bacteroidia |
| 0.0117469113115 | 1.17% | k\_\_Bacteria;p\_\_Proteobacteria;c\_\_Gammaproteobacteria |
| 0.00838257329451 | 0.84% | k\_\_Bacteria;p\_\_Proteobacteria;c\_\_Deltaproteobacteria |
| 0.00506064289955 | 0.51% | k\_\_Bacteria;p\_\_Firmicutes;c\_\_Bacilli |
| 0.00248791382771 | 0.25% | k\_\_Bacteria;p\_\_Firmicutes;c\_\_Erysipelotrichia |
| 0.00188007124481 | 0.19% | k\_\_Bacteria;Other;Other |
| 0.00130050040994 | 0.13% | k\_\_Bacteria;p\_\_Verrucomicrobia;c\_\_Verrucomicrobiae |
| 0.000862288315287 | 0.09% | k\_\_Bacteria;p\_\_Firmicutes;Other |
| 0.000819880693223 | 0.08% | k\_\_Bacteria;p\_\_Tenericutes;c\_\_Mollicutes |
| 0.00077747307116 | 0.08% | k\_\_Bacteria;p\_\_Firmicutes;c\_\_Negativicutes |
| 0.000565434960844 | 0.06% | k\_\_Bacteria;p\_\_unidentified\_Bacteria;c\_\_unidentified\_Bacteria |
| 0.000339260976506 | 0.03% | k\_\_Bacteria;p\_\_Actinobacteria;c\_\_unidentified\_Actinobacteria |
| 0.000325125102485 | 0.03% | k\_\_Bacteria;p\_\_Melainabacteria;Other |
| 0.000310989228464 | 0.03% | k\_\_Bacteria;p\_\_Fusobacteria;c\_\_Fusobacteriia |
| 0.00012722286619 | 0.01% | k\_\_Bacteria;p\_\_Proteobacteria;c\_\_Alphaproteobacteria |
| 4.24076220633e-05 | 0.00% | k\_\_Bacteria;p\_\_Oxyphotobacteria;c\_\_unidentified\_Oxyphotobacteria |

|  |  |
| --- | --- |
|  | |
| Taxonomy Summary. Current Level: \_MC5 | |
| View Figure (.pdf)  View Legend (.pdf) |  |
|  |  |

|  |  |  |
| --- | --- | --- |
| Count | Pct | Taxonomy |
| 0.51364111843 | 51.36% | k\_\_Bacteria;p\_\_Bacteroidetes;c\_\_Bacteroidia |
| 0.393316558763 | 39.33% | k\_\_Bacteria;p\_\_Firmicutes;c\_\_Clostridia |
| 0.0486839501286 | 4.87% | k\_\_Bacteria;p\_\_Proteobacteria;c\_\_Gammaproteobacteria |
| 0.0141782816432 | 1.42% | k\_\_Bacteria;p\_\_Verrucomicrobia;c\_\_Verrucomicrobiae |
| 0.0101919651692 | 1.02% | k\_\_Bacteria;p\_\_Proteobacteria;c\_\_Deltaproteobacteria |
| 0.00773232308954 | 0.77% | k\_\_Bacteria;p\_\_Firmicutes;c\_\_Bacilli |
| 0.00460829493088 | 0.46% | k\_\_Bacteria;p\_\_Tenericutes;c\_\_Mollicutes |
| 0.00221933222131 | 0.22% | k\_\_Bacteria;p\_\_Firmicutes;c\_\_Erysipelotrichia |
| 0.00144185915015 | 0.14% | k\_\_Bacteria;p\_\_Melainabacteria;Other |
| 0.0010743264256 | 0.11% | k\_\_Bacteria;Other;Other |
| 0.000862288315287 | 0.09% | k\_\_Bacteria;p\_\_unidentified\_Bacteria;c\_\_unidentified\_Bacteria |
| 0.000664386078991 | 0.07% | k\_\_Bacteria;p\_\_Actinobacteria;c\_\_unidentified\_Actinobacteria |
| 0.000607842582907 | 0.06% | k\_\_Bacteria;p\_\_Firmicutes;c\_\_Negativicutes |
| 0.000339260976506 | 0.03% | k\_\_Bacteria;p\_\_Fusobacteria;c\_\_Fusobacteriia |
| 0.000310989228464 | 0.03% | k\_\_Bacteria;p\_\_Firmicutes;Other |
| 5.65434960844e-05 | 0.01% | k\_\_Bacteria;p\_\_Proteobacteria;c\_\_Alphaproteobacteria |
| 2.82717480422e-05 | 0.00% | k\_\_Bacteria;p\_\_Acidobacteria;c\_\_unidentified\_Acidobacteria |
| 2.82717480422e-05 | 0.00% | k\_\_Bacteria;p\_\_Acidobacteria;c\_\_Holophagae |
| 1.41358740211e-05 | 0.00% | k\_\_Bacteria;p\_\_Deinococcus-Thermus;c\_\_Deinococci |

|  |  |
| --- | --- |
|  | |
| Taxonomy Summary. Current Level: | |
| View Figure (.pdf)  View Legend (.pdf) |  |
|  |  |
| View Figure (.pdf)  View Legend (.pdf) |  |
|  |  |
|  |  |

|  |  |  |
| --- | --- | --- |
| Count | Pct | Taxonomy |
| 5.2833818665 | 52.83% | k\_\_Bacteria;p\_\_Bacteroidetes;c\_\_Bacteroidia;o\_\_Bacteroidales |
| 3.66468293235 | 36.65% | k\_\_Bacteria;p\_\_Firmicutes;c\_\_Clostridia;o\_\_Clostridiales |
| 0.569901897034 | 5.70% | k\_\_Bacteria;p\_\_Proteobacteria;c\_\_Gammaproteobacteria;o\_\_Enterobacteriales |
| 0.135718526477 | 1.36% | k\_\_Bacteria;p\_\_Verrucomicrobia;c\_\_Verrucomicrobiae;o\_\_Verrucomicrobiales |
| 0.1115461819 | 1.12% | k\_\_Bacteria;p\_\_Proteobacteria;c\_\_Deltaproteobacteria;o\_\_Desulfovibrionales |
| 0.0995306889825 | 1.00% | k\_\_Bacteria;p\_\_Firmicutes;c\_\_Bacilli;o\_\_Lactobacillales |
| 0.0450510305052 | 0.45% | k\_\_Bacteria;p\_\_Tenericutes;c\_\_Mollicutes;o\_\_ |
| 0.0404427355743 | 0.40% | k\_\_Bacteria;p\_\_Firmicutes;c\_\_Erysipelotrichia;o\_\_Erysipelotrichales |
| 0.00845325266461 | 0.08% | k\_\_Bacteria;Other;Other;Other |
| 0.00503237115151 | 0.05% | k\_\_Bacteria;p\_\_Proteobacteria;c\_\_Gammaproteobacteria;o\_\_unidentified\_Gammaproteobacteria |
| 0.00490514828532 | 0.05% | k\_\_Bacteria;p\_\_unidentified\_Bacteria;c\_\_unidentified\_Bacteria;o\_\_unidentified\_Bacteria |
| 0.00487687653728 | 0.05% | k\_\_Bacteria;p\_\_Firmicutes;c\_\_Negativicutes;o\_\_Selenomonadales |
| 0.00463656667892 | 0.05% | k\_\_Bacteria;p\_\_Melainabacteria;Other;Other |
| 0.00400045234797 | 0.04% | k\_\_Bacteria;p\_\_Oxyphotobacteria;c\_\_unidentified\_Oxyphotobacteria;o\_\_unidentified\_Oxyphotobacteria |
| 0.00347742500919 | 0.03% | k\_\_Bacteria;p\_\_Firmicutes;Other;Other |
| 0.00295439767041 | 0.03% | k\_\_Bacteria;p\_\_Fusobacteria;c\_\_Fusobacteriia;o\_\_Fusobacteriales |
| 0.00269995193803 | 0.03% | k\_\_Bacteria;p\_\_Actinobacteria;c\_\_unidentified\_Actinobacteria;o\_\_Bifidobacteriales |
| 0.00206383760708 | 0.02% | k\_\_Bacteria;p\_\_Proteobacteria;c\_\_Gammaproteobacteria;o\_\_Pseudomonadales |
| 0.00115914166973 | 0.01% | k\_\_Bacteria;p\_\_unidentified\_Bacteria;c\_\_unidentified\_Bacteria;o\_\_Campylobacterales |
| 0.000777473071161 | 0.01% | k\_\_Bacteria;p\_\_Actinobacteria;c\_\_unidentified\_Actinobacteria;o\_\_Corynebacteriales |
| 0.000749201323118 | 0.01% | k\_\_Bacteria;p\_\_Proteobacteria;c\_\_Gammaproteobacteria;o\_\_Pasteurellales |
| 0.000480619716717 | 0.00% | k\_\_Bacteria;p\_\_Proteobacteria;c\_\_Alphaproteobacteria;o\_\_Rhizobiales |
| 0.000438212094654 | 0.00% | k\_\_Bacteria;p\_\_Proteobacteria;c\_\_Gammaproteobacteria;o\_\_Xanthomonadales |
| 0.000409940346612 | 0.00% | k\_\_Bacteria;p\_\_Firmicutes;c\_\_Bacilli;o\_\_Bacillales |
| 0.000395804472591 | 0.00% | k\_\_Bacteria;p\_\_Proteobacteria;c\_\_Alphaproteobacteria;o\_\_Sphingomonadales |
| 0.00025444573238 | 0.00% | k\_\_Bacteria;p\_\_Proteobacteria;c\_\_Alphaproteobacteria;o\_\_Rhodospirillales |
| 0.000226173984338 | 0.00% | k\_\_Bacteria;p\_\_Actinobacteria;c\_\_Thermoleophilia;o\_\_Solirubrobacterales |
| 0.000212038110316 | 0.00% | k\_\_Bacteria;p\_\_Actinobacteria;c\_\_Rubrobacteria;o\_\_Rubrobacterales |
| 0.000212038110316 | 0.00% | k\_\_Bacteria;p\_\_Bacteroidetes;c\_\_Bacteroidia;o\_\_Flavobacteriales |
| 0.000197902236295 | 0.00% | k\_\_Bacteria;p\_\_Proteobacteria;c\_\_Alphaproteobacteria;o\_\_unidentified\_Alphaproteobacteria |
| 0.000169630488253 | 0.00% | k\_\_Bacteria;p\_\_Acidobacteria;c\_\_unidentified\_Acidobacteria;o\_\_unidentified\_Acidobacteria |
| 0.00012722286619 | 0.00% | k\_\_Bacteria;p\_\_Actinobacteria;c\_\_unidentified\_Actinobacteria;o\_\_Actinomycetales |
| 8.48152441266e-05 | 0.00% | k\_\_Bacteria;p\_\_Actinobacteria;c\_\_unidentified\_Actinobacteria;o\_\_Micrococcales |
| 8.48152441266e-05 | 0.00% | k\_\_Bacteria;p\_\_Acidobacteria;c\_\_Holophagae;o\_\_ |
| 7.06793701055e-05 | 0.00% | k\_\_Bacteria;p\_\_Bacteroidetes;c\_\_Bacteroidia;o\_\_Chitinophagales |
| 7.06793701055e-05 | 0.00% | k\_\_Bacteria;p\_\_Actinobacteria;c\_\_Coriobacteriia;o\_\_Coriobacteriales |
| 7.06793701055e-05 | 0.00% | k\_\_Archaea;p\_\_Euryarchaeota;c\_\_Methanobacteria;o\_\_Methanobacteriales |
| 5.65434960844e-05 | 0.00% | k\_\_Bacteria;p\_\_Actinobacteria;c\_\_unidentified\_Actinobacteria;o\_\_Propionibacteriales |
| 5.65434960844e-05 | 0.00% | k\_\_Bacteria;p\_\_Actinobacteria;c\_\_Acidimicrobiia;Other |
| 4.24076220633e-05 | 0.00% | k\_\_Bacteria;p\_\_Proteobacteria;Other;Other |
| 4.24076220633e-05 | 0.00% | k\_\_Bacteria;p\_\_Bacteroidetes;c\_\_Bacteroidia;o\_\_Cytophagales |
| 4.24076220633e-05 | 0.00% | k\_\_Bacteria;p\_\_Tenericutes;c\_\_Mollicutes;o\_\_Anaeroplasmatales |
| 2.82717480422e-05 | 0.00% | k\_\_Bacteria;p\_\_Verrucomicrobia;c\_\_Verrucomicrobiae;o\_\_Chthoniobacterales |
| 2.82717480422e-05 | 0.00% | k\_\_Bacteria;p\_\_Proteobacteria;c\_\_Gammaproteobacteria;o\_\_Alteromonadales |
| 2.82717480422e-05 | 0.00% | k\_\_Bacteria;p\_\_Proteobacteria;c\_\_Alphaproteobacteria;o\_\_Caulobacterales |
| 2.82717480422e-05 | 0.00% | k\_\_Bacteria;p\_\_Deinococcus-Thermus;c\_\_Deinococci;o\_\_Deinococcales |
| 2.82717480422e-05 | 0.00% | k\_\_Bacteria;p\_\_Bacteroidetes;c\_\_Bacteroidia;o\_\_Sphingobacteriales |
| 2.82717480422e-05 | 0.00% | k\_\_Bacteria;p\_\_Armatimonadetes;c\_\_Fimbriimonadia;o\_\_Fimbriimonadales |
| 2.82717480422e-05 | 0.00% | k\_\_Bacteria;p\_\_Actinobacteria;c\_\_Acidimicrobiia;o\_\_ |
| 1.41358740211e-05 | 0.00% | k\_\_Bacteria;p\_\_Actinobacteria;c\_\_Thermoleophilia;o\_\_Gaiellales |

|  |  |
| --- | --- |
|  | |
| Taxonomy Summary. Current Level: \_MD1 | |
| View Figure (.pdf)  View Legend (.pdf) |  |
|  |  |
| View Figure (.pdf)  View Legend (.pdf) |  |
|  |  |
|  |  |

|  |  |  |
| --- | --- | --- |
| Count | Pct | Taxonomy |
| 0.468717310791 | 46.87% | k\_\_Bacteria;p\_\_Firmicutes;c\_\_Clostridia;o\_\_Clostridiales |
| 0.430168782336 | 43.02% | k\_\_Bacteria;p\_\_Bacteroidetes;c\_\_Bacteroidia;o\_\_Bacteroidales |
| 0.0776200842498 | 7.76% | k\_\_Bacteria;p\_\_Proteobacteria;c\_\_Gammaproteobacteria;o\_\_Enterobacteriales |
| 0.00993751943683 | 0.99% | k\_\_Bacteria;p\_\_Proteobacteria;c\_\_Deltaproteobacteria;o\_\_Desulfovibrionales |
| 0.00554126261627 | 0.55% | k\_\_Bacteria;p\_\_Firmicutes;c\_\_Bacilli;o\_\_Lactobacillales |
| 0.00244550620565 | 0.24% | k\_\_Bacteria;p\_\_Firmicutes;c\_\_Erysipelotrichia;o\_\_Erysipelotrichales |
| 0.00166803313449 | 0.17% | k\_\_Bacteria;p\_\_Verrucomicrobia;c\_\_Verrucomicrobiae;o\_\_Verrucomicrobiales |
| 0.000819880693223 | 0.08% | k\_\_Bacteria;p\_\_Tenericutes;c\_\_Mollicutes;o\_\_ |
| 0.000664386078991 | 0.07% | k\_\_Bacteria;p\_\_Proteobacteria;c\_\_Gammaproteobacteria;o\_\_unidentified\_Gammaproteobacteria |
| 0.000409940346612 | 0.04% | k\_\_Bacteria;p\_\_Firmicutes;c\_\_Negativicutes;o\_\_Selenomonadales |
| 0.000353396850527 | 0.04% | k\_\_Bacteria;Other;Other;Other |
| 0.000296853354443 | 0.03% | k\_\_Bacteria;p\_\_Proteobacteria;c\_\_Gammaproteobacteria;o\_\_Pseudomonadales |
| 0.000296853354443 | 0.03% | k\_\_Bacteria;p\_\_Fusobacteria;c\_\_Fusobacteriia;o\_\_Fusobacteriales |
| 0.000240309858359 | 0.02% | k\_\_Bacteria;p\_\_Actinobacteria;c\_\_unidentified\_Actinobacteria;o\_\_Bifidobacteriales |
| 0.000212038110316 | 0.02% | k\_\_Bacteria;p\_\_Firmicutes;Other;Other |
| 0.000113086992169 | 0.01% | k\_\_Bacteria;p\_\_unidentified\_Bacteria;c\_\_unidentified\_Bacteria;o\_\_Campylobacterales |
| 9.89511181476e-05 | 0.01% | k\_\_Bacteria;p\_\_Melainabacteria;Other;Other |
| 8.48152441265e-05 | 0.01% | k\_\_Bacteria;p\_\_Proteobacteria;c\_\_Gammaproteobacteria;o\_\_Xanthomonadales |
| 7.06793701055e-05 | 0.01% | k\_\_Bacteria;p\_\_Firmicutes;c\_\_Bacilli;o\_\_Bacillales |
| 5.65434960844e-05 | 0.01% | k\_\_Bacteria;p\_\_unidentified\_Bacteria;c\_\_unidentified\_Bacteria;o\_\_unidentified\_Bacteria |
| 4.24076220633e-05 | 0.00% | k\_\_Bacteria;p\_\_Proteobacteria;c\_\_Gammaproteobacteria;o\_\_Pasteurellales |
| 4.24076220633e-05 | 0.00% | k\_\_Bacteria;p\_\_Proteobacteria;c\_\_Alphaproteobacteria;o\_\_Rhizobiales |
| 2.82717480422e-05 | 0.00% | k\_\_Bacteria;p\_\_Proteobacteria;c\_\_Alphaproteobacteria;o\_\_Sphingomonadales |
| 2.82717480422e-05 | 0.00% | k\_\_Bacteria;p\_\_Oxyphotobacteria;c\_\_unidentified\_Oxyphotobacteria;o\_\_unidentified\_Oxyphotobacteria |
| 1.41358740211e-05 | 0.00% | k\_\_Bacteria;p\_\_Actinobacteria;c\_\_unidentified\_Actinobacteria;o\_\_Corynebacteriales |
| 1.41358740211e-05 | 0.00% | k\_\_Bacteria;p\_\_Actinobacteria;c\_\_unidentified\_Actinobacteria;o\_\_Actinomycetales |
| 1.41358740211e-05 | 0.00% | k\_\_Bacteria;p\_\_Actinobacteria;c\_\_Thermoleophilia;o\_\_Solirubrobacterales |

|  |  |
| --- | --- |
|  | |
| Taxonomy Summary. Current Level: \_MD2 | |
| View Figure (.pdf)  View Legend (.pdf) |  |
|  |  |
| View Figure (.pdf)  View Legend (.pdf) |  |
|  |  |
|  |  |

|  |  |  |
| --- | --- | --- |
| Count | Pct | Taxonomy |
| 0.474852280116 | 47.49% | k\_\_Bacteria;p\_\_Bacteroidetes;c\_\_Bacteroidia;o\_\_Bacteroidales |
| 0.399239489978 | 39.92% | k\_\_Bacteria;p\_\_Firmicutes;c\_\_Clostridia;o\_\_Clostridiales |
| 0.091854909389 | 9.19% | k\_\_Bacteria;p\_\_Proteobacteria;c\_\_Gammaproteobacteria;o\_\_Enterobacteriales |
| 0.0117751830596 | 1.18% | k\_\_Bacteria;p\_\_Proteobacteria;c\_\_Deltaproteobacteria;o\_\_Desulfovibrionales |
| 0.00732238274292 | 0.73% | k\_\_Bacteria;p\_\_Firmicutes;c\_\_Bacilli;o\_\_Lactobacillales |
| 0.00407113171807 | 0.41% | k\_\_Bacteria;p\_\_Verrucomicrobia;c\_\_Verrucomicrobiae;o\_\_Verrucomicrobiales |
| 0.00296853354443 | 0.30% | k\_\_Bacteria;p\_\_Firmicutes;c\_\_Erysipelotrichia;o\_\_Erysipelotrichales |
| 0.00190834299285 | 0.19% | k\_\_Bacteria;p\_\_Oxyphotobacteria;c\_\_unidentified\_Oxyphotobacteria;o\_\_unidentified\_Oxyphotobacteria |
| 0.00171044075655 | 0.17% | k\_\_Bacteria;Other;Other;Other |
| 0.001342908032 | 0.13% | k\_\_Bacteria;p\_\_Tenericutes;c\_\_Mollicutes;o\_\_ |
| 0.000735065449097 | 0.07% | k\_\_Bacteria;p\_\_Proteobacteria;c\_\_Gammaproteobacteria;o\_\_unidentified\_Gammaproteobacteria |
| 0.000325125102485 | 0.03% | k\_\_Bacteria;p\_\_Firmicutes;c\_\_Negativicutes;o\_\_Selenomonadales |
| 0.000268581606401 | 0.03% | k\_\_Bacteria;p\_\_Proteobacteria;c\_\_Gammaproteobacteria;o\_\_Pseudomonadales |
| 0.00025444573238 | 0.03% | k\_\_Bacteria;p\_\_unidentified\_Bacteria;c\_\_unidentified\_Bacteria;o\_\_unidentified\_Bacteria |
| 0.00025444573238 | 0.03% | k\_\_Bacteria;p\_\_Fusobacteria;c\_\_Fusobacteriia;o\_\_Fusobacteriales |
| 0.00025444573238 | 0.03% | k\_\_Bacteria;p\_\_Firmicutes;Other;Other |
| 0.00025444573238 | 0.03% | k\_\_Bacteria;p\_\_Actinobacteria;c\_\_unidentified\_Actinobacteria;o\_\_Bifidobacteriales |
| 0.000169630488253 | 0.02% | k\_\_Bacteria;p\_\_Melainabacteria;Other;Other |
| 0.000141358740211 | 0.01% | k\_\_Bacteria;p\_\_unidentified\_Bacteria;c\_\_unidentified\_Bacteria;o\_\_Campylobacterales |
| 5.65434960844e-05 | 0.01% | k\_\_Bacteria;p\_\_Proteobacteria;c\_\_Gammaproteobacteria;o\_\_Pasteurellales |
| 5.65434960844e-05 | 0.01% | k\_\_Bacteria;p\_\_Actinobacteria;c\_\_unidentified\_Actinobacteria;o\_\_Corynebacteriales |
| 4.24076220633e-05 | 0.00% | k\_\_Bacteria;p\_\_Proteobacteria;c\_\_Alphaproteobacteria;o\_\_Rhodospirillales |
| 4.24076220633e-05 | 0.00% | k\_\_Bacteria;p\_\_Actinobacteria;c\_\_Coriobacteriia;o\_\_Coriobacteriales |
| 2.82717480422e-05 | 0.00% | k\_\_Bacteria;p\_\_Proteobacteria;c\_\_Alphaproteobacteria;o\_\_Rhizobiales |
| 2.82717480422e-05 | 0.00% | k\_\_Bacteria;p\_\_Firmicutes;c\_\_Bacilli;o\_\_Bacillales |
| 2.82717480422e-05 | 0.00% | k\_\_Bacteria;p\_\_Bacteroidetes;c\_\_Bacteroidia;o\_\_Flavobacteriales |
| 1.41358740211e-05 | 0.00% | k\_\_Bacteria;p\_\_Tenericutes;c\_\_Mollicutes;o\_\_Anaeroplasmatales |

|  |  |
| --- | --- |
|  | |
| Taxonomy Summary. Current Level: \_MD3 | |
| View Figure (.pdf)  View Legend (.pdf) |  |
|  |  |
| View Figure (.pdf)  View Legend (.pdf) |  |
|  |  |
|  |  |

|  |  |  |
| --- | --- | --- |
| Count | Pct | Taxonomy |
| 0.471586893218 | 47.16% | k\_\_Bacteria;p\_\_Bacteroidetes;c\_\_Bacteroidia;o\_\_Bacteroidales |
| 0.401091289474 | 40.11% | k\_\_Bacteria;p\_\_Firmicutes;c\_\_Clostridia;o\_\_Clostridiales |
| 0.0923779367278 | 9.24% | k\_\_Bacteria;p\_\_Proteobacteria;c\_\_Gammaproteobacteria;o\_\_Enterobacteriales |
| 0.01342908032 | 1.34% | k\_\_Bacteria;p\_\_Proteobacteria;c\_\_Deltaproteobacteria;o\_\_Desulfovibrionales |
| 0.0075768284753 | 0.76% | k\_\_Bacteria;p\_\_Firmicutes;c\_\_Bacilli;o\_\_Lactobacillales |
| 0.00460829493088 | 0.46% | k\_\_Bacteria;p\_\_Firmicutes;c\_\_Erysipelotrichia;o\_\_Erysipelotrichales |
| 0.00337847389104 | 0.34% | k\_\_Bacteria;p\_\_Verrucomicrobia;c\_\_Verrucomicrobiae;o\_\_Verrucomicrobiales |
| 0.000932967685392 | 0.09% | k\_\_Bacteria;Other;Other;Other |
| 0.000819880693223 | 0.08% | k\_\_Bacteria;p\_\_Tenericutes;c\_\_Mollicutes;o\_\_ |
| 0.000819880693223 | 0.08% | k\_\_Bacteria;p\_\_Oxyphotobacteria;c\_\_unidentified\_Oxyphotobacteria;o\_\_unidentified\_Oxyphotobacteria |
| 0.000466483842696 | 0.05% | k\_\_Bacteria;p\_\_Proteobacteria;c\_\_Gammaproteobacteria;o\_\_unidentified\_Gammaproteobacteria |
| 0.000438212094654 | 0.04% | k\_\_Bacteria;p\_\_Firmicutes;c\_\_Negativicutes;o\_\_Selenomonadales |
| 0.000296853354443 | 0.03% | k\_\_Bacteria;p\_\_Firmicutes;Other;Other |
| 0.000212038110316 | 0.02% | k\_\_Bacteria;p\_\_Proteobacteria;c\_\_Gammaproteobacteria;o\_\_Pseudomonadales |
| 0.000212038110316 | 0.02% | k\_\_Bacteria;p\_\_Fusobacteria;c\_\_Fusobacteriia;o\_\_Fusobacteriales |
| 0.000183766362274 | 0.02% | k\_\_Bacteria;p\_\_unidentified\_Bacteria;c\_\_unidentified\_Bacteria;o\_\_Campylobacterales |
| 0.000183766362274 | 0.02% | k\_\_Bacteria;p\_\_Proteobacteria;c\_\_Alphaproteobacteria;o\_\_unidentified\_Alphaproteobacteria |
| 0.000155494614232 | 0.02% | k\_\_Bacteria;p\_\_Actinobacteria;c\_\_Rubrobacteria;o\_\_Rubrobacterales |
| 0.000141358740211 | 0.01% | k\_\_Bacteria;p\_\_Proteobacteria;c\_\_Gammaproteobacteria;o\_\_Pasteurellales |
| 0.000141358740211 | 0.01% | k\_\_Bacteria;p\_\_Actinobacteria;c\_\_unidentified\_Actinobacteria;o\_\_Bifidobacteriales |
| 0.00012722286619 | 0.01% | k\_\_Bacteria;p\_\_unidentified\_Bacteria;c\_\_unidentified\_Bacteria;o\_\_unidentified\_Bacteria |
| 0.000113086992169 | 0.01% | k\_\_Bacteria;p\_\_Acidobacteria;c\_\_unidentified\_Acidobacteria;o\_\_unidentified\_Acidobacteria |
| 8.48152441265e-05 | 0.01% | k\_\_Bacteria;p\_\_Proteobacteria;c\_\_Alphaproteobacteria;o\_\_Rhizobiales |
| 8.48152441265e-05 | 0.01% | k\_\_Bacteria;p\_\_Melainabacteria;Other;Other |
| 8.48152441265e-05 | 0.01% | k\_\_Bacteria;p\_\_Actinobacteria;c\_\_Thermoleophilia;o\_\_Solirubrobacterales |
| 5.65434960844e-05 | 0.01% | k\_\_Bacteria;p\_\_Proteobacteria;c\_\_Alphaproteobacteria;o\_\_Sphingomonadales |
| 5.65434960844e-05 | 0.01% | k\_\_Bacteria;p\_\_Actinobacteria;c\_\_unidentified\_Actinobacteria;o\_\_Propionibacteriales |
| 5.65434960844e-05 | 0.01% | k\_\_Bacteria;p\_\_Actinobacteria;c\_\_Acidimicrobiia;Other |
| 5.65434960844e-05 | 0.01% | k\_\_Bacteria;p\_\_Acidobacteria;c\_\_Holophagae;o\_\_ |
| 4.24076220633e-05 | 0.00% | k\_\_Bacteria;p\_\_Proteobacteria;c\_\_Gammaproteobacteria;o\_\_Xanthomonadales |
| 4.24076220633e-05 | 0.00% | k\_\_Bacteria;p\_\_Proteobacteria;c\_\_Alphaproteobacteria;o\_\_Rhodospirillales |
| 4.24076220633e-05 | 0.00% | k\_\_Bacteria;p\_\_Bacteroidetes;c\_\_Bacteroidia;o\_\_Cytophagales |
| 4.24076220633e-05 | 0.00% | k\_\_Bacteria;p\_\_Actinobacteria;c\_\_unidentified\_Actinobacteria;o\_\_Corynebacteriales |
| 2.82717480422e-05 | 0.00% | k\_\_Bacteria;p\_\_Actinobacteria;c\_\_Acidimicrobiia;o\_\_ |
| 1.41358740211e-05 | 0.00% | k\_\_Bacteria;p\_\_Firmicutes;c\_\_Bacilli;o\_\_Bacillales |
| 1.41358740211e-05 | 0.00% | k\_\_Bacteria;p\_\_Actinobacteria;c\_\_unidentified\_Actinobacteria;o\_\_Actinomycetales |

|  |  |
| --- | --- |
|  | |
| Taxonomy Summary. Current Level: \_MD4 | |
| View Figure (.pdf)  View Legend (.pdf) |  |
|  |  |
| View Figure (.pdf)  View Legend (.pdf) |  |
|  |  |
|  |  |

|  |  |  |
| --- | --- | --- |
| Count | Pct | Taxonomy |
| 0.682932345707 | 68.29% | k\_\_Bacteria;p\_\_Bacteroidetes;c\_\_Bacteroidia;o\_\_Bacteroidales |
| 0.208617228803 | 20.86% | k\_\_Bacteria;p\_\_Firmicutes;c\_\_Clostridia;o\_\_Clostridiales |
| 0.0753300726584 | 7.53% | k\_\_Bacteria;p\_\_Proteobacteria;c\_\_Gammaproteobacteria;o\_\_Enterobacteriales |
| 0.0103333239094 | 1.03% | k\_\_Bacteria;p\_\_Verrucomicrobia;c\_\_Verrucomicrobiae;o\_\_Verrucomicrobiales |
| 0.00988097594074 | 0.99% | k\_\_Bacteria;p\_\_Firmicutes;c\_\_Bacilli;o\_\_Lactobacillales |
| 0.00802917644398 | 0.80% | k\_\_Bacteria;p\_\_Proteobacteria;c\_\_Deltaproteobacteria;o\_\_Desulfovibrionales |
| 0.0016114896384 | 0.16% | k\_\_Bacteria;p\_\_Firmicutes;c\_\_Erysipelotrichia;o\_\_Erysipelotrichales |
| 0.000692657827033 | 0.07% | k\_\_Bacteria;p\_\_unidentified\_Bacteria;c\_\_unidentified\_Bacteria;o\_\_unidentified\_Bacteria |
| 0.000537163212801 | 0.05% | k\_\_Bacteria;p\_\_Proteobacteria;c\_\_Gammaproteobacteria;o\_\_unidentified\_Gammaproteobacteria |
| 0.000339260976506 | 0.03% | k\_\_Bacteria;p\_\_Firmicutes;c\_\_Negativicutes;o\_\_Selenomonadales |
| 0.000325125102485 | 0.03% | k\_\_Bacteria;p\_\_Fusobacteria;c\_\_Fusobacteriia;o\_\_Fusobacteriales |
| 0.000296853354443 | 0.03% | k\_\_Bacteria;p\_\_Melainabacteria;Other;Other |
| 0.000282717480422 | 0.03% | k\_\_Bacteria;p\_\_Tenericutes;c\_\_Mollicutes;o\_\_ |
| 0.000197902236295 | 0.02% | k\_\_Bacteria;p\_\_Actinobacteria;c\_\_unidentified\_Actinobacteria;o\_\_Bifidobacteriales |
| 0.000141358740211 | 0.01% | k\_\_Bacteria;p\_\_Proteobacteria;c\_\_Gammaproteobacteria;o\_\_Pseudomonadales |
| 8.48152441265e-05 | 0.01% | k\_\_Bacteria;p\_\_Proteobacteria;c\_\_Gammaproteobacteria;o\_\_Pasteurellales |
| 7.06793701055e-05 | 0.01% | k\_\_Bacteria;p\_\_Oxyphotobacteria;c\_\_unidentified\_Oxyphotobacteria;o\_\_unidentified\_Oxyphotobacteria |
| 7.06793701055e-05 | 0.01% | k\_\_Bacteria;p\_\_Firmicutes;Other;Other |
| 5.65434960844e-05 | 0.01% | k\_\_Bacteria;p\_\_Proteobacteria;c\_\_Alphaproteobacteria;o\_\_Rhizobiales |
| 5.65434960844e-05 | 0.01% | k\_\_Bacteria;p\_\_Actinobacteria;c\_\_unidentified\_Actinobacteria;o\_\_Corynebacteriales |
| 2.82717480422e-05 | 0.00% | k\_\_Bacteria;p\_\_unidentified\_Bacteria;c\_\_unidentified\_Bacteria;o\_\_Campylobacterales |
| 2.82717480422e-05 | 0.00% | k\_\_Bacteria;p\_\_Proteobacteria;c\_\_Gammaproteobacteria;o\_\_Xanthomonadales |
| 2.82717480422e-05 | 0.00% | k\_\_Bacteria;p\_\_Proteobacteria;c\_\_Alphaproteobacteria;o\_\_Sphingomonadales |
| 1.41358740211e-05 | 0.00% | k\_\_Bacteria;p\_\_Firmicutes;c\_\_Bacilli;o\_\_Bacillales |
| 1.41358740211e-05 | 0.00% | k\_\_Bacteria;p\_\_Actinobacteria;c\_\_unidentified\_Actinobacteria;o\_\_Micrococcales |

|  |  |
| --- | --- |
|  | |
| Taxonomy Summary. Current Level: \_MD5 | |
| View Figure (.pdf)  View Legend (.pdf) |  |
|  |  |
| View Figure (.pdf)  View Legend (.pdf) |  |
|  |  |
|  |  |

|  |  |  |
| --- | --- | --- |
| Count | Pct | Taxonomy |
| 0.500367532725 | 50.04% | k\_\_Bacteria;p\_\_Bacteroidetes;c\_\_Bacteroidia;o\_\_Bacteroidales |
| 0.434678126149 | 43.47% | k\_\_Bacteria;p\_\_Firmicutes;c\_\_Clostridia;o\_\_Clostridiales |
| 0.0227446212999 | 2.27% | k\_\_Bacteria;p\_\_Firmicutes;c\_\_Bacilli;o\_\_Lactobacillales |
| 0.0193944191569 | 1.94% | k\_\_Bacteria;p\_\_Proteobacteria;c\_\_Gammaproteobacteria;o\_\_Enterobacteriales |
| 0.0136128466823 | 1.36% | k\_\_Bacteria;p\_\_Proteobacteria;c\_\_Deltaproteobacteria;o\_\_Desulfovibrionales |
| 0.0041559469622 | 0.42% | k\_\_Bacteria;p\_\_Tenericutes;c\_\_Mollicutes;o\_\_ |
| 0.00117327754375 | 0.12% | k\_\_Bacteria;p\_\_Firmicutes;c\_\_Erysipelotrichia;o\_\_Erysipelotrichales |
| 0.000735065449097 | 0.07% | k\_\_Bacteria;p\_\_Firmicutes;c\_\_Negativicutes;o\_\_Selenomonadales |
| 0.000593706708886 | 0.06% | k\_\_Bacteria;p\_\_Firmicutes;Other;Other |
| 0.000452347968675 | 0.05% | k\_\_Bacteria;p\_\_unidentified\_Bacteria;c\_\_unidentified\_Bacteria;o\_\_unidentified\_Bacteria |
| 0.000395804472591 | 0.04% | k\_\_Bacteria;p\_\_Verrucomicrobia;c\_\_Verrucomicrobiae;o\_\_Verrucomicrobiales |
| 0.000381668598569 | 0.04% | k\_\_Bacteria;p\_\_Proteobacteria;c\_\_Gammaproteobacteria;o\_\_unidentified\_Gammaproteobacteria |
| 0.000268581606401 | 0.03% | k\_\_Bacteria;p\_\_Fusobacteria;c\_\_Fusobacteriia;o\_\_Fusobacteriales |
| 0.000268581606401 | 0.03% | k\_\_Bacteria;Other;Other;Other |
| 0.000169630488253 | 0.02% | k\_\_Bacteria;p\_\_Actinobacteria;c\_\_unidentified\_Actinobacteria;o\_\_Bifidobacteriales |
| 0.00012722286619 | 0.01% | k\_\_Bacteria;p\_\_Proteobacteria;c\_\_Gammaproteobacteria;o\_\_Xanthomonadales |
| 8.48152441265e-05 | 0.01% | k\_\_Bacteria;p\_\_Melainabacteria;Other;Other |
| 7.06793701055e-05 | 0.01% | k\_\_Bacteria;p\_\_Proteobacteria;c\_\_Gammaproteobacteria;o\_\_Pseudomonadales |
| 7.06793701055e-05 | 0.01% | k\_\_Bacteria;p\_\_Proteobacteria;c\_\_Alphaproteobacteria;o\_\_Sphingomonadales |
| 5.65434960844e-05 | 0.01% | k\_\_Bacteria;p\_\_unidentified\_Bacteria;c\_\_unidentified\_Bacteria;o\_\_Campylobacterales |
| 4.24076220633e-05 | 0.00% | k\_\_Bacteria;p\_\_Proteobacteria;c\_\_Alphaproteobacteria;o\_\_Rhizobiales |
| 4.24076220633e-05 | 0.00% | k\_\_Bacteria;p\_\_Proteobacteria;Other;Other |
| 2.82717480422e-05 | 0.00% | k\_\_Bacteria;p\_\_Bacteroidetes;c\_\_Bacteroidia;o\_\_Flavobacteriales |
| 1.41358740211e-05 | 0.00% | k\_\_Bacteria;p\_\_Proteobacteria;c\_\_Gammaproteobacteria;o\_\_Pasteurellales |
| 1.41358740211e-05 | 0.00% | k\_\_Bacteria;p\_\_Proteobacteria;c\_\_Alphaproteobacteria;o\_\_Rhodospirillales |
| 1.41358740211e-05 | 0.00% | k\_\_Bacteria;p\_\_Oxyphotobacteria;c\_\_unidentified\_Oxyphotobacteria;o\_\_unidentified\_Oxyphotobacteria |
| 1.41358740211e-05 | 0.00% | k\_\_Bacteria;p\_\_Firmicutes;c\_\_Bacilli;o\_\_Bacillales |
| 1.41358740211e-05 | 0.00% | k\_\_Bacteria;p\_\_Actinobacteria;c\_\_unidentified\_Actinobacteria;o\_\_Corynebacteriales |
| 1.41358740211e-05 | 0.00% | k\_\_Bacteria;p\_\_Actinobacteria;c\_\_Coriobacteriia;o\_\_Coriobacteriales |

|  |  |
| --- | --- |
|  | |
| Taxonomy Summary. Current Level: \_MC1 | |
| View Figure (.pdf)  View Legend (.pdf) |  |
|  |  |
| View Figure (.pdf)  View Legend (.pdf) |  |
|  |  |
|  |  |

|  |  |  |
| --- | --- | --- |
| Count | Pct | Taxonomy |
| 0.563484210229 | 56.35% | k\_\_Bacteria;p\_\_Bacteroidetes;c\_\_Bacteroidia;o\_\_Bacteroidales |
| 0.266079556699 | 26.61% | k\_\_Bacteria;p\_\_Firmicutes;c\_\_Clostridia;o\_\_Clostridiales |
| 0.0867801306155 | 8.68% | k\_\_Bacteria;p\_\_Verrucomicrobia;c\_\_Verrucomicrobiae;o\_\_Verrucomicrobiales |
| 0.0290774928614 | 2.91% | k\_\_Bacteria;p\_\_Proteobacteria;c\_\_Gammaproteobacteria;o\_\_Enterobacteriales |
| 0.0251901275056 | 2.52% | k\_\_Bacteria;p\_\_Tenericutes;c\_\_Mollicutes;o\_\_ |
| 0.0103474597834 | 1.03% | k\_\_Bacteria;p\_\_Firmicutes;c\_\_Erysipelotrichia;o\_\_Erysipelotrichales |
| 0.00718102400271 | 0.72% | k\_\_Bacteria;p\_\_Firmicutes;c\_\_Bacilli;o\_\_Lactobacillales |
| 0.00453761556077 | 0.45% | k\_\_Bacteria;p\_\_Proteobacteria;c\_\_Deltaproteobacteria;o\_\_Desulfovibrionales |
| 0.00166803313449 | 0.17% | k\_\_Bacteria;p\_\_Melainabacteria;Other;Other |
| 0.0012722286619 | 0.13% | k\_\_Bacteria;Other;Other;Other |
| 0.00104605467756 | 0.10% | k\_\_Bacteria;p\_\_Oxyphotobacteria;c\_\_unidentified\_Oxyphotobacteria;o\_\_unidentified\_Oxyphotobacteria |
| 0.000593706708886 | 0.06% | k\_\_Bacteria;p\_\_unidentified\_Bacteria;c\_\_unidentified\_Bacteria;o\_\_unidentified\_Bacteria |
| 0.000480619716717 | 0.05% | k\_\_Bacteria;p\_\_Actinobacteria;c\_\_unidentified\_Actinobacteria;o\_\_Bifidobacteriales |
| 0.000409940346612 | 0.04% | k\_\_Bacteria;p\_\_Proteobacteria;c\_\_Gammaproteobacteria;o\_\_unidentified\_Gammaproteobacteria |
| 0.000409940346612 | 0.04% | k\_\_Bacteria;p\_\_Firmicutes;c\_\_Negativicutes;o\_\_Selenomonadales |
| 0.000395804472591 | 0.04% | k\_\_Bacteria;p\_\_Fusobacteria;c\_\_Fusobacteriia;o\_\_Fusobacteriales |
| 0.000310989228464 | 0.03% | k\_\_Bacteria;p\_\_Proteobacteria;c\_\_Gammaproteobacteria;o\_\_Pseudomonadales |
| 0.000183766362274 | 0.02% | k\_\_Bacteria;p\_\_Firmicutes;Other;Other |
| 8.48152441265e-05 | 0.01% | k\_\_Bacteria;p\_\_Proteobacteria;c\_\_Alphaproteobacteria;o\_\_Rhizobiales |
| 8.48152441265e-05 | 0.01% | k\_\_Bacteria;p\_\_Bacteroidetes;c\_\_Bacteroidia;o\_\_Flavobacteriales |
| 5.65434960844e-05 | 0.01% | k\_\_Bacteria;p\_\_unidentified\_Bacteria;c\_\_unidentified\_Bacteria;o\_\_Campylobacterales |
| 5.65434960844e-05 | 0.01% | k\_\_Bacteria;p\_\_Proteobacteria;c\_\_Alphaproteobacteria;o\_\_Sphingomonadales |
| 5.65434960844e-05 | 0.01% | k\_\_Bacteria;p\_\_Firmicutes;c\_\_Bacilli;o\_\_Bacillales |
| 4.24076220633e-05 | 0.00% | k\_\_Bacteria;p\_\_Proteobacteria;c\_\_Gammaproteobacteria;o\_\_Pasteurellales |
| 4.24076220633e-05 | 0.00% | k\_\_Bacteria;p\_\_Actinobacteria;c\_\_unidentified\_Actinobacteria;o\_\_Corynebacteriales |
| 2.82717480422e-05 | 0.00% | k\_\_Bacteria;p\_\_Verrucomicrobia;c\_\_Verrucomicrobiae;o\_\_Chthoniobacterales |
| 2.82717480422e-05 | 0.00% | k\_\_Bacteria;p\_\_Proteobacteria;c\_\_Gammaproteobacteria;o\_\_Alteromonadales |
| 2.82717480422e-05 | 0.00% | k\_\_Bacteria;p\_\_Proteobacteria;c\_\_Alphaproteobacteria;o\_\_Rhodospirillales |
| 2.82717480422e-05 | 0.00% | k\_\_Bacteria;p\_\_Armatimonadetes;c\_\_Fimbriimonadia;o\_\_Fimbriimonadales |
| 1.41358740211e-05 | 0.00% | k\_\_Bacteria;p\_\_Actinobacteria;c\_\_unidentified\_Actinobacteria;o\_\_Actinomycetales |

|  |  |
| --- | --- |
|  | |
| Taxonomy Summary. Current Level: \_MC2 | |
| View Figure (.pdf)  View Legend (.pdf) |  |
|  |  |
| View Figure (.pdf)  View Legend (.pdf) |  |
|  |  |
|  |  |

|  |  |  |
| --- | --- | --- |
| Count | Pct | Taxonomy |
| 0.644666534732 | 64.47% | k\_\_Bacteria;p\_\_Bacteroidetes;c\_\_Bacteroidia;o\_\_Bacteroidales |
| 0.219968335642 | 22.00% | k\_\_Bacteria;p\_\_Firmicutes;c\_\_Clostridia;o\_\_Clostridiales |
| 0.0829351728817 | 8.29% | k\_\_Bacteria;p\_\_Proteobacteria;c\_\_Gammaproteobacteria;o\_\_Enterobacteriales |
| 0.0137824771706 | 1.38% | k\_\_Bacteria;p\_\_Firmicutes;c\_\_Bacilli;o\_\_Lactobacillales |
| 0.0128636453592 | 1.29% | k\_\_Bacteria;p\_\_Proteobacteria;c\_\_Deltaproteobacteria;o\_\_Desulfovibrionales |
| 0.0124678408866 | 1.25% | k\_\_Bacteria;p\_\_Verrucomicrobia;c\_\_Verrucomicrobiae;o\_\_Verrucomicrobiales |
| 0.00568262135648 | 0.57% | k\_\_Bacteria;p\_\_Firmicutes;c\_\_Erysipelotrichia;o\_\_Erysipelotrichales |
| 0.00385909360776 | 0.39% | k\_\_Bacteria;p\_\_Tenericutes;c\_\_Mollicutes;o\_\_ |
| 0.00111673404767 | 0.11% | k\_\_Bacteria;p\_\_unidentified\_Bacteria;c\_\_unidentified\_Bacteria;o\_\_unidentified\_Bacteria |
| 0.000395804472591 | 0.04% | k\_\_Bacteria;p\_\_Actinobacteria;c\_\_unidentified\_Actinobacteria;o\_\_Bifidobacteriales |
| 0.000339260976506 | 0.03% | k\_\_Bacteria;p\_\_Proteobacteria;c\_\_Gammaproteobacteria;o\_\_unidentified\_Gammaproteobacteria |
| 0.000325125102485 | 0.03% | k\_\_Bacteria;p\_\_Firmicutes;c\_\_Negativicutes;o\_\_Selenomonadales |
| 0.000310989228464 | 0.03% | k\_\_Bacteria;p\_\_Melainabacteria;Other;Other |
| 0.000282717480422 | 0.03% | k\_\_Bacteria;p\_\_Fusobacteria;c\_\_Fusobacteriia;o\_\_Fusobacteriales |
| 0.00025444573238 | 0.03% | k\_\_Bacteria;Other;Other;Other |
| 0.000240309858359 | 0.02% | k\_\_Bacteria;p\_\_Firmicutes;Other;Other |
| 0.000141358740211 | 0.01% | k\_\_Bacteria;p\_\_Proteobacteria;c\_\_Gammaproteobacteria;o\_\_Pseudomonadales |
| 7.06793701055e-05 | 0.01% | k\_\_Bacteria;p\_\_Actinobacteria;c\_\_unidentified\_Actinobacteria;o\_\_Micrococcales |
| 5.65434960844e-05 | 0.01% | k\_\_Bacteria;p\_\_Bacteroidetes;c\_\_Bacteroidia;o\_\_Flavobacteriales |
| 5.65434960844e-05 | 0.01% | k\_\_Bacteria;p\_\_Actinobacteria;c\_\_unidentified\_Actinobacteria;o\_\_Corynebacteriales |
| 2.82717480422e-05 | 0.00% | k\_\_Bacteria;p\_\_unidentified\_Bacteria;c\_\_unidentified\_Bacteria;o\_\_Campylobacterales |
| 2.82717480422e-05 | 0.00% | k\_\_Bacteria;p\_\_Proteobacteria;c\_\_Alphaproteobacteria;o\_\_Sphingomonadales |
| 2.82717480422e-05 | 0.00% | k\_\_Bacteria;p\_\_Firmicutes;c\_\_Bacilli;o\_\_Bacillales |
| 1.41358740211e-05 | 0.00% | k\_\_Bacteria;p\_\_Proteobacteria;c\_\_Gammaproteobacteria;o\_\_Xanthomonadales |
| 1.41358740211e-05 | 0.00% | k\_\_Bacteria;p\_\_Proteobacteria;c\_\_Gammaproteobacteria;o\_\_Pasteurellales |
| 1.41358740211e-05 | 0.00% | k\_\_Bacteria;p\_\_Proteobacteria;c\_\_Alphaproteobacteria;o\_\_unidentified\_Alphaproteobacteria |
| 1.41358740211e-05 | 0.00% | k\_\_Bacteria;p\_\_Proteobacteria;c\_\_Alphaproteobacteria;o\_\_Rhizobiales |
| 1.41358740211e-05 | 0.00% | k\_\_Bacteria;p\_\_Oxyphotobacteria;c\_\_unidentified\_Oxyphotobacteria;o\_\_unidentified\_Oxyphotobacteria |
| 1.41358740211e-05 | 0.00% | k\_\_Bacteria;p\_\_Actinobacteria;c\_\_Rubrobacteria;o\_\_Rubrobacterales |
| 1.41358740211e-05 | 0.00% | k\_\_Archaea;p\_\_Euryarchaeota;c\_\_Methanobacteria;o\_\_Methanobacteriales |

|  |  |
| --- | --- |
|  | |
| Taxonomy Summary. Current Level: \_MC3 | |
| View Figure (.pdf)  View Legend (.pdf) |  |
|  |  |
| View Figure (.pdf)  View Legend (.pdf) |  |
|  |  |
|  |  |

|  |  |  |
| --- | --- | --- |
| Count | Pct | Taxonomy |
| 0.614444036075 | 61.44% | k\_\_Bacteria;p\_\_Bacteroidetes;c\_\_Bacteroidia;o\_\_Bacteroidales |
| 0.295241864805 | 29.52% | k\_\_Bacteria;p\_\_Firmicutes;c\_\_Clostridia;o\_\_Clostridiales |
| 0.0424500296853 | 4.25% | k\_\_Bacteria;p\_\_Proteobacteria;c\_\_Gammaproteobacteria;o\_\_Enterobacteriales |
| 0.018786576574 | 1.88% | k\_\_Bacteria;p\_\_Proteobacteria;c\_\_Deltaproteobacteria;o\_\_Desulfovibrionales |
| 0.012764694241 | 1.28% | k\_\_Bacteria;p\_\_Firmicutes;c\_\_Bacilli;o\_\_Lactobacillales |
| 0.00689830652229 | 0.69% | k\_\_Bacteria;p\_\_Firmicutes;c\_\_Erysipelotrichia;o\_\_Erysipelotrichales |
| 0.0031522999067 | 0.32% | k\_\_Bacteria;p\_\_Tenericutes;c\_\_Mollicutes;o\_\_ |
| 0.00114500579571 | 0.11% | k\_\_Bacteria;p\_\_Verrucomicrobia;c\_\_Verrucomicrobiae;o\_\_Verrucomicrobiales |
| 0.000706793701055 | 0.07% | k\_\_Bacteria;Other;Other;Other |
| 0.000593706708886 | 0.06% | k\_\_Bacteria;p\_\_unidentified\_Bacteria;c\_\_unidentified\_Bacteria;o\_\_unidentified\_Bacteria |
| 0.000508891464759 | 0.05% | k\_\_Bacteria;p\_\_Proteobacteria;c\_\_Gammaproteobacteria;o\_\_unidentified\_Gammaproteobacteria |
| 0.000508891464759 | 0.05% | k\_\_Bacteria;p\_\_Firmicutes;c\_\_Negativicutes;o\_\_Selenomonadales |
| 0.000452347968675 | 0.05% | k\_\_Bacteria;p\_\_Firmicutes;Other;Other |
| 0.000353396850527 | 0.04% | k\_\_Bacteria;p\_\_Actinobacteria;c\_\_unidentified\_Actinobacteria;o\_\_Bifidobacteriales |
| 0.000268581606401 | 0.03% | k\_\_Bacteria;p\_\_Proteobacteria;c\_\_Gammaproteobacteria;o\_\_Pseudomonadales |
| 0.000268581606401 | 0.03% | k\_\_Bacteria;p\_\_Fusobacteria;c\_\_Fusobacteriia;o\_\_Fusobacteriales |
| 0.000183766362274 | 0.02% | k\_\_Bacteria;p\_\_Proteobacteria;c\_\_Gammaproteobacteria;o\_\_Pasteurellales |
| 0.000155494614232 | 0.02% | k\_\_Bacteria;p\_\_Melainabacteria;Other;Other |
| 0.000141358740211 | 0.01% | k\_\_Bacteria;p\_\_unidentified\_Bacteria;c\_\_unidentified\_Bacteria;o\_\_Campylobacterales |
| 0.00012722286619 | 0.01% | k\_\_Bacteria;p\_\_Firmicutes;c\_\_Bacilli;o\_\_Bacillales |
| 0.00012722286619 | 0.01% | k\_\_Bacteria;p\_\_Actinobacteria;c\_\_Thermoleophilia;o\_\_Solirubrobacterales |
| 8.48152441265e-05 | 0.01% | k\_\_Bacteria;p\_\_Proteobacteria;c\_\_Gammaproteobacteria;o\_\_Xanthomonadales |
| 8.48152441265e-05 | 0.01% | k\_\_Bacteria;p\_\_Proteobacteria;c\_\_Alphaproteobacteria;o\_\_Sphingomonadales |
| 8.48152441265e-05 | 0.01% | k\_\_Bacteria;p\_\_Proteobacteria;c\_\_Alphaproteobacteria;o\_\_Rhizobiales |
| 7.06793701055e-05 | 0.01% | k\_\_Bacteria;p\_\_Bacteroidetes;c\_\_Bacteroidia;o\_\_Chitinophagales |
| 5.65434960844e-05 | 0.01% | k\_\_Bacteria;p\_\_Proteobacteria;c\_\_Alphaproteobacteria;o\_\_Rhodospirillales |
| 5.65434960844e-05 | 0.01% | k\_\_Bacteria;p\_\_Oxyphotobacteria;c\_\_unidentified\_Oxyphotobacteria;o\_\_unidentified\_Oxyphotobacteria |
| 5.65434960844e-05 | 0.01% | k\_\_Archaea;p\_\_Euryarchaeota;c\_\_Methanobacteria;o\_\_Methanobacteriales |
| 4.24076220633e-05 | 0.00% | k\_\_Bacteria;p\_\_Actinobacteria;c\_\_unidentified\_Actinobacteria;o\_\_Corynebacteriales |
| 4.24076220633e-05 | 0.00% | k\_\_Bacteria;p\_\_Actinobacteria;c\_\_Rubrobacteria;o\_\_Rubrobacterales |
| 2.82717480422e-05 | 0.00% | k\_\_Bacteria;p\_\_Tenericutes;c\_\_Mollicutes;o\_\_Anaeroplasmatales |
| 2.82717480422e-05 | 0.00% | k\_\_Bacteria;p\_\_Bacteroidetes;c\_\_Bacteroidia;o\_\_Sphingobacteriales |
| 2.82717480422e-05 | 0.00% | k\_\_Bacteria;p\_\_Acidobacteria;c\_\_unidentified\_Acidobacteria;o\_\_unidentified\_Acidobacteria |
| 1.41358740211e-05 | 0.00% | k\_\_Bacteria;p\_\_Deinococcus-Thermus;c\_\_Deinococci;o\_\_Deinococcales |
| 1.41358740211e-05 | 0.00% | k\_\_Bacteria;p\_\_Bacteroidetes;c\_\_Bacteroidia;o\_\_Flavobacteriales |
| 1.41358740211e-05 | 0.00% | k\_\_Bacteria;p\_\_Actinobacteria;c\_\_Thermoleophilia;o\_\_Gaiellales |
| 1.41358740211e-05 | 0.00% | k\_\_Bacteria;p\_\_Actinobacteria;c\_\_Coriobacteriia;o\_\_Coriobacteriales |

|  |  |
| --- | --- |
|  | |
| Taxonomy Summary. Current Level: \_MC4 | |
| View Figure (.pdf)  View Legend (.pdf) |  |
|  |  |
| View Figure (.pdf)  View Legend (.pdf) |  |
|  |  |
|  |  |

|  |  |  |
| --- | --- | --- |
| Count | Pct | Taxonomy |
| 0.577733171242 | 57.77% | k\_\_Bacteria;p\_\_Firmicutes;c\_\_Clostridia;o\_\_Clostridiales |
| 0.387238132934 | 38.72% | k\_\_Bacteria;p\_\_Bacteroidetes;c\_\_Bacteroidia;o\_\_Bacteroidales |
| 0.0109694382404 | 1.10% | k\_\_Bacteria;p\_\_Proteobacteria;c\_\_Gammaproteobacteria;o\_\_Enterobacteriales |
| 0.00838257329451 | 0.84% | k\_\_Bacteria;p\_\_Proteobacteria;c\_\_Deltaproteobacteria;o\_\_Desulfovibrionales |
| 0.00500409940347 | 0.50% | k\_\_Bacteria;p\_\_Firmicutes;c\_\_Bacilli;o\_\_Lactobacillales |
| 0.00248791382771 | 0.25% | k\_\_Bacteria;p\_\_Firmicutes;c\_\_Erysipelotrichia;o\_\_Erysipelotrichales |
| 0.00188007124481 | 0.19% | k\_\_Bacteria;Other;Other;Other |
| 0.00130050040994 | 0.13% | k\_\_Bacteria;p\_\_Verrucomicrobia;c\_\_Verrucomicrobiae;o\_\_Verrucomicrobiales |
| 0.000862288315287 | 0.09% | k\_\_Bacteria;p\_\_Firmicutes;Other;Other |
| 0.000819880693223 | 0.08% | k\_\_Bacteria;p\_\_Tenericutes;c\_\_Mollicutes;o\_\_ |
| 0.00077747307116 | 0.08% | k\_\_Bacteria;p\_\_Firmicutes;c\_\_Negativicutes;o\_\_Selenomonadales |
| 0.000579570834865 | 0.06% | k\_\_Bacteria;p\_\_Proteobacteria;c\_\_Gammaproteobacteria;o\_\_unidentified\_Gammaproteobacteria |
| 0.000367532724548 | 0.04% | k\_\_Bacteria;p\_\_unidentified\_Bacteria;c\_\_unidentified\_Bacteria;o\_\_Campylobacterales |
| 0.000325125102485 | 0.03% | k\_\_Bacteria;p\_\_Melainabacteria;Other;Other |
| 0.000310989228464 | 0.03% | k\_\_Bacteria;p\_\_Fusobacteria;c\_\_Fusobacteriia;o\_\_Fusobacteriales |
| 0.000212038110316 | 0.02% | k\_\_Bacteria;p\_\_Actinobacteria;c\_\_unidentified\_Actinobacteria;o\_\_Bifidobacteriales |
| 0.000197902236295 | 0.02% | k\_\_Bacteria;p\_\_unidentified\_Bacteria;c\_\_unidentified\_Bacteria;o\_\_unidentified\_Bacteria |
| 0.000113086992169 | 0.01% | k\_\_Bacteria;p\_\_Proteobacteria;c\_\_Gammaproteobacteria;o\_\_Pseudomonadales |
| 8.48152441265e-05 | 0.01% | k\_\_Bacteria;p\_\_Actinobacteria;c\_\_unidentified\_Actinobacteria;o\_\_Actinomycetales |
| 5.65434960844e-05 | 0.01% | k\_\_Bacteria;p\_\_Proteobacteria;c\_\_Alphaproteobacteria;o\_\_Rhodospirillales |
| 5.65434960844e-05 | 0.01% | k\_\_Bacteria;p\_\_Firmicutes;c\_\_Bacilli;o\_\_Bacillales |
| 4.24076220633e-05 | 0.00% | k\_\_Bacteria;p\_\_Proteobacteria;c\_\_Gammaproteobacteria;o\_\_Xanthomonadales |
| 4.24076220633e-05 | 0.00% | k\_\_Bacteria;p\_\_Proteobacteria;c\_\_Gammaproteobacteria;o\_\_Pasteurellales |
| 4.24076220633e-05 | 0.00% | k\_\_Bacteria;p\_\_Proteobacteria;c\_\_Alphaproteobacteria;o\_\_Sphingomonadales |
| 4.24076220633e-05 | 0.00% | k\_\_Bacteria;p\_\_Oxyphotobacteria;c\_\_unidentified\_Oxyphotobacteria;o\_\_unidentified\_Oxyphotobacteria |
| 4.24076220633e-05 | 0.00% | k\_\_Bacteria;p\_\_Actinobacteria;c\_\_unidentified\_Actinobacteria;o\_\_Corynebacteriales |
| 2.82717480422e-05 | 0.00% | k\_\_Bacteria;p\_\_Proteobacteria;c\_\_Alphaproteobacteria;o\_\_Rhizobiales |

|  |  |
| --- | --- |
|  | |
| Taxonomy Summary. Current Level: \_MC5 | |
| View Figure (.pdf)  View Legend (.pdf) |  |
|  |  |
| View Figure (.pdf)  View Legend (.pdf) |  |
|  |  |
|  |  |

|  |  |  |
| --- | --- | --- |
| Count | Pct | Taxonomy |
| 0.51364111843 | 51.36% | k\_\_Bacteria;p\_\_Bacteroidetes;c\_\_Bacteroidia;o\_\_Bacteroidales |
| 0.393316558763 | 39.33% | k\_\_Bacteria;p\_\_Firmicutes;c\_\_Clostridia;o\_\_Clostridiales |
| 0.0478923411835 | 4.79% | k\_\_Bacteria;p\_\_Proteobacteria;c\_\_Gammaproteobacteria;o\_\_Enterobacteriales |
| 0.0141782816432 | 1.42% | k\_\_Bacteria;p\_\_Verrucomicrobia;c\_\_Verrucomicrobiae;o\_\_Verrucomicrobiales |
| 0.0101919651692 | 1.02% | k\_\_Bacteria;p\_\_Proteobacteria;c\_\_Deltaproteobacteria;o\_\_Desulfovibrionales |
| 0.00773232308954 | 0.77% | k\_\_Bacteria;p\_\_Firmicutes;c\_\_Bacilli;o\_\_Lactobacillales |
| 0.00460829493088 | 0.46% | k\_\_Bacteria;p\_\_Tenericutes;c\_\_Mollicutes;o\_\_ |
| 0.00221933222131 | 0.22% | k\_\_Bacteria;p\_\_Firmicutes;c\_\_Erysipelotrichia;o\_\_Erysipelotrichales |
| 0.00144185915015 | 0.14% | k\_\_Bacteria;p\_\_Melainabacteria;Other;Other |
| 0.0010743264256 | 0.11% | k\_\_Bacteria;Other;Other;Other |
| 0.000819880693223 | 0.08% | k\_\_Bacteria;p\_\_unidentified\_Bacteria;c\_\_unidentified\_Bacteria;o\_\_unidentified\_Bacteria |
| 0.000607842582907 | 0.06% | k\_\_Bacteria;p\_\_Firmicutes;c\_\_Negativicutes;o\_\_Selenomonadales |
| 0.000409940346612 | 0.04% | k\_\_Bacteria;p\_\_Proteobacteria;c\_\_Gammaproteobacteria;o\_\_unidentified\_Gammaproteobacteria |
| 0.000409940346612 | 0.04% | k\_\_Bacteria;p\_\_Actinobacteria;c\_\_unidentified\_Actinobacteria;o\_\_Corynebacteriales |
| 0.000339260976506 | 0.03% | k\_\_Bacteria;p\_\_Fusobacteria;c\_\_Fusobacteriia;o\_\_Fusobacteriales |
| 0.000310989228464 | 0.03% | k\_\_Bacteria;p\_\_Firmicutes;Other;Other |
| 0.00025444573238 | 0.03% | k\_\_Bacteria;p\_\_Actinobacteria;c\_\_unidentified\_Actinobacteria;o\_\_Bifidobacteriales |
| 0.000240309858359 | 0.02% | k\_\_Bacteria;p\_\_Proteobacteria;c\_\_Gammaproteobacteria;o\_\_Pseudomonadales |
| 0.00012722286619 | 0.01% | k\_\_Bacteria;p\_\_Proteobacteria;c\_\_Gammaproteobacteria;o\_\_Pasteurellales |
| 4.24076220633e-05 | 0.00% | k\_\_Bacteria;p\_\_unidentified\_Bacteria;c\_\_unidentified\_Bacteria;o\_\_Campylobacterales |
| 2.82717480422e-05 | 0.00% | k\_\_Bacteria;p\_\_Proteobacteria;c\_\_Alphaproteobacteria;o\_\_Caulobacterales |
| 2.82717480422e-05 | 0.00% | k\_\_Bacteria;p\_\_Acidobacteria;c\_\_unidentified\_Acidobacteria;o\_\_unidentified\_Acidobacteria |
| 2.82717480422e-05 | 0.00% | k\_\_Bacteria;p\_\_Acidobacteria;c\_\_Holophagae;o\_\_ |
| 1.41358740211e-05 | 0.00% | k\_\_Bacteria;p\_\_Proteobacteria;c\_\_Gammaproteobacteria;o\_\_Xanthomonadales |
| 1.41358740211e-05 | 0.00% | k\_\_Bacteria;p\_\_Proteobacteria;c\_\_Alphaproteobacteria;o\_\_Rhodospirillales |
| 1.41358740211e-05 | 0.00% | k\_\_Bacteria;p\_\_Proteobacteria;c\_\_Alphaproteobacteria;o\_\_Rhizobiales |
| 1.41358740211e-05 | 0.00% | k\_\_Bacteria;p\_\_Deinococcus-Thermus;c\_\_Deinococci;o\_\_Deinococcales |

|  |  |
| --- | --- |
|  | |
| Taxonomy Summary. Current Level: | |
| View Figure (.pdf)  View Legend (.pdf) |  |
|  |  |
| View Figure (.pdf)  View Legend (.pdf) |  |
|  |  |
|  |  |

|  |  |  |
| --- | --- | --- |
| Count | Pct | Taxonomy |
| 3.05165248367 | 30.52% | k\_\_Bacteria;p\_\_Bacteroidetes;c\_\_Bacteroidia;o\_\_Bacteroidales;f\_\_Muribaculaceae |
| 2.3339882955 | 23.34% | k\_\_Bacteria;p\_\_Firmicutes;c\_\_Clostridia;o\_\_Clostridiales;f\_\_Lachnospiraceae |
| 1.77482966272 | 17.75% | k\_\_Bacteria;p\_\_Bacteroidetes;c\_\_Bacteroidia;o\_\_Bacteroidales;f\_\_Bacteroidaceae |
| 1.26831302479 | 12.68% | k\_\_Bacteria;p\_\_Firmicutes;c\_\_Clostridia;o\_\_Clostridiales;f\_\_Ruminococcaceae |
| 0.569901897034 | 5.70% | k\_\_Bacteria;p\_\_Proteobacteria;c\_\_Gammaproteobacteria;o\_\_Enterobacteriales;f\_\_Enterobacteriaceae |
| 0.1945803059 | 1.95% | k\_\_Bacteria;p\_\_Bacteroidetes;c\_\_Bacteroidia;o\_\_Bacteroidales;f\_\_Rikenellaceae |
| 0.192728506404 | 1.93% | k\_\_Bacteria;p\_\_Bacteroidetes;c\_\_Bacteroidia;o\_\_Bacteroidales;f\_\_Prevotellaceae |
| 0.135718526477 | 1.36% | k\_\_Bacteria;p\_\_Verrucomicrobia;c\_\_Verrucomicrobiae;o\_\_Verrucomicrobiales;f\_\_Akkermansiaceae |
| 0.1115461819 | 1.12% | k\_\_Bacteria;p\_\_Proteobacteria;c\_\_Deltaproteobacteria;o\_\_Desulfovibrionales;f\_\_Desulfovibrionaceae |
| 0.0599502417235 | 0.60% | k\_\_Bacteria;p\_\_Bacteroidetes;c\_\_Bacteroidia;o\_\_Bacteroidales;f\_\_Tannerellaceae |
| 0.0537445930282 | 0.54% | k\_\_Bacteria;p\_\_Firmicutes;c\_\_Bacilli;o\_\_Lactobacillales;f\_\_Lactobacillaceae |
| 0.0450510305052 | 0.45% | k\_\_Bacteria;p\_\_Tenericutes;c\_\_Mollicutes;o\_\_;f\_\_ |
| 0.0445280031664 | 0.45% | k\_\_Bacteria;p\_\_Firmicutes;c\_\_Bacilli;o\_\_Lactobacillales;f\_\_Enterococcaceae |
| 0.0404427355743 | 0.40% | k\_\_Bacteria;p\_\_Firmicutes;c\_\_Erysipelotrichia;o\_\_Erysipelotrichales;f\_\_Erysipelotrichaceae |
| 0.0377851912584 | 0.38% | k\_\_Bacteria;p\_\_Firmicutes;c\_\_Clostridia;o\_\_Clostridiales;Other |
| 0.0145458143677 | 0.15% | k\_\_Bacteria;p\_\_Firmicutes;c\_\_Clostridia;o\_\_Clostridiales;f\_\_unidentified\_Clostridiales |
| 0.00856633965678 | 0.09% | k\_\_Bacteria;p\_\_Firmicutes;c\_\_Clostridia;o\_\_Clostridiales;f\_\_Peptococcaceae |
| 0.00845325266461 | 0.08% | k\_\_Bacteria;Other;Other;Other;Other |
| 0.00793022532583 | 0.08% | k\_\_Bacteria;p\_\_Bacteroidetes;c\_\_Bacteroidia;o\_\_Bacteroidales;f\_\_Marinifilaceae |
| 0.00490514828532 | 0.05% | k\_\_Bacteria;p\_\_unidentified\_Bacteria;c\_\_unidentified\_Bacteria;o\_\_unidentified\_Bacteria;f\_\_unidentified\_Bacteria |
| 0.00486274066325 | 0.05% | k\_\_Bacteria;p\_\_Proteobacteria;c\_\_Gammaproteobacteria;o\_\_unidentified\_Gammaproteobacteria;f\_\_Burkholderiaceae |
| 0.00463656667892 | 0.05% | k\_\_Bacteria;p\_\_Melainabacteria;Other;Other;Other |
| 0.00400045234797 | 0.04% | k\_\_Bacteria;p\_\_Oxyphotobacteria;c\_\_unidentified\_Oxyphotobacteria;o\_\_unidentified\_Oxyphotobacteria;f\_\_unidentified\_Oxyphotobacteria |
| 0.00385909360776 | 0.04% | k\_\_Bacteria;p\_\_Firmicutes;c\_\_Negativicutes;o\_\_Selenomonadales;f\_\_Veillonellaceae |
| 0.00347742500919 | 0.03% | k\_\_Bacteria;p\_\_Firmicutes;Other;Other;Other |
| 0.00295439767041 | 0.03% | k\_\_Bacteria;p\_\_Fusobacteria;c\_\_Fusobacteriia;o\_\_Fusobacteriales;f\_\_Fusobacteriaceae |
| 0.00269995193803 | 0.03% | k\_\_Bacteria;p\_\_Actinobacteria;c\_\_unidentified\_Actinobacteria;o\_\_Bifidobacteriales;f\_\_Bifidobacteriaceae |
| 0.00118741341777 | 0.01% | k\_\_Bacteria;p\_\_Firmicutes;c\_\_Bacilli;o\_\_Lactobacillales;f\_\_Streptococcaceae |
| 0.00114500579571 | 0.01% | k\_\_Bacteria;p\_\_Proteobacteria;c\_\_Gammaproteobacteria;o\_\_Pseudomonadales;f\_\_Moraxellaceae |
| 0.00113086992169 | 0.01% | k\_\_Bacteria;p\_\_unidentified\_Bacteria;c\_\_unidentified\_Bacteria;o\_\_Campylobacterales;f\_\_unidentified\_Campylobacterales |
| 0.00110259817365 | 0.01% | k\_\_Bacteria;p\_\_Firmicutes;c\_\_Clostridia;o\_\_Clostridiales;f\_\_Peptostreptococcaceae |
| 0.00101778292952 | 0.01% | k\_\_Bacteria;p\_\_Firmicutes;c\_\_Negativicutes;o\_\_Selenomonadales;f\_\_Acidaminococcaceae |
| 0.000918831811371 | 0.01% | k\_\_Bacteria;p\_\_Proteobacteria;c\_\_Gammaproteobacteria;o\_\_Pseudomonadales;f\_\_Pseudomonadaceae |
| 0.00089056006333 | 0.01% | k\_\_Bacteria;p\_\_Bacteroidetes;c\_\_Bacteroidia;o\_\_Bacteroidales;f\_\_Porphyromonadaceae |
| 0.000819880693223 | 0.01% | k\_\_Bacteria;p\_\_Bacteroidetes;c\_\_Bacteroidia;o\_\_Bacteroidales;f\_\_Barnesiellaceae |
| 0.000749201323118 | 0.01% | k\_\_Bacteria;p\_\_Proteobacteria;c\_\_Gammaproteobacteria;o\_\_Pasteurellales;f\_\_Pasteurellaceae |
| 0.000720929575076 | 0.01% | k\_\_Bacteria;p\_\_Actinobacteria;c\_\_unidentified\_Actinobacteria;o\_\_Corynebacteriales;f\_\_Nocardiaceae |
| 0.000409940346612 | 0.00% | k\_\_Bacteria;p\_\_Proteobacteria;c\_\_Gammaproteobacteria;o\_\_Xanthomonadales;f\_\_Xanthomonadaceae |
| 0.000395804472591 | 0.00% | k\_\_Bacteria;p\_\_Proteobacteria;c\_\_Alphaproteobacteria;o\_\_Sphingomonadales;f\_\_Sphingomonadaceae |
| 0.000310989228464 | 0.00% | k\_\_Bacteria;p\_\_Proteobacteria;c\_\_Alphaproteobacteria;o\_\_Rhizobiales;f\_\_Rhizobiaceae |
| 0.000268581606401 | 0.00% | k\_\_Bacteria;p\_\_Firmicutes;c\_\_Clostridia;o\_\_Clostridiales;f\_\_Eubacteriaceae |
| 0.00025444573238 | 0.00% | k\_\_Bacteria;p\_\_Proteobacteria;c\_\_Alphaproteobacteria;o\_\_Rhodospirillales;f\_\_unidentified\_Rhodospirillales |
| 0.000212038110317 | 0.00% | k\_\_Bacteria;p\_\_Firmicutes;c\_\_Bacilli;o\_\_Bacillales;f\_\_Staphylococcaceae |
| 0.000212038110316 | 0.00% | k\_\_Bacteria;p\_\_Actinobacteria;c\_\_Rubrobacteria;o\_\_Rubrobacterales;f\_\_unidentified\_Rubrobacterales |
| 0.000197902236296 | 0.00% | k\_\_Bacteria;p\_\_Actinobacteria;c\_\_Thermoleophilia;o\_\_Solirubrobacterales;f\_\_ |
| 0.000197902236295 | 0.00% | k\_\_Bacteria;p\_\_Firmicutes;c\_\_Bacilli;o\_\_Bacillales;f\_\_unidentified\_Bacillales |
| 0.000197902236295 | 0.00% | k\_\_Bacteria;p\_\_Proteobacteria;c\_\_Alphaproteobacteria;o\_\_unidentified\_Alphaproteobacteria;f\_\_unidentified\_Alphaproteobacteria |
| 0.000169630488253 | 0.00% | k\_\_Bacteria;p\_\_Proteobacteria;c\_\_Gammaproteobacteria;o\_\_unidentified\_Gammaproteobacteria;f\_\_Neisseriaceae |
| 0.000141358740211 | 0.00% | k\_\_Bacteria;p\_\_Bacteroidetes;c\_\_Bacteroidia;o\_\_Flavobacteriales;f\_\_Flavobacteriaceae |
| 0.00012722286619 | 0.00% | k\_\_Bacteria;p\_\_Actinobacteria;c\_\_unidentified\_Actinobacteria;o\_\_Actinomycetales;f\_\_Actinomycetaceae |
| 0.000113086992169 | 0.00% | k\_\_Bacteria;p\_\_Firmicutes;c\_\_Clostridia;o\_\_Clostridiales;f\_\_Christensenellaceae |
| 9.89511181477e-05 | 0.00% | k\_\_Bacteria;p\_\_Acidobacteria;c\_\_unidentified\_Acidobacteria;o\_\_unidentified\_Acidobacteria;f\_\_unidentified\_Acidobacteria |
| 8.48152441266e-05 | 0.00% | k\_\_Bacteria;p\_\_Proteobacteria;c\_\_Alphaproteobacteria;o\_\_Rhizobiales;Other |
| 8.48152441266e-05 | 0.00% | k\_\_Bacteria;p\_\_Actinobacteria;c\_\_unidentified\_Actinobacteria;o\_\_Micrococcales;f\_\_Microbacteriaceae |
| 8.48152441266e-05 | 0.00% | k\_\_Bacteria;p\_\_Acidobacteria;c\_\_Holophagae;o\_\_;f\_\_ |
| 7.06793701055e-05 | 0.00% | k\_\_Bacteria;p\_\_Firmicutes;c\_\_Bacilli;o\_\_Lactobacillales;f\_\_Leuconostocaceae |
| 7.06793701055e-05 | 0.00% | k\_\_Bacteria;p\_\_Bacteroidetes;c\_\_Bacteroidia;o\_\_Flavobacteriales;f\_\_unidentified\_Flavobacteriales |
| 7.06793701055e-05 | 0.00% | k\_\_Bacteria;p\_\_Bacteroidetes;c\_\_Bacteroidia;o\_\_Chitinophagales;f\_\_Chitinophagaceae |
| 7.06793701055e-05 | 0.00% | k\_\_Bacteria;p\_\_Actinobacteria;c\_\_Coriobacteriia;o\_\_Coriobacteriales;f\_\_Atopobiaceae |
| 7.06793701055e-05 | 0.00% | k\_\_Bacteria;p\_\_Acidobacteria;c\_\_unidentified\_Acidobacteria;o\_\_unidentified\_Acidobacteria;f\_\_Pyrinomonadaceae |
| 7.06793701055e-05 | 0.00% | k\_\_Archaea;p\_\_Euryarchaeota;c\_\_Methanobacteria;o\_\_Methanobacteriales;f\_\_Methanobacteriaceae |
| 5.65434960844e-05 | 0.00% | k\_\_Bacteria;p\_\_Proteobacteria;c\_\_Alphaproteobacteria;o\_\_Rhizobiales;f\_\_Xanthobacteraceae |
| 5.65434960844e-05 | 0.00% | k\_\_Bacteria;p\_\_Actinobacteria;c\_\_unidentified\_Actinobacteria;o\_\_Propionibacteriales;f\_\_Nocardioidaceae |
| 5.65434960844e-05 | 0.00% | k\_\_Bacteria;p\_\_Actinobacteria;c\_\_unidentified\_Actinobacteria;o\_\_Corynebacteriales;f\_\_Corynebacteriaceae |
| 5.65434960844e-05 | 0.00% | k\_\_Bacteria;p\_\_Actinobacteria;c\_\_Acidimicrobiia;Other;Other |
| 4.24076220633e-05 | 0.00% | k\_\_Bacteria;p\_\_Proteobacteria;Other;Other;Other |
| 4.24076220633e-05 | 0.00% | k\_\_Bacteria;p\_\_Bacteroidetes;c\_\_Bacteroidia;o\_\_Cytophagales;Other |
| 4.24076220633e-05 | 0.00% | k\_\_Bacteria;p\_\_Tenericutes;c\_\_Mollicutes;o\_\_Anaeroplasmatales;f\_\_Anaeroplasmataceae |
| 2.82717480422e-05 | 0.00% | k\_\_Bacteria;p\_\_unidentified\_Bacteria;c\_\_unidentified\_Bacteria;o\_\_Campylobacterales;f\_\_Campylobacteraceae |
| 2.82717480422e-05 | 0.00% | k\_\_Bacteria;p\_\_Verrucomicrobia;c\_\_Verrucomicrobiae;o\_\_Chthoniobacterales;f\_\_Chthoniobacteraceae |
| 2.82717480422e-05 | 0.00% | k\_\_Bacteria;p\_\_Proteobacteria;c\_\_Gammaproteobacteria;o\_\_Xanthomonadales;f\_\_Rhodanobacteraceae |
| 2.82717480422e-05 | 0.00% | k\_\_Bacteria;p\_\_Proteobacteria;c\_\_Gammaproteobacteria;o\_\_Alteromonadales;f\_\_Shewanellaceae |
| 2.82717480422e-05 | 0.00% | k\_\_Bacteria;p\_\_Proteobacteria;c\_\_Alphaproteobacteria;o\_\_Rhizobiales;f\_\_Beijerinckiaceae |
| 2.82717480422e-05 | 0.00% | k\_\_Bacteria;p\_\_Proteobacteria;c\_\_Alphaproteobacteria;o\_\_Caulobacterales;f\_\_Caulobacteraceae |
| 2.82717480422e-05 | 0.00% | k\_\_Bacteria;p\_\_Deinococcus-Thermus;c\_\_Deinococci;o\_\_Deinococcales;f\_\_Deinococcaceae |
| 2.82717480422e-05 | 0.00% | k\_\_Bacteria;p\_\_Bacteroidetes;c\_\_Bacteroidia;o\_\_Sphingobacteriales;f\_\_ |
| 2.82717480422e-05 | 0.00% | k\_\_Bacteria;p\_\_Armatimonadetes;c\_\_Fimbriimonadia;o\_\_Fimbriimonadales;f\_\_Fimbriimonadaceae |
| 2.82717480422e-05 | 0.00% | k\_\_Bacteria;p\_\_Actinobacteria;c\_\_Thermoleophilia;o\_\_Solirubrobacterales;Other |
| 2.82717480422e-05 | 0.00% | k\_\_Bacteria;p\_\_Actinobacteria;c\_\_Acidimicrobiia;o\_\_;f\_\_ |
| 1.41358740211e-05 | 0.00% | k\_\_Bacteria;p\_\_Actinobacteria;c\_\_Thermoleophilia;o\_\_Gaiellales;f\_\_ |

|  |  |
| --- | --- |
|  | |
| Taxonomy Summary. Current Level: \_MD1 | |
| View Figure (.pdf)  View Legend (.pdf) |  |
|  |  |
| View Figure (.pdf)  View Legend (.pdf) |  |
|  |  |
|  |  |

|  |  |  |
| --- | --- | --- |
| Count | Pct | Taxonomy |
| 0.375109553024 | 37.51% | k\_\_Bacteria;p\_\_Firmicutes;c\_\_Clostridia;o\_\_Clostridiales;f\_\_Lachnospiraceae |
| 0.280314381838 | 28.03% | k\_\_Bacteria;p\_\_Bacteroidetes;c\_\_Bacteroidia;o\_\_Bacteroidales;f\_\_Muribaculaceae |
| 0.11963190184 | 11.96% | k\_\_Bacteria;p\_\_Bacteroidetes;c\_\_Bacteroidia;o\_\_Bacteroidales;f\_\_Bacteroidaceae |
| 0.0882361256396 | 8.82% | k\_\_Bacteria;p\_\_Firmicutes;c\_\_Clostridia;o\_\_Clostridiales;f\_\_Ruminococcaceae |
| 0.0776200842498 | 7.76% | k\_\_Bacteria;p\_\_Proteobacteria;c\_\_Gammaproteobacteria;o\_\_Enterobacteriales;f\_\_Enterobacteriaceae |
| 0.0129767323514 | 1.30% | k\_\_Bacteria;p\_\_Bacteroidetes;c\_\_Bacteroidia;o\_\_Bacteroidales;f\_\_Rikenellaceae |
| 0.0123547538944 | 1.24% | k\_\_Bacteria;p\_\_Bacteroidetes;c\_\_Bacteroidia;o\_\_Bacteroidales;f\_\_Prevotellaceae |
| 0.00993751943683 | 0.99% | k\_\_Bacteria;p\_\_Proteobacteria;c\_\_Deltaproteobacteria;o\_\_Desulfovibrionales;f\_\_Desulfovibrionaceae |
| 0.0043538491985 | 0.44% | k\_\_Bacteria;p\_\_Bacteroidetes;c\_\_Bacteroidia;o\_\_Bacteroidales;f\_\_Tannerellaceae |
| 0.003350202143 | 0.34% | k\_\_Bacteria;p\_\_Firmicutes;c\_\_Bacilli;o\_\_Lactobacillales;f\_\_Lactobacillaceae |
| 0.00294026179639 | 0.29% | k\_\_Bacteria;p\_\_Firmicutes;c\_\_Clostridia;o\_\_Clostridiales;Other |
| 0.00244550620565 | 0.24% | k\_\_Bacteria;p\_\_Firmicutes;c\_\_Erysipelotrichia;o\_\_Erysipelotrichales;f\_\_Erysipelotrichaceae |
| 0.00214865285121 | 0.21% | k\_\_Bacteria;p\_\_Firmicutes;c\_\_Bacilli;o\_\_Lactobacillales;f\_\_Enterococcaceae |
| 0.00166803313449 | 0.17% | k\_\_Bacteria;p\_\_Verrucomicrobia;c\_\_Verrucomicrobiae;o\_\_Verrucomicrobiales;f\_\_Akkermansiaceae |
| 0.00114500579571 | 0.11% | k\_\_Bacteria;p\_\_Firmicutes;c\_\_Clostridia;o\_\_Clostridiales;f\_\_unidentified\_Clostridiales |
| 0.00106019055158 | 0.11% | k\_\_Bacteria;p\_\_Firmicutes;c\_\_Clostridia;o\_\_Clostridiales;f\_\_Peptococcaceae |
| 0.000819880693223 | 0.08% | k\_\_Bacteria;p\_\_Tenericutes;c\_\_Mollicutes;o\_\_;f\_\_ |
| 0.000636114330949 | 0.06% | k\_\_Bacteria;p\_\_Proteobacteria;c\_\_Gammaproteobacteria;o\_\_unidentified\_Gammaproteobacteria;f\_\_Burkholderiaceae |
| 0.000353396850527 | 0.04% | k\_\_Bacteria;Other;Other;Other;Other |
| 0.000339260976506 | 0.03% | k\_\_Bacteria;p\_\_Bacteroidetes;c\_\_Bacteroidia;o\_\_Bacteroidales;f\_\_Marinifilaceae |
| 0.000310989228464 | 0.03% | k\_\_Bacteria;p\_\_Firmicutes;c\_\_Negativicutes;o\_\_Selenomonadales;f\_\_Veillonellaceae |
| 0.000296853354443 | 0.03% | k\_\_Bacteria;p\_\_Fusobacteria;c\_\_Fusobacteriia;o\_\_Fusobacteriales;f\_\_Fusobacteriaceae |
| 0.000240309858359 | 0.02% | k\_\_Bacteria;p\_\_Actinobacteria;c\_\_unidentified\_Actinobacteria;o\_\_Bifidobacteriales;f\_\_Bifidobacteriaceae |
| 0.000212038110316 | 0.02% | k\_\_Bacteria;p\_\_Firmicutes;c\_\_Clostridia;o\_\_Clostridiales;f\_\_Peptostreptococcaceae |
| 0.000212038110316 | 0.02% | k\_\_Bacteria;p\_\_Firmicutes;Other;Other;Other |
| 0.000197902236295 | 0.02% | k\_\_Bacteria;p\_\_Proteobacteria;c\_\_Gammaproteobacteria;o\_\_Pseudomonadales;f\_\_Pseudomonadaceae |
| 0.000113086992169 | 0.01% | k\_\_Bacteria;p\_\_unidentified\_Bacteria;c\_\_unidentified\_Bacteria;o\_\_Campylobacterales;f\_\_unidentified\_Campylobacterales |
| 0.000113086992169 | 0.01% | k\_\_Bacteria;p\_\_Bacteroidetes;c\_\_Bacteroidia;o\_\_Bacteroidales;f\_\_Porphyromonadaceae |
| 9.89511181476e-05 | 0.01% | k\_\_Bacteria;p\_\_Proteobacteria;c\_\_Gammaproteobacteria;o\_\_Pseudomonadales;f\_\_Moraxellaceae |
| 9.89511181476e-05 | 0.01% | k\_\_Bacteria;p\_\_Melainabacteria;Other;Other;Other |
| 9.89511181476e-05 | 0.01% | k\_\_Bacteria;p\_\_Firmicutes;c\_\_Negativicutes;o\_\_Selenomonadales;f\_\_Acidaminococcaceae |
| 8.48152441265e-05 | 0.01% | k\_\_Bacteria;p\_\_Bacteroidetes;c\_\_Bacteroidia;o\_\_Bacteroidales;f\_\_Barnesiellaceae |
| 7.06793701055e-05 | 0.01% | k\_\_Bacteria;p\_\_Proteobacteria;c\_\_Gammaproteobacteria;o\_\_Xanthomonadales;f\_\_Xanthomonadaceae |
| 5.65434960844e-05 | 0.01% | k\_\_Bacteria;p\_\_unidentified\_Bacteria;c\_\_unidentified\_Bacteria;o\_\_unidentified\_Bacteria;f\_\_unidentified\_Bacteria |
| 4.24076220633e-05 | 0.00% | k\_\_Bacteria;p\_\_Proteobacteria;c\_\_Gammaproteobacteria;o\_\_Pasteurellales;f\_\_Pasteurellaceae |
| 4.24076220633e-05 | 0.00% | k\_\_Bacteria;p\_\_Proteobacteria;c\_\_Alphaproteobacteria;o\_\_Rhizobiales;f\_\_Rhizobiaceae |
| 4.24076220633e-05 | 0.00% | k\_\_Bacteria;p\_\_Firmicutes;c\_\_Bacilli;o\_\_Lactobacillales;f\_\_Streptococcaceae |
| 4.24076220633e-05 | 0.00% | k\_\_Bacteria;p\_\_Firmicutes;c\_\_Bacilli;o\_\_Bacillales;f\_\_unidentified\_Bacillales |
| 2.82717480422e-05 | 0.00% | k\_\_Bacteria;p\_\_Proteobacteria;c\_\_Gammaproteobacteria;o\_\_unidentified\_Gammaproteobacteria;f\_\_Neisseriaceae |
| 2.82717480422e-05 | 0.00% | k\_\_Bacteria;p\_\_Proteobacteria;c\_\_Alphaproteobacteria;o\_\_Sphingomonadales;f\_\_Sphingomonadaceae |
| 2.82717480422e-05 | 0.00% | k\_\_Bacteria;p\_\_Oxyphotobacteria;c\_\_unidentified\_Oxyphotobacteria;o\_\_unidentified\_Oxyphotobacteria;f\_\_unidentified\_Oxyphotobacteria |
| 2.82717480422e-05 | 0.00% | k\_\_Bacteria;p\_\_Firmicutes;c\_\_Bacilli;o\_\_Bacillales;f\_\_Staphylococcaceae |
| 1.41358740211e-05 | 0.00% | k\_\_Bacteria;p\_\_Proteobacteria;c\_\_Gammaproteobacteria;o\_\_Xanthomonadales;f\_\_Rhodanobacteraceae |
| 1.41358740211e-05 | 0.00% | k\_\_Bacteria;p\_\_Firmicutes;c\_\_Clostridia;o\_\_Clostridiales;f\_\_Christensenellaceae |
| 1.41358740211e-05 | 0.00% | k\_\_Bacteria;p\_\_Actinobacteria;c\_\_unidentified\_Actinobacteria;o\_\_Corynebacteriales;f\_\_Nocardiaceae |
| 1.41358740211e-05 | 0.00% | k\_\_Bacteria;p\_\_Actinobacteria;c\_\_unidentified\_Actinobacteria;o\_\_Actinomycetales;f\_\_Actinomycetaceae |
| 1.41358740211e-05 | 0.00% | k\_\_Bacteria;p\_\_Actinobacteria;c\_\_Thermoleophilia;o\_\_Solirubrobacterales;f\_\_ |

|  |  |
| --- | --- |
|  | |
| Taxonomy Summary. Current Level: \_MD2 | |
| View Figure (.pdf)  View Legend (.pdf) |  |
|  |  |
| View Figure (.pdf)  View Legend (.pdf) |  |
|  |  |
|  |  |

|  |  |  |
| --- | --- | --- |
| Count | Pct | Taxonomy |
| 0.304939074383 | 30.49% | k\_\_Bacteria;p\_\_Bacteroidetes;c\_\_Bacteroidia;o\_\_Bacteroidales;f\_\_Muribaculaceae |
| 0.303327584745 | 30.33% | k\_\_Bacteria;p\_\_Firmicutes;c\_\_Clostridia;o\_\_Clostridiales;f\_\_Lachnospiraceae |
| 0.133145797405 | 13.31% | k\_\_Bacteria;p\_\_Bacteroidetes;c\_\_Bacteroidia;o\_\_Bacteroidales;f\_\_Bacteroidaceae |
| 0.091854909389 | 9.19% | k\_\_Bacteria;p\_\_Proteobacteria;c\_\_Gammaproteobacteria;o\_\_Enterobacteriales;f\_\_Enterobacteriaceae |
| 0.0894235390574 | 8.94% | k\_\_Bacteria;p\_\_Firmicutes;c\_\_Clostridia;o\_\_Clostridiales;f\_\_Ruminococcaceae |
| 0.0192671962907 | 1.93% | k\_\_Bacteria;p\_\_Bacteroidetes;c\_\_Bacteroidia;o\_\_Bacteroidales;f\_\_Prevotellaceae |
| 0.0128212377371 | 1.28% | k\_\_Bacteria;p\_\_Bacteroidetes;c\_\_Bacteroidia;o\_\_Bacteroidales;f\_\_Rikenellaceae |
| 0.0117751830596 | 1.18% | k\_\_Bacteria;p\_\_Proteobacteria;c\_\_Deltaproteobacteria;o\_\_Desulfovibrionales;f\_\_Desulfovibrionaceae |
| 0.00429730570241 | 0.43% | k\_\_Bacteria;p\_\_Firmicutes;c\_\_Bacilli;o\_\_Lactobacillales;f\_\_Lactobacillaceae |
| 0.00407113171807 | 0.41% | k\_\_Bacteria;p\_\_Verrucomicrobia;c\_\_Verrucomicrobiae;o\_\_Verrucomicrobiales;f\_\_Akkermansiaceae |
| 0.00395804472591 | 0.40% | k\_\_Bacteria;p\_\_Firmicutes;c\_\_Clostridia;o\_\_Clostridiales;Other |
| 0.00384495773374 | 0.38% | k\_\_Bacteria;p\_\_Bacteroidetes;c\_\_Bacteroidia;o\_\_Bacteroidales;f\_\_Tannerellaceae |
| 0.00296853354443 | 0.30% | k\_\_Bacteria;p\_\_Firmicutes;c\_\_Erysipelotrichia;o\_\_Erysipelotrichales;f\_\_Erysipelotrichaceae |
| 0.00286958242628 | 0.29% | k\_\_Bacteria;p\_\_Firmicutes;c\_\_Bacilli;o\_\_Lactobacillales;f\_\_Enterococcaceae |
| 0.00190834299285 | 0.19% | k\_\_Bacteria;p\_\_Oxyphotobacteria;c\_\_unidentified\_Oxyphotobacteria;o\_\_unidentified\_Oxyphotobacteria;f\_\_unidentified\_Oxyphotobacteria |
| 0.00171044075655 | 0.17% | k\_\_Bacteria;Other;Other;Other;Other |
| 0.001342908032 | 0.13% | k\_\_Bacteria;p\_\_Tenericutes;c\_\_Mollicutes;o\_\_;f\_\_ |
| 0.00128636453592 | 0.13% | k\_\_Bacteria;p\_\_Firmicutes;c\_\_Clostridia;o\_\_Clostridiales;f\_\_unidentified\_Clostridiales |
| 0.00115914166973 | 0.12% | k\_\_Bacteria;p\_\_Firmicutes;c\_\_Clostridia;o\_\_Clostridiales;f\_\_Peptococcaceae |
| 0.00077747307116 | 0.08% | k\_\_Bacteria;p\_\_Bacteroidetes;c\_\_Bacteroidia;o\_\_Bacteroidales;f\_\_Marinifilaceae |
| 0.000678521953012 | 0.07% | k\_\_Bacteria;p\_\_Proteobacteria;c\_\_Gammaproteobacteria;o\_\_unidentified\_Gammaproteobacteria;f\_\_Burkholderiaceae |
| 0.00025444573238 | 0.03% | k\_\_Bacteria;p\_\_unidentified\_Bacteria;c\_\_unidentified\_Bacteria;o\_\_unidentified\_Bacteria;f\_\_unidentified\_Bacteria |
| 0.00025444573238 | 0.03% | k\_\_Bacteria;p\_\_Fusobacteria;c\_\_Fusobacteriia;o\_\_Fusobacteriales;f\_\_Fusobacteriaceae |
| 0.00025444573238 | 0.03% | k\_\_Bacteria;p\_\_Firmicutes;Other;Other;Other |
| 0.00025444573238 | 0.03% | k\_\_Bacteria;p\_\_Actinobacteria;c\_\_unidentified\_Actinobacteria;o\_\_Bifidobacteriales;f\_\_Bifidobacteriaceae |
| 0.000240309858359 | 0.02% | k\_\_Bacteria;p\_\_Firmicutes;c\_\_Negativicutes;o\_\_Selenomonadales;f\_\_Veillonellaceae |
| 0.000183766362274 | 0.02% | k\_\_Bacteria;p\_\_Proteobacteria;c\_\_Gammaproteobacteria;o\_\_Pseudomonadales;f\_\_Moraxellaceae |
| 0.000169630488253 | 0.02% | k\_\_Bacteria;p\_\_Melainabacteria;Other;Other;Other |
| 0.000141358740211 | 0.01% | k\_\_Bacteria;p\_\_unidentified\_Bacteria;c\_\_unidentified\_Bacteria;o\_\_Campylobacterales;f\_\_unidentified\_Campylobacterales |
| 0.000113086992169 | 0.01% | k\_\_Bacteria;p\_\_Firmicutes;c\_\_Bacilli;o\_\_Lactobacillales;f\_\_Streptococcaceae |
| 8.48152441265e-05 | 0.01% | k\_\_Bacteria;p\_\_Proteobacteria;c\_\_Gammaproteobacteria;o\_\_Pseudomonadales;f\_\_Pseudomonadaceae |
| 8.48152441265e-05 | 0.01% | k\_\_Bacteria;p\_\_Firmicutes;c\_\_Negativicutes;o\_\_Selenomonadales;f\_\_Acidaminococcaceae |
| 7.06793701055e-05 | 0.01% | k\_\_Bacteria;p\_\_Firmicutes;c\_\_Clostridia;o\_\_Clostridiales;f\_\_Peptostreptococcaceae |
| 5.65434960844e-05 | 0.01% | k\_\_Bacteria;p\_\_Proteobacteria;c\_\_Gammaproteobacteria;o\_\_unidentified\_Gammaproteobacteria;f\_\_Neisseriaceae |
| 5.65434960844e-05 | 0.01% | k\_\_Bacteria;p\_\_Proteobacteria;c\_\_Gammaproteobacteria;o\_\_Pasteurellales;f\_\_Pasteurellaceae |
| 4.24076220633e-05 | 0.00% | k\_\_Bacteria;p\_\_Proteobacteria;c\_\_Alphaproteobacteria;o\_\_Rhodospirillales;f\_\_unidentified\_Rhodospirillales |
| 4.24076220633e-05 | 0.00% | k\_\_Bacteria;p\_\_Firmicutes;c\_\_Bacilli;o\_\_Lactobacillales;f\_\_Leuconostocaceae |
| 4.24076220633e-05 | 0.00% | k\_\_Bacteria;p\_\_Bacteroidetes;c\_\_Bacteroidia;o\_\_Bacteroidales;f\_\_Barnesiellaceae |
| 4.24076220633e-05 | 0.00% | k\_\_Bacteria;p\_\_Actinobacteria;c\_\_unidentified\_Actinobacteria;o\_\_Corynebacteriales;f\_\_Nocardiaceae |
| 4.24076220633e-05 | 0.00% | k\_\_Bacteria;p\_\_Actinobacteria;c\_\_Coriobacteriia;o\_\_Coriobacteriales;f\_\_Atopobiaceae |
| 2.82717480422e-05 | 0.00% | k\_\_Bacteria;p\_\_Proteobacteria;c\_\_Alphaproteobacteria;o\_\_Rhizobiales;f\_\_Rhizobiaceae |
| 2.82717480422e-05 | 0.00% | k\_\_Bacteria;p\_\_Firmicutes;c\_\_Bacilli;o\_\_Bacillales;f\_\_unidentified\_Bacillales |
| 2.82717480422e-05 | 0.00% | k\_\_Bacteria;p\_\_Bacteroidetes;c\_\_Bacteroidia;o\_\_Flavobacteriales;f\_\_Flavobacteriaceae |
| 1.41358740211e-05 | 0.00% | k\_\_Bacteria;p\_\_Tenericutes;c\_\_Mollicutes;o\_\_Anaeroplasmatales;f\_\_Anaeroplasmataceae |
| 1.41358740211e-05 | 0.00% | k\_\_Bacteria;p\_\_Firmicutes;c\_\_Clostridia;o\_\_Clostridiales;f\_\_Christensenellaceae |
| 1.41358740211e-05 | 0.00% | k\_\_Bacteria;p\_\_Bacteroidetes;c\_\_Bacteroidia;o\_\_Bacteroidales;f\_\_Porphyromonadaceae |
| 1.41358740211e-05 | 0.00% | k\_\_Bacteria;p\_\_Actinobacteria;c\_\_unidentified\_Actinobacteria;o\_\_Corynebacteriales;f\_\_Corynebacteriaceae |

|  |  |
| --- | --- |
|  | |
| Taxonomy Summary. Current Level: \_MD3 | |
| View Figure (.pdf)  View Legend (.pdf) |  |
|  |  |
| View Figure (.pdf)  View Legend (.pdf) |  |
|  |  |
|  |  |

|  |  |  |
| --- | --- | --- |
| Count | Pct | Taxonomy |
| 0.323895281445 | 32.39% | k\_\_Bacteria;p\_\_Bacteroidetes;c\_\_Bacteroidia;o\_\_Bacteroidales;f\_\_Muribaculaceae |
| 0.314212207741 | 31.42% | k\_\_Bacteria;p\_\_Firmicutes;c\_\_Clostridia;o\_\_Clostridiales;f\_\_Lachnospiraceae |
| 0.121511973085 | 12.15% | k\_\_Bacteria;p\_\_Bacteroidetes;c\_\_Bacteroidia;o\_\_Bacteroidales;f\_\_Bacteroidaceae |
| 0.0923779367278 | 9.24% | k\_\_Bacteria;p\_\_Proteobacteria;c\_\_Gammaproteobacteria;o\_\_Enterobacteriales;f\_\_Enterobacteriaceae |
| 0.0828927652597 | 8.29% | k\_\_Bacteria;p\_\_Firmicutes;c\_\_Clostridia;o\_\_Clostridiales;f\_\_Ruminococcaceae |
| 0.01342908032 | 1.34% | k\_\_Bacteria;p\_\_Proteobacteria;c\_\_Deltaproteobacteria;o\_\_Desulfovibrionales;f\_\_Desulfovibrionaceae |
| 0.0111249328546 | 1.11% | k\_\_Bacteria;p\_\_Bacteroidetes;c\_\_Bacteroidia;o\_\_Bacteroidales;f\_\_Prevotellaceae |
| 0.0102343727913 | 1.02% | k\_\_Bacteria;p\_\_Bacteroidetes;c\_\_Bacteroidia;o\_\_Bacteroidales;f\_\_Rikenellaceae |
| 0.00460829493088 | 0.46% | k\_\_Bacteria;p\_\_Firmicutes;c\_\_Erysipelotrichia;o\_\_Erysipelotrichales;f\_\_Erysipelotrichaceae |
| 0.00409940346612 | 0.41% | k\_\_Bacteria;p\_\_Firmicutes;c\_\_Bacilli;o\_\_Lactobacillales;f\_\_Lactobacillaceae |
| 0.00392977297786 | 0.39% | k\_\_Bacteria;p\_\_Bacteroidetes;c\_\_Bacteroidia;o\_\_Bacteroidales;f\_\_Tannerellaceae |
| 0.00337847389104 | 0.34% | k\_\_Bacteria;p\_\_Verrucomicrobia;c\_\_Verrucomicrobiae;o\_\_Verrucomicrobiales;f\_\_Akkermansiaceae |
| 0.00336433801702 | 0.34% | k\_\_Bacteria;p\_\_Firmicutes;c\_\_Bacilli;o\_\_Lactobacillales;f\_\_Enterococcaceae |
| 0.00197902236295 | 0.20% | k\_\_Bacteria;p\_\_Firmicutes;c\_\_Clostridia;o\_\_Clostridiales;Other |
| 0.00104605467756 | 0.10% | k\_\_Bacteria;p\_\_Firmicutes;c\_\_Clostridia;o\_\_Clostridiales;f\_\_unidentified\_Clostridiales |
| 0.000932967685392 | 0.09% | k\_\_Bacteria;Other;Other;Other;Other |
| 0.000890560063329 | 0.09% | k\_\_Bacteria;p\_\_Firmicutes;c\_\_Clostridia;o\_\_Clostridiales;f\_\_Peptococcaceae |
| 0.000819880693223 | 0.08% | k\_\_Bacteria;p\_\_Tenericutes;c\_\_Mollicutes;o\_\_;f\_\_ |
| 0.000819880693223 | 0.08% | k\_\_Bacteria;p\_\_Oxyphotobacteria;c\_\_unidentified\_Oxyphotobacteria;o\_\_unidentified\_Oxyphotobacteria;f\_\_unidentified\_Oxyphotobacteria |
| 0.000763337197139 | 0.08% | k\_\_Bacteria;p\_\_Bacteroidetes;c\_\_Bacteroidia;o\_\_Bacteroidales;f\_\_Marinifilaceae |
| 0.000466483842696 | 0.05% | k\_\_Bacteria;p\_\_Proteobacteria;c\_\_Gammaproteobacteria;o\_\_unidentified\_Gammaproteobacteria;f\_\_Burkholderiaceae |
| 0.000395804472591 | 0.04% | k\_\_Bacteria;p\_\_Firmicutes;c\_\_Negativicutes;o\_\_Selenomonadales;f\_\_Veillonellaceae |
| 0.000296853354443 | 0.03% | k\_\_Bacteria;p\_\_Firmicutes;Other;Other;Other |
| 0.000212038110316 | 0.02% | k\_\_Bacteria;p\_\_Fusobacteria;c\_\_Fusobacteriia;o\_\_Fusobacteriales;f\_\_Fusobacteriaceae |
| 0.000183766362274 | 0.02% | k\_\_Bacteria;p\_\_unidentified\_Bacteria;c\_\_unidentified\_Bacteria;o\_\_Campylobacterales;f\_\_unidentified\_Campylobacterales |
| 0.000183766362274 | 0.02% | k\_\_Bacteria;p\_\_Proteobacteria;c\_\_Alphaproteobacteria;o\_\_unidentified\_Alphaproteobacteria;f\_\_unidentified\_Alphaproteobacteria |
| 0.000155494614232 | 0.02% | k\_\_Bacteria;p\_\_Actinobacteria;c\_\_Rubrobacteria;o\_\_Rubrobacterales;f\_\_unidentified\_Rubrobacterales |
| 0.000141358740211 | 0.01% | k\_\_Bacteria;p\_\_Proteobacteria;c\_\_Gammaproteobacteria;o\_\_Pasteurellales;f\_\_Pasteurellaceae |
| 0.000141358740211 | 0.01% | k\_\_Bacteria;p\_\_Actinobacteria;c\_\_unidentified\_Actinobacteria;o\_\_Bifidobacteriales;f\_\_Bifidobacteriaceae |
| 0.00012722286619 | 0.01% | k\_\_Bacteria;p\_\_unidentified\_Bacteria;c\_\_unidentified\_Bacteria;o\_\_unidentified\_Bacteria;f\_\_unidentified\_Bacteria |
| 0.000113086992169 | 0.01% | k\_\_Bacteria;p\_\_Proteobacteria;c\_\_Gammaproteobacteria;o\_\_Pseudomonadales;f\_\_Pseudomonadaceae |
| 0.000113086992169 | 0.01% | k\_\_Bacteria;p\_\_Firmicutes;c\_\_Bacilli;o\_\_Lactobacillales;f\_\_Streptococcaceae |
| 9.89511181476e-05 | 0.01% | k\_\_Bacteria;p\_\_Proteobacteria;c\_\_Gammaproteobacteria;o\_\_Pseudomonadales;f\_\_Moraxellaceae |
| 8.48152441265e-05 | 0.01% | k\_\_Bacteria;p\_\_Melainabacteria;Other;Other;Other |
| 7.06793701055e-05 | 0.01% | k\_\_Bacteria;p\_\_Proteobacteria;c\_\_Alphaproteobacteria;o\_\_Rhizobiales;Other |
| 7.06793701055e-05 | 0.01% | k\_\_Bacteria;p\_\_Bacteroidetes;c\_\_Bacteroidia;o\_\_Bacteroidales;f\_\_Porphyromonadaceae |
| 7.06793701055e-05 | 0.01% | k\_\_Bacteria;p\_\_Acidobacteria;c\_\_unidentified\_Acidobacteria;o\_\_unidentified\_Acidobacteria;f\_\_Pyrinomonadaceae |
| 5.65434960844e-05 | 0.01% | k\_\_Bacteria;p\_\_Proteobacteria;c\_\_Alphaproteobacteria;o\_\_Sphingomonadales;f\_\_Sphingomonadaceae |
| 5.65434960844e-05 | 0.01% | k\_\_Bacteria;p\_\_Firmicutes;c\_\_Clostridia;o\_\_Clostridiales;f\_\_Peptostreptococcaceae |
| 5.65434960844e-05 | 0.01% | k\_\_Bacteria;p\_\_Bacteroidetes;c\_\_Bacteroidia;o\_\_Bacteroidales;f\_\_Barnesiellaceae |
| 5.65434960844e-05 | 0.01% | k\_\_Bacteria;p\_\_Actinobacteria;c\_\_unidentified\_Actinobacteria;o\_\_Propionibacteriales;f\_\_Nocardioidaceae |
| 5.65434960844e-05 | 0.01% | k\_\_Bacteria;p\_\_Actinobacteria;c\_\_Thermoleophilia;o\_\_Solirubrobacterales;f\_\_ |
| 5.65434960844e-05 | 0.01% | k\_\_Bacteria;p\_\_Actinobacteria;c\_\_Acidimicrobiia;Other;Other |
| 5.65434960844e-05 | 0.01% | k\_\_Bacteria;p\_\_Acidobacteria;c\_\_Holophagae;o\_\_;f\_\_ |
| 4.24076220633e-05 | 0.00% | k\_\_Bacteria;p\_\_Proteobacteria;c\_\_Gammaproteobacteria;o\_\_Xanthomonadales;f\_\_Xanthomonadaceae |
| 4.24076220633e-05 | 0.00% | k\_\_Bacteria;p\_\_Proteobacteria;c\_\_Alphaproteobacteria;o\_\_Rhodospirillales;f\_\_unidentified\_Rhodospirillales |
| 4.24076220633e-05 | 0.00% | k\_\_Bacteria;p\_\_Firmicutes;c\_\_Negativicutes;o\_\_Selenomonadales;f\_\_Acidaminococcaceae |
| 4.24076220633e-05 | 0.00% | k\_\_Bacteria;p\_\_Bacteroidetes;c\_\_Bacteroidia;o\_\_Cytophagales;Other |
| 4.24076220633e-05 | 0.00% | k\_\_Bacteria;p\_\_Actinobacteria;c\_\_unidentified\_Actinobacteria;o\_\_Corynebacteriales;f\_\_Nocardiaceae |
| 4.24076220633e-05 | 0.00% | k\_\_Bacteria;p\_\_Acidobacteria;c\_\_unidentified\_Acidobacteria;o\_\_unidentified\_Acidobacteria;f\_\_unidentified\_Acidobacteria |
| 2.82717480422e-05 | 0.00% | k\_\_Bacteria;p\_\_Actinobacteria;c\_\_Thermoleophilia;o\_\_Solirubrobacterales;Other |
| 2.82717480422e-05 | 0.00% | k\_\_Bacteria;p\_\_Actinobacteria;c\_\_Acidimicrobiia;o\_\_;f\_\_ |
| 1.41358740211e-05 | 0.00% | k\_\_Bacteria;p\_\_Proteobacteria;c\_\_Alphaproteobacteria;o\_\_Rhizobiales;f\_\_Xanthobacteraceae |
| 1.41358740211e-05 | 0.00% | k\_\_Bacteria;p\_\_Firmicutes;c\_\_Clostridia;o\_\_Clostridiales;f\_\_Christensenellaceae |
| 1.41358740211e-05 | 0.00% | k\_\_Bacteria;p\_\_Firmicutes;c\_\_Bacilli;o\_\_Bacillales;f\_\_Staphylococcaceae |
| 1.41358740211e-05 | 0.00% | k\_\_Bacteria;p\_\_Actinobacteria;c\_\_unidentified\_Actinobacteria;o\_\_Actinomycetales;f\_\_Actinomycetaceae |

|  |  |
| --- | --- |
|  | |
| Taxonomy Summary. Current Level: \_MD4 | |
| View Figure (.pdf)  View Legend (.pdf) |  |
|  |  |
| View Figure (.pdf)  View Legend (.pdf) |  |
|  |  |
|  |  |

|  |  |  |
| --- | --- | --- |
| Count | Pct | Taxonomy |
| 0.329196234203 | 32.92% | k\_\_Bacteria;p\_\_Bacteroidetes;c\_\_Bacteroidia;o\_\_Bacteroidales;f\_\_Muribaculaceae |
| 0.291750303921 | 29.18% | k\_\_Bacteria;p\_\_Bacteroidetes;c\_\_Bacteroidia;o\_\_Bacteroidales;f\_\_Bacteroidaceae |
| 0.137754092336 | 13.78% | k\_\_Bacteria;p\_\_Firmicutes;c\_\_Clostridia;o\_\_Clostridiales;f\_\_Lachnospiraceae |
| 0.0753300726584 | 7.53% | k\_\_Bacteria;p\_\_Proteobacteria;c\_\_Gammaproteobacteria;o\_\_Enterobacteriales;f\_\_Enterobacteriaceae |
| 0.0667354612536 | 6.67% | k\_\_Bacteria;p\_\_Firmicutes;c\_\_Clostridia;o\_\_Clostridiales;f\_\_Ruminococcaceae |
| 0.0297701506884 | 2.98% | k\_\_Bacteria;p\_\_Bacteroidetes;c\_\_Bacteroidia;o\_\_Bacteroidales;f\_\_Prevotellaceae |
| 0.023437279127 | 2.34% | k\_\_Bacteria;p\_\_Bacteroidetes;c\_\_Bacteroidia;o\_\_Bacteroidales;f\_\_Rikenellaceae |
| 0.0103333239094 | 1.03% | k\_\_Bacteria;p\_\_Verrucomicrobia;c\_\_Verrucomicrobiae;o\_\_Verrucomicrobiales;f\_\_Akkermansiaceae |
| 0.00802917644398 | 0.80% | k\_\_Bacteria;p\_\_Proteobacteria;c\_\_Deltaproteobacteria;o\_\_Desulfovibrionales;f\_\_Desulfovibrionaceae |
| 0.00699725764044 | 0.70% | k\_\_Bacteria;p\_\_Bacteroidetes;c\_\_Bacteroidia;o\_\_Bacteroidales;f\_\_Tannerellaceae |
| 0.00498996352945 | 0.50% | k\_\_Bacteria;p\_\_Firmicutes;c\_\_Bacilli;o\_\_Lactobacillales;f\_\_Enterococcaceae |
| 0.00477792541913 | 0.48% | k\_\_Bacteria;p\_\_Firmicutes;c\_\_Bacilli;o\_\_Lactobacillales;f\_\_Lactobacillaceae |
| 0.00233241921348 | 0.23% | k\_\_Bacteria;p\_\_Firmicutes;c\_\_Clostridia;o\_\_Clostridiales;Other |
| 0.0016114896384 | 0.16% | k\_\_Bacteria;p\_\_Firmicutes;c\_\_Erysipelotrichia;o\_\_Erysipelotrichales;f\_\_Erysipelotrichaceae |
| 0.00148426677221 | 0.15% | k\_\_Bacteria;p\_\_Bacteroidetes;c\_\_Bacteroidia;o\_\_Bacteroidales;f\_\_Marinifilaceae |
| 0.00135704390602 | 0.14% | k\_\_Bacteria;p\_\_Firmicutes;c\_\_Clostridia;o\_\_Clostridiales;f\_\_unidentified\_Clostridiales |
| 0.000692657827033 | 0.07% | k\_\_Bacteria;p\_\_unidentified\_Bacteria;c\_\_unidentified\_Bacteria;o\_\_unidentified\_Bacteria;f\_\_unidentified\_Bacteria |
| 0.00052302733878 | 0.05% | k\_\_Bacteria;p\_\_Proteobacteria;c\_\_Gammaproteobacteria;o\_\_unidentified\_Gammaproteobacteria;f\_\_Burkholderiaceae |
| 0.000325125102485 | 0.03% | k\_\_Bacteria;p\_\_Fusobacteria;c\_\_Fusobacteriia;o\_\_Fusobacteriales;f\_\_Fusobacteriaceae |
| 0.000325125102485 | 0.03% | k\_\_Bacteria;p\_\_Firmicutes;c\_\_Clostridia;o\_\_Clostridiales;f\_\_Peptococcaceae |
| 0.000296853354443 | 0.03% | k\_\_Bacteria;p\_\_Melainabacteria;Other;Other;Other |
| 0.000296853354443 | 0.03% | k\_\_Bacteria;p\_\_Firmicutes;c\_\_Negativicutes;o\_\_Selenomonadales;f\_\_Veillonellaceae |
| 0.000282717480422 | 0.03% | k\_\_Bacteria;p\_\_Tenericutes;c\_\_Mollicutes;o\_\_;f\_\_ |
| 0.000197902236295 | 0.02% | k\_\_Bacteria;p\_\_Actinobacteria;c\_\_unidentified\_Actinobacteria;o\_\_Bifidobacteriales;f\_\_Bifidobacteriaceae |
| 0.000183766362274 | 0.02% | k\_\_Bacteria;p\_\_Bacteroidetes;c\_\_Bacteroidia;o\_\_Bacteroidales;f\_\_Barnesiellaceae |
| 0.000113086992169 | 0.01% | k\_\_Bacteria;p\_\_Proteobacteria;c\_\_Gammaproteobacteria;o\_\_Pseudomonadales;f\_\_Moraxellaceae |
| 0.000113086992169 | 0.01% | k\_\_Bacteria;p\_\_Firmicutes;c\_\_Clostridia;o\_\_Clostridiales;f\_\_Peptostreptococcaceae |
| 0.000113086992169 | 0.01% | k\_\_Bacteria;p\_\_Firmicutes;c\_\_Bacilli;o\_\_Lactobacillales;f\_\_Streptococcaceae |
| 0.000113086992169 | 0.01% | k\_\_Bacteria;p\_\_Bacteroidetes;c\_\_Bacteroidia;o\_\_Bacteroidales;f\_\_Porphyromonadaceae |
| 8.48152441265e-05 | 0.01% | k\_\_Bacteria;p\_\_Proteobacteria;c\_\_Gammaproteobacteria;o\_\_Pasteurellales;f\_\_Pasteurellaceae |
| 7.06793701055e-05 | 0.01% | k\_\_Bacteria;p\_\_Oxyphotobacteria;c\_\_unidentified\_Oxyphotobacteria;o\_\_unidentified\_Oxyphotobacteria;f\_\_unidentified\_Oxyphotobacteria |
| 7.06793701055e-05 | 0.01% | k\_\_Bacteria;p\_\_Firmicutes;Other;Other;Other |
| 5.65434960844e-05 | 0.01% | k\_\_Bacteria;p\_\_Proteobacteria;c\_\_Alphaproteobacteria;o\_\_Rhizobiales;f\_\_Rhizobiaceae |
| 5.65434960844e-05 | 0.01% | k\_\_Bacteria;p\_\_Actinobacteria;c\_\_unidentified\_Actinobacteria;o\_\_Corynebacteriales;f\_\_Nocardiaceae |
| 4.24076220633e-05 | 0.00% | k\_\_Bacteria;p\_\_Firmicutes;c\_\_Negativicutes;o\_\_Selenomonadales;f\_\_Acidaminococcaceae |
| 2.82717480422e-05 | 0.00% | k\_\_Bacteria;p\_\_unidentified\_Bacteria;c\_\_unidentified\_Bacteria;o\_\_Campylobacterales;f\_\_unidentified\_Campylobacterales |
| 2.82717480422e-05 | 0.00% | k\_\_Bacteria;p\_\_Proteobacteria;c\_\_Gammaproteobacteria;o\_\_Xanthomonadales;f\_\_Xanthomonadaceae |
| 2.82717480422e-05 | 0.00% | k\_\_Bacteria;p\_\_Proteobacteria;c\_\_Gammaproteobacteria;o\_\_Pseudomonadales;f\_\_Pseudomonadaceae |
| 2.82717480422e-05 | 0.00% | k\_\_Bacteria;p\_\_Proteobacteria;c\_\_Alphaproteobacteria;o\_\_Sphingomonadales;f\_\_Sphingomonadaceae |
| 1.41358740211e-05 | 0.00% | k\_\_Bacteria;p\_\_Proteobacteria;c\_\_Gammaproteobacteria;o\_\_unidentified\_Gammaproteobacteria;f\_\_Neisseriaceae |
| 1.41358740211e-05 | 0.00% | k\_\_Bacteria;p\_\_Firmicutes;c\_\_Bacilli;o\_\_Bacillales;f\_\_unidentified\_Bacillales |
| 1.41358740211e-05 | 0.00% | k\_\_Bacteria;p\_\_Actinobacteria;c\_\_unidentified\_Actinobacteria;o\_\_Micrococcales;f\_\_Microbacteriaceae |

|  |  |
| --- | --- |
|  | |
| Taxonomy Summary. Current Level: \_MD5 | |
| View Figure (.pdf)  View Legend (.pdf) |  |
|  |  |
| View Figure (.pdf)  View Legend (.pdf) |  |
|  |  |
|  |  |

|  |  |  |
| --- | --- | --- |
| Count | Pct | Taxonomy |
| 0.331316615306 | 33.13% | k\_\_Bacteria;p\_\_Firmicutes;c\_\_Clostridia;o\_\_Clostridiales;f\_\_Lachnospiraceae |
| 0.3003590512 | 30.04% | k\_\_Bacteria;p\_\_Bacteroidetes;c\_\_Bacteroidia;o\_\_Bacteroidales;f\_\_Muribaculaceae |
| 0.171128890899 | 17.11% | k\_\_Bacteria;p\_\_Bacteroidetes;c\_\_Bacteroidia;o\_\_Bacteroidales;f\_\_Bacteroidaceae |
| 0.0959401769811 | 9.59% | k\_\_Bacteria;p\_\_Firmicutes;c\_\_Clostridia;o\_\_Clostridiales;f\_\_Ruminococcaceae |
| 0.0193944191569 | 1.94% | k\_\_Bacteria;p\_\_Proteobacteria;c\_\_Gammaproteobacteria;o\_\_Enterobacteriales;f\_\_Enterobacteriaceae |
| 0.0138248847926 | 1.38% | k\_\_Bacteria;p\_\_Bacteroidetes;c\_\_Bacteroidia;o\_\_Bacteroidales;f\_\_Rikenellaceae |
| 0.0136128466823 | 1.36% | k\_\_Bacteria;p\_\_Proteobacteria;c\_\_Deltaproteobacteria;o\_\_Desulfovibrionales;f\_\_Desulfovibrionaceae |
| 0.0119872211699 | 1.20% | k\_\_Bacteria;p\_\_Firmicutes;c\_\_Bacilli;o\_\_Lactobacillales;f\_\_Lactobacillaceae |
| 0.0106019055158 | 1.06% | k\_\_Bacteria;p\_\_Firmicutes;c\_\_Bacilli;o\_\_Lactobacillales;f\_\_Enterococcaceae |
| 0.00880664951514 | 0.88% | k\_\_Bacteria;p\_\_Bacteroidetes;c\_\_Bacteroidia;o\_\_Bacteroidales;f\_\_Prevotellaceae |
| 0.00530095275791 | 0.53% | k\_\_Bacteria;p\_\_Bacteroidetes;c\_\_Bacteroidia;o\_\_Bacteroidales;f\_\_Tannerellaceae |
| 0.004693110175 | 0.47% | k\_\_Bacteria;p\_\_Firmicutes;c\_\_Clostridia;o\_\_Clostridiales;Other |
| 0.0041559469622 | 0.42% | k\_\_Bacteria;p\_\_Tenericutes;c\_\_Mollicutes;o\_\_;f\_\_ |
| 0.00213451697718 | 0.21% | k\_\_Bacteria;p\_\_Firmicutes;c\_\_Clostridia;o\_\_Clostridiales;f\_\_unidentified\_Clostridiales |
| 0.00117327754375 | 0.12% | k\_\_Bacteria;p\_\_Firmicutes;c\_\_Erysipelotrichia;o\_\_Erysipelotrichales;f\_\_Erysipelotrichaceae |
| 0.000805744819202 | 0.08% | k\_\_Bacteria;p\_\_Bacteroidetes;c\_\_Bacteroidia;o\_\_Bacteroidales;f\_\_Marinifilaceae |
| 0.000607842582907 | 0.06% | k\_\_Bacteria;p\_\_Firmicutes;c\_\_Negativicutes;o\_\_Selenomonadales;f\_\_Veillonellaceae |
| 0.000593706708886 | 0.06% | k\_\_Bacteria;p\_\_Firmicutes;Other;Other;Other |
| 0.000452347968675 | 0.05% | k\_\_Bacteria;p\_\_unidentified\_Bacteria;c\_\_unidentified\_Bacteria;o\_\_unidentified\_Bacteria;f\_\_unidentified\_Bacteria |
| 0.000395804472591 | 0.04% | k\_\_Bacteria;p\_\_Verrucomicrobia;c\_\_Verrucomicrobiae;o\_\_Verrucomicrobiales;f\_\_Akkermansiaceae |
| 0.000395804472591 | 0.04% | k\_\_Bacteria;p\_\_Firmicutes;c\_\_Clostridia;o\_\_Clostridiales;f\_\_Peptococcaceae |
| 0.000353396850527 | 0.04% | k\_\_Bacteria;p\_\_Proteobacteria;c\_\_Gammaproteobacteria;o\_\_unidentified\_Gammaproteobacteria;f\_\_Burkholderiaceae |
| 0.000268581606401 | 0.03% | k\_\_Bacteria;p\_\_Fusobacteria;c\_\_Fusobacteriia;o\_\_Fusobacteriales;f\_\_Fusobacteriaceae |
| 0.000268581606401 | 0.03% | k\_\_Bacteria;Other;Other;Other;Other |
| 0.000169630488253 | 0.02% | k\_\_Bacteria;p\_\_Actinobacteria;c\_\_unidentified\_Actinobacteria;o\_\_Bifidobacteriales;f\_\_Bifidobacteriaceae |
| 0.000155494614232 | 0.02% | k\_\_Bacteria;p\_\_Firmicutes;c\_\_Bacilli;o\_\_Lactobacillales;f\_\_Streptococcaceae |
| 0.00012722286619 | 0.01% | k\_\_Bacteria;p\_\_Proteobacteria;c\_\_Gammaproteobacteria;o\_\_Xanthomonadales;f\_\_Xanthomonadaceae |
| 0.00012722286619 | 0.01% | k\_\_Bacteria;p\_\_Firmicutes;c\_\_Negativicutes;o\_\_Selenomonadales;f\_\_Acidaminococcaceae |
| 0.000113086992169 | 0.01% | k\_\_Bacteria;p\_\_Firmicutes;c\_\_Clostridia;o\_\_Clostridiales;f\_\_Peptostreptococcaceae |
| 9.89511181476e-05 | 0.01% | k\_\_Bacteria;p\_\_Bacteroidetes;c\_\_Bacteroidia;o\_\_Bacteroidales;f\_\_Porphyromonadaceae |
| 8.48152441265e-05 | 0.01% | k\_\_Bacteria;p\_\_Melainabacteria;Other;Other;Other |
| 8.48152441265e-05 | 0.01% | k\_\_Bacteria;p\_\_Firmicutes;c\_\_Clostridia;o\_\_Clostridiales;f\_\_Eubacteriaceae |
| 7.06793701055e-05 | 0.01% | k\_\_Bacteria;p\_\_Proteobacteria;c\_\_Alphaproteobacteria;o\_\_Sphingomonadales;f\_\_Sphingomonadaceae |
| 5.65434960844e-05 | 0.01% | k\_\_Bacteria;p\_\_unidentified\_Bacteria;c\_\_unidentified\_Bacteria;o\_\_Campylobacterales;f\_\_unidentified\_Campylobacterales |
| 4.24076220633e-05 | 0.00% | k\_\_Bacteria;p\_\_Proteobacteria;c\_\_Gammaproteobacteria;o\_\_Pseudomonadales;f\_\_Moraxellaceae |
| 4.24076220633e-05 | 0.00% | k\_\_Bacteria;p\_\_Proteobacteria;Other;Other;Other |
| 4.24076220633e-05 | 0.00% | k\_\_Bacteria;p\_\_Bacteroidetes;c\_\_Bacteroidia;o\_\_Bacteroidales;f\_\_Barnesiellaceae |
| 2.82717480422e-05 | 0.00% | k\_\_Bacteria;p\_\_Proteobacteria;c\_\_Gammaproteobacteria;o\_\_unidentified\_Gammaproteobacteria;f\_\_Neisseriaceae |
| 2.82717480422e-05 | 0.00% | k\_\_Bacteria;p\_\_Proteobacteria;c\_\_Gammaproteobacteria;o\_\_Pseudomonadales;f\_\_Pseudomonadaceae |
| 2.82717480422e-05 | 0.00% | k\_\_Bacteria;p\_\_Bacteroidetes;c\_\_Bacteroidia;o\_\_Flavobacteriales;f\_\_Flavobacteriaceae |
| 1.41358740211e-05 | 0.00% | k\_\_Bacteria;p\_\_Proteobacteria;c\_\_Gammaproteobacteria;o\_\_Pasteurellales;f\_\_Pasteurellaceae |
| 1.41358740211e-05 | 0.00% | k\_\_Bacteria;p\_\_Proteobacteria;c\_\_Alphaproteobacteria;o\_\_Rhodospirillales;f\_\_unidentified\_Rhodospirillales |
| 1.41358740211e-05 | 0.00% | k\_\_Bacteria;p\_\_Proteobacteria;c\_\_Alphaproteobacteria;o\_\_Rhizobiales;f\_\_Xanthobacteraceae |
| 1.41358740211e-05 | 0.00% | k\_\_Bacteria;p\_\_Proteobacteria;c\_\_Alphaproteobacteria;o\_\_Rhizobiales;f\_\_Rhizobiaceae |
| 1.41358740211e-05 | 0.00% | k\_\_Bacteria;p\_\_Proteobacteria;c\_\_Alphaproteobacteria;o\_\_Rhizobiales;f\_\_Beijerinckiaceae |
| 1.41358740211e-05 | 0.00% | k\_\_Bacteria;p\_\_Oxyphotobacteria;c\_\_unidentified\_Oxyphotobacteria;o\_\_unidentified\_Oxyphotobacteria;f\_\_unidentified\_Oxyphotobacteria |
| 1.41358740211e-05 | 0.00% | k\_\_Bacteria;p\_\_Firmicutes;c\_\_Bacilli;o\_\_Bacillales;f\_\_unidentified\_Bacillales |
| 1.41358740211e-05 | 0.00% | k\_\_Bacteria;p\_\_Actinobacteria;c\_\_unidentified\_Actinobacteria;o\_\_Corynebacteriales;f\_\_Corynebacteriaceae |
| 1.41358740211e-05 | 0.00% | k\_\_Bacteria;p\_\_Actinobacteria;c\_\_Coriobacteriia;o\_\_Coriobacteriales;f\_\_Atopobiaceae |

|  |  |
| --- | --- |
|  | |
| Taxonomy Summary. Current Level: \_MC1 | |
| View Figure (.pdf)  View Legend (.pdf) |  |
|  |  |
| View Figure (.pdf)  View Legend (.pdf) |  |
|  |  |
|  |  |

|  |  |  |
| --- | --- | --- |
| Count | Pct | Taxonomy |
| 0.36996409488 | 37.00% | k\_\_Bacteria;p\_\_Bacteroidetes;c\_\_Bacteroidia;o\_\_Bacteroidales;f\_\_Muribaculaceae |
| 0.155155353255 | 15.52% | k\_\_Bacteria;p\_\_Bacteroidetes;c\_\_Bacteroidia;o\_\_Bacteroidales;f\_\_Bacteroidaceae |
| 0.13639704843 | 13.64% | k\_\_Bacteria;p\_\_Firmicutes;c\_\_Clostridia;o\_\_Clostridiales;f\_\_Ruminococcaceae |
| 0.124862175228 | 12.49% | k\_\_Bacteria;p\_\_Firmicutes;c\_\_Clostridia;o\_\_Clostridiales;f\_\_Lachnospiraceae |
| 0.0867801306155 | 8.68% | k\_\_Bacteria;p\_\_Verrucomicrobia;c\_\_Verrucomicrobiae;o\_\_Verrucomicrobiales;f\_\_Akkermansiaceae |
| 0.0290774928614 | 2.91% | k\_\_Bacteria;p\_\_Proteobacteria;c\_\_Gammaproteobacteria;o\_\_Enterobacteriales;f\_\_Enterobacteriaceae |
| 0.0251901275056 | 2.52% | k\_\_Bacteria;p\_\_Tenericutes;c\_\_Mollicutes;o\_\_;f\_\_ |
| 0.0177546577705 | 1.78% | k\_\_Bacteria;p\_\_Bacteroidetes;c\_\_Bacteroidia;o\_\_Bacteroidales;f\_\_Rikenellaceae |
| 0.0119872211699 | 1.20% | k\_\_Bacteria;p\_\_Bacteroidetes;c\_\_Bacteroidia;o\_\_Bacteroidales;f\_\_Prevotellaceae |
| 0.0103474597834 | 1.03% | k\_\_Bacteria;p\_\_Firmicutes;c\_\_Erysipelotrichia;o\_\_Erysipelotrichales;f\_\_Erysipelotrichaceae |
| 0.00702552938848 | 0.70% | k\_\_Bacteria;p\_\_Bacteroidetes;c\_\_Bacteroidia;o\_\_Bacteroidales;f\_\_Tannerellaceae |
| 0.00453761556077 | 0.45% | k\_\_Bacteria;p\_\_Proteobacteria;c\_\_Deltaproteobacteria;o\_\_Desulfovibrionales;f\_\_Desulfovibrionaceae |
| 0.00432557745045 | 0.43% | k\_\_Bacteria;p\_\_Firmicutes;c\_\_Bacilli;o\_\_Lactobacillales;f\_\_Lactobacillaceae |
| 0.00286958242628 | 0.29% | k\_\_Bacteria;p\_\_Firmicutes;c\_\_Clostridia;o\_\_Clostridiales;Other |
| 0.00278476718215 | 0.28% | k\_\_Bacteria;p\_\_Firmicutes;c\_\_Bacilli;o\_\_Lactobacillales;f\_\_Enterococcaceae |
| 0.00166803313449 | 0.17% | k\_\_Bacteria;p\_\_Melainabacteria;Other;Other;Other |
| 0.00145599502417 | 0.15% | k\_\_Bacteria;p\_\_Firmicutes;c\_\_Clostridia;o\_\_Clostridiales;f\_\_unidentified\_Clostridiales |
| 0.00138531565407 | 0.14% | k\_\_Bacteria;p\_\_Bacteroidetes;c\_\_Bacteroidia;o\_\_Bacteroidales;f\_\_Marinifilaceae |
| 0.0012722286619 | 0.13% | k\_\_Bacteria;Other;Other;Other;Other |
| 0.00104605467756 | 0.10% | k\_\_Bacteria;p\_\_Oxyphotobacteria;c\_\_unidentified\_Oxyphotobacteria;o\_\_unidentified\_Oxyphotobacteria;f\_\_unidentified\_Oxyphotobacteria |
| 0.000593706708886 | 0.06% | k\_\_Bacteria;p\_\_unidentified\_Bacteria;c\_\_unidentified\_Bacteria;o\_\_unidentified\_Bacteria;f\_\_unidentified\_Bacteria |
| 0.000480619716717 | 0.05% | k\_\_Bacteria;p\_\_Actinobacteria;c\_\_unidentified\_Actinobacteria;o\_\_Bifidobacteriales;f\_\_Bifidobacteriaceae |
| 0.000409940346612 | 0.04% | k\_\_Bacteria;p\_\_Proteobacteria;c\_\_Gammaproteobacteria;o\_\_unidentified\_Gammaproteobacteria;f\_\_Burkholderiaceae |
| 0.000395804472591 | 0.04% | k\_\_Bacteria;p\_\_Fusobacteria;c\_\_Fusobacteriia;o\_\_Fusobacteriales;f\_\_Fusobacteriaceae |
| 0.000296853354443 | 0.03% | k\_\_Bacteria;p\_\_Firmicutes;c\_\_Negativicutes;o\_\_Selenomonadales;f\_\_Veillonellaceae |
| 0.00025444573238 | 0.03% | k\_\_Bacteria;p\_\_Firmicutes;c\_\_Clostridia;o\_\_Clostridiales;f\_\_Peptococcaceae |
| 0.000197902236295 | 0.02% | k\_\_Bacteria;p\_\_Proteobacteria;c\_\_Gammaproteobacteria;o\_\_Pseudomonadales;f\_\_Moraxellaceae |
| 0.000183766362274 | 0.02% | k\_\_Bacteria;p\_\_Firmicutes;c\_\_Clostridia;o\_\_Clostridiales;f\_\_Peptostreptococcaceae |
| 0.000183766362274 | 0.02% | k\_\_Bacteria;p\_\_Firmicutes;Other;Other;Other |
| 0.000113086992169 | 0.01% | k\_\_Bacteria;p\_\_Proteobacteria;c\_\_Gammaproteobacteria;o\_\_Pseudomonadales;f\_\_Pseudomonadaceae |
| 0.000113086992169 | 0.01% | k\_\_Bacteria;p\_\_Firmicutes;c\_\_Negativicutes;o\_\_Selenomonadales;f\_\_Acidaminococcaceae |
| 0.000113086992169 | 0.01% | k\_\_Bacteria;p\_\_Bacteroidetes;c\_\_Bacteroidia;o\_\_Bacteroidales;f\_\_Barnesiellaceae |
| 9.89511181476e-05 | 0.01% | k\_\_Bacteria;p\_\_Bacteroidetes;c\_\_Bacteroidia;o\_\_Bacteroidales;f\_\_Porphyromonadaceae |
| 7.06793701055e-05 | 0.01% | k\_\_Bacteria;p\_\_Firmicutes;c\_\_Bacilli;o\_\_Lactobacillales;f\_\_Streptococcaceae |
| 7.06793701055e-05 | 0.01% | k\_\_Bacteria;p\_\_Bacteroidetes;c\_\_Bacteroidia;o\_\_Flavobacteriales;f\_\_unidentified\_Flavobacteriales |
| 5.65434960844e-05 | 0.01% | k\_\_Bacteria;p\_\_unidentified\_Bacteria;c\_\_unidentified\_Bacteria;o\_\_Campylobacterales;f\_\_unidentified\_Campylobacterales |
| 5.65434960844e-05 | 0.01% | k\_\_Bacteria;p\_\_Proteobacteria;c\_\_Alphaproteobacteria;o\_\_Sphingomonadales;f\_\_Sphingomonadaceae |
| 5.65434960844e-05 | 0.01% | k\_\_Bacteria;p\_\_Firmicutes;c\_\_Clostridia;o\_\_Clostridiales;f\_\_Eubacteriaceae |
| 4.24076220633e-05 | 0.00% | k\_\_Bacteria;p\_\_Proteobacteria;c\_\_Gammaproteobacteria;o\_\_Pasteurellales;f\_\_Pasteurellaceae |
| 4.24076220633e-05 | 0.00% | k\_\_Bacteria;p\_\_Proteobacteria;c\_\_Alphaproteobacteria;o\_\_Rhizobiales;f\_\_Rhizobiaceae |
| 4.24076220633e-05 | 0.00% | k\_\_Bacteria;p\_\_Firmicutes;c\_\_Bacilli;o\_\_Bacillales;f\_\_unidentified\_Bacillales |
| 4.24076220633e-05 | 0.00% | k\_\_Bacteria;p\_\_Actinobacteria;c\_\_unidentified\_Actinobacteria;o\_\_Corynebacteriales;f\_\_Nocardiaceae |
| 2.82717480422e-05 | 0.00% | k\_\_Bacteria;p\_\_Verrucomicrobia;c\_\_Verrucomicrobiae;o\_\_Chthoniobacterales;f\_\_Chthoniobacteraceae |
| 2.82717480422e-05 | 0.00% | k\_\_Bacteria;p\_\_Proteobacteria;c\_\_Gammaproteobacteria;o\_\_Alteromonadales;f\_\_Shewanellaceae |
| 2.82717480422e-05 | 0.00% | k\_\_Bacteria;p\_\_Proteobacteria;c\_\_Alphaproteobacteria;o\_\_Rhodospirillales;f\_\_unidentified\_Rhodospirillales |
| 2.82717480422e-05 | 0.00% | k\_\_Bacteria;p\_\_Proteobacteria;c\_\_Alphaproteobacteria;o\_\_Rhizobiales;f\_\_Xanthobacteraceae |
| 2.82717480422e-05 | 0.00% | k\_\_Bacteria;p\_\_Armatimonadetes;c\_\_Fimbriimonadia;o\_\_Fimbriimonadales;f\_\_Fimbriimonadaceae |
| 1.41358740211e-05 | 0.00% | k\_\_Bacteria;p\_\_Proteobacteria;c\_\_Alphaproteobacteria;o\_\_Rhizobiales;f\_\_Beijerinckiaceae |
| 1.41358740211e-05 | 0.00% | k\_\_Bacteria;p\_\_Firmicutes;c\_\_Bacilli;o\_\_Bacillales;f\_\_Staphylococcaceae |
| 1.41358740211e-05 | 0.00% | k\_\_Bacteria;p\_\_Bacteroidetes;c\_\_Bacteroidia;o\_\_Flavobacteriales;f\_\_Flavobacteriaceae |
| 1.41358740211e-05 | 0.00% | k\_\_Bacteria;p\_\_Actinobacteria;c\_\_unidentified\_Actinobacteria;o\_\_Actinomycetales;f\_\_Actinomycetaceae |

|  |  |
| --- | --- |
|  | |
| Taxonomy Summary. Current Level: \_MC2 | |
| View Figure (.pdf)  View Legend (.pdf) |  |
|  |  |
| View Figure (.pdf)  View Legend (.pdf) |  |
|  |  |
|  |  |

|  |  |  |
| --- | --- | --- |
| Count | Pct | Taxonomy |
| 0.300966893783 | 30.10% | k\_\_Bacteria;p\_\_Bacteroidetes;c\_\_Bacteroidia;o\_\_Bacteroidales;f\_\_Muribaculaceae |
| 0.270037601425 | 27.00% | k\_\_Bacteria;p\_\_Bacteroidetes;c\_\_Bacteroidia;o\_\_Bacteroidales;f\_\_Bacteroidaceae |
| 0.108280795002 | 10.83% | k\_\_Bacteria;p\_\_Firmicutes;c\_\_Clostridia;o\_\_Clostridiales;f\_\_Lachnospiraceae |
| 0.106598625993 | 10.66% | k\_\_Bacteria;p\_\_Firmicutes;c\_\_Clostridia;o\_\_Clostridiales;f\_\_Ruminococcaceae |
| 0.0829351728817 | 8.29% | k\_\_Bacteria;p\_\_Proteobacteria;c\_\_Gammaproteobacteria;o\_\_Enterobacteriales;f\_\_Enterobacteriaceae |
| 0.0384213055893 | 3.84% | k\_\_Bacteria;p\_\_Bacteroidetes;c\_\_Bacteroidia;o\_\_Bacteroidales;f\_\_Prevotellaceae |
| 0.0250770405134 | 2.51% | k\_\_Bacteria;p\_\_Bacteroidetes;c\_\_Bacteroidia;o\_\_Bacteroidales;f\_\_Rikenellaceae |
| 0.0128636453592 | 1.29% | k\_\_Bacteria;p\_\_Proteobacteria;c\_\_Deltaproteobacteria;o\_\_Desulfovibrionales;f\_\_Desulfovibrionaceae |
| 0.0124678408866 | 1.25% | k\_\_Bacteria;p\_\_Verrucomicrobia;c\_\_Verrucomicrobiae;o\_\_Verrucomicrobiales;f\_\_Akkermansiaceae |
| 0.00941449209805 | 0.94% | k\_\_Bacteria;p\_\_Bacteroidetes;c\_\_Bacteroidia;o\_\_Bacteroidales;f\_\_Tannerellaceae |
| 0.00934381272794 | 0.93% | k\_\_Bacteria;p\_\_Firmicutes;c\_\_Bacilli;o\_\_Lactobacillales;f\_\_Enterococcaceae |
| 0.00568262135648 | 0.57% | k\_\_Bacteria;p\_\_Firmicutes;c\_\_Erysipelotrichia;o\_\_Erysipelotrichales;f\_\_Erysipelotrichaceae |
| 0.00422662633231 | 0.42% | k\_\_Bacteria;p\_\_Firmicutes;c\_\_Bacilli;o\_\_Lactobacillales;f\_\_Lactobacillaceae |
| 0.00385909360776 | 0.39% | k\_\_Bacteria;p\_\_Tenericutes;c\_\_Mollicutes;o\_\_;f\_\_ |
| 0.0034208815131 | 0.34% | k\_\_Bacteria;p\_\_Firmicutes;c\_\_Clostridia;o\_\_Clostridiales;Other |
| 0.00115914166973 | 0.12% | k\_\_Bacteria;p\_\_Firmicutes;c\_\_Clostridia;o\_\_Clostridiales;f\_\_unidentified\_Clostridiales |
| 0.00111673404767 | 0.11% | k\_\_Bacteria;p\_\_unidentified\_Bacteria;c\_\_unidentified\_Bacteria;o\_\_unidentified\_Bacteria;f\_\_unidentified\_Bacteria |
| 0.000636114330949 | 0.06% | k\_\_Bacteria;p\_\_Bacteroidetes;c\_\_Bacteroidia;o\_\_Bacteroidales;f\_\_Marinifilaceae |
| 0.000494755590738 | 0.05% | k\_\_Bacteria;p\_\_Firmicutes;c\_\_Clostridia;o\_\_Clostridiales;f\_\_Peptococcaceae |
| 0.000395804472591 | 0.04% | k\_\_Bacteria;p\_\_Actinobacteria;c\_\_unidentified\_Actinobacteria;o\_\_Bifidobacteriales;f\_\_Bifidobacteriaceae |
| 0.000339260976506 | 0.03% | k\_\_Bacteria;p\_\_Proteobacteria;c\_\_Gammaproteobacteria;o\_\_unidentified\_Gammaproteobacteria;f\_\_Burkholderiaceae |
| 0.000310989228464 | 0.03% | k\_\_Bacteria;p\_\_Melainabacteria;Other;Other;Other |
| 0.000282717480422 | 0.03% | k\_\_Bacteria;p\_\_Fusobacteria;c\_\_Fusobacteriia;o\_\_Fusobacteriales;f\_\_Fusobacteriaceae |
| 0.00025444573238 | 0.03% | k\_\_Bacteria;Other;Other;Other;Other |
| 0.000240309858359 | 0.02% | k\_\_Bacteria;p\_\_Firmicutes;Other;Other;Other |
| 0.000226173984337 | 0.02% | k\_\_Bacteria;p\_\_Firmicutes;c\_\_Negativicutes;o\_\_Selenomonadales;f\_\_Veillonellaceae |
| 0.000212038110316 | 0.02% | k\_\_Bacteria;p\_\_Firmicutes;c\_\_Bacilli;o\_\_Lactobacillales;f\_\_Streptococcaceae |
| 9.89511181476e-05 | 0.01% | k\_\_Bacteria;p\_\_Firmicutes;c\_\_Negativicutes;o\_\_Selenomonadales;f\_\_Acidaminococcaceae |
| 7.06793701055e-05 | 0.01% | k\_\_Bacteria;p\_\_Proteobacteria;c\_\_Gammaproteobacteria;o\_\_Pseudomonadales;f\_\_Pseudomonadaceae |
| 7.06793701055e-05 | 0.01% | k\_\_Bacteria;p\_\_Proteobacteria;c\_\_Gammaproteobacteria;o\_\_Pseudomonadales;f\_\_Moraxellaceae |
| 7.06793701055e-05 | 0.01% | k\_\_Bacteria;p\_\_Actinobacteria;c\_\_unidentified\_Actinobacteria;o\_\_Micrococcales;f\_\_Microbacteriaceae |
| 5.65434960844e-05 | 0.01% | k\_\_Bacteria;p\_\_Bacteroidetes;c\_\_Bacteroidia;o\_\_Flavobacteriales;f\_\_Flavobacteriaceae |
| 5.65434960844e-05 | 0.01% | k\_\_Bacteria;p\_\_Bacteroidetes;c\_\_Bacteroidia;o\_\_Bacteroidales;f\_\_Porphyromonadaceae |
| 5.65434960844e-05 | 0.01% | k\_\_Bacteria;p\_\_Bacteroidetes;c\_\_Bacteroidia;o\_\_Bacteroidales;f\_\_Barnesiellaceae |
| 5.65434960844e-05 | 0.01% | k\_\_Bacteria;p\_\_Actinobacteria;c\_\_unidentified\_Actinobacteria;o\_\_Corynebacteriales;f\_\_Nocardiaceae |
| 2.82717480422e-05 | 0.00% | k\_\_Bacteria;p\_\_unidentified\_Bacteria;c\_\_unidentified\_Bacteria;o\_\_Campylobacterales;f\_\_unidentified\_Campylobacterales |
| 2.82717480422e-05 | 0.00% | k\_\_Bacteria;p\_\_Proteobacteria;c\_\_Alphaproteobacteria;o\_\_Sphingomonadales;f\_\_Sphingomonadaceae |
| 2.82717480422e-05 | 0.00% | k\_\_Bacteria;p\_\_Firmicutes;c\_\_Bacilli;o\_\_Bacillales;f\_\_Staphylococcaceae |
| 1.41358740211e-05 | 0.00% | k\_\_Bacteria;p\_\_Proteobacteria;c\_\_Gammaproteobacteria;o\_\_Xanthomonadales;f\_\_Xanthomonadaceae |
| 1.41358740211e-05 | 0.00% | k\_\_Bacteria;p\_\_Proteobacteria;c\_\_Gammaproteobacteria;o\_\_Pasteurellales;f\_\_Pasteurellaceae |
| 1.41358740211e-05 | 0.00% | k\_\_Bacteria;p\_\_Proteobacteria;c\_\_Alphaproteobacteria;o\_\_unidentified\_Alphaproteobacteria;f\_\_unidentified\_Alphaproteobacteria |
| 1.41358740211e-05 | 0.00% | k\_\_Bacteria;p\_\_Proteobacteria;c\_\_Alphaproteobacteria;o\_\_Rhizobiales;Other |
| 1.41358740211e-05 | 0.00% | k\_\_Bacteria;p\_\_Oxyphotobacteria;c\_\_unidentified\_Oxyphotobacteria;o\_\_unidentified\_Oxyphotobacteria;f\_\_unidentified\_Oxyphotobacteria |
| 1.41358740211e-05 | 0.00% | k\_\_Bacteria;p\_\_Firmicutes;c\_\_Clostridia;o\_\_Clostridiales;f\_\_Eubacteriaceae |
| 1.41358740211e-05 | 0.00% | k\_\_Bacteria;p\_\_Actinobacteria;c\_\_Rubrobacteria;o\_\_Rubrobacterales;f\_\_unidentified\_Rubrobacterales |
| 1.41358740211e-05 | 0.00% | k\_\_Archaea;p\_\_Euryarchaeota;c\_\_Methanobacteria;o\_\_Methanobacteriales;f\_\_Methanobacteriaceae |

|  |  |
| --- | --- |
|  | |
| Taxonomy Summary. Current Level: \_MC3 | |
| View Figure (.pdf)  View Legend (.pdf) |  |
|  |  |
| View Figure (.pdf)  View Legend (.pdf) |  |
|  |  |
|  |  |

|  |  |  |
| --- | --- | --- |
| Count | Pct | Taxonomy |
| 0.340999689011 | 34.10% | k\_\_Bacteria;p\_\_Bacteroidetes;c\_\_Bacteroidia;o\_\_Bacteroidales;f\_\_Muribaculaceae |
| 0.213508241215 | 21.35% | k\_\_Bacteria;p\_\_Bacteroidetes;c\_\_Bacteroidia;o\_\_Bacteroidales;f\_\_Bacteroidaceae |
| 0.161813349919 | 16.18% | k\_\_Bacteria;p\_\_Firmicutes;c\_\_Clostridia;o\_\_Clostridiales;f\_\_Ruminococcaceae |
| 0.128226513245 | 12.82% | k\_\_Bacteria;p\_\_Firmicutes;c\_\_Clostridia;o\_\_Clostridiales;f\_\_Lachnospiraceae |
| 0.0424500296853 | 4.25% | k\_\_Bacteria;p\_\_Proteobacteria;c\_\_Gammaproteobacteria;o\_\_Enterobacteriales;f\_\_Enterobacteriaceae |
| 0.0307879336179 | 3.08% | k\_\_Bacteria;p\_\_Bacteroidetes;c\_\_Bacteroidia;o\_\_Bacteroidales;f\_\_Rikenellaceae |
| 0.0222357298352 | 2.22% | k\_\_Bacteria;p\_\_Bacteroidetes;c\_\_Bacteroidia;o\_\_Bacteroidales;f\_\_Prevotellaceae |
| 0.018786576574 | 1.88% | k\_\_Bacteria;p\_\_Proteobacteria;c\_\_Deltaproteobacteria;o\_\_Desulfovibrionales;f\_\_Desulfovibrionaceae |
| 0.00835430154646 | 0.84% | k\_\_Bacteria;p\_\_Firmicutes;c\_\_Bacilli;o\_\_Lactobacillales;f\_\_Lactobacillaceae |
| 0.00689830652229 | 0.69% | k\_\_Bacteria;p\_\_Firmicutes;c\_\_Erysipelotrichia;o\_\_Erysipelotrichales;f\_\_Erysipelotrichaceae |
| 0.00634700743547 | 0.63% | k\_\_Bacteria;p\_\_Bacteroidetes;c\_\_Bacteroidia;o\_\_Bacteroidales;f\_\_Tannerellaceae |
| 0.00414181108818 | 0.41% | k\_\_Bacteria;p\_\_Firmicutes;c\_\_Bacilli;o\_\_Lactobacillales;f\_\_Enterococcaceae |
| 0.0031522999067 | 0.32% | k\_\_Bacteria;p\_\_Tenericutes;c\_\_Mollicutes;o\_\_;f\_\_ |
| 0.00286958242628 | 0.29% | k\_\_Bacteria;p\_\_Firmicutes;c\_\_Clostridia;o\_\_Clostridiales;Other |
| 0.00162562551243 | 0.16% | k\_\_Bacteria;p\_\_Firmicutes;c\_\_Clostridia;o\_\_Clostridiales;f\_\_unidentified\_Clostridiales |
| 0.00114500579571 | 0.11% | k\_\_Bacteria;p\_\_Verrucomicrobia;c\_\_Verrucomicrobiae;o\_\_Verrucomicrobiales;f\_\_Akkermansiaceae |
| 0.000706793701055 | 0.07% | k\_\_Bacteria;Other;Other;Other;Other |
| 0.000593706708886 | 0.06% | k\_\_Bacteria;p\_\_unidentified\_Bacteria;c\_\_unidentified\_Bacteria;o\_\_unidentified\_Bacteria;f\_\_unidentified\_Bacteria |
| 0.000480619716717 | 0.05% | k\_\_Bacteria;p\_\_Firmicutes;c\_\_Clostridia;o\_\_Clostridiales;f\_\_Peptococcaceae |
| 0.000466483842696 | 0.05% | k\_\_Bacteria;p\_\_Proteobacteria;c\_\_Gammaproteobacteria;o\_\_unidentified\_Gammaproteobacteria;f\_\_Burkholderiaceae |
| 0.000452347968675 | 0.05% | k\_\_Bacteria;p\_\_Firmicutes;Other;Other;Other |
| 0.000424076220633 | 0.04% | k\_\_Bacteria;p\_\_Firmicutes;c\_\_Negativicutes;o\_\_Selenomonadales;f\_\_Veillonellaceae |
| 0.000367532724548 | 0.04% | k\_\_Bacteria;p\_\_Bacteroidetes;c\_\_Bacteroidia;o\_\_Bacteroidales;f\_\_Marinifilaceae |
| 0.000353396850527 | 0.04% | k\_\_Bacteria;p\_\_Actinobacteria;c\_\_unidentified\_Actinobacteria;o\_\_Bifidobacteriales;f\_\_Bifidobacteriaceae |
| 0.000268581606401 | 0.03% | k\_\_Bacteria;p\_\_Fusobacteria;c\_\_Fusobacteriia;o\_\_Fusobacteriales;f\_\_Fusobacteriaceae |
| 0.000240309858359 | 0.02% | k\_\_Bacteria;p\_\_Firmicutes;c\_\_Bacilli;o\_\_Lactobacillales;f\_\_Streptococcaceae |
| 0.000183766362274 | 0.02% | k\_\_Bacteria;p\_\_Proteobacteria;c\_\_Gammaproteobacteria;o\_\_Pasteurellales;f\_\_Pasteurellaceae |
| 0.000155494614232 | 0.02% | k\_\_Bacteria;p\_\_Melainabacteria;Other;Other;Other |
| 0.000141358740211 | 0.01% | k\_\_Bacteria;p\_\_Proteobacteria;c\_\_Gammaproteobacteria;o\_\_Pseudomonadales;f\_\_Pseudomonadaceae |
| 0.00012722286619 | 0.01% | k\_\_Bacteria;p\_\_Proteobacteria;c\_\_Gammaproteobacteria;o\_\_Pseudomonadales;f\_\_Moraxellaceae |
| 0.00012722286619 | 0.01% | k\_\_Bacteria;p\_\_Actinobacteria;c\_\_Thermoleophilia;o\_\_Solirubrobacterales;f\_\_ |
| 0.000113086992169 | 0.01% | k\_\_Bacteria;p\_\_unidentified\_Bacteria;c\_\_unidentified\_Bacteria;o\_\_Campylobacterales;f\_\_unidentified\_Campylobacterales |
| 0.000113086992169 | 0.01% | k\_\_Bacteria;p\_\_Firmicutes;c\_\_Clostridia;o\_\_Clostridiales;f\_\_Peptostreptococcaceae |
| 0.000113086992169 | 0.01% | k\_\_Bacteria;p\_\_Firmicutes;c\_\_Bacilli;o\_\_Bacillales;f\_\_Staphylococcaceae |
| 0.000113086992169 | 0.01% | k\_\_Bacteria;p\_\_Bacteroidetes;c\_\_Bacteroidia;o\_\_Bacteroidales;f\_\_Porphyromonadaceae |
| 8.48152441265e-05 | 0.01% | k\_\_Bacteria;p\_\_Proteobacteria;c\_\_Alphaproteobacteria;o\_\_Sphingomonadales;f\_\_Sphingomonadaceae |
| 8.48152441265e-05 | 0.01% | k\_\_Bacteria;p\_\_Proteobacteria;c\_\_Alphaproteobacteria;o\_\_Rhizobiales;f\_\_Rhizobiaceae |
| 8.48152441265e-05 | 0.01% | k\_\_Bacteria;p\_\_Firmicutes;c\_\_Negativicutes;o\_\_Selenomonadales;f\_\_Acidaminococcaceae |
| 8.48152441265e-05 | 0.01% | k\_\_Bacteria;p\_\_Bacteroidetes;c\_\_Bacteroidia;o\_\_Bacteroidales;f\_\_Barnesiellaceae |
| 7.06793701055e-05 | 0.01% | k\_\_Bacteria;p\_\_Proteobacteria;c\_\_Gammaproteobacteria;o\_\_Xanthomonadales;f\_\_Xanthomonadaceae |
| 7.06793701055e-05 | 0.01% | k\_\_Bacteria;p\_\_Firmicutes;c\_\_Clostridia;o\_\_Clostridiales;f\_\_Eubacteriaceae |
| 7.06793701055e-05 | 0.01% | k\_\_Bacteria;p\_\_Bacteroidetes;c\_\_Bacteroidia;o\_\_Chitinophagales;f\_\_Chitinophagaceae |
| 5.65434960844e-05 | 0.01% | k\_\_Bacteria;p\_\_Proteobacteria;c\_\_Alphaproteobacteria;o\_\_Rhodospirillales;f\_\_unidentified\_Rhodospirillales |
| 5.65434960844e-05 | 0.01% | k\_\_Bacteria;p\_\_Oxyphotobacteria;c\_\_unidentified\_Oxyphotobacteria;o\_\_unidentified\_Oxyphotobacteria;f\_\_unidentified\_Oxyphotobacteria |
| 5.65434960844e-05 | 0.01% | k\_\_Archaea;p\_\_Euryarchaeota;c\_\_Methanobacteria;o\_\_Methanobacteriales;f\_\_Methanobacteriaceae |
| 4.24076220633e-05 | 0.00% | k\_\_Bacteria;p\_\_Proteobacteria;c\_\_Gammaproteobacteria;o\_\_unidentified\_Gammaproteobacteria;f\_\_Neisseriaceae |
| 4.24076220633e-05 | 0.00% | k\_\_Bacteria;p\_\_Firmicutes;c\_\_Clostridia;o\_\_Clostridiales;f\_\_Christensenellaceae |
| 4.24076220633e-05 | 0.00% | k\_\_Bacteria;p\_\_Actinobacteria;c\_\_unidentified\_Actinobacteria;o\_\_Corynebacteriales;f\_\_Nocardiaceae |
| 4.24076220633e-05 | 0.00% | k\_\_Bacteria;p\_\_Actinobacteria;c\_\_Rubrobacteria;o\_\_Rubrobacterales;f\_\_unidentified\_Rubrobacterales |
| 2.82717480422e-05 | 0.00% | k\_\_Bacteria;p\_\_unidentified\_Bacteria;c\_\_unidentified\_Bacteria;o\_\_Campylobacterales;f\_\_Campylobacteraceae |
| 2.82717480422e-05 | 0.00% | k\_\_Bacteria;p\_\_Tenericutes;c\_\_Mollicutes;o\_\_Anaeroplasmatales;f\_\_Anaeroplasmataceae |
| 2.82717480422e-05 | 0.00% | k\_\_Bacteria;p\_\_Firmicutes;c\_\_Bacilli;o\_\_Lactobacillales;f\_\_Leuconostocaceae |
| 2.82717480422e-05 | 0.00% | k\_\_Bacteria;p\_\_Bacteroidetes;c\_\_Bacteroidia;o\_\_Sphingobacteriales;f\_\_ |
| 2.82717480422e-05 | 0.00% | k\_\_Bacteria;p\_\_Acidobacteria;c\_\_unidentified\_Acidobacteria;o\_\_unidentified\_Acidobacteria;f\_\_unidentified\_Acidobacteria |
| 1.41358740211e-05 | 0.00% | k\_\_Bacteria;p\_\_Proteobacteria;c\_\_Gammaproteobacteria;o\_\_Xanthomonadales;f\_\_Rhodanobacteraceae |
| 1.41358740211e-05 | 0.00% | k\_\_Bacteria;p\_\_Firmicutes;c\_\_Bacilli;o\_\_Bacillales;f\_\_unidentified\_Bacillales |
| 1.41358740211e-05 | 0.00% | k\_\_Bacteria;p\_\_Deinococcus-Thermus;c\_\_Deinococci;o\_\_Deinococcales;f\_\_Deinococcaceae |
| 1.41358740211e-05 | 0.00% | k\_\_Bacteria;p\_\_Bacteroidetes;c\_\_Bacteroidia;o\_\_Flavobacteriales;f\_\_Flavobacteriaceae |
| 1.41358740211e-05 | 0.00% | k\_\_Bacteria;p\_\_Actinobacteria;c\_\_Thermoleophilia;o\_\_Gaiellales;f\_\_ |
| 1.41358740211e-05 | 0.00% | k\_\_Bacteria;p\_\_Actinobacteria;c\_\_Coriobacteriia;o\_\_Coriobacteriales;f\_\_Atopobiaceae |

|  |  |
| --- | --- |
|  | |
| Taxonomy Summary. Current Level: \_MC4 | |
| View Figure (.pdf)  View Legend (.pdf) |  |
|  |  |
| View Figure (.pdf)  View Legend (.pdf) |  |
|  |  |
|  |  |

|  |  |  |
| --- | --- | --- |
| Count | Pct | Taxonomy |
| 0.310876141472 | 31.09% | k\_\_Bacteria;p\_\_Firmicutes;c\_\_Clostridia;o\_\_Clostridiales;f\_\_Lachnospiraceae |
| 0.256806423341 | 25.68% | k\_\_Bacteria;p\_\_Firmicutes;c\_\_Clostridia;o\_\_Clostridiales;f\_\_Ruminococcaceae |
| 0.239009357949 | 23.90% | k\_\_Bacteria;p\_\_Bacteroidetes;c\_\_Bacteroidia;o\_\_Bacteroidales;f\_\_Muribaculaceae |
| 0.103941081677 | 10.39% | k\_\_Bacteria;p\_\_Bacteroidetes;c\_\_Bacteroidia;o\_\_Bacteroidales;f\_\_Bacteroidaceae |
| 0.0282717480422 | 2.83% | k\_\_Bacteria;p\_\_Bacteroidetes;c\_\_Bacteroidia;o\_\_Bacteroidales;f\_\_Rikenellaceae |
| 0.0109694382404 | 1.10% | k\_\_Bacteria;p\_\_Proteobacteria;c\_\_Gammaproteobacteria;o\_\_Enterobacteriales;f\_\_Enterobacteriaceae |
| 0.0101071499251 | 1.01% | k\_\_Bacteria;p\_\_Bacteroidetes;c\_\_Bacteroidia;o\_\_Bacteroidales;f\_\_Prevotellaceae |
| 0.00838257329451 | 0.84% | k\_\_Bacteria;p\_\_Proteobacteria;c\_\_Deltaproteobacteria;o\_\_Desulfovibrionales;f\_\_Desulfovibrionaceae |
| 0.00554126261627 | 0.55% | k\_\_Bacteria;p\_\_Firmicutes;c\_\_Clostridia;o\_\_Clostridiales;Other |
| 0.00537163212801 | 0.54% | k\_\_Bacteria;p\_\_Bacteroidetes;c\_\_Bacteroidia;o\_\_Bacteroidales;f\_\_Tannerellaceae |
| 0.00371773486755 | 0.37% | k\_\_Bacteria;p\_\_Firmicutes;c\_\_Bacilli;o\_\_Lactobacillales;f\_\_Lactobacillaceae |
| 0.00272822368607 | 0.27% | k\_\_Bacteria;p\_\_Firmicutes;c\_\_Clostridia;o\_\_Clostridiales;f\_\_Peptococcaceae |
| 0.00248791382771 | 0.25% | k\_\_Bacteria;p\_\_Firmicutes;c\_\_Erysipelotrichia;o\_\_Erysipelotrichales;f\_\_Erysipelotrichaceae |
| 0.00188007124481 | 0.19% | k\_\_Bacteria;Other;Other;Other;Other |
| 0.00155494614232 | 0.16% | k\_\_Bacteria;p\_\_Firmicutes;c\_\_Clostridia;o\_\_Clostridiales;f\_\_unidentified\_Clostridiales |
| 0.00130050040994 | 0.13% | k\_\_Bacteria;p\_\_Verrucomicrobia;c\_\_Verrucomicrobiae;o\_\_Verrucomicrobiales;f\_\_Akkermansiaceae |
| 0.00125809278788 | 0.13% | k\_\_Bacteria;p\_\_Firmicutes;c\_\_Bacilli;o\_\_Lactobacillales;f\_\_Enterococcaceae |
| 0.000862288315287 | 0.09% | k\_\_Bacteria;p\_\_Firmicutes;Other;Other;Other |
| 0.000819880693223 | 0.08% | k\_\_Bacteria;p\_\_Tenericutes;c\_\_Mollicutes;o\_\_;f\_\_ |
| 0.000607842582907 | 0.06% | k\_\_Bacteria;p\_\_Firmicutes;c\_\_Negativicutes;o\_\_Selenomonadales;f\_\_Veillonellaceae |
| 0.000579570834865 | 0.06% | k\_\_Bacteria;p\_\_Proteobacteria;c\_\_Gammaproteobacteria;o\_\_unidentified\_Gammaproteobacteria;f\_\_Burkholderiaceae |
| 0.000367532724548 | 0.04% | k\_\_Bacteria;p\_\_unidentified\_Bacteria;c\_\_unidentified\_Bacteria;o\_\_Campylobacterales;f\_\_unidentified\_Campylobacterales |
| 0.000353396850527 | 0.04% | k\_\_Bacteria;p\_\_Bacteroidetes;c\_\_Bacteroidia;o\_\_Bacteroidales;f\_\_Marinifilaceae |
| 0.000325125102485 | 0.03% | k\_\_Bacteria;p\_\_Melainabacteria;Other;Other;Other |
| 0.000310989228464 | 0.03% | k\_\_Bacteria;p\_\_Fusobacteria;c\_\_Fusobacteriia;o\_\_Fusobacteriales;f\_\_Fusobacteriaceae |
| 0.000212038110316 | 0.02% | k\_\_Bacteria;p\_\_Actinobacteria;c\_\_unidentified\_Actinobacteria;o\_\_Bifidobacteriales;f\_\_Bifidobacteriaceae |
| 0.000197902236295 | 0.02% | k\_\_Bacteria;p\_\_unidentified\_Bacteria;c\_\_unidentified\_Bacteria;o\_\_unidentified\_Bacteria;f\_\_unidentified\_Bacteria |
| 0.000169630488253 | 0.02% | k\_\_Bacteria;p\_\_Firmicutes;c\_\_Negativicutes;o\_\_Selenomonadales;f\_\_Acidaminococcaceae |
| 0.000155494614232 | 0.02% | k\_\_Bacteria;p\_\_Firmicutes;c\_\_Clostridia;o\_\_Clostridiales;f\_\_Peptostreptococcaceae |
| 0.00012722286619 | 0.01% | k\_\_Bacteria;p\_\_Bacteroidetes;c\_\_Bacteroidia;o\_\_Bacteroidales;f\_\_Porphyromonadaceae |
| 8.48152441265e-05 | 0.01% | k\_\_Bacteria;p\_\_Actinobacteria;c\_\_unidentified\_Actinobacteria;o\_\_Actinomycetales;f\_\_Actinomycetaceae |
| 7.06793701055e-05 | 0.01% | k\_\_Bacteria;p\_\_Proteobacteria;c\_\_Gammaproteobacteria;o\_\_Pseudomonadales;f\_\_Moraxellaceae |
| 5.65434960844e-05 | 0.01% | k\_\_Bacteria;p\_\_Proteobacteria;c\_\_Alphaproteobacteria;o\_\_Rhodospirillales;f\_\_unidentified\_Rhodospirillales |
| 5.65434960844e-05 | 0.01% | k\_\_Bacteria;p\_\_Bacteroidetes;c\_\_Bacteroidia;o\_\_Bacteroidales;f\_\_Barnesiellaceae |
| 4.24076220633e-05 | 0.00% | k\_\_Bacteria;p\_\_Proteobacteria;c\_\_Gammaproteobacteria;o\_\_Xanthomonadales;f\_\_Xanthomonadaceae |
| 4.24076220633e-05 | 0.00% | k\_\_Bacteria;p\_\_Proteobacteria;c\_\_Gammaproteobacteria;o\_\_Pseudomonadales;f\_\_Pseudomonadaceae |
| 4.24076220633e-05 | 0.00% | k\_\_Bacteria;p\_\_Proteobacteria;c\_\_Gammaproteobacteria;o\_\_Pasteurellales;f\_\_Pasteurellaceae |
| 4.24076220633e-05 | 0.00% | k\_\_Bacteria;p\_\_Proteobacteria;c\_\_Alphaproteobacteria;o\_\_Sphingomonadales;f\_\_Sphingomonadaceae |
| 4.24076220633e-05 | 0.00% | k\_\_Bacteria;p\_\_Oxyphotobacteria;c\_\_unidentified\_Oxyphotobacteria;o\_\_unidentified\_Oxyphotobacteria;f\_\_unidentified\_Oxyphotobacteria |
| 4.24076220633e-05 | 0.00% | k\_\_Bacteria;p\_\_Firmicutes;c\_\_Clostridia;o\_\_Clostridiales;f\_\_Eubacteriaceae |
| 4.24076220633e-05 | 0.00% | k\_\_Bacteria;p\_\_Firmicutes;c\_\_Bacilli;o\_\_Bacillales;f\_\_unidentified\_Bacillales |
| 2.82717480422e-05 | 0.00% | k\_\_Bacteria;p\_\_Proteobacteria;c\_\_Alphaproteobacteria;o\_\_Rhizobiales;f\_\_Rhizobiaceae |
| 2.82717480422e-05 | 0.00% | k\_\_Bacteria;p\_\_Firmicutes;c\_\_Clostridia;o\_\_Clostridiales;f\_\_Christensenellaceae |
| 2.82717480422e-05 | 0.00% | k\_\_Bacteria;p\_\_Firmicutes;c\_\_Bacilli;o\_\_Lactobacillales;f\_\_Streptococcaceae |
| 2.82717480422e-05 | 0.00% | k\_\_Bacteria;p\_\_Actinobacteria;c\_\_unidentified\_Actinobacteria;o\_\_Corynebacteriales;f\_\_Corynebacteriaceae |
| 1.41358740211e-05 | 0.00% | k\_\_Bacteria;p\_\_Firmicutes;c\_\_Bacilli;o\_\_Bacillales;f\_\_Staphylococcaceae |
| 1.41358740211e-05 | 0.00% | k\_\_Bacteria;p\_\_Actinobacteria;c\_\_unidentified\_Actinobacteria;o\_\_Corynebacteriales;f\_\_Nocardiaceae |

|  |  |
| --- | --- |
|  | |
| Taxonomy Summary. Current Level: \_MC5 | |
| View Figure (.pdf)  View Legend (.pdf) |  |
|  |  |
| View Figure (.pdf)  View Legend (.pdf) |  |
|  |  |
|  |  |

|  |  |  |
| --- | --- | --- |
| Count | Pct | Taxonomy |
| 0.262008424981 | 26.20% | k\_\_Bacteria;p\_\_Bacteroidetes;c\_\_Bacteroidia;o\_\_Bacteroidales;f\_\_Muribaculaceae |
| 0.200022617398 | 20.00% | k\_\_Bacteria;p\_\_Firmicutes;c\_\_Clostridia;o\_\_Clostridiales;f\_\_Lachnospiraceae |
| 0.195018517995 | 19.50% | k\_\_Bacteria;p\_\_Bacteroidetes;c\_\_Bacteroidia;o\_\_Bacteroidales;f\_\_Bacteroidaceae |
| 0.18346950892 | 18.35% | k\_\_Bacteria;p\_\_Firmicutes;c\_\_Clostridia;o\_\_Clostridiales;f\_\_Ruminococcaceae |
| 0.0478923411835 | 4.79% | k\_\_Bacteria;p\_\_Proteobacteria;c\_\_Gammaproteobacteria;o\_\_Enterobacteriales;f\_\_Enterobacteriaceae |
| 0.0286534166408 | 2.87% | k\_\_Bacteria;p\_\_Bacteroidetes;c\_\_Bacteroidia;o\_\_Bacteroidales;f\_\_Prevotellaceae |
| 0.0193944191569 | 1.94% | k\_\_Bacteria;p\_\_Bacteroidetes;c\_\_Bacteroidia;o\_\_Bacteroidales;f\_\_Rikenellaceae |
| 0.0141782816432 | 1.42% | k\_\_Bacteria;p\_\_Verrucomicrobia;c\_\_Verrucomicrobiae;o\_\_Verrucomicrobiales;f\_\_Akkermansiaceae |
| 0.0101919651692 | 1.02% | k\_\_Bacteria;p\_\_Proteobacteria;c\_\_Deltaproteobacteria;o\_\_Desulfovibrionales;f\_\_Desulfovibrionaceae |
| 0.00736479036499 | 0.74% | k\_\_Bacteria;p\_\_Bacteroidetes;c\_\_Bacteroidia;o\_\_Bacteroidales;f\_\_Tannerellaceae |
| 0.00718102400271 | 0.72% | k\_\_Bacteria;p\_\_Firmicutes;c\_\_Clostridia;o\_\_Clostridiales;Other |
| 0.00460829493088 | 0.46% | k\_\_Bacteria;p\_\_Tenericutes;c\_\_Mollicutes;o\_\_;f\_\_ |
| 0.00460829493088 | 0.46% | k\_\_Bacteria;p\_\_Firmicutes;c\_\_Bacilli;o\_\_Lactobacillales;f\_\_Lactobacillaceae |
| 0.00302507704051 | 0.30% | k\_\_Bacteria;p\_\_Firmicutes;c\_\_Bacilli;o\_\_Lactobacillales;f\_\_Enterococcaceae |
| 0.00221933222131 | 0.22% | k\_\_Bacteria;p\_\_Firmicutes;c\_\_Erysipelotrichia;o\_\_Erysipelotrichales;f\_\_Erysipelotrichaceae |
| 0.00178112012666 | 0.18% | k\_\_Bacteria;p\_\_Firmicutes;c\_\_Clostridia;o\_\_Clostridiales;f\_\_unidentified\_Clostridiales |
| 0.00144185915015 | 0.14% | k\_\_Bacteria;p\_\_Melainabacteria;Other;Other;Other |
| 0.0010743264256 | 0.11% | k\_\_Bacteria;Other;Other;Other;Other |
| 0.00101778292952 | 0.10% | k\_\_Bacteria;p\_\_Bacteroidetes;c\_\_Bacteroidia;o\_\_Bacteroidales;f\_\_Marinifilaceae |
| 0.000819880693223 | 0.08% | k\_\_Bacteria;p\_\_unidentified\_Bacteria;c\_\_unidentified\_Bacteria;o\_\_unidentified\_Bacteria;f\_\_unidentified\_Bacteria |
| 0.00077747307116 | 0.08% | k\_\_Bacteria;p\_\_Firmicutes;c\_\_Clostridia;o\_\_Clostridiales;f\_\_Peptococcaceae |
| 0.000452347968675 | 0.05% | k\_\_Bacteria;p\_\_Firmicutes;c\_\_Negativicutes;o\_\_Selenomonadales;f\_\_Veillonellaceae |
| 0.000409940346612 | 0.04% | k\_\_Bacteria;p\_\_Proteobacteria;c\_\_Gammaproteobacteria;o\_\_unidentified\_Gammaproteobacteria;f\_\_Burkholderiaceae |
| 0.000409940346612 | 0.04% | k\_\_Bacteria;p\_\_Actinobacteria;c\_\_unidentified\_Actinobacteria;o\_\_Corynebacteriales;f\_\_Nocardiaceae |
| 0.000339260976506 | 0.03% | k\_\_Bacteria;p\_\_Fusobacteria;c\_\_Fusobacteriia;o\_\_Fusobacteriales;f\_\_Fusobacteriaceae |
| 0.000310989228464 | 0.03% | k\_\_Bacteria;p\_\_Firmicutes;Other;Other;Other |
| 0.00025444573238 | 0.03% | k\_\_Bacteria;p\_\_Actinobacteria;c\_\_unidentified\_Actinobacteria;o\_\_Bifidobacteriales;f\_\_Bifidobacteriaceae |
| 0.000155494614232 | 0.02% | k\_\_Bacteria;p\_\_Firmicutes;c\_\_Negativicutes;o\_\_Selenomonadales;f\_\_Acidaminococcaceae |
| 0.000141358740211 | 0.01% | k\_\_Bacteria;p\_\_Proteobacteria;c\_\_Gammaproteobacteria;o\_\_Pseudomonadales;f\_\_Moraxellaceae |
| 0.00012722286619 | 0.01% | k\_\_Bacteria;p\_\_Proteobacteria;c\_\_Gammaproteobacteria;o\_\_Pasteurellales;f\_\_Pasteurellaceae |
| 9.89511181476e-05 | 0.01% | k\_\_Bacteria;p\_\_Proteobacteria;c\_\_Gammaproteobacteria;o\_\_Pseudomonadales;f\_\_Pseudomonadaceae |
| 9.89511181476e-05 | 0.01% | k\_\_Bacteria;p\_\_Firmicutes;c\_\_Bacilli;o\_\_Lactobacillales;f\_\_Streptococcaceae |
| 9.89511181476e-05 | 0.01% | k\_\_Bacteria;p\_\_Bacteroidetes;c\_\_Bacteroidia;o\_\_Bacteroidales;f\_\_Barnesiellaceae |
| 8.48152441265e-05 | 0.01% | k\_\_Bacteria;p\_\_Firmicutes;c\_\_Clostridia;o\_\_Clostridiales;f\_\_Peptostreptococcaceae |
| 8.48152441265e-05 | 0.01% | k\_\_Bacteria;p\_\_Bacteroidetes;c\_\_Bacteroidia;o\_\_Bacteroidales;f\_\_Porphyromonadaceae |
| 4.24076220633e-05 | 0.00% | k\_\_Bacteria;p\_\_unidentified\_Bacteria;c\_\_unidentified\_Bacteria;o\_\_Campylobacterales;f\_\_unidentified\_Campylobacterales |
| 2.82717480422e-05 | 0.00% | k\_\_Bacteria;p\_\_Proteobacteria;c\_\_Alphaproteobacteria;o\_\_Caulobacterales;f\_\_Caulobacteraceae |
| 2.82717480422e-05 | 0.00% | k\_\_Bacteria;p\_\_Acidobacteria;c\_\_unidentified\_Acidobacteria;o\_\_unidentified\_Acidobacteria;f\_\_unidentified\_Acidobacteria |
| 2.82717480422e-05 | 0.00% | k\_\_Bacteria;p\_\_Acidobacteria;c\_\_Holophagae;o\_\_;f\_\_ |
| 1.41358740211e-05 | 0.00% | k\_\_Bacteria;p\_\_Proteobacteria;c\_\_Gammaproteobacteria;o\_\_Xanthomonadales;f\_\_Xanthomonadaceae |
| 1.41358740211e-05 | 0.00% | k\_\_Bacteria;p\_\_Proteobacteria;c\_\_Alphaproteobacteria;o\_\_Rhodospirillales;f\_\_unidentified\_Rhodospirillales |
| 1.41358740211e-05 | 0.00% | k\_\_Bacteria;p\_\_Proteobacteria;c\_\_Alphaproteobacteria;o\_\_Rhizobiales;f\_\_Rhizobiaceae |
| 1.41358740211e-05 | 0.00% | k\_\_Bacteria;p\_\_Deinococcus-Thermus;c\_\_Deinococci;o\_\_Deinococcales;f\_\_Deinococcaceae |

|  |  |
| --- | --- |
|  | |
| Taxonomy Summary. Current Level: | |
| View Figure (.pdf)  View Legend (.pdf) |  |
|  |  |
| View Figure (.pdf)  View Legend (.pdf) |  |
|  |  |
|  |  |

|  |  |  |
| --- | --- | --- |
| Count | Pct | Taxonomy |
| 2.97736846569 | 29.77% | k\_\_Bacteria;p\_\_Bacteroidetes;c\_\_Bacteroidia;o\_\_Bacteroidales;f\_\_Muribaculaceae;g\_\_ |
| 1.77482966272 | 17.75% | k\_\_Bacteria;p\_\_Bacteroidetes;c\_\_Bacteroidia;o\_\_Bacteroidales;f\_\_Bacteroidaceae;g\_\_Bacteroides |
| 1.40578439965 | 14.06% | k\_\_Bacteria;p\_\_Firmicutes;c\_\_Clostridia;o\_\_Clostridiales;f\_\_Lachnospiraceae;Other |
| 0.632410731956 | 6.32% | k\_\_Bacteria;p\_\_Firmicutes;c\_\_Clostridia;o\_\_Clostridiales;f\_\_Ruminococcaceae;Other |
| 0.50267168019 | 5.03% | k\_\_Bacteria;p\_\_Firmicutes;c\_\_Clostridia;o\_\_Clostridiales;f\_\_Lachnospiraceae;g\_\_unidentified\_Lachnospiraceae |
| 0.404116366515 | 4.04% | k\_\_Bacteria;p\_\_Firmicutes;c\_\_Clostridia;o\_\_Clostridiales;f\_\_Ruminococcaceae;g\_\_unidentified\_Ruminococcaceae |
| 0.372437872834 | 3.72% | k\_\_Bacteria;p\_\_Proteobacteria;c\_\_Gammaproteobacteria;o\_\_Enterobacteriales;f\_\_Enterobacteriaceae;g\_\_unidentified\_Enterobacteriaceae |
| 0.1945803059 | 1.95% | k\_\_Bacteria;p\_\_Bacteroidetes;c\_\_Bacteroidia;o\_\_Bacteroidales;f\_\_Rikenellaceae;g\_\_Alistipes |
| 0.158491419524 | 1.58% | k\_\_Bacteria;p\_\_Bacteroidetes;c\_\_Bacteroidia;o\_\_Bacteroidales;f\_\_Prevotellaceae;g\_\_Alloprevotella |
| 0.135718526477 | 1.36% | k\_\_Bacteria;p\_\_Verrucomicrobia;c\_\_Verrucomicrobiae;o\_\_Verrucomicrobiales;f\_\_Akkermansiaceae;g\_\_Akkermansia |
| 0.132792400554 | 1.33% | k\_\_Bacteria;p\_\_Proteobacteria;c\_\_Gammaproteobacteria;o\_\_Enterobacteriales;f\_\_Enterobacteriaceae;Other |
| 0.125130756835 | 1.25% | k\_\_Bacteria;p\_\_Firmicutes;c\_\_Clostridia;o\_\_Clostridiales;f\_\_Lachnospiraceae;g\_\_Lachnoclostridium |
| 0.115659721241 | 1.16% | k\_\_Bacteria;p\_\_Firmicutes;c\_\_Clostridia;o\_\_Clostridiales;f\_\_Lachnospiraceae;g\_\_Roseburia |
| 0.114260269712 | 1.14% | k\_\_Bacteria;p\_\_Firmicutes;c\_\_Clostridia;o\_\_Clostridiales;f\_\_Lachnospiraceae;g\_\_Blautia |
| 0.110726301207 | 1.11% | k\_\_Bacteria;p\_\_Proteobacteria;c\_\_Deltaproteobacteria;o\_\_Desulfovibrionales;f\_\_Desulfovibrionaceae;g\_\_Desulfovibrio |
| 0.0742840179809 | 0.74% | k\_\_Bacteria;p\_\_Bacteroidetes;c\_\_Bacteroidia;o\_\_Bacteroidales;f\_\_Muribaculaceae;g\_\_Muribaculum |
| 0.0670464504821 | 0.67% | k\_\_Bacteria;p\_\_Firmicutes;c\_\_Clostridia;o\_\_Clostridiales;f\_\_Ruminococcaceae;g\_\_Oscillibacter |
| 0.0644171779141 | 0.64% | k\_\_Bacteria;p\_\_Proteobacteria;c\_\_Gammaproteobacteria;o\_\_Enterobacteriales;f\_\_Enterobacteriaceae;g\_\_Proteus |
| 0.0599502417235 | 0.60% | k\_\_Bacteria;p\_\_Bacteroidetes;c\_\_Bacteroidia;o\_\_Bacteroidales;f\_\_Tannerellaceae;g\_\_Parabacteroides |
| 0.0537445930282 | 0.54% | k\_\_Bacteria;p\_\_Firmicutes;c\_\_Bacilli;o\_\_Lactobacillales;f\_\_Lactobacillaceae;g\_\_Lactobacillus |
| 0.0475248084589 | 0.48% | k\_\_Bacteria;p\_\_Firmicutes;c\_\_Clostridia;o\_\_Clostridiales;f\_\_Lachnospiraceae;g\_\_ |
| 0.04693110175 | 0.47% | k\_\_Bacteria;p\_\_Firmicutes;c\_\_Clostridia;o\_\_Clostridiales;f\_\_Ruminococcaceae;g\_\_Ruminiclostridium |
| 0.0450510305052 | 0.45% | k\_\_Bacteria;p\_\_Tenericutes;c\_\_Mollicutes;o\_\_;f\_\_;g\_\_ |
| 0.0445280031664 | 0.45% | k\_\_Bacteria;p\_\_Firmicutes;c\_\_Bacilli;o\_\_Lactobacillales;f\_\_Enterococcaceae;g\_\_Enterococcus |
| 0.0428741059059 | 0.43% | k\_\_Bacteria;p\_\_Firmicutes;c\_\_Clostridia;o\_\_Clostridiales;f\_\_Ruminococcaceae;g\_\_Butyricicoccus |
| 0.0377851912584 | 0.38% | k\_\_Bacteria;p\_\_Firmicutes;c\_\_Clostridia;o\_\_Clostridiales;Other;Other |
| 0.0284979220265 | 0.28% | k\_\_Bacteria;p\_\_Bacteroidetes;c\_\_Bacteroidia;o\_\_Bacteroidales;f\_\_Prevotellaceae;Other |
| 0.0283848350343 | 0.28% | k\_\_Bacteria;p\_\_Firmicutes;c\_\_Clostridia;o\_\_Clostridiales;f\_\_Ruminococcaceae;g\_\_Anaerotruncus |
| 0.0260241440728 | 0.26% | k\_\_Bacteria;p\_\_Firmicutes;c\_\_Clostridia;o\_\_Clostridiales;f\_\_Ruminococcaceae;g\_\_Intestinimonas |
| 0.0201718922281 | 0.20% | k\_\_Bacteria;p\_\_Firmicutes;c\_\_Erysipelotrichia;o\_\_Erysipelotrichales;f\_\_Erysipelotrichaceae;Other |
| 0.0184614514715 | 0.18% | k\_\_Bacteria;p\_\_Firmicutes;c\_\_Erysipelotrichia;o\_\_Erysipelotrichales;f\_\_Erysipelotrichaceae;g\_\_Dubosiella |
| 0.0126798789969 | 0.13% | k\_\_Bacteria;p\_\_Firmicutes;c\_\_Clostridia;o\_\_Clostridiales;f\_\_Lachnospiraceae;g\_\_Acetatifactor |
| 0.00886319301122 | 0.09% | k\_\_Bacteria;p\_\_Firmicutes;c\_\_Clostridia;o\_\_Clostridiales;f\_\_unidentified\_Clostridiales;g\_\_unidentified\_Clostridiales |
| 0.00845325266461 | 0.08% | k\_\_Bacteria;Other;Other;Other;Other;Other |
| 0.00771818721551 | 0.08% | k\_\_Bacteria;p\_\_Firmicutes;c\_\_Clostridia;o\_\_Clostridiales;f\_\_Ruminococcaceae;g\_\_Faecalibacterium |
| 0.00732238274292 | 0.07% | k\_\_Bacteria;p\_\_Firmicutes;c\_\_Clostridia;o\_\_Clostridiales;f\_\_Peptococcaceae;g\_\_ |
| 0.00687003477425 | 0.07% | k\_\_Bacteria;p\_\_Bacteroidetes;c\_\_Bacteroidia;o\_\_Bacteroidales;f\_\_Marinifilaceae;g\_\_Butyricimonas |
| 0.00602188233298 | 0.06% | k\_\_Bacteria;p\_\_Firmicutes;c\_\_Clostridia;o\_\_Clostridiales;f\_\_Ruminococcaceae;g\_\_Harryflintia |
| 0.00487687653728 | 0.05% | k\_\_Bacteria;p\_\_unidentified\_Bacteria;c\_\_unidentified\_Bacteria;o\_\_unidentified\_Bacteria;f\_\_unidentified\_Bacteria;g\_\_Candidatus\_Saccharimonas |
| 0.00465070255294 | 0.05% | k\_\_Bacteria;p\_\_Bacteroidetes;c\_\_Bacteroidia;o\_\_Bacteroidales;f\_\_Prevotellaceae;g\_\_unidentified\_Prevotellaceae |
| 0.00463656667892 | 0.05% | k\_\_Bacteria;p\_\_Melainabacteria;Other;Other;Other;Other |
| 0.00453761556077 | 0.05% | k\_\_Bacteria;p\_\_Firmicutes;c\_\_Clostridia;o\_\_Clostridiales;f\_\_Lachnospiraceae;g\_\_Tyzzerella |
| 0.00400045234797 | 0.04% | k\_\_Bacteria;p\_\_Oxyphotobacteria;c\_\_unidentified\_Oxyphotobacteria;o\_\_unidentified\_Oxyphotobacteria;f\_\_unidentified\_Oxyphotobacteria;g\_\_unidentified\_Oxyphotobacteria |
| 0.00347742500919 | 0.03% | k\_\_Bacteria;p\_\_Firmicutes;Other;Other;Other;Other |
| 0.00305334878856 | 0.03% | k\_\_Bacteria;p\_\_Firmicutes;c\_\_Clostridia;o\_\_Clostridiales;f\_\_unidentified\_Clostridiales;g\_\_Anaerovorax |
| 0.00291199004835 | 0.03% | k\_\_Bacteria;p\_\_Fusobacteria;c\_\_Fusobacteriia;o\_\_Fusobacteriales;f\_\_Fusobacteriaceae;g\_\_Fusobacterium |
| 0.0028130389302 | 0.03% | k\_\_Bacteria;p\_\_Firmicutes;c\_\_Clostridia;o\_\_Clostridiales;f\_\_Ruminococcaceae;g\_\_Subdoligranulum |
| 0.00271408781205 | 0.03% | k\_\_Bacteria;p\_\_Firmicutes;c\_\_Clostridia;o\_\_Clostridiales;f\_\_Ruminococcaceae;g\_\_Caproiciproducens |
| 0.00269995193803 | 0.03% | k\_\_Bacteria;p\_\_Actinobacteria;c\_\_unidentified\_Actinobacteria;o\_\_Bifidobacteriales;f\_\_Bifidobacteriaceae;g\_\_Bifidobacterium |
| 0.00247377795369 | 0.02% | k\_\_Bacteria;p\_\_Firmicutes;c\_\_Clostridia;o\_\_Clostridiales;f\_\_unidentified\_Clostridiales;Other |
| 0.00238896270956 | 0.02% | k\_\_Bacteria;p\_\_Firmicutes;c\_\_Negativicutes;o\_\_Selenomonadales;f\_\_Veillonellaceae;g\_\_Dialister |
| 0.00209210935512 | 0.02% | k\_\_Bacteria;p\_\_Firmicutes;c\_\_Clostridia;o\_\_Clostridiales;f\_\_Lachnospiraceae;g\_\_Fusicatenibacter |
| 0.00190834299285 | 0.02% | k\_\_Bacteria;p\_\_Firmicutes;c\_\_Clostridia;o\_\_Clostridiales;f\_\_Lachnospiraceae;g\_\_Marvinbryantia |
| 0.00145599502417 | 0.01% | k\_\_Bacteria;p\_\_Proteobacteria;c\_\_Gammaproteobacteria;o\_\_unidentified\_Gammaproteobacteria;f\_\_Burkholderiaceae;g\_\_Sutterella |
| 0.00118741341777 | 0.01% | k\_\_Bacteria;p\_\_Firmicutes;c\_\_Bacilli;o\_\_Lactobacillales;f\_\_Streptococcaceae;g\_\_Streptococcus |
| 0.00114500579571 | 0.01% | k\_\_Bacteria;p\_\_Proteobacteria;c\_\_Gammaproteobacteria;o\_\_Pseudomonadales;f\_\_Moraxellaceae;g\_\_Acinetobacter |
| 0.00113086992169 | 0.01% | k\_\_Bacteria;p\_\_unidentified\_Bacteria;c\_\_unidentified\_Bacteria;o\_\_Campylobacterales;f\_\_unidentified\_Campylobacterales;g\_\_Sulfurospirillum |
| 0.00111673404767 | 0.01% | k\_\_Bacteria;p\_\_Firmicutes;c\_\_Clostridia;o\_\_Clostridiales;f\_\_Peptococcaceae;g\_\_Peptococcus |
| 0.00110259817365 | 0.01% | k\_\_Bacteria;p\_\_Proteobacteria;c\_\_Gammaproteobacteria;o\_\_unidentified\_Gammaproteobacteria;f\_\_Burkholderiaceae;g\_\_Parasutterella |
| 0.0010743264256 | 0.01% | k\_\_Bacteria;p\_\_Firmicutes;c\_\_Clostridia;o\_\_Clostridiales;f\_\_Ruminococcaceae;g\_\_Flavonifractor |
| 0.00106019055158 | 0.01% | k\_\_Bacteria;p\_\_Bacteroidetes;c\_\_Bacteroidia;o\_\_Bacteroidales;f\_\_Marinifilaceae;g\_\_Odoribacter |
| 0.00106019055158 | 0.01% | k\_\_Bacteria;p\_\_Firmicutes;c\_\_Clostridia;o\_\_Clostridiales;f\_\_Lachnospiraceae;g\_\_Anaerostipes |
| 0.000961239433435 | 0.01% | k\_\_Bacteria;p\_\_Bacteroidetes;c\_\_Bacteroidia;o\_\_Bacteroidales;f\_\_Prevotellaceae;g\_\_Prevotella |
| 0.000918831811372 | 0.01% | k\_\_Bacteria;p\_\_Proteobacteria;c\_\_Gammaproteobacteria;o\_\_unidentified\_Gammaproteobacteria;f\_\_Burkholderiaceae;g\_\_Diaphorobacter |
| 0.000918831811372 | 0.01% | k\_\_Bacteria;p\_\_Firmicutes;c\_\_Erysipelotrichia;o\_\_Erysipelotrichales;f\_\_Erysipelotrichaceae;g\_\_unidentified\_Erysipelotrichaceae |
| 0.000918831811371 | 0.01% | k\_\_Bacteria;p\_\_Proteobacteria;c\_\_Gammaproteobacteria;o\_\_Pseudomonadales;f\_\_Pseudomonadaceae;g\_\_Pseudomonas |
| 0.00089056006333 | 0.01% | k\_\_Bacteria;p\_\_Bacteroidetes;c\_\_Bacteroidia;o\_\_Bacteroidales;f\_\_Porphyromonadaceae;g\_\_Porphyromonas |
| 0.000791608945181 | 0.01% | k\_\_Bacteria;p\_\_Firmicutes;c\_\_Negativicutes;o\_\_Selenomonadales;f\_\_Acidaminococcaceae;g\_\_Phascolarctobacterium |
| 0.000749201323118 | 0.01% | k\_\_Bacteria;p\_\_Proteobacteria;c\_\_Gammaproteobacteria;o\_\_Pasteurellales;f\_\_Pasteurellaceae;Other |
| 0.000720929575076 | 0.01% | k\_\_Bacteria;p\_\_Actinobacteria;c\_\_unidentified\_Actinobacteria;o\_\_Corynebacteriales;f\_\_Nocardiaceae;g\_\_Rhodococcus |
| 0.000650250204971 | 0.01% | k\_\_Bacteria;p\_\_Proteobacteria;c\_\_Gammaproteobacteria;o\_\_unidentified\_Gammaproteobacteria;f\_\_Burkholderiaceae;g\_\_Delftia |
| 0.000593706708886 | 0.01% | k\_\_Bacteria;p\_\_Firmicutes;c\_\_Negativicutes;o\_\_Selenomonadales;f\_\_Veillonellaceae;g\_\_Veillonella |
| 0.000593706708886 | 0.01% | k\_\_Bacteria;p\_\_Firmicutes;c\_\_Clostridia;o\_\_Clostridiales;f\_\_Lachnospiraceae;g\_\_Lachnospira |
| 0.000579570834865 | 0.01% | k\_\_Bacteria;p\_\_Firmicutes;c\_\_Clostridia;o\_\_Clostridiales;f\_\_Peptostreptococcaceae;g\_\_Romboutsia |
| 0.000523027338781 | 0.01% | k\_\_Bacteria;p\_\_Bacteroidetes;c\_\_Bacteroidia;o\_\_Bacteroidales;f\_\_Barnesiellaceae;g\_\_Barnesiella |
| 0.000494755590738 | 0.00% | k\_\_Bacteria;p\_\_Proteobacteria;c\_\_Deltaproteobacteria;o\_\_Desulfovibrionales;f\_\_Desulfovibrionaceae;g\_\_ |
| 0.000395804472591 | 0.00% | k\_\_Bacteria;p\_\_Firmicutes;c\_\_Negativicutes;o\_\_Selenomonadales;f\_\_Veillonellaceae;g\_\_Megamonas |
| 0.000367532724549 | 0.00% | k\_\_Bacteria;p\_\_Proteobacteria;c\_\_Gammaproteobacteria;o\_\_unidentified\_Gammaproteobacteria;f\_\_Burkholderiaceae;Other |
| 0.000353396850527 | 0.00% | k\_\_Bacteria;p\_\_Firmicutes;c\_\_Clostridia;o\_\_Clostridiales;f\_\_Peptostreptococcaceae;g\_\_Clostridioides |
| 0.000339260976506 | 0.00% | k\_\_Bacteria;p\_\_Proteobacteria;c\_\_Gammaproteobacteria;o\_\_Xanthomonadales;f\_\_Xanthomonadaceae;g\_\_Stenotrophomonas |
| 0.000339260976506 | 0.00% | k\_\_Bacteria;p\_\_Firmicutes;c\_\_Negativicutes;o\_\_Selenomonadales;f\_\_Veillonellaceae;g\_\_Megasphaera |
| 0.000325125102485 | 0.00% | k\_\_Bacteria;p\_\_Proteobacteria;c\_\_Alphaproteobacteria;o\_\_Sphingomonadales;f\_\_Sphingomonadaceae;g\_\_Sphingomonas |
| 0.000325125102485 | 0.00% | k\_\_Bacteria;p\_\_Proteobacteria;c\_\_Deltaproteobacteria;o\_\_Desulfovibrionales;f\_\_Desulfovibrionaceae;g\_\_Bilophila |
| 0.000296853354443 | 0.00% | k\_\_Bacteria;p\_\_Firmicutes;c\_\_Erysipelotrichia;o\_\_Erysipelotrichales;f\_\_Erysipelotrichaceae;g\_\_Erysipelatoclostridium |
| 0.000296853354443 | 0.00% | k\_\_Bacteria;p\_\_Bacteroidetes;c\_\_Bacteroidia;o\_\_Bacteroidales;f\_\_Barnesiellaceae;g\_\_Coprobacter |
| 0.000268581606401 | 0.00% | k\_\_Bacteria;p\_\_Firmicutes;c\_\_Clostridia;o\_\_Clostridiales;f\_\_Eubacteriaceae;g\_\_Anaerofustis |
| 0.00025444573238 | 0.00% | k\_\_Bacteria;p\_\_Firmicutes;c\_\_Erysipelotrichia;o\_\_Erysipelotrichales;f\_\_Erysipelotrichaceae;g\_\_Faecalitalea |
| 0.000254445732379 | 0.00% | k\_\_Bacteria;p\_\_Proteobacteria;c\_\_Gammaproteobacteria;o\_\_Enterobacteriales;f\_\_Enterobacteriaceae;g\_\_Morganella |
| 0.000240309858359 | 0.00% | k\_\_Bacteria;p\_\_Proteobacteria;c\_\_Alphaproteobacteria;o\_\_Rhodospirillales;f\_\_unidentified\_Rhodospirillales;g\_\_Telmatospirillum |
| 0.000226173984337 | 0.00% | k\_\_Bacteria;p\_\_Firmicutes;c\_\_Negativicutes;o\_\_Selenomonadales;f\_\_Acidaminococcaceae;g\_\_Acidaminococcus |
| 0.000212038110317 | 0.00% | k\_\_Bacteria;p\_\_Firmicutes;c\_\_Bacilli;o\_\_Bacillales;f\_\_Staphylococcaceae;g\_\_Staphylococcus |
| 0.000212038110317 | 0.00% | k\_\_Bacteria;p\_\_Proteobacteria;c\_\_Gammaproteobacteria;o\_\_unidentified\_Gammaproteobacteria;f\_\_Burkholderiaceae;g\_\_Achromobacter |
| 0.000212038110316 | 0.00% | k\_\_Bacteria;p\_\_Proteobacteria;c\_\_Alphaproteobacteria;o\_\_Rhizobiales;f\_\_Rhizobiaceae;g\_\_Phyllobacterium |
| 0.000212038110316 | 0.00% | k\_\_Bacteria;p\_\_Actinobacteria;c\_\_Rubrobacteria;o\_\_Rubrobacterales;f\_\_unidentified\_Rubrobacterales;g\_\_Rubrobacter |
| 0.000197902236296 | 0.00% | k\_\_Bacteria;p\_\_Actinobacteria;c\_\_Thermoleophilia;o\_\_Solirubrobacterales;f\_\_;g\_\_ |
| 0.000197902236295 | 0.00% | k\_\_Bacteria;p\_\_Firmicutes;c\_\_Bacilli;o\_\_Bacillales;f\_\_unidentified\_Bacillales;g\_\_Exiguobacterium |
| 0.000183766362274 | 0.00% | k\_\_Bacteria;p\_\_Firmicutes;c\_\_Erysipelotrichia;o\_\_Erysipelotrichales;f\_\_Erysipelotrichaceae;g\_\_Holdemania |
| 0.000183766362274 | 0.00% | k\_\_Bacteria;p\_\_Firmicutes;c\_\_Clostridia;o\_\_Clostridiales;f\_\_Ruminococcaceae;g\_\_Hydrogenoanaerobacterium |
| 0.000169630488253 | 0.00% | k\_\_Bacteria;p\_\_Proteobacteria;c\_\_Gammaproteobacteria;o\_\_unidentified\_Gammaproteobacteria;f\_\_Neisseriaceae;g\_\_Neisseria |
| 0.000169630488253 | 0.00% | k\_\_Bacteria;p\_\_Firmicutes;c\_\_Clostridia;o\_\_Clostridiales;f\_\_Peptostreptococcaceae;g\_\_Peptostreptococcus |
| 0.000155494614232 | 0.00% | k\_\_Bacteria;p\_\_Firmicutes;c\_\_Clostridia;o\_\_Clostridiales;f\_\_unidentified\_Clostridiales;g\_\_Parvimonas |
| 0.000155494614232 | 0.00% | k\_\_Bacteria;p\_\_Firmicutes;c\_\_Erysipelotrichia;o\_\_Erysipelotrichales;f\_\_Erysipelotrichaceae;g\_\_Turicibacter |
| 0.000155494614232 | 0.00% | k\_\_Bacteria;p\_\_Proteobacteria;c\_\_Gammaproteobacteria;o\_\_unidentified\_Gammaproteobacteria;f\_\_Burkholderiaceae;g\_\_Ralstonia |
| 0.000141358740211 | 0.00% | k\_\_Bacteria;p\_\_Proteobacteria;c\_\_Alphaproteobacteria;o\_\_unidentified\_Alphaproteobacteria;f\_\_unidentified\_Alphaproteobacteria;g\_\_Reyranella |
| 0.000141358740211 | 0.00% | k\_\_Bacteria;p\_\_Firmicutes;c\_\_Negativicutes;o\_\_Selenomonadales;f\_\_Veillonellaceae;g\_\_Allisonella |
| 0.00012722286619 | 0.00% | k\_\_Bacteria;p\_\_Firmicutes;c\_\_Clostridia;o\_\_Clostridiales;f\_\_Peptococcaceae;Other |
| 0.00012722286619 | 0.00% | k\_\_Bacteria;p\_\_Bacteroidetes;c\_\_Bacteroidia;o\_\_Bacteroidales;f\_\_Prevotellaceae;g\_\_ |
| 0.00012722286619 | 0.00% | k\_\_Bacteria;p\_\_Actinobacteria;c\_\_unidentified\_Actinobacteria;o\_\_Actinomycetales;f\_\_Actinomycetaceae;g\_\_Actinomyces |
| 0.000113086992169 | 0.00% | k\_\_Bacteria;p\_\_Firmicutes;c\_\_Clostridia;o\_\_Clostridiales;f\_\_Christensenellaceae;g\_\_ |
| 9.89511181477e-05 | 0.00% | k\_\_Bacteria;p\_\_Acidobacteria;c\_\_unidentified\_Acidobacteria;o\_\_unidentified\_Acidobacteria;f\_\_unidentified\_Acidobacteria;g\_\_Blastocatella |
| 9.89511181477e-05 | 0.00% | k\_\_Bacteria;p\_\_Bacteroidetes;c\_\_Bacteroidia;o\_\_Flavobacteriales;f\_\_Flavobacteriaceae;g\_\_Tenacibaculum |
| 8.48152441266e-05 | 0.00% | k\_\_Bacteria;p\_\_Proteobacteria;c\_\_Alphaproteobacteria;o\_\_Rhizobiales;Other;Other |
| 8.48152441266e-05 | 0.00% | k\_\_Bacteria;p\_\_Actinobacteria;c\_\_unidentified\_Actinobacteria;o\_\_Micrococcales;f\_\_Microbacteriaceae;Other |
| 8.48152441266e-05 | 0.00% | k\_\_Bacteria;p\_\_Firmicutes;c\_\_Clostridia;o\_\_Clostridiales;f\_\_Lachnospiraceae;g\_\_Agathobacter |
| 8.48152441266e-05 | 0.00% | k\_\_Bacteria;p\_\_Acidobacteria;c\_\_Holophagae;o\_\_;f\_\_;g\_\_ |
| 7.06793701055e-05 | 0.00% | k\_\_Bacteria;p\_\_Proteobacteria;c\_\_Gammaproteobacteria;o\_\_Xanthomonadales;f\_\_Xanthomonadaceae;g\_\_Arenimonas |
| 7.06793701055e-05 | 0.00% | k\_\_Bacteria;p\_\_Proteobacteria;c\_\_Alphaproteobacteria;o\_\_Sphingomonadales;f\_\_Sphingomonadaceae;g\_\_Novosphingobium |
| 7.06793701055e-05 | 0.00% | k\_\_Bacteria;p\_\_Firmicutes;c\_\_Bacilli;o\_\_Lactobacillales;f\_\_Leuconostocaceae;g\_\_Leuconostoc |
| 7.06793701055e-05 | 0.00% | k\_\_Bacteria;p\_\_Bacteroidetes;c\_\_Bacteroidia;o\_\_Flavobacteriales;f\_\_unidentified\_Flavobacteriales;g\_\_Chryseobacterium |
| 7.06793701055e-05 | 0.00% | k\_\_Bacteria;p\_\_Bacteroidetes;c\_\_Bacteroidia;o\_\_Chitinophagales;f\_\_Chitinophagaceae;g\_\_Segetibacter |
| 7.06793701055e-05 | 0.00% | k\_\_Bacteria;p\_\_Actinobacteria;c\_\_Coriobacteriia;o\_\_Coriobacteriales;f\_\_Atopobiaceae;g\_\_Atopobium |
| 7.06793701055e-05 | 0.00% | k\_\_Bacteria;p\_\_Acidobacteria;c\_\_unidentified\_Acidobacteria;o\_\_unidentified\_Acidobacteria;f\_\_Pyrinomonadaceae;g\_\_unidentified\_Pyrinomonadaceae |
| 7.06793701055e-05 | 0.00% | k\_\_Bacteria;p\_\_Proteobacteria;c\_\_Alphaproteobacteria;o\_\_Rhizobiales;f\_\_Rhizobiaceae;g\_\_Brucella |
| 7.06793701055e-05 | 0.00% | k\_\_Archaea;p\_\_Euryarchaeota;c\_\_Methanobacteria;o\_\_Methanobacteriales;f\_\_Methanobacteriaceae;g\_\_Methanobrevibacter |
| 5.65434960844e-05 | 0.00% | k\_\_Bacteria;p\_\_Proteobacteria;c\_\_Alphaproteobacteria;o\_\_unidentified\_Alphaproteobacteria;f\_\_unidentified\_Alphaproteobacteria;g\_\_Dongia |
| 5.65434960844e-05 | 0.00% | k\_\_Bacteria;p\_\_Proteobacteria;c\_\_Alphaproteobacteria;o\_\_Rhizobiales;f\_\_Xanthobacteraceae;g\_\_Bradyrhizobium |
| 5.65434960844e-05 | 0.00% | k\_\_Bacteria;p\_\_Actinobacteria;c\_\_unidentified\_Actinobacteria;o\_\_Propionibacteriales;f\_\_Nocardioidaceae;g\_\_Aeromicrobium |
| 5.65434960844e-05 | 0.00% | k\_\_Bacteria;p\_\_Actinobacteria;c\_\_unidentified\_Actinobacteria;o\_\_Corynebacteriales;f\_\_Corynebacteriaceae;g\_\_unidentified\_Corynebacteriaceae |
| 5.65434960844e-05 | 0.00% | k\_\_Bacteria;p\_\_Actinobacteria;c\_\_Acidimicrobiia;Other;Other;Other |
| 4.24076220633e-05 | 0.00% | k\_\_Bacteria;p\_\_Proteobacteria;Other;Other;Other;Other |
| 4.24076220633e-05 | 0.00% | k\_\_Bacteria;p\_\_Bacteroidetes;c\_\_Bacteroidia;o\_\_Cytophagales;Other;Other |
| 4.24076220633e-05 | 0.00% | k\_\_Bacteria;p\_\_Tenericutes;c\_\_Mollicutes;o\_\_Anaeroplasmatales;f\_\_Anaeroplasmataceae;g\_\_Anaeroplasma |
| 4.24076220633e-05 | 0.00% | k\_\_Bacteria;p\_\_Fusobacteria;c\_\_Fusobacteriia;o\_\_Fusobacteriales;f\_\_Fusobacteriaceae;Other |
| 4.24076220633e-05 | 0.00% | k\_\_Bacteria;p\_\_Bacteroidetes;c\_\_Bacteroidia;o\_\_Flavobacteriales;f\_\_Flavobacteriaceae;Other |
| 2.82717480422e-05 | 0.00% | k\_\_Bacteria;p\_\_unidentified\_Bacteria;c\_\_unidentified\_Bacteria;o\_\_unidentified\_Bacteria;f\_\_unidentified\_Bacteria;g\_\_unidentified\_Bacteria |
| 2.82717480422e-05 | 0.00% | k\_\_Bacteria;p\_\_unidentified\_Bacteria;c\_\_unidentified\_Bacteria;o\_\_Campylobacterales;f\_\_Campylobacteraceae;g\_\_Campylobacter |
| 2.82717480422e-05 | 0.00% | k\_\_Bacteria;p\_\_Verrucomicrobia;c\_\_Verrucomicrobiae;o\_\_Chthoniobacterales;f\_\_Chthoniobacteraceae;Other |
| 2.82717480422e-05 | 0.00% | k\_\_Bacteria;p\_\_Proteobacteria;c\_\_Gammaproteobacteria;o\_\_Alteromonadales;f\_\_Shewanellaceae;g\_\_Shewanella |
| 2.82717480422e-05 | 0.00% | k\_\_Bacteria;p\_\_Proteobacteria;c\_\_Alphaproteobacteria;o\_\_Rhizobiales;f\_\_Rhizobiaceae;g\_\_Ensifer |
| 2.82717480422e-05 | 0.00% | k\_\_Bacteria;p\_\_Proteobacteria;c\_\_Alphaproteobacteria;o\_\_Rhizobiales;f\_\_Beijerinckiaceae;g\_\_Methylobacterium |
| 2.82717480422e-05 | 0.00% | k\_\_Bacteria;p\_\_Proteobacteria;c\_\_Alphaproteobacteria;o\_\_Caulobacterales;f\_\_Caulobacteraceae;g\_\_Brevundimonas |
| 2.82717480422e-05 | 0.00% | k\_\_Bacteria;p\_\_Deinococcus-Thermus;c\_\_Deinococci;o\_\_Deinococcales;f\_\_Deinococcaceae;g\_\_Deinococcus |
| 2.82717480422e-05 | 0.00% | k\_\_Bacteria;p\_\_Bacteroidetes;c\_\_Bacteroidia;o\_\_Sphingobacteriales;f\_\_;g\_\_ |
| 2.82717480422e-05 | 0.00% | k\_\_Bacteria;p\_\_Armatimonadetes;c\_\_Fimbriimonadia;o\_\_Fimbriimonadales;f\_\_Fimbriimonadaceae;g\_\_ |
| 2.82717480422e-05 | 0.00% | k\_\_Bacteria;p\_\_Actinobacteria;c\_\_Thermoleophilia;o\_\_Solirubrobacterales;Other;Other |
| 2.82717480422e-05 | 0.00% | k\_\_Bacteria;p\_\_Actinobacteria;c\_\_Acidimicrobiia;o\_\_;f\_\_;g\_\_ |
| 1.41358740211e-05 | 0.00% | k\_\_Bacteria;p\_\_Proteobacteria;c\_\_Gammaproteobacteria;o\_\_Xanthomonadales;f\_\_Rhodanobacteraceae;g\_\_Rhodanobacter |
| 1.41358740211e-05 | 0.00% | k\_\_Bacteria;p\_\_Proteobacteria;c\_\_Gammaproteobacteria;o\_\_Xanthomonadales;f\_\_Rhodanobacteraceae;g\_\_Chujaibacter |
| 1.41358740211e-05 | 0.00% | k\_\_Bacteria;p\_\_Proteobacteria;c\_\_Alphaproteobacteria;o\_\_Rhodospirillales;f\_\_unidentified\_Rhodospirillales;g\_\_Defluviicoccus |
| 1.41358740211e-05 | 0.00% | k\_\_Bacteria;p\_\_Actinobacteria;c\_\_Thermoleophilia;o\_\_Gaiellales;f\_\_;g\_\_ |

|  |  |
| --- | --- |
|  | |
| Taxonomy Summary. Current Level: \_MD1 | |
| View Figure (.pdf)  View Legend (.pdf) |  |
|  |  |
| View Figure (.pdf)  View Legend (.pdf) |  |
|  |  |
|  |  |

|  |  |  |
| --- | --- | --- |
| Count | Pct | Taxonomy |
| 0.272016623788 | 27.20% | k\_\_Bacteria;p\_\_Bacteroidetes;c\_\_Bacteroidia;o\_\_Bacteroidales;f\_\_Muribaculaceae;g\_\_ |
| 0.228704305787 | 22.87% | k\_\_Bacteria;p\_\_Firmicutes;c\_\_Clostridia;o\_\_Clostridiales;f\_\_Lachnospiraceae;Other |
| 0.11963190184 | 11.96% | k\_\_Bacteria;p\_\_Bacteroidetes;c\_\_Bacteroidia;o\_\_Bacteroidales;f\_\_Bacteroidaceae;g\_\_Bacteroides |
| 0.0845042548981 | 8.45% | k\_\_Bacteria;p\_\_Firmicutes;c\_\_Clostridia;o\_\_Clostridiales;f\_\_Lachnospiraceae;g\_\_unidentified\_Lachnospiraceae |
| 0.0564304090922 | 5.64% | k\_\_Bacteria;p\_\_Proteobacteria;c\_\_Gammaproteobacteria;o\_\_Enterobacteriales;f\_\_Enterobacteriaceae;g\_\_unidentified\_Enterobacteriaceae |
| 0.0366967289588 | 3.67% | k\_\_Bacteria;p\_\_Firmicutes;c\_\_Clostridia;o\_\_Clostridiales;f\_\_Ruminococcaceae;Other |
| 0.0251901275056 | 2.52% | k\_\_Bacteria;p\_\_Firmicutes;c\_\_Clostridia;o\_\_Clostridiales;f\_\_Ruminococcaceae;g\_\_unidentified\_Ruminococcaceae |
| 0.0177829295185 | 1.78% | k\_\_Bacteria;p\_\_Firmicutes;c\_\_Clostridia;o\_\_Clostridiales;f\_\_Lachnospiraceae;g\_\_Blautia |
| 0.0162562551243 | 1.63% | k\_\_Bacteria;p\_\_Firmicutes;c\_\_Clostridia;o\_\_Clostridiales;f\_\_Lachnospiraceae;g\_\_Roseburia |
| 0.0151960645727 | 1.52% | k\_\_Bacteria;p\_\_Firmicutes;c\_\_Clostridia;o\_\_Clostridiales;f\_\_Lachnospiraceae;g\_\_Lachnoclostridium |
| 0.0142348251392 | 1.42% | k\_\_Bacteria;p\_\_Proteobacteria;c\_\_Gammaproteobacteria;o\_\_Enterobacteriales;f\_\_Enterobacteriaceae;Other |
| 0.0129767323514 | 1.30% | k\_\_Bacteria;p\_\_Bacteroidetes;c\_\_Bacteroidia;o\_\_Bacteroidales;f\_\_Rikenellaceae;g\_\_Alistipes |
| 0.0119730852959 | 1.20% | k\_\_Bacteria;p\_\_Bacteroidetes;c\_\_Bacteroidia;o\_\_Bacteroidales;f\_\_Prevotellaceae;g\_\_Alloprevotella |
| 0.00989511181476 | 0.99% | k\_\_Bacteria;p\_\_Proteobacteria;c\_\_Deltaproteobacteria;o\_\_Desulfovibrionales;f\_\_Desulfovibrionaceae;g\_\_Desulfovibrio |
| 0.00889146475927 | 0.89% | k\_\_Bacteria;p\_\_Firmicutes;c\_\_Clostridia;o\_\_Clostridiales;f\_\_Lachnospiraceae;g\_\_ |
| 0.00829775805038 | 0.83% | k\_\_Bacteria;p\_\_Bacteroidetes;c\_\_Bacteroidia;o\_\_Bacteroidales;f\_\_Muribaculaceae;g\_\_Muribaculum |
| 0.00761923609737 | 0.76% | k\_\_Bacteria;p\_\_Firmicutes;c\_\_Clostridia;o\_\_Clostridiales;f\_\_Ruminococcaceae;g\_\_Ruminiclostridium |
| 0.00694071414436 | 0.69% | k\_\_Bacteria;p\_\_Proteobacteria;c\_\_Gammaproteobacteria;o\_\_Enterobacteriales;f\_\_Enterobacteriaceae;g\_\_Proteus |
| 0.00500409940347 | 0.50% | k\_\_Bacteria;p\_\_Firmicutes;c\_\_Clostridia;o\_\_Clostridiales;f\_\_Ruminococcaceae;g\_\_Butyricicoccus |
| 0.00491928415934 | 0.49% | k\_\_Bacteria;p\_\_Firmicutes;c\_\_Clostridia;o\_\_Clostridiales;f\_\_Ruminococcaceae;g\_\_Oscillibacter |
| 0.0043538491985 | 0.44% | k\_\_Bacteria;p\_\_Bacteroidetes;c\_\_Bacteroidia;o\_\_Bacteroidales;f\_\_Tannerellaceae;g\_\_Parabacteroides |
| 0.00425489808035 | 0.43% | k\_\_Bacteria;p\_\_Firmicutes;c\_\_Clostridia;o\_\_Clostridiales;f\_\_Ruminococcaceae;g\_\_Anaerotruncus |
| 0.003350202143 | 0.34% | k\_\_Bacteria;p\_\_Firmicutes;c\_\_Bacilli;o\_\_Lactobacillales;f\_\_Lactobacillaceae;g\_\_Lactobacillus |
| 0.00294026179639 | 0.29% | k\_\_Bacteria;p\_\_Firmicutes;c\_\_Clostridia;o\_\_Clostridiales;Other;Other |
| 0.00272822368607 | 0.27% | k\_\_Bacteria;p\_\_Firmicutes;c\_\_Clostridia;o\_\_Clostridiales;f\_\_Ruminococcaceae;g\_\_Intestinimonas |
| 0.00240309858359 | 0.24% | k\_\_Bacteria;p\_\_Firmicutes;c\_\_Clostridia;o\_\_Clostridiales;f\_\_Lachnospiraceae;g\_\_Acetatifactor |
| 0.00214865285121 | 0.21% | k\_\_Bacteria;p\_\_Firmicutes;c\_\_Bacilli;o\_\_Lactobacillales;f\_\_Enterococcaceae;g\_\_Enterococcus |
| 0.00166803313449 | 0.17% | k\_\_Bacteria;p\_\_Verrucomicrobia;c\_\_Verrucomicrobiae;o\_\_Verrucomicrobiales;f\_\_Akkermansiaceae;g\_\_Akkermansia |
| 0.00139945152809 | 0.14% | k\_\_Bacteria;p\_\_Firmicutes;c\_\_Erysipelotrichia;o\_\_Erysipelotrichales;f\_\_Erysipelotrichaceae;g\_\_Dubosiella |
| 0.000862288315287 | 0.09% | k\_\_Bacteria;p\_\_Firmicutes;c\_\_Clostridia;o\_\_Clostridiales;f\_\_Peptococcaceae;g\_\_ |
| 0.000848152441265 | 0.08% | k\_\_Bacteria;p\_\_Firmicutes;c\_\_Erysipelotrichia;o\_\_Erysipelotrichales;f\_\_Erysipelotrichaceae;Other |
| 0.000819880693223 | 0.08% | k\_\_Bacteria;p\_\_Tenericutes;c\_\_Mollicutes;o\_\_;f\_\_;g\_\_ |
| 0.000819880693223 | 0.08% | k\_\_Bacteria;p\_\_Firmicutes;c\_\_Clostridia;o\_\_Clostridiales;f\_\_Ruminococcaceae;g\_\_Faecalibacterium |
| 0.000720929575076 | 0.07% | k\_\_Bacteria;p\_\_Firmicutes;c\_\_Clostridia;o\_\_Clostridiales;f\_\_Ruminococcaceae;g\_\_Harryflintia |
| 0.000678521953012 | 0.07% | k\_\_Bacteria;p\_\_Firmicutes;c\_\_Clostridia;o\_\_Clostridiales;f\_\_Lachnospiraceae;g\_\_Tyzzerella |
| 0.000579570834865 | 0.06% | k\_\_Bacteria;p\_\_Firmicutes;c\_\_Clostridia;o\_\_Clostridiales;f\_\_unidentified\_Clostridiales;g\_\_unidentified\_Clostridiales |
| 0.000381668598569 | 0.04% | k\_\_Bacteria;p\_\_Firmicutes;c\_\_Clostridia;o\_\_Clostridiales;f\_\_unidentified\_Clostridiales;g\_\_Anaerovorax |
| 0.000353396850527 | 0.04% | k\_\_Bacteria;p\_\_Bacteroidetes;c\_\_Bacteroidia;o\_\_Bacteroidales;f\_\_Prevotellaceae;g\_\_unidentified\_Prevotellaceae |
| 0.000353396850527 | 0.04% | k\_\_Bacteria;Other;Other;Other;Other;Other |
| 0.000296853354443 | 0.03% | k\_\_Bacteria;p\_\_Fusobacteria;c\_\_Fusobacteriia;o\_\_Fusobacteriales;f\_\_Fusobacteriaceae;g\_\_Fusobacterium |
| 0.000268581606401 | 0.03% | k\_\_Bacteria;p\_\_Firmicutes;c\_\_Clostridia;o\_\_Clostridiales;f\_\_Lachnospiraceae;g\_\_Fusicatenibacter |
| 0.000268581606401 | 0.03% | k\_\_Bacteria;p\_\_Bacteroidetes;c\_\_Bacteroidia;o\_\_Bacteroidales;f\_\_Marinifilaceae;g\_\_Butyricimonas |
| 0.000240309858359 | 0.02% | k\_\_Bacteria;p\_\_Firmicutes;c\_\_Clostridia;o\_\_Clostridiales;f\_\_Lachnospiraceae;g\_\_Marvinbryantia |
| 0.000240309858359 | 0.02% | k\_\_Bacteria;p\_\_Actinobacteria;c\_\_unidentified\_Actinobacteria;o\_\_Bifidobacteriales;f\_\_Bifidobacteriaceae;g\_\_Bifidobacterium |
| 0.000212038110316 | 0.02% | k\_\_Bacteria;p\_\_Firmicutes;Other;Other;Other;Other |
| 0.000197902236295 | 0.02% | k\_\_Bacteria;p\_\_Proteobacteria;c\_\_Gammaproteobacteria;o\_\_Pseudomonadales;f\_\_Pseudomonadaceae;g\_\_Pseudomonas |
| 0.000197902236295 | 0.02% | k\_\_Bacteria;p\_\_Firmicutes;c\_\_Clostridia;o\_\_Clostridiales;f\_\_Peptococcaceae;g\_\_Peptococcus |
| 0.000183766362274 | 0.02% | k\_\_Bacteria;p\_\_Proteobacteria;c\_\_Gammaproteobacteria;o\_\_unidentified\_Gammaproteobacteria;f\_\_Burkholderiaceae;g\_\_Parasutterella |
| 0.000183766362274 | 0.02% | k\_\_Bacteria;p\_\_Firmicutes;c\_\_Clostridia;o\_\_Clostridiales;f\_\_unidentified\_Clostridiales;Other |
| 0.000169630488253 | 0.02% | k\_\_Bacteria;p\_\_Firmicutes;c\_\_Negativicutes;o\_\_Selenomonadales;f\_\_Veillonellaceae;g\_\_Dialister |
| 0.000141358740211 | 0.01% | k\_\_Bacteria;p\_\_Proteobacteria;c\_\_Gammaproteobacteria;o\_\_unidentified\_Gammaproteobacteria;f\_\_Burkholderiaceae;g\_\_Sutterella |
| 0.00012722286619 | 0.01% | k\_\_Bacteria;p\_\_Firmicutes;c\_\_Clostridia;o\_\_Clostridiales;f\_\_Ruminococcaceae;g\_\_Caproiciproducens |
| 0.000113086992169 | 0.01% | k\_\_Bacteria;p\_\_unidentified\_Bacteria;c\_\_unidentified\_Bacteria;o\_\_Campylobacterales;f\_\_unidentified\_Campylobacterales;g\_\_Sulfurospirillum |
| 0.000113086992169 | 0.01% | k\_\_Bacteria;p\_\_Proteobacteria;c\_\_Gammaproteobacteria;o\_\_unidentified\_Gammaproteobacteria;f\_\_Burkholderiaceae;g\_\_Diaphorobacter |
| 0.000113086992169 | 0.01% | k\_\_Bacteria;p\_\_Proteobacteria;c\_\_Gammaproteobacteria;o\_\_unidentified\_Gammaproteobacteria;f\_\_Burkholderiaceae;g\_\_Delftia |
| 0.000113086992169 | 0.01% | k\_\_Bacteria;p\_\_Bacteroidetes;c\_\_Bacteroidia;o\_\_Bacteroidales;f\_\_Porphyromonadaceae;g\_\_Porphyromonas |
| 9.89511181476e-05 | 0.01% | k\_\_Bacteria;p\_\_Proteobacteria;c\_\_Gammaproteobacteria;o\_\_Pseudomonadales;f\_\_Moraxellaceae;g\_\_Acinetobacter |
| 9.89511181476e-05 | 0.01% | k\_\_Bacteria;p\_\_Melainabacteria;Other;Other;Other;Other |
| 9.89511181476e-05 | 0.01% | k\_\_Bacteria;p\_\_Firmicutes;c\_\_Negativicutes;o\_\_Selenomonadales;f\_\_Acidaminococcaceae;g\_\_Phascolarctobacterium |
| 9.89511181476e-05 | 0.01% | k\_\_Bacteria;p\_\_Firmicutes;c\_\_Clostridia;o\_\_Clostridiales;f\_\_Peptostreptococcaceae;g\_\_Romboutsia |
| 8.48152441265e-05 | 0.01% | k\_\_Bacteria;p\_\_Firmicutes;c\_\_Negativicutes;o\_\_Selenomonadales;f\_\_Veillonellaceae;g\_\_Veillonella |
| 8.48152441265e-05 | 0.01% | k\_\_Bacteria;p\_\_Firmicutes;c\_\_Clostridia;o\_\_Clostridiales;f\_\_Ruminococcaceae;g\_\_Subdoligranulum |
| 8.48152441265e-05 | 0.01% | k\_\_Bacteria;p\_\_Firmicutes;c\_\_Clostridia;o\_\_Clostridiales;f\_\_Lachnospiraceae;g\_\_Anaerostipes |
| 7.06793701055e-05 | 0.01% | k\_\_Bacteria;p\_\_Proteobacteria;c\_\_Gammaproteobacteria;o\_\_Xanthomonadales;f\_\_Xanthomonadaceae;g\_\_Stenotrophomonas |
| 7.06793701055e-05 | 0.01% | k\_\_Bacteria;p\_\_Firmicutes;c\_\_Erysipelotrichia;o\_\_Erysipelotrichales;f\_\_Erysipelotrichaceae;g\_\_Erysipelatoclostridium |
| 7.06793701055e-05 | 0.01% | k\_\_Bacteria;p\_\_Firmicutes;c\_\_Clostridia;o\_\_Clostridiales;f\_\_Peptostreptococcaceae;g\_\_Clostridioides |
| 7.06793701055e-05 | 0.01% | k\_\_Bacteria;p\_\_Firmicutes;c\_\_Clostridia;o\_\_Clostridiales;f\_\_Lachnospiraceae;g\_\_Lachnospira |
| 7.06793701055e-05 | 0.01% | k\_\_Bacteria;p\_\_Bacteroidetes;c\_\_Bacteroidia;o\_\_Bacteroidales;f\_\_Marinifilaceae;g\_\_Odoribacter |
| 5.65434960844e-05 | 0.01% | k\_\_Bacteria;p\_\_unidentified\_Bacteria;c\_\_unidentified\_Bacteria;o\_\_unidentified\_Bacteria;f\_\_unidentified\_Bacteria;g\_\_Candidatus\_Saccharimonas |
| 5.65434960844e-05 | 0.01% | k\_\_Bacteria;p\_\_Firmicutes;c\_\_Clostridia;o\_\_Clostridiales;f\_\_Ruminococcaceae;g\_\_Flavonifractor |
| 4.24076220633e-05 | 0.00% | k\_\_Bacteria;p\_\_Proteobacteria;c\_\_Gammaproteobacteria;o\_\_unidentified\_Gammaproteobacteria;f\_\_Burkholderiaceae;g\_\_Achromobacter |
| 4.24076220633e-05 | 0.00% | k\_\_Bacteria;p\_\_Proteobacteria;c\_\_Gammaproteobacteria;o\_\_Pasteurellales;f\_\_Pasteurellaceae;Other |
| 4.24076220633e-05 | 0.00% | k\_\_Bacteria;p\_\_Firmicutes;c\_\_Erysipelotrichia;o\_\_Erysipelotrichales;f\_\_Erysipelotrichaceae;g\_\_unidentified\_Erysipelotrichaceae |
| 4.24076220633e-05 | 0.00% | k\_\_Bacteria;p\_\_Firmicutes;c\_\_Erysipelotrichia;o\_\_Erysipelotrichales;f\_\_Erysipelotrichaceae;g\_\_Turicibacter |
| 4.24076220633e-05 | 0.00% | k\_\_Bacteria;p\_\_Firmicutes;c\_\_Erysipelotrichia;o\_\_Erysipelotrichales;f\_\_Erysipelotrichaceae;g\_\_Holdemania |
| 4.24076220633e-05 | 0.00% | k\_\_Bacteria;p\_\_Firmicutes;c\_\_Clostridia;o\_\_Clostridiales;f\_\_Peptostreptococcaceae;g\_\_Peptostreptococcus |
| 4.24076220633e-05 | 0.00% | k\_\_Bacteria;p\_\_Firmicutes;c\_\_Bacilli;o\_\_Lactobacillales;f\_\_Streptococcaceae;g\_\_Streptococcus |
| 4.24076220633e-05 | 0.00% | k\_\_Bacteria;p\_\_Firmicutes;c\_\_Bacilli;o\_\_Bacillales;f\_\_unidentified\_Bacillales;g\_\_Exiguobacterium |
| 4.24076220633e-05 | 0.00% | k\_\_Bacteria;p\_\_Bacteroidetes;c\_\_Bacteroidia;o\_\_Bacteroidales;f\_\_Barnesiellaceae;g\_\_Coprobacter |
| 4.24076220633e-05 | 0.00% | k\_\_Bacteria;p\_\_Bacteroidetes;c\_\_Bacteroidia;o\_\_Bacteroidales;f\_\_Barnesiellaceae;g\_\_Barnesiella |
| 2.82717480422e-05 | 0.00% | k\_\_Bacteria;p\_\_Proteobacteria;c\_\_Gammaproteobacteria;o\_\_unidentified\_Gammaproteobacteria;f\_\_Neisseriaceae;g\_\_Neisseria |
| 2.82717480422e-05 | 0.00% | k\_\_Bacteria;p\_\_Proteobacteria;c\_\_Gammaproteobacteria;o\_\_unidentified\_Gammaproteobacteria;f\_\_Burkholderiaceae;Other |
| 2.82717480422e-05 | 0.00% | k\_\_Bacteria;p\_\_Proteobacteria;c\_\_Deltaproteobacteria;o\_\_Desulfovibrionales;f\_\_Desulfovibrionaceae;g\_\_Bilophila |
| 2.82717480422e-05 | 0.00% | k\_\_Bacteria;p\_\_Proteobacteria;c\_\_Alphaproteobacteria;o\_\_Sphingomonadales;f\_\_Sphingomonadaceae;g\_\_Sphingomonas |
| 2.82717480422e-05 | 0.00% | k\_\_Bacteria;p\_\_Proteobacteria;c\_\_Alphaproteobacteria;o\_\_Rhizobiales;f\_\_Rhizobiaceae;g\_\_Phyllobacterium |
| 2.82717480422e-05 | 0.00% | k\_\_Bacteria;p\_\_Oxyphotobacteria;c\_\_unidentified\_Oxyphotobacteria;o\_\_unidentified\_Oxyphotobacteria;f\_\_unidentified\_Oxyphotobacteria;g\_\_unidentified\_Oxyphotobacteria |
| 2.82717480422e-05 | 0.00% | k\_\_Bacteria;p\_\_Firmicutes;c\_\_Negativicutes;o\_\_Selenomonadales;f\_\_Veillonellaceae;g\_\_Megasphaera |
| 2.82717480422e-05 | 0.00% | k\_\_Bacteria;p\_\_Firmicutes;c\_\_Negativicutes;o\_\_Selenomonadales;f\_\_Veillonellaceae;g\_\_Megamonas |
| 2.82717480422e-05 | 0.00% | k\_\_Bacteria;p\_\_Firmicutes;c\_\_Clostridia;o\_\_Clostridiales;f\_\_Lachnospiraceae;g\_\_Agathobacter |
| 2.82717480422e-05 | 0.00% | k\_\_Bacteria;p\_\_Firmicutes;c\_\_Bacilli;o\_\_Bacillales;f\_\_Staphylococcaceae;g\_\_Staphylococcus |
| 2.82717480422e-05 | 0.00% | k\_\_Bacteria;p\_\_Bacteroidetes;c\_\_Bacteroidia;o\_\_Bacteroidales;f\_\_Prevotellaceae;g\_\_Prevotella |
| 1.41358740211e-05 | 0.00% | k\_\_Bacteria;p\_\_Proteobacteria;c\_\_Gammaproteobacteria;o\_\_unidentified\_Gammaproteobacteria;f\_\_Burkholderiaceae;g\_\_Ralstonia |
| 1.41358740211e-05 | 0.00% | k\_\_Bacteria;p\_\_Proteobacteria;c\_\_Gammaproteobacteria;o\_\_Xanthomonadales;f\_\_Rhodanobacteraceae;g\_\_Chujaibacter |
| 1.41358740211e-05 | 0.00% | k\_\_Bacteria;p\_\_Proteobacteria;c\_\_Gammaproteobacteria;o\_\_Enterobacteriales;f\_\_Enterobacteriaceae;g\_\_Morganella |
| 1.41358740211e-05 | 0.00% | k\_\_Bacteria;p\_\_Proteobacteria;c\_\_Deltaproteobacteria;o\_\_Desulfovibrionales;f\_\_Desulfovibrionaceae;g\_\_ |
| 1.41358740211e-05 | 0.00% | k\_\_Bacteria;p\_\_Proteobacteria;c\_\_Alphaproteobacteria;o\_\_Rhizobiales;f\_\_Rhizobiaceae;g\_\_Ensifer |
| 1.41358740211e-05 | 0.00% | k\_\_Bacteria;p\_\_Firmicutes;c\_\_Clostridia;o\_\_Clostridiales;f\_\_Ruminococcaceae;g\_\_Hydrogenoanaerobacterium |
| 1.41358740211e-05 | 0.00% | k\_\_Bacteria;p\_\_Firmicutes;c\_\_Clostridia;o\_\_Clostridiales;f\_\_Christensenellaceae;g\_\_ |
| 1.41358740211e-05 | 0.00% | k\_\_Bacteria;p\_\_Actinobacteria;c\_\_unidentified\_Actinobacteria;o\_\_Corynebacteriales;f\_\_Nocardiaceae;g\_\_Rhodococcus |
| 1.41358740211e-05 | 0.00% | k\_\_Bacteria;p\_\_Actinobacteria;c\_\_unidentified\_Actinobacteria;o\_\_Actinomycetales;f\_\_Actinomycetaceae;g\_\_Actinomyces |
| 1.41358740211e-05 | 0.00% | k\_\_Bacteria;p\_\_Actinobacteria;c\_\_Thermoleophilia;o\_\_Solirubrobacterales;f\_\_;g\_\_ |

|  |  |
| --- | --- |
|  | |
| Taxonomy Summary. Current Level: \_MD2 | |
| View Figure (.pdf)  View Legend (.pdf) |  |
|  |  |
| View Figure (.pdf)  View Legend (.pdf) |  |
|  |  |
|  |  |

|  |  |  |
| --- | --- | --- |
| Count | Pct | Taxonomy |
| 0.2963868706 | 29.64% | k\_\_Bacteria;p\_\_Bacteroidetes;c\_\_Bacteroidia;o\_\_Bacteroidales;f\_\_Muribaculaceae;g\_\_ |
| 0.183511916542 | 18.35% | k\_\_Bacteria;p\_\_Firmicutes;c\_\_Clostridia;o\_\_Clostridiales;f\_\_Lachnospiraceae;Other |
| 0.133145797405 | 13.31% | k\_\_Bacteria;p\_\_Bacteroidetes;c\_\_Bacteroidia;o\_\_Bacteroidales;f\_\_Bacteroidaceae;g\_\_Bacteroides |
| 0.0662407056628 | 6.62% | k\_\_Bacteria;p\_\_Proteobacteria;c\_\_Gammaproteobacteria;o\_\_Enterobacteriales;f\_\_Enterobacteriaceae;g\_\_unidentified\_Enterobacteriaceae |
| 0.0658873088123 | 6.59% | k\_\_Bacteria;p\_\_Firmicutes;c\_\_Clostridia;o\_\_Clostridiales;f\_\_Lachnospiraceae;g\_\_unidentified\_Lachnospiraceae |
| 0.0363150603602 | 3.63% | k\_\_Bacteria;p\_\_Firmicutes;c\_\_Clostridia;o\_\_Clostridiales;f\_\_Ruminococcaceae;Other |
| 0.0272256933646 | 2.72% | k\_\_Bacteria;p\_\_Firmicutes;c\_\_Clostridia;o\_\_Clostridiales;f\_\_Ruminococcaceae;g\_\_unidentified\_Ruminococcaceae |
| 0.0188572559441 | 1.89% | k\_\_Bacteria;p\_\_Bacteroidetes;c\_\_Bacteroidia;o\_\_Bacteroidales;f\_\_Prevotellaceae;g\_\_Alloprevotella |
| 0.0172174945577 | 1.72% | k\_\_Bacteria;p\_\_Firmicutes;c\_\_Clostridia;o\_\_Clostridiales;f\_\_Lachnospiraceae;g\_\_Blautia |
| 0.0166803313449 | 1.67% | k\_\_Bacteria;p\_\_Proteobacteria;c\_\_Gammaproteobacteria;o\_\_Enterobacteriales;f\_\_Enterobacteriaceae;Other |
| 0.0158180430296 | 1.58% | k\_\_Bacteria;p\_\_Firmicutes;c\_\_Clostridia;o\_\_Clostridiales;f\_\_Lachnospiraceae;g\_\_Roseburia |
| 0.0128212377371 | 1.28% | k\_\_Bacteria;p\_\_Bacteroidetes;c\_\_Bacteroidia;o\_\_Bacteroidales;f\_\_Rikenellaceae;g\_\_Alistipes |
| 0.0115914166973 | 1.16% | k\_\_Bacteria;p\_\_Proteobacteria;c\_\_Deltaproteobacteria;o\_\_Desulfovibrionales;f\_\_Desulfovibrionaceae;g\_\_Desulfovibrio |
| 0.0111956122247 | 1.12% | k\_\_Bacteria;p\_\_Firmicutes;c\_\_Clostridia;o\_\_Clostridiales;f\_\_Lachnospiraceae;g\_\_Lachnoclostridium |
| 0.00893387238133 | 0.89% | k\_\_Bacteria;p\_\_Proteobacteria;c\_\_Gammaproteobacteria;o\_\_Enterobacteriales;f\_\_Enterobacteriaceae;g\_\_Proteus |
| 0.00855220378276 | 0.86% | k\_\_Bacteria;p\_\_Bacteroidetes;c\_\_Bacteroidia;o\_\_Bacteroidales;f\_\_Muribaculaceae;g\_\_Muribaculum |
| 0.0082412145543 | 0.82% | k\_\_Bacteria;p\_\_Firmicutes;c\_\_Clostridia;o\_\_Clostridiales;f\_\_Ruminococcaceae;g\_\_Ruminiclostridium |
| 0.00679935540414 | 0.68% | k\_\_Bacteria;p\_\_Firmicutes;c\_\_Clostridia;o\_\_Clostridiales;f\_\_Lachnospiraceae;g\_\_ |
| 0.00619151282124 | 0.62% | k\_\_Bacteria;p\_\_Firmicutes;c\_\_Clostridia;o\_\_Clostridiales;f\_\_Ruminococcaceae;g\_\_Oscillibacter |
| 0.00429730570241 | 0.43% | k\_\_Bacteria;p\_\_Firmicutes;c\_\_Bacilli;o\_\_Lactobacillales;f\_\_Lactobacillaceae;g\_\_Lactobacillus |
| 0.00407113171807 | 0.41% | k\_\_Bacteria;p\_\_Verrucomicrobia;c\_\_Verrucomicrobiae;o\_\_Verrucomicrobiales;f\_\_Akkermansiaceae;g\_\_Akkermansia |
| 0.00395804472591 | 0.40% | k\_\_Bacteria;p\_\_Firmicutes;c\_\_Clostridia;o\_\_Clostridiales;Other;Other |
| 0.00384495773374 | 0.38% | k\_\_Bacteria;p\_\_Bacteroidetes;c\_\_Bacteroidia;o\_\_Bacteroidales;f\_\_Tannerellaceae;g\_\_Parabacteroides |
| 0.00356224025331 | 0.36% | k\_\_Bacteria;p\_\_Firmicutes;c\_\_Clostridia;o\_\_Clostridiales;f\_\_Ruminococcaceae;g\_\_Anaerotruncus |
| 0.00354810437929 | 0.35% | k\_\_Bacteria;p\_\_Firmicutes;c\_\_Clostridia;o\_\_Clostridiales;f\_\_Ruminococcaceae;g\_\_Butyricicoccus |
| 0.00286958242628 | 0.29% | k\_\_Bacteria;p\_\_Firmicutes;c\_\_Bacilli;o\_\_Lactobacillales;f\_\_Enterococcaceae;g\_\_Enterococcus |
| 0.00253032144978 | 0.25% | k\_\_Bacteria;p\_\_Firmicutes;c\_\_Clostridia;o\_\_Clostridiales;f\_\_Ruminococcaceae;g\_\_Intestinimonas |
| 0.00190834299285 | 0.19% | k\_\_Bacteria;p\_\_Oxyphotobacteria;c\_\_unidentified\_Oxyphotobacteria;o\_\_unidentified\_Oxyphotobacteria;f\_\_unidentified\_Oxyphotobacteria;g\_\_unidentified\_Oxyphotobacteria |
| 0.00190834299285 | 0.19% | k\_\_Bacteria;p\_\_Firmicutes;c\_\_Clostridia;o\_\_Clostridiales;f\_\_Lachnospiraceae;g\_\_Acetatifactor |
| 0.00171044075655 | 0.17% | k\_\_Bacteria;Other;Other;Other;Other;Other |
| 0.00156908201634 | 0.16% | k\_\_Bacteria;p\_\_Firmicutes;c\_\_Erysipelotrichia;o\_\_Erysipelotrichales;f\_\_Erysipelotrichaceae;g\_\_Dubosiella |
| 0.001342908032 | 0.13% | k\_\_Bacteria;p\_\_Tenericutes;c\_\_Mollicutes;o\_\_;f\_\_;g\_\_ |
| 0.0012722286619 | 0.13% | k\_\_Bacteria;p\_\_Firmicutes;c\_\_Erysipelotrichia;o\_\_Erysipelotrichales;f\_\_Erysipelotrichaceae;Other |
| 0.000975375307455 | 0.10% | k\_\_Bacteria;p\_\_Firmicutes;c\_\_Clostridia;o\_\_Clostridiales;f\_\_Peptococcaceae;g\_\_ |
| 0.000763337197139 | 0.08% | k\_\_Bacteria;p\_\_Firmicutes;c\_\_Clostridia;o\_\_Clostridiales;f\_\_unidentified\_Clostridiales;g\_\_unidentified\_Clostridiales |
| 0.000692657827033 | 0.07% | k\_\_Bacteria;p\_\_Bacteroidetes;c\_\_Bacteroidia;o\_\_Bacteroidales;f\_\_Marinifilaceae;g\_\_Butyricimonas |
| 0.000636114330949 | 0.06% | k\_\_Bacteria;p\_\_Firmicutes;c\_\_Clostridia;o\_\_Clostridiales;f\_\_Ruminococcaceae;g\_\_Faecalibacterium |
| 0.000593706708886 | 0.06% | k\_\_Bacteria;p\_\_Firmicutes;c\_\_Clostridia;o\_\_Clostridiales;f\_\_Ruminococcaceae;g\_\_Harryflintia |
| 0.000466483842696 | 0.05% | k\_\_Bacteria;p\_\_Firmicutes;c\_\_Clostridia;o\_\_Clostridiales;f\_\_Lachnospiraceae;g\_\_Tyzzerella |
| 0.000310989228464 | 0.03% | k\_\_Bacteria;p\_\_Firmicutes;c\_\_Clostridia;o\_\_Clostridiales;f\_\_unidentified\_Clostridiales;g\_\_Anaerovorax |
| 0.000296853354443 | 0.03% | k\_\_Bacteria;p\_\_Bacteroidetes;c\_\_Bacteroidia;o\_\_Bacteroidales;f\_\_Prevotellaceae;g\_\_unidentified\_Prevotellaceae |
| 0.00025444573238 | 0.03% | k\_\_Bacteria;p\_\_unidentified\_Bacteria;c\_\_unidentified\_Bacteria;o\_\_unidentified\_Bacteria;f\_\_unidentified\_Bacteria;g\_\_Candidatus\_Saccharimonas |
| 0.00025444573238 | 0.03% | k\_\_Bacteria;p\_\_Fusobacteria;c\_\_Fusobacteriia;o\_\_Fusobacteriales;f\_\_Fusobacteriaceae;g\_\_Fusobacterium |
| 0.00025444573238 | 0.03% | k\_\_Bacteria;p\_\_Firmicutes;c\_\_Clostridia;o\_\_Clostridiales;f\_\_Ruminococcaceae;g\_\_Subdoligranulum |
| 0.00025444573238 | 0.03% | k\_\_Bacteria;p\_\_Firmicutes;Other;Other;Other;Other |
| 0.00025444573238 | 0.03% | k\_\_Bacteria;p\_\_Actinobacteria;c\_\_unidentified\_Actinobacteria;o\_\_Bifidobacteriales;f\_\_Bifidobacteriaceae;g\_\_Bifidobacterium |
| 0.000240309858359 | 0.02% | k\_\_Bacteria;p\_\_Proteobacteria;c\_\_Gammaproteobacteria;o\_\_unidentified\_Gammaproteobacteria;f\_\_Burkholderiaceae;g\_\_Sutterella |
| 0.000226173984337 | 0.02% | k\_\_Bacteria;p\_\_Firmicutes;c\_\_Clostridia;o\_\_Clostridiales;f\_\_Ruminococcaceae;g\_\_Caproiciproducens |
| 0.000212038110316 | 0.02% | k\_\_Bacteria;p\_\_Firmicutes;c\_\_Clostridia;o\_\_Clostridiales;f\_\_unidentified\_Clostridiales;Other |
| 0.000183766362274 | 0.02% | k\_\_Bacteria;p\_\_Proteobacteria;c\_\_Gammaproteobacteria;o\_\_Pseudomonadales;f\_\_Moraxellaceae;g\_\_Acinetobacter |
| 0.000169630488253 | 0.02% | k\_\_Bacteria;p\_\_Melainabacteria;Other;Other;Other;Other |
| 0.000169630488253 | 0.02% | k\_\_Bacteria;p\_\_Firmicutes;c\_\_Clostridia;o\_\_Clostridiales;f\_\_Peptococcaceae;g\_\_Peptococcus |
| 0.000169630488253 | 0.02% | k\_\_Bacteria;p\_\_Firmicutes;c\_\_Clostridia;o\_\_Clostridiales;f\_\_Lachnospiraceae;g\_\_Lachnospira |
| 0.000169630488253 | 0.02% | k\_\_Bacteria;p\_\_Firmicutes;c\_\_Clostridia;o\_\_Clostridiales;f\_\_Lachnospiraceae;g\_\_Fusicatenibacter |
| 0.000155494614232 | 0.02% | k\_\_Bacteria;p\_\_Proteobacteria;c\_\_Gammaproteobacteria;o\_\_unidentified\_Gammaproteobacteria;f\_\_Burkholderiaceae;g\_\_Parasutterella |
| 0.000141358740211 | 0.01% | k\_\_Bacteria;p\_\_unidentified\_Bacteria;c\_\_unidentified\_Bacteria;o\_\_Campylobacterales;f\_\_unidentified\_Campylobacterales;g\_\_Sulfurospirillum |
| 0.000141358740211 | 0.01% | k\_\_Bacteria;p\_\_Firmicutes;c\_\_Negativicutes;o\_\_Selenomonadales;f\_\_Veillonellaceae;g\_\_Dialister |
| 0.00012722286619 | 0.01% | k\_\_Bacteria;p\_\_Firmicutes;c\_\_Clostridia;o\_\_Clostridiales;f\_\_Lachnospiraceae;g\_\_Marvinbryantia |
| 0.000113086992169 | 0.01% | k\_\_Bacteria;p\_\_Firmicutes;c\_\_Erysipelotrichia;o\_\_Erysipelotrichales;f\_\_Erysipelotrichaceae;g\_\_unidentified\_Erysipelotrichaceae |
| 0.000113086992169 | 0.01% | k\_\_Bacteria;p\_\_Firmicutes;c\_\_Bacilli;o\_\_Lactobacillales;f\_\_Streptococcaceae;g\_\_Streptococcus |
| 0.000113086992169 | 0.01% | k\_\_Bacteria;p\_\_Bacteroidetes;c\_\_Bacteroidia;o\_\_Bacteroidales;f\_\_Prevotellaceae;g\_\_Prevotella |
| 9.89511181476e-05 | 0.01% | k\_\_Bacteria;p\_\_Proteobacteria;c\_\_Gammaproteobacteria;o\_\_unidentified\_Gammaproteobacteria;f\_\_Burkholderiaceae;g\_\_Delftia |
| 9.89511181476e-05 | 0.01% | k\_\_Bacteria;p\_\_Proteobacteria;c\_\_Deltaproteobacteria;o\_\_Desulfovibrionales;f\_\_Desulfovibrionaceae;g\_\_Bilophila |
| 9.89511181476e-05 | 0.01% | k\_\_Bacteria;p\_\_Firmicutes;c\_\_Negativicutes;o\_\_Selenomonadales;f\_\_Veillonellaceae;g\_\_Veillonella |
| 8.48152441265e-05 | 0.01% | k\_\_Bacteria;p\_\_Proteobacteria;c\_\_Gammaproteobacteria;o\_\_unidentified\_Gammaproteobacteria;f\_\_Burkholderiaceae;g\_\_Diaphorobacter |
| 8.48152441265e-05 | 0.01% | k\_\_Bacteria;p\_\_Proteobacteria;c\_\_Gammaproteobacteria;o\_\_Pseudomonadales;f\_\_Pseudomonadaceae;g\_\_Pseudomonas |
| 8.48152441265e-05 | 0.01% | k\_\_Bacteria;p\_\_Proteobacteria;c\_\_Deltaproteobacteria;o\_\_Desulfovibrionales;f\_\_Desulfovibrionaceae;g\_\_ |
| 8.48152441265e-05 | 0.01% | k\_\_Bacteria;p\_\_Firmicutes;c\_\_Negativicutes;o\_\_Selenomonadales;f\_\_Acidaminococcaceae;g\_\_Phascolarctobacterium |
| 8.48152441265e-05 | 0.01% | k\_\_Bacteria;p\_\_Bacteroidetes;c\_\_Bacteroidia;o\_\_Bacteroidales;f\_\_Marinifilaceae;g\_\_Odoribacter |
| 5.65434960844e-05 | 0.01% | k\_\_Bacteria;p\_\_Proteobacteria;c\_\_Gammaproteobacteria;o\_\_unidentified\_Gammaproteobacteria;f\_\_Neisseriaceae;g\_\_Neisseria |
| 5.65434960844e-05 | 0.01% | k\_\_Bacteria;p\_\_Proteobacteria;c\_\_Gammaproteobacteria;o\_\_unidentified\_Gammaproteobacteria;f\_\_Burkholderiaceae;Other |
| 5.65434960844e-05 | 0.01% | k\_\_Bacteria;p\_\_Proteobacteria;c\_\_Gammaproteobacteria;o\_\_Pasteurellales;f\_\_Pasteurellaceae;Other |
| 5.65434960844e-05 | 0.01% | k\_\_Bacteria;p\_\_Firmicutes;c\_\_Clostridia;o\_\_Clostridiales;f\_\_Ruminococcaceae;g\_\_Flavonifractor |
| 5.65434960844e-05 | 0.01% | k\_\_Bacteria;p\_\_Firmicutes;c\_\_Clostridia;o\_\_Clostridiales;f\_\_Peptostreptococcaceae;g\_\_Romboutsia |
| 5.65434960844e-05 | 0.01% | k\_\_Bacteria;p\_\_Firmicutes;c\_\_Clostridia;o\_\_Clostridiales;f\_\_Lachnospiraceae;g\_\_Anaerostipes |
| 4.24076220633e-05 | 0.00% | k\_\_Bacteria;p\_\_Proteobacteria;c\_\_Alphaproteobacteria;o\_\_Rhodospirillales;f\_\_unidentified\_Rhodospirillales;g\_\_Telmatospirillum |
| 4.24076220633e-05 | 0.00% | k\_\_Bacteria;p\_\_Firmicutes;c\_\_Clostridia;o\_\_Clostridiales;f\_\_Ruminococcaceae;g\_\_Hydrogenoanaerobacterium |
| 4.24076220633e-05 | 0.00% | k\_\_Bacteria;p\_\_Firmicutes;c\_\_Bacilli;o\_\_Lactobacillales;f\_\_Leuconostocaceae;g\_\_Leuconostoc |
| 4.24076220633e-05 | 0.00% | k\_\_Bacteria;p\_\_Actinobacteria;c\_\_unidentified\_Actinobacteria;o\_\_Corynebacteriales;f\_\_Nocardiaceae;g\_\_Rhodococcus |
| 4.24076220633e-05 | 0.00% | k\_\_Bacteria;p\_\_Actinobacteria;c\_\_Coriobacteriia;o\_\_Coriobacteriales;f\_\_Atopobiaceae;g\_\_Atopobium |
| 2.82717480422e-05 | 0.00% | k\_\_Bacteria;p\_\_Proteobacteria;c\_\_Gammaproteobacteria;o\_\_unidentified\_Gammaproteobacteria;f\_\_Burkholderiaceae;g\_\_Achromobacter |
| 2.82717480422e-05 | 0.00% | k\_\_Bacteria;p\_\_Proteobacteria;c\_\_Alphaproteobacteria;o\_\_Rhizobiales;f\_\_Rhizobiaceae;g\_\_Brucella |
| 2.82717480422e-05 | 0.00% | k\_\_Bacteria;p\_\_Firmicutes;c\_\_Bacilli;o\_\_Bacillales;f\_\_unidentified\_Bacillales;g\_\_Exiguobacterium |
| 2.82717480422e-05 | 0.00% | k\_\_Bacteria;p\_\_Bacteroidetes;c\_\_Bacteroidia;o\_\_Flavobacteriales;f\_\_Flavobacteriaceae;Other |
| 2.82717480422e-05 | 0.00% | k\_\_Bacteria;p\_\_Bacteroidetes;c\_\_Bacteroidia;o\_\_Bacteroidales;f\_\_Barnesiellaceae;g\_\_Barnesiella |
| 1.41358740211e-05 | 0.00% | k\_\_Bacteria;p\_\_Tenericutes;c\_\_Mollicutes;o\_\_Anaeroplasmatales;f\_\_Anaeroplasmataceae;g\_\_Anaeroplasma |
| 1.41358740211e-05 | 0.00% | k\_\_Bacteria;p\_\_Proteobacteria;c\_\_Gammaproteobacteria;o\_\_unidentified\_Gammaproteobacteria;f\_\_Burkholderiaceae;g\_\_Ralstonia |
| 1.41358740211e-05 | 0.00% | k\_\_Bacteria;p\_\_Firmicutes;c\_\_Erysipelotrichia;o\_\_Erysipelotrichales;f\_\_Erysipelotrichaceae;g\_\_Erysipelatoclostridium |
| 1.41358740211e-05 | 0.00% | k\_\_Bacteria;p\_\_Firmicutes;c\_\_Clostridia;o\_\_Clostridiales;f\_\_Peptostreptococcaceae;g\_\_Clostridioides |
| 1.41358740211e-05 | 0.00% | k\_\_Bacteria;p\_\_Firmicutes;c\_\_Clostridia;o\_\_Clostridiales;f\_\_Peptococcaceae;Other |
| 1.41358740211e-05 | 0.00% | k\_\_Bacteria;p\_\_Firmicutes;c\_\_Clostridia;o\_\_Clostridiales;f\_\_Christensenellaceae;g\_\_ |
| 1.41358740211e-05 | 0.00% | k\_\_Bacteria;p\_\_Bacteroidetes;c\_\_Bacteroidia;o\_\_Bacteroidales;f\_\_Porphyromonadaceae;g\_\_Porphyromonas |
| 1.41358740211e-05 | 0.00% | k\_\_Bacteria;p\_\_Bacteroidetes;c\_\_Bacteroidia;o\_\_Bacteroidales;f\_\_Barnesiellaceae;g\_\_Coprobacter |
| 1.41358740211e-05 | 0.00% | k\_\_Bacteria;p\_\_Actinobacteria;c\_\_unidentified\_Actinobacteria;o\_\_Corynebacteriales;f\_\_Corynebacteriaceae;g\_\_unidentified\_Corynebacteriaceae |

|  |  |
| --- | --- |
|  | |
| Taxonomy Summary. Current Level: \_MD3 | |
| View Figure (.pdf)  View Legend (.pdf) |  |
|  |  |
| View Figure (.pdf)  View Legend (.pdf) |  |
|  |  |
|  |  |

|  |  |  |
| --- | --- | --- |
| Count | Pct | Taxonomy |
| 0.316516355206 | 31.65% | k\_\_Bacteria;p\_\_Bacteroidetes;c\_\_Bacteroidia;o\_\_Bacteroidales;f\_\_Muribaculaceae;g\_\_ |
| 0.187074156795 | 18.71% | k\_\_Bacteria;p\_\_Firmicutes;c\_\_Clostridia;o\_\_Clostridiales;f\_\_Lachnospiraceae;Other |
| 0.121511973085 | 12.15% | k\_\_Bacteria;p\_\_Bacteroidetes;c\_\_Bacteroidia;o\_\_Bacteroidales;f\_\_Bacteroidaceae;g\_\_Bacteroides |
| 0.0703401091289 | 7.03% | k\_\_Bacteria;p\_\_Firmicutes;c\_\_Clostridia;o\_\_Clostridiales;f\_\_Lachnospiraceae;g\_\_unidentified\_Lachnospiraceae |
| 0.0665941025134 | 6.66% | k\_\_Bacteria;p\_\_Proteobacteria;c\_\_Gammaproteobacteria;o\_\_Enterobacteriales;f\_\_Enterobacteriaceae;g\_\_unidentified\_Enterobacteriaceae |
| 0.0325690537446 | 3.26% | k\_\_Bacteria;p\_\_Firmicutes;c\_\_Clostridia;o\_\_Clostridiales;f\_\_Ruminococcaceae;Other |
| 0.0271832857426 | 2.72% | k\_\_Bacteria;p\_\_Firmicutes;c\_\_Clostridia;o\_\_Clostridiales;f\_\_Ruminococcaceae;g\_\_unidentified\_Ruminococcaceae |
| 0.0184473155975 | 1.84% | k\_\_Bacteria;p\_\_Proteobacteria;c\_\_Gammaproteobacteria;o\_\_Enterobacteriales;f\_\_Enterobacteriaceae;Other |
| 0.0183483644794 | 1.83% | k\_\_Bacteria;p\_\_Firmicutes;c\_\_Clostridia;o\_\_Clostridiales;f\_\_Lachnospiraceae;g\_\_Roseburia |
| 0.0173447174239 | 1.73% | k\_\_Bacteria;p\_\_Firmicutes;c\_\_Clostridia;o\_\_Clostridiales;f\_\_Lachnospiraceae;g\_\_Blautia |
| 0.013400808572 | 1.34% | k\_\_Bacteria;p\_\_Proteobacteria;c\_\_Deltaproteobacteria;o\_\_Desulfovibrionales;f\_\_Desulfovibrionaceae;g\_\_Desulfovibrio |
| 0.0106725848859 | 1.07% | k\_\_Bacteria;p\_\_Bacteroidetes;c\_\_Bacteroidia;o\_\_Bacteroidales;f\_\_Prevotellaceae;g\_\_Alloprevotella |
| 0.0104181391535 | 1.04% | k\_\_Bacteria;p\_\_Firmicutes;c\_\_Clostridia;o\_\_Clostridiales;f\_\_Lachnospiraceae;g\_\_Lachnoclostridium |
| 0.0102343727913 | 1.02% | k\_\_Bacteria;p\_\_Bacteroidetes;c\_\_Bacteroidia;o\_\_Bacteroidales;f\_\_Rikenellaceae;g\_\_Alistipes |
| 0.00737892623901 | 0.74% | k\_\_Bacteria;p\_\_Bacteroidetes;c\_\_Bacteroidia;o\_\_Bacteroidales;f\_\_Muribaculaceae;g\_\_Muribaculum |
| 0.00732238274292 | 0.73% | k\_\_Bacteria;p\_\_Proteobacteria;c\_\_Gammaproteobacteria;o\_\_Enterobacteriales;f\_\_Enterobacteriaceae;g\_\_Proteus |
| 0.00698312176642 | 0.70% | k\_\_Bacteria;p\_\_Firmicutes;c\_\_Clostridia;o\_\_Clostridiales;f\_\_Lachnospiraceae;g\_\_ |
| 0.00688417064827 | 0.69% | k\_\_Bacteria;p\_\_Firmicutes;c\_\_Clostridia;o\_\_Clostridiales;f\_\_Ruminococcaceae;g\_\_Ruminiclostridium |
| 0.00548471912018 | 0.55% | k\_\_Bacteria;p\_\_Firmicutes;c\_\_Clostridia;o\_\_Clostridiales;f\_\_Ruminococcaceae;g\_\_Oscillibacter |
| 0.00409940346612 | 0.41% | k\_\_Bacteria;p\_\_Firmicutes;c\_\_Bacilli;o\_\_Lactobacillales;f\_\_Lactobacillaceae;g\_\_Lactobacillus |
| 0.00392977297786 | 0.39% | k\_\_Bacteria;p\_\_Bacteroidetes;c\_\_Bacteroidia;o\_\_Bacteroidales;f\_\_Tannerellaceae;g\_\_Parabacteroides |
| 0.00357637612734 | 0.36% | k\_\_Bacteria;p\_\_Firmicutes;c\_\_Clostridia;o\_\_Clostridiales;f\_\_Ruminococcaceae;g\_\_Butyricicoccus |
| 0.00337847389104 | 0.34% | k\_\_Bacteria;p\_\_Verrucomicrobia;c\_\_Verrucomicrobiae;o\_\_Verrucomicrobiales;f\_\_Akkermansiaceae;g\_\_Akkermansia |
| 0.00336433801702 | 0.34% | k\_\_Bacteria;p\_\_Firmicutes;c\_\_Bacilli;o\_\_Lactobacillales;f\_\_Enterococcaceae;g\_\_Enterococcus |
| 0.00333606626898 | 0.33% | k\_\_Bacteria;p\_\_Firmicutes;c\_\_Erysipelotrichia;o\_\_Erysipelotrichales;f\_\_Erysipelotrichaceae;g\_\_Dubosiella |
| 0.00320884340279 | 0.32% | k\_\_Bacteria;p\_\_Firmicutes;c\_\_Clostridia;o\_\_Clostridiales;f\_\_Ruminococcaceae;g\_\_Anaerotruncus |
| 0.00275649543411 | 0.28% | k\_\_Bacteria;p\_\_Firmicutes;c\_\_Clostridia;o\_\_Clostridiales;f\_\_Lachnospiraceae;g\_\_Acetatifactor |
| 0.00233241921348 | 0.23% | k\_\_Bacteria;p\_\_Firmicutes;c\_\_Clostridia;o\_\_Clostridiales;f\_\_Ruminococcaceae;g\_\_Intestinimonas |
| 0.00197902236295 | 0.20% | k\_\_Bacteria;p\_\_Firmicutes;c\_\_Clostridia;o\_\_Clostridiales;Other;Other |
| 0.00113086992169 | 0.11% | k\_\_Bacteria;p\_\_Firmicutes;c\_\_Erysipelotrichia;o\_\_Erysipelotrichales;f\_\_Erysipelotrichaceae;Other |
| 0.000932967685392 | 0.09% | k\_\_Bacteria;Other;Other;Other;Other;Other |
| 0.000819880693223 | 0.08% | k\_\_Bacteria;p\_\_Tenericutes;c\_\_Mollicutes;o\_\_;f\_\_;g\_\_ |
| 0.000819880693223 | 0.08% | k\_\_Bacteria;p\_\_Oxyphotobacteria;c\_\_unidentified\_Oxyphotobacteria;o\_\_unidentified\_Oxyphotobacteria;f\_\_unidentified\_Oxyphotobacteria;g\_\_unidentified\_Oxyphotobacteria |
| 0.000678521953012 | 0.07% | k\_\_Bacteria;p\_\_Firmicutes;c\_\_Clostridia;o\_\_Clostridiales;f\_\_unidentified\_Clostridiales;g\_\_unidentified\_Clostridiales |
| 0.000678521953012 | 0.07% | k\_\_Bacteria;p\_\_Firmicutes;c\_\_Clostridia;o\_\_Clostridiales;f\_\_Peptococcaceae;g\_\_ |
| 0.000551299086823 | 0.06% | k\_\_Bacteria;p\_\_Firmicutes;c\_\_Clostridia;o\_\_Clostridiales;f\_\_Ruminococcaceae;g\_\_Faecalibacterium |
| 0.00052302733878 | 0.05% | k\_\_Bacteria;p\_\_Firmicutes;c\_\_Clostridia;o\_\_Clostridiales;f\_\_Ruminococcaceae;g\_\_Harryflintia |
| 0.000508891464759 | 0.05% | k\_\_Bacteria;p\_\_Bacteroidetes;c\_\_Bacteroidia;o\_\_Bacteroidales;f\_\_Marinifilaceae;g\_\_Butyricimonas |
| 0.000409940346612 | 0.04% | k\_\_Bacteria;p\_\_Firmicutes;c\_\_Clostridia;o\_\_Clostridiales;f\_\_Lachnospiraceae;g\_\_Tyzzerella |
| 0.000367532724548 | 0.04% | k\_\_Bacteria;p\_\_Bacteroidetes;c\_\_Bacteroidia;o\_\_Bacteroidales;f\_\_Prevotellaceae;g\_\_unidentified\_Prevotellaceae |
| 0.000296853354443 | 0.03% | k\_\_Bacteria;p\_\_Firmicutes;Other;Other;Other;Other |
| 0.00025444573238 | 0.03% | k\_\_Bacteria;p\_\_Bacteroidetes;c\_\_Bacteroidia;o\_\_Bacteroidales;f\_\_Marinifilaceae;g\_\_Odoribacter |
| 0.000240309858359 | 0.02% | k\_\_Bacteria;p\_\_Firmicutes;c\_\_Clostridia;o\_\_Clostridiales;f\_\_unidentified\_Clostridiales;g\_\_Anaerovorax |
| 0.000240309858359 | 0.02% | k\_\_Bacteria;p\_\_Firmicutes;c\_\_Clostridia;o\_\_Clostridiales;f\_\_Ruminococcaceae;g\_\_Subdoligranulum |
| 0.000183766362274 | 0.02% | k\_\_Bacteria;p\_\_unidentified\_Bacteria;c\_\_unidentified\_Bacteria;o\_\_Campylobacterales;f\_\_unidentified\_Campylobacterales;g\_\_Sulfurospirillum |
| 0.000183766362274 | 0.02% | k\_\_Bacteria;p\_\_Fusobacteria;c\_\_Fusobacteriia;o\_\_Fusobacteriales;f\_\_Fusobacteriaceae;g\_\_Fusobacterium |
| 0.000183766362274 | 0.02% | k\_\_Bacteria;p\_\_Firmicutes;c\_\_Negativicutes;o\_\_Selenomonadales;f\_\_Veillonellaceae;g\_\_Dialister |
| 0.000183766362274 | 0.02% | k\_\_Bacteria;p\_\_Firmicutes;c\_\_Clostridia;o\_\_Clostridiales;f\_\_Ruminococcaceae;g\_\_Flavonifractor |
| 0.000183766362274 | 0.02% | k\_\_Bacteria;p\_\_Firmicutes;c\_\_Clostridia;o\_\_Clostridiales;f\_\_Peptococcaceae;g\_\_Peptococcus |
| 0.000155494614232 | 0.02% | k\_\_Bacteria;p\_\_Firmicutes;c\_\_Clostridia;o\_\_Clostridiales;f\_\_Lachnospiraceae;g\_\_Anaerostipes |
| 0.000155494614232 | 0.02% | k\_\_Bacteria;p\_\_Actinobacteria;c\_\_Rubrobacteria;o\_\_Rubrobacterales;f\_\_unidentified\_Rubrobacterales;g\_\_Rubrobacter |
| 0.000141358740211 | 0.01% | k\_\_Bacteria;p\_\_Proteobacteria;c\_\_Gammaproteobacteria;o\_\_Pasteurellales;f\_\_Pasteurellaceae;Other |
| 0.000141358740211 | 0.01% | k\_\_Bacteria;p\_\_Proteobacteria;c\_\_Alphaproteobacteria;o\_\_unidentified\_Alphaproteobacteria;f\_\_unidentified\_Alphaproteobacteria;g\_\_Reyranella |
| 0.000141358740211 | 0.01% | k\_\_Bacteria;p\_\_Firmicutes;c\_\_Clostridia;o\_\_Clostridiales;f\_\_Lachnospiraceae;g\_\_Fusicatenibacter |
| 0.000141358740211 | 0.01% | k\_\_Bacteria;p\_\_Actinobacteria;c\_\_unidentified\_Actinobacteria;o\_\_Bifidobacteriales;f\_\_Bifidobacteriaceae;g\_\_Bifidobacterium |
| 0.00012722286619 | 0.01% | k\_\_Bacteria;p\_\_unidentified\_Bacteria;c\_\_unidentified\_Bacteria;o\_\_unidentified\_Bacteria;f\_\_unidentified\_Bacteria;g\_\_Candidatus\_Saccharimonas |
| 0.00012722286619 | 0.01% | k\_\_Bacteria;p\_\_Proteobacteria;c\_\_Gammaproteobacteria;o\_\_unidentified\_Gammaproteobacteria;f\_\_Burkholderiaceae;g\_\_Sutterella |
| 0.00012722286619 | 0.01% | k\_\_Bacteria;p\_\_Proteobacteria;c\_\_Gammaproteobacteria;o\_\_unidentified\_Gammaproteobacteria;f\_\_Burkholderiaceae;g\_\_Diaphorobacter |
| 0.00012722286619 | 0.01% | k\_\_Bacteria;p\_\_Firmicutes;c\_\_Clostridia;o\_\_Clostridiales;f\_\_unidentified\_Clostridiales;Other |
| 0.00012722286619 | 0.01% | k\_\_Bacteria;p\_\_Firmicutes;c\_\_Clostridia;o\_\_Clostridiales;f\_\_Lachnospiraceae;g\_\_Lachnospira |
| 0.000113086992169 | 0.01% | k\_\_Bacteria;p\_\_Proteobacteria;c\_\_Gammaproteobacteria;o\_\_Pseudomonadales;f\_\_Pseudomonadaceae;g\_\_Pseudomonas |
| 0.000113086992169 | 0.01% | k\_\_Bacteria;p\_\_Firmicutes;c\_\_Negativicutes;o\_\_Selenomonadales;f\_\_Veillonellaceae;g\_\_Veillonella |
| 0.000113086992169 | 0.01% | k\_\_Bacteria;p\_\_Firmicutes;c\_\_Clostridia;o\_\_Clostridiales;f\_\_Lachnospiraceae;g\_\_Marvinbryantia |
| 0.000113086992169 | 0.01% | k\_\_Bacteria;p\_\_Firmicutes;c\_\_Bacilli;o\_\_Lactobacillales;f\_\_Streptococcaceae;g\_\_Streptococcus |
| 9.89511181476e-05 | 0.01% | k\_\_Bacteria;p\_\_Proteobacteria;c\_\_Gammaproteobacteria;o\_\_unidentified\_Gammaproteobacteria;f\_\_Burkholderiaceae;g\_\_Parasutterella |
| 9.89511181476e-05 | 0.01% | k\_\_Bacteria;p\_\_Proteobacteria;c\_\_Gammaproteobacteria;o\_\_Pseudomonadales;f\_\_Moraxellaceae;g\_\_Acinetobacter |
| 9.89511181476e-05 | 0.01% | k\_\_Bacteria;p\_\_Firmicutes;c\_\_Erysipelotrichia;o\_\_Erysipelotrichales;f\_\_Erysipelotrichaceae;g\_\_unidentified\_Erysipelotrichaceae |
| 9.89511181476e-05 | 0.01% | k\_\_Bacteria;p\_\_Firmicutes;c\_\_Clostridia;o\_\_Clostridiales;f\_\_Ruminococcaceae;g\_\_Caproiciproducens |
| 8.48152441265e-05 | 0.01% | k\_\_Bacteria;p\_\_Melainabacteria;Other;Other;Other;Other |
| 8.48152441265e-05 | 0.01% | k\_\_Bacteria;p\_\_Bacteroidetes;c\_\_Bacteroidia;o\_\_Bacteroidales;f\_\_Prevotellaceae;g\_\_Prevotella |
| 7.06793701055e-05 | 0.01% | k\_\_Bacteria;p\_\_Proteobacteria;c\_\_Alphaproteobacteria;o\_\_Rhizobiales;Other;Other |
| 7.06793701055e-05 | 0.01% | k\_\_Bacteria;p\_\_Bacteroidetes;c\_\_Bacteroidia;o\_\_Bacteroidales;f\_\_Porphyromonadaceae;g\_\_Porphyromonas |
| 7.06793701055e-05 | 0.01% | k\_\_Bacteria;p\_\_Acidobacteria;c\_\_unidentified\_Acidobacteria;o\_\_unidentified\_Acidobacteria;f\_\_Pyrinomonadaceae;g\_\_unidentified\_Pyrinomonadaceae |
| 5.65434960844e-05 | 0.01% | k\_\_Bacteria;p\_\_Proteobacteria;c\_\_Alphaproteobacteria;o\_\_Sphingomonadales;f\_\_Sphingomonadaceae;g\_\_Sphingomonas |
| 5.65434960844e-05 | 0.01% | k\_\_Bacteria;p\_\_Firmicutes;c\_\_Clostridia;o\_\_Clostridiales;f\_\_Ruminococcaceae;g\_\_Hydrogenoanaerobacterium |
| 5.65434960844e-05 | 0.01% | k\_\_Bacteria;p\_\_Bacteroidetes;c\_\_Bacteroidia;o\_\_Bacteroidales;f\_\_Barnesiellaceae;g\_\_Barnesiella |
| 5.65434960844e-05 | 0.01% | k\_\_Bacteria;p\_\_Actinobacteria;c\_\_unidentified\_Actinobacteria;o\_\_Propionibacteriales;f\_\_Nocardioidaceae;g\_\_Aeromicrobium |
| 5.65434960844e-05 | 0.01% | k\_\_Bacteria;p\_\_Actinobacteria;c\_\_Thermoleophilia;o\_\_Solirubrobacterales;f\_\_;g\_\_ |
| 5.65434960844e-05 | 0.01% | k\_\_Bacteria;p\_\_Actinobacteria;c\_\_Acidimicrobiia;Other;Other;Other |
| 5.65434960844e-05 | 0.01% | k\_\_Bacteria;p\_\_Acidobacteria;c\_\_Holophagae;o\_\_;f\_\_;g\_\_ |
| 4.24076220633e-05 | 0.00% | k\_\_Bacteria;p\_\_Proteobacteria;c\_\_Gammaproteobacteria;o\_\_unidentified\_Gammaproteobacteria;f\_\_Burkholderiaceae;g\_\_Delftia |
| 4.24076220633e-05 | 0.00% | k\_\_Bacteria;p\_\_Proteobacteria;c\_\_Gammaproteobacteria;o\_\_Xanthomonadales;f\_\_Xanthomonadaceae;g\_\_Stenotrophomonas |
| 4.24076220633e-05 | 0.00% | k\_\_Bacteria;p\_\_Proteobacteria;c\_\_Alphaproteobacteria;o\_\_unidentified\_Alphaproteobacteria;f\_\_unidentified\_Alphaproteobacteria;g\_\_Dongia |
| 4.24076220633e-05 | 0.00% | k\_\_Bacteria;p\_\_Proteobacteria;c\_\_Alphaproteobacteria;o\_\_Rhodospirillales;f\_\_unidentified\_Rhodospirillales;g\_\_Telmatospirillum |
| 4.24076220633e-05 | 0.00% | k\_\_Bacteria;p\_\_Firmicutes;c\_\_Negativicutes;o\_\_Selenomonadales;f\_\_Veillonellaceae;g\_\_Allisonella |
| 4.24076220633e-05 | 0.00% | k\_\_Bacteria;p\_\_Firmicutes;c\_\_Clostridia;o\_\_Clostridiales;f\_\_Peptostreptococcaceae;g\_\_Clostridioides |
| 4.24076220633e-05 | 0.00% | k\_\_Bacteria;p\_\_Bacteroidetes;c\_\_Bacteroidia;o\_\_Cytophagales;Other;Other |
| 4.24076220633e-05 | 0.00% | k\_\_Bacteria;p\_\_Actinobacteria;c\_\_unidentified\_Actinobacteria;o\_\_Corynebacteriales;f\_\_Nocardiaceae;g\_\_Rhodococcus |
| 4.24076220633e-05 | 0.00% | k\_\_Bacteria;p\_\_Acidobacteria;c\_\_unidentified\_Acidobacteria;o\_\_unidentified\_Acidobacteria;f\_\_unidentified\_Acidobacteria;g\_\_Blastocatella |
| 2.82717480422e-05 | 0.00% | k\_\_Bacteria;p\_\_Proteobacteria;c\_\_Gammaproteobacteria;o\_\_unidentified\_Gammaproteobacteria;f\_\_Burkholderiaceae;g\_\_Ralstonia |
| 2.82717480422e-05 | 0.00% | k\_\_Bacteria;p\_\_Proteobacteria;c\_\_Gammaproteobacteria;o\_\_unidentified\_Gammaproteobacteria;f\_\_Burkholderiaceae;g\_\_Achromobacter |
| 2.82717480422e-05 | 0.00% | k\_\_Bacteria;p\_\_Proteobacteria;c\_\_Deltaproteobacteria;o\_\_Desulfovibrionales;f\_\_Desulfovibrionaceae;g\_\_Bilophila |
| 2.82717480422e-05 | 0.00% | k\_\_Bacteria;p\_\_Fusobacteria;c\_\_Fusobacteriia;o\_\_Fusobacteriales;f\_\_Fusobacteriaceae;Other |
| 2.82717480422e-05 | 0.00% | k\_\_Bacteria;p\_\_Firmicutes;c\_\_Negativicutes;o\_\_Selenomonadales;f\_\_Veillonellaceae;g\_\_Megasphaera |
| 2.82717480422e-05 | 0.00% | k\_\_Bacteria;p\_\_Firmicutes;c\_\_Negativicutes;o\_\_Selenomonadales;f\_\_Veillonellaceae;g\_\_Megamonas |
| 2.82717480422e-05 | 0.00% | k\_\_Bacteria;p\_\_Firmicutes;c\_\_Negativicutes;o\_\_Selenomonadales;f\_\_Acidaminococcaceae;g\_\_Phascolarctobacterium |
| 2.82717480422e-05 | 0.00% | k\_\_Bacteria;p\_\_Firmicutes;c\_\_Erysipelotrichia;o\_\_Erysipelotrichales;f\_\_Erysipelotrichaceae;g\_\_Faecalitalea |
| 2.82717480422e-05 | 0.00% | k\_\_Bacteria;p\_\_Firmicutes;c\_\_Clostridia;o\_\_Clostridiales;f\_\_Peptococcaceae;Other |
| 2.82717480422e-05 | 0.00% | k\_\_Bacteria;p\_\_Actinobacteria;c\_\_Thermoleophilia;o\_\_Solirubrobacterales;Other;Other |
| 2.82717480422e-05 | 0.00% | k\_\_Bacteria;p\_\_Actinobacteria;c\_\_Acidimicrobiia;o\_\_;f\_\_;g\_\_ |
| 1.41358740211e-05 | 0.00% | k\_\_Bacteria;p\_\_Proteobacteria;c\_\_Gammaproteobacteria;o\_\_unidentified\_Gammaproteobacteria;f\_\_Burkholderiaceae;Other |
| 1.41358740211e-05 | 0.00% | k\_\_Bacteria;p\_\_Proteobacteria;c\_\_Gammaproteobacteria;o\_\_Enterobacteriales;f\_\_Enterobacteriaceae;g\_\_Morganella |
| 1.41358740211e-05 | 0.00% | k\_\_Bacteria;p\_\_Proteobacteria;c\_\_Alphaproteobacteria;o\_\_Rhizobiales;f\_\_Xanthobacteraceae;g\_\_Bradyrhizobium |
| 1.41358740211e-05 | 0.00% | k\_\_Bacteria;p\_\_Firmicutes;c\_\_Negativicutes;o\_\_Selenomonadales;f\_\_Acidaminococcaceae;g\_\_Acidaminococcus |
| 1.41358740211e-05 | 0.00% | k\_\_Bacteria;p\_\_Firmicutes;c\_\_Erysipelotrichia;o\_\_Erysipelotrichales;f\_\_Erysipelotrichaceae;g\_\_Turicibacter |
| 1.41358740211e-05 | 0.00% | k\_\_Bacteria;p\_\_Firmicutes;c\_\_Clostridia;o\_\_Clostridiales;f\_\_Peptostreptococcaceae;g\_\_Romboutsia |
| 1.41358740211e-05 | 0.00% | k\_\_Bacteria;p\_\_Firmicutes;c\_\_Clostridia;o\_\_Clostridiales;f\_\_Christensenellaceae;g\_\_ |
| 1.41358740211e-05 | 0.00% | k\_\_Bacteria;p\_\_Firmicutes;c\_\_Bacilli;o\_\_Bacillales;f\_\_Staphylococcaceae;g\_\_Staphylococcus |
| 1.41358740211e-05 | 0.00% | k\_\_Bacteria;p\_\_Actinobacteria;c\_\_unidentified\_Actinobacteria;o\_\_Actinomycetales;f\_\_Actinomycetaceae;g\_\_Actinomyces |

|  |  |
| --- | --- |
|  | |
| Taxonomy Summary. Current Level: \_MD4 | |
| View Figure (.pdf)  View Legend (.pdf) |  |
|  |  |
| View Figure (.pdf)  View Legend (.pdf) |  |
|  |  |
|  |  |

|  |  |  |
| --- | --- | --- |
| Count | Pct | Taxonomy |
| 0.323343982358 | 32.33% | k\_\_Bacteria;p\_\_Bacteroidetes;c\_\_Bacteroidia;o\_\_Bacteroidales;f\_\_Muribaculaceae;g\_\_ |
| 0.291750303921 | 29.18% | k\_\_Bacteria;p\_\_Bacteroidetes;c\_\_Bacteroidia;o\_\_Bacteroidales;f\_\_Bacteroidaceae;g\_\_Bacteroides |
| 0.0852534562212 | 8.53% | k\_\_Bacteria;p\_\_Firmicutes;c\_\_Clostridia;o\_\_Clostridiales;f\_\_Lachnospiraceae;Other |
| 0.0438494812134 | 4.38% | k\_\_Bacteria;p\_\_Proteobacteria;c\_\_Gammaproteobacteria;o\_\_Enterobacteriales;f\_\_Enterobacteriaceae;g\_\_unidentified\_Enterobacteriaceae |
| 0.0291199004834 | 2.91% | k\_\_Bacteria;p\_\_Bacteroidetes;c\_\_Bacteroidia;o\_\_Bacteroidales;f\_\_Prevotellaceae;g\_\_Alloprevotella |
| 0.0287523677589 | 2.88% | k\_\_Bacteria;p\_\_Firmicutes;c\_\_Clostridia;o\_\_Clostridiales;f\_\_Ruminococcaceae;g\_\_unidentified\_Ruminococcaceae |
| 0.026801617144 | 2.68% | k\_\_Bacteria;p\_\_Firmicutes;c\_\_Clostridia;o\_\_Clostridiales;f\_\_Ruminococcaceae;Other |
| 0.023437279127 | 2.34% | k\_\_Bacteria;p\_\_Bacteroidetes;c\_\_Bacteroidia;o\_\_Bacteroidales;f\_\_Rikenellaceae;g\_\_Alistipes |
| 0.0211331316615 | 2.11% | k\_\_Bacteria;p\_\_Proteobacteria;c\_\_Gammaproteobacteria;o\_\_Enterobacteriales;f\_\_Enterobacteriaceae;Other |
| 0.0206101043228 | 2.06% | k\_\_Bacteria;p\_\_Firmicutes;c\_\_Clostridia;o\_\_Clostridiales;f\_\_Lachnospiraceae;g\_\_unidentified\_Lachnospiraceae |
| 0.0120296287919 | 1.20% | k\_\_Bacteria;p\_\_Firmicutes;c\_\_Clostridia;o\_\_Clostridiales;f\_\_Lachnospiraceae;g\_\_Roseburia |
| 0.0103474597834 | 1.03% | k\_\_Bacteria;p\_\_Proteobacteria;c\_\_Gammaproteobacteria;o\_\_Enterobacteriales;f\_\_Enterobacteriaceae;g\_\_Proteus |
| 0.0103333239094 | 1.03% | k\_\_Bacteria;p\_\_Verrucomicrobia;c\_\_Verrucomicrobiae;o\_\_Verrucomicrobiales;f\_\_Akkermansiaceae;g\_\_Akkermansia |
| 0.0101636934212 | 1.02% | k\_\_Bacteria;p\_\_Firmicutes;c\_\_Clostridia;o\_\_Clostridiales;f\_\_Lachnospiraceae;g\_\_Lachnoclostridium |
| 0.00798676882192 | 0.80% | k\_\_Bacteria;p\_\_Proteobacteria;c\_\_Deltaproteobacteria;o\_\_Desulfovibrionales;f\_\_Desulfovibrionaceae;g\_\_Desulfovibrio |
| 0.00768991546747 | 0.77% | k\_\_Bacteria;p\_\_Firmicutes;c\_\_Clostridia;o\_\_Clostridiales;f\_\_Lachnospiraceae;g\_\_Blautia |
| 0.00699725764044 | 0.70% | k\_\_Bacteria;p\_\_Bacteroidetes;c\_\_Bacteroidia;o\_\_Bacteroidales;f\_\_Tannerellaceae;g\_\_Parabacteroides |
| 0.00585225184473 | 0.59% | k\_\_Bacteria;p\_\_Bacteroidetes;c\_\_Bacteroidia;o\_\_Bacteroidales;f\_\_Muribaculaceae;g\_\_Muribaculum |
| 0.00498996352945 | 0.50% | k\_\_Bacteria;p\_\_Firmicutes;c\_\_Bacilli;o\_\_Lactobacillales;f\_\_Enterococcaceae;g\_\_Enterococcus |
| 0.00477792541913 | 0.48% | k\_\_Bacteria;p\_\_Firmicutes;c\_\_Bacilli;o\_\_Lactobacillales;f\_\_Lactobacillaceae;g\_\_Lactobacillus |
| 0.00306748466258 | 0.31% | k\_\_Bacteria;p\_\_Firmicutes;c\_\_Clostridia;o\_\_Clostridiales;f\_\_Ruminococcaceae;g\_\_Ruminiclostridium |
| 0.00302507704051 | 0.30% | k\_\_Bacteria;p\_\_Firmicutes;c\_\_Clostridia;o\_\_Clostridiales;f\_\_Ruminococcaceae;g\_\_Anaerotruncus |
| 0.00233241921348 | 0.23% | k\_\_Bacteria;p\_\_Firmicutes;c\_\_Clostridia;o\_\_Clostridiales;Other;Other |
| 0.00202142998502 | 0.20% | k\_\_Bacteria;p\_\_Firmicutes;c\_\_Clostridia;o\_\_Clostridiales;f\_\_Ruminococcaceae;g\_\_Oscillibacter |
| 0.001342908032 | 0.13% | k\_\_Bacteria;p\_\_Bacteroidetes;c\_\_Bacteroidia;o\_\_Bacteroidales;f\_\_Marinifilaceae;g\_\_Butyricimonas |
| 0.00131463628396 | 0.13% | k\_\_Bacteria;p\_\_Firmicutes;c\_\_Clostridia;o\_\_Clostridiales;f\_\_Ruminococcaceae;g\_\_Intestinimonas |
| 0.00122982103983 | 0.12% | k\_\_Bacteria;p\_\_Firmicutes;c\_\_Clostridia;o\_\_Clostridiales;f\_\_Lachnospiraceae;g\_\_ |
| 0.00115914166973 | 0.12% | k\_\_Bacteria;p\_\_Firmicutes;c\_\_Erysipelotrichia;o\_\_Erysipelotrichales;f\_\_Erysipelotrichaceae;Other |
| 0.000692657827033 | 0.07% | k\_\_Bacteria;p\_\_Firmicutes;c\_\_Clostridia;o\_\_Clostridiales;f\_\_unidentified\_Clostridiales;g\_\_unidentified\_Clostridiales |
| 0.000678521953012 | 0.07% | k\_\_Bacteria;p\_\_Firmicutes;c\_\_Clostridia;o\_\_Clostridiales;f\_\_Ruminococcaceae;g\_\_Faecalibacterium |
| 0.000664386078991 | 0.07% | k\_\_Bacteria;p\_\_unidentified\_Bacteria;c\_\_unidentified\_Bacteria;o\_\_unidentified\_Bacteria;f\_\_unidentified\_Bacteria;g\_\_Candidatus\_Saccharimonas |
| 0.000565434960844 | 0.06% | k\_\_Bacteria;p\_\_Firmicutes;c\_\_Clostridia;o\_\_Clostridiales;f\_\_unidentified\_Clostridiales;g\_\_Anaerovorax |
| 0.000508891464759 | 0.05% | k\_\_Bacteria;p\_\_Firmicutes;c\_\_Clostridia;o\_\_Clostridiales;f\_\_Ruminococcaceae;g\_\_Butyricicoccus |
| 0.000494755590738 | 0.05% | k\_\_Bacteria;p\_\_Bacteroidetes;c\_\_Bacteroidia;o\_\_Bacteroidales;f\_\_Prevotellaceae;g\_\_unidentified\_Prevotellaceae |
| 0.000325125102485 | 0.03% | k\_\_Bacteria;p\_\_Fusobacteria;c\_\_Fusobacteriia;o\_\_Fusobacteriales;f\_\_Fusobacteriaceae;g\_\_Fusobacterium |
| 0.000310989228464 | 0.03% | k\_\_Bacteria;p\_\_Firmicutes;c\_\_Clostridia;o\_\_Clostridiales;f\_\_Peptococcaceae;g\_\_ |
| 0.000296853354443 | 0.03% | k\_\_Bacteria;p\_\_Melainabacteria;Other;Other;Other;Other |
| 0.000282717480422 | 0.03% | k\_\_Bacteria;p\_\_Tenericutes;c\_\_Mollicutes;o\_\_;f\_\_;g\_\_ |
| 0.00025444573238 | 0.03% | k\_\_Bacteria;p\_\_Firmicutes;c\_\_Erysipelotrichia;o\_\_Erysipelotrichales;f\_\_Erysipelotrichaceae;g\_\_Dubosiella |
| 0.00025444573238 | 0.03% | k\_\_Bacteria;p\_\_Firmicutes;c\_\_Clostridia;o\_\_Clostridiales;f\_\_Ruminococcaceae;g\_\_Subdoligranulum |
| 0.000226173984337 | 0.02% | k\_\_Bacteria;p\_\_Firmicutes;c\_\_Clostridia;o\_\_Clostridiales;f\_\_Lachnospiraceae;g\_\_Fusicatenibacter |
| 0.000212038110316 | 0.02% | k\_\_Bacteria;p\_\_Firmicutes;c\_\_Clostridia;o\_\_Clostridiales;f\_\_Ruminococcaceae;g\_\_Harryflintia |
| 0.000197902236295 | 0.02% | k\_\_Bacteria;p\_\_Actinobacteria;c\_\_unidentified\_Actinobacteria;o\_\_Bifidobacteriales;f\_\_Bifidobacteriaceae;g\_\_Bifidobacterium |
| 0.000183766362274 | 0.02% | k\_\_Bacteria;p\_\_Firmicutes;c\_\_Negativicutes;o\_\_Selenomonadales;f\_\_Veillonellaceae;g\_\_Dialister |
| 0.000169630488253 | 0.02% | k\_\_Bacteria;p\_\_Firmicutes;c\_\_Clostridia;o\_\_Clostridiales;f\_\_Lachnospiraceae;g\_\_Acetatifactor |
| 0.000155494614232 | 0.02% | k\_\_Bacteria;p\_\_Proteobacteria;c\_\_Gammaproteobacteria;o\_\_unidentified\_Gammaproteobacteria;f\_\_Burkholderiaceae;g\_\_Sutterella |
| 0.000141358740211 | 0.01% | k\_\_Bacteria;p\_\_Proteobacteria;c\_\_Gammaproteobacteria;o\_\_unidentified\_Gammaproteobacteria;f\_\_Burkholderiaceae;g\_\_Parasutterella |
| 0.000141358740211 | 0.01% | k\_\_Bacteria;p\_\_Firmicutes;c\_\_Clostridia;o\_\_Clostridiales;f\_\_Lachnospiraceae;g\_\_Tyzzerella |
| 0.000141358740211 | 0.01% | k\_\_Bacteria;p\_\_Firmicutes;c\_\_Clostridia;o\_\_Clostridiales;f\_\_Lachnospiraceae;g\_\_Marvinbryantia |
| 0.000141358740211 | 0.01% | k\_\_Bacteria;p\_\_Bacteroidetes;c\_\_Bacteroidia;o\_\_Bacteroidales;f\_\_Marinifilaceae;g\_\_Odoribacter |
| 0.000113086992169 | 0.01% | k\_\_Bacteria;p\_\_Proteobacteria;c\_\_Gammaproteobacteria;o\_\_Pseudomonadales;f\_\_Moraxellaceae;g\_\_Acinetobacter |
| 0.000113086992169 | 0.01% | k\_\_Bacteria;p\_\_Firmicutes;c\_\_Bacilli;o\_\_Lactobacillales;f\_\_Streptococcaceae;g\_\_Streptococcus |
| 0.000113086992169 | 0.01% | k\_\_Bacteria;p\_\_Bacteroidetes;c\_\_Bacteroidia;o\_\_Bacteroidales;f\_\_Prevotellaceae;g\_\_Prevotella |
| 0.000113086992169 | 0.01% | k\_\_Bacteria;p\_\_Bacteroidetes;c\_\_Bacteroidia;o\_\_Bacteroidales;f\_\_Porphyromonadaceae;g\_\_Porphyromonas |
| 0.000113086992169 | 0.01% | k\_\_Bacteria;p\_\_Bacteroidetes;c\_\_Bacteroidia;o\_\_Bacteroidales;f\_\_Barnesiellaceae;g\_\_Barnesiella |
| 9.89511181476e-05 | 0.01% | k\_\_Bacteria;p\_\_Firmicutes;c\_\_Erysipelotrichia;o\_\_Erysipelotrichales;f\_\_Erysipelotrichaceae;g\_\_unidentified\_Erysipelotrichaceae |
| 8.48152441265e-05 | 0.01% | k\_\_Bacteria;p\_\_Proteobacteria;c\_\_Gammaproteobacteria;o\_\_Pasteurellales;f\_\_Pasteurellaceae;Other |
| 8.48152441265e-05 | 0.01% | k\_\_Bacteria;p\_\_Firmicutes;c\_\_Clostridia;o\_\_Clostridiales;f\_\_Ruminococcaceae;g\_\_Caproiciproducens |
| 7.06793701055e-05 | 0.01% | k\_\_Bacteria;p\_\_Proteobacteria;c\_\_Gammaproteobacteria;o\_\_unidentified\_Gammaproteobacteria;f\_\_Burkholderiaceae;g\_\_Ralstonia |
| 7.06793701055e-05 | 0.01% | k\_\_Bacteria;p\_\_Proteobacteria;c\_\_Gammaproteobacteria;o\_\_unidentified\_Gammaproteobacteria;f\_\_Burkholderiaceae;g\_\_Diaphorobacter |
| 7.06793701055e-05 | 0.01% | k\_\_Bacteria;p\_\_Proteobacteria;c\_\_Gammaproteobacteria;o\_\_unidentified\_Gammaproteobacteria;f\_\_Burkholderiaceae;g\_\_Delftia |
| 7.06793701055e-05 | 0.01% | k\_\_Bacteria;p\_\_Oxyphotobacteria;c\_\_unidentified\_Oxyphotobacteria;o\_\_unidentified\_Oxyphotobacteria;f\_\_unidentified\_Oxyphotobacteria;g\_\_unidentified\_Oxyphotobacteria |
| 7.06793701055e-05 | 0.01% | k\_\_Bacteria;p\_\_Firmicutes;c\_\_Erysipelotrichia;o\_\_Erysipelotrichales;f\_\_Erysipelotrichaceae;g\_\_Faecalitalea |
| 7.06793701055e-05 | 0.01% | k\_\_Bacteria;p\_\_Firmicutes;Other;Other;Other;Other |
| 7.06793701055e-05 | 0.01% | k\_\_Bacteria;p\_\_Bacteroidetes;c\_\_Bacteroidia;o\_\_Bacteroidales;f\_\_Barnesiellaceae;g\_\_Coprobacter |
| 5.65434960844e-05 | 0.01% | k\_\_Bacteria;p\_\_Firmicutes;c\_\_Clostridia;o\_\_Clostridiales;f\_\_unidentified\_Clostridiales;Other |
| 5.65434960844e-05 | 0.01% | k\_\_Bacteria;p\_\_Firmicutes;c\_\_Clostridia;o\_\_Clostridiales;f\_\_Peptostreptococcaceae;g\_\_Romboutsia |
| 5.65434960844e-05 | 0.01% | k\_\_Bacteria;p\_\_Firmicutes;c\_\_Clostridia;o\_\_Clostridiales;f\_\_Lachnospiraceae;g\_\_Lachnospira |
| 5.65434960844e-05 | 0.01% | k\_\_Bacteria;p\_\_Actinobacteria;c\_\_unidentified\_Actinobacteria;o\_\_Corynebacteriales;f\_\_Nocardiaceae;g\_\_Rhodococcus |
| 4.24076220633e-05 | 0.00% | k\_\_Bacteria;p\_\_Proteobacteria;c\_\_Deltaproteobacteria;o\_\_Desulfovibrionales;f\_\_Desulfovibrionaceae;g\_\_Bilophila |
| 4.24076220633e-05 | 0.00% | k\_\_Bacteria;p\_\_Proteobacteria;c\_\_Alphaproteobacteria;o\_\_Rhizobiales;f\_\_Rhizobiaceae;g\_\_Phyllobacterium |
| 4.24076220633e-05 | 0.00% | k\_\_Bacteria;p\_\_Firmicutes;c\_\_Negativicutes;o\_\_Selenomonadales;f\_\_Veillonellaceae;g\_\_Veillonella |
| 4.24076220633e-05 | 0.00% | k\_\_Bacteria;p\_\_Firmicutes;c\_\_Negativicutes;o\_\_Selenomonadales;f\_\_Veillonellaceae;g\_\_Allisonella |
| 4.24076220633e-05 | 0.00% | k\_\_Bacteria;p\_\_Firmicutes;c\_\_Negativicutes;o\_\_Selenomonadales;f\_\_Acidaminococcaceae;g\_\_Phascolarctobacterium |
| 4.24076220633e-05 | 0.00% | k\_\_Bacteria;p\_\_Firmicutes;c\_\_Clostridia;o\_\_Clostridiales;f\_\_unidentified\_Clostridiales;g\_\_Parvimonas |
| 4.24076220633e-05 | 0.00% | k\_\_Bacteria;p\_\_Firmicutes;c\_\_Clostridia;o\_\_Clostridiales;f\_\_Peptostreptococcaceae;g\_\_Peptostreptococcus |
| 4.24076220633e-05 | 0.00% | k\_\_Bacteria;p\_\_Bacteroidetes;c\_\_Bacteroidia;o\_\_Bacteroidales;f\_\_Prevotellaceae;Other |
| 2.82717480422e-05 | 0.00% | k\_\_Bacteria;p\_\_unidentified\_Bacteria;c\_\_unidentified\_Bacteria;o\_\_unidentified\_Bacteria;f\_\_unidentified\_Bacteria;g\_\_unidentified\_Bacteria |
| 2.82717480422e-05 | 0.00% | k\_\_Bacteria;p\_\_unidentified\_Bacteria;c\_\_unidentified\_Bacteria;o\_\_Campylobacterales;f\_\_unidentified\_Campylobacterales;g\_\_Sulfurospirillum |
| 2.82717480422e-05 | 0.00% | k\_\_Bacteria;p\_\_Proteobacteria;c\_\_Gammaproteobacteria;o\_\_Xanthomonadales;f\_\_Xanthomonadaceae;g\_\_Stenotrophomonas |
| 2.82717480422e-05 | 0.00% | k\_\_Bacteria;p\_\_Proteobacteria;c\_\_Gammaproteobacteria;o\_\_Pseudomonadales;f\_\_Pseudomonadaceae;g\_\_Pseudomonas |
| 2.82717480422e-05 | 0.00% | k\_\_Bacteria;p\_\_Proteobacteria;c\_\_Alphaproteobacteria;o\_\_Sphingomonadales;f\_\_Sphingomonadaceae;g\_\_Sphingomonas |
| 2.82717480422e-05 | 0.00% | k\_\_Bacteria;p\_\_Firmicutes;c\_\_Negativicutes;o\_\_Selenomonadales;f\_\_Veillonellaceae;g\_\_Megasphaera |
| 2.82717480422e-05 | 0.00% | k\_\_Bacteria;p\_\_Firmicutes;c\_\_Erysipelotrichia;o\_\_Erysipelotrichales;f\_\_Erysipelotrichaceae;g\_\_Holdemania |
| 2.82717480422e-05 | 0.00% | k\_\_Bacteria;p\_\_Firmicutes;c\_\_Clostridia;o\_\_Clostridiales;f\_\_Lachnospiraceae;g\_\_Anaerostipes |
| 1.41358740211e-05 | 0.00% | k\_\_Bacteria;p\_\_Proteobacteria;c\_\_Gammaproteobacteria;o\_\_unidentified\_Gammaproteobacteria;f\_\_Neisseriaceae;g\_\_Neisseria |
| 1.41358740211e-05 | 0.00% | k\_\_Bacteria;p\_\_Proteobacteria;c\_\_Gammaproteobacteria;o\_\_unidentified\_Gammaproteobacteria;f\_\_Burkholderiaceae;Other |
| 1.41358740211e-05 | 0.00% | k\_\_Bacteria;p\_\_Proteobacteria;c\_\_Alphaproteobacteria;o\_\_Rhizobiales;f\_\_Rhizobiaceae;g\_\_Ensifer |
| 1.41358740211e-05 | 0.00% | k\_\_Bacteria;p\_\_Firmicutes;c\_\_Clostridia;o\_\_Clostridiales;f\_\_Ruminococcaceae;g\_\_Flavonifractor |
| 1.41358740211e-05 | 0.00% | k\_\_Bacteria;p\_\_Firmicutes;c\_\_Clostridia;o\_\_Clostridiales;f\_\_Peptostreptococcaceae;g\_\_Clostridioides |
| 1.41358740211e-05 | 0.00% | k\_\_Bacteria;p\_\_Firmicutes;c\_\_Clostridia;o\_\_Clostridiales;f\_\_Peptococcaceae;g\_\_Peptococcus |
| 1.41358740211e-05 | 0.00% | k\_\_Bacteria;p\_\_Firmicutes;c\_\_Clostridia;o\_\_Clostridiales;f\_\_Lachnospiraceae;g\_\_Agathobacter |
| 1.41358740211e-05 | 0.00% | k\_\_Bacteria;p\_\_Firmicutes;c\_\_Bacilli;o\_\_Bacillales;f\_\_unidentified\_Bacillales;g\_\_Exiguobacterium |
| 1.41358740211e-05 | 0.00% | k\_\_Bacteria;p\_\_Actinobacteria;c\_\_unidentified\_Actinobacteria;o\_\_Micrococcales;f\_\_Microbacteriaceae;Other |

|  |  |
| --- | --- |
|  | |
| Taxonomy Summary. Current Level: \_MD5 | |
| View Figure (.pdf)  View Legend (.pdf) |  |
|  |  |
| View Figure (.pdf)  View Legend (.pdf) |  |
|  |  |
|  |  |

|  |  |  |
| --- | --- | --- |
| Count | Pct | Taxonomy |
| 0.29631619123 | 29.63% | k\_\_Bacteria;p\_\_Bacteroidetes;c\_\_Bacteroidia;o\_\_Bacteroidales;f\_\_Muribaculaceae;g\_\_ |
| 0.198821068107 | 19.88% | k\_\_Bacteria;p\_\_Firmicutes;c\_\_Clostridia;o\_\_Clostridiales;f\_\_Lachnospiraceae;Other |
| 0.171128890899 | 17.11% | k\_\_Bacteria;p\_\_Bacteroidetes;c\_\_Bacteroidia;o\_\_Bacteroidales;f\_\_Bacteroidaceae;g\_\_Bacteroides |
| 0.0718243759012 | 7.18% | k\_\_Bacteria;p\_\_Firmicutes;c\_\_Clostridia;o\_\_Clostridiales;f\_\_Lachnospiraceae;g\_\_unidentified\_Lachnospiraceae |
| 0.035551723163 | 3.56% | k\_\_Bacteria;p\_\_Firmicutes;c\_\_Clostridia;o\_\_Clostridiales;f\_\_Ruminococcaceae;Other |
| 0.0350428316983 | 3.50% | k\_\_Bacteria;p\_\_Firmicutes;c\_\_Clostridia;o\_\_Clostridiales;f\_\_Ruminococcaceae;g\_\_unidentified\_Ruminococcaceae |
| 0.0229425235362 | 2.29% | k\_\_Bacteria;p\_\_Firmicutes;c\_\_Clostridia;o\_\_Clostridiales;f\_\_Lachnospiraceae;g\_\_Lachnoclostridium |
| 0.0175143479121 | 1.75% | k\_\_Bacteria;p\_\_Firmicutes;c\_\_Clostridia;o\_\_Clostridiales;f\_\_Lachnospiraceae;g\_\_Blautia |
| 0.0138248847926 | 1.38% | k\_\_Bacteria;p\_\_Bacteroidetes;c\_\_Bacteroidia;o\_\_Bacteroidales;f\_\_Rikenellaceae;g\_\_Alistipes |
| 0.0134997596901 | 1.35% | k\_\_Bacteria;p\_\_Proteobacteria;c\_\_Deltaproteobacteria;o\_\_Desulfovibrionales;f\_\_Desulfovibrionaceae;g\_\_Desulfovibrio |
| 0.0119872211699 | 1.20% | k\_\_Bacteria;p\_\_Firmicutes;c\_\_Bacilli;o\_\_Lactobacillales;f\_\_Lactobacillaceae;g\_\_Lactobacillus |
| 0.0117469113115 | 1.17% | k\_\_Bacteria;p\_\_Proteobacteria;c\_\_Gammaproteobacteria;o\_\_Enterobacteriales;f\_\_Enterobacteriaceae;g\_\_unidentified\_Enterobacteriaceae |
| 0.0114500579571 | 1.15% | k\_\_Bacteria;p\_\_Firmicutes;c\_\_Clostridia;o\_\_Clostridiales;f\_\_Lachnospiraceae;g\_\_Roseburia |
| 0.0106019055158 | 1.06% | k\_\_Bacteria;p\_\_Firmicutes;c\_\_Bacilli;o\_\_Lactobacillales;f\_\_Enterococcaceae;g\_\_Enterococcus |
| 0.00996579118487 | 1.00% | k\_\_Bacteria;p\_\_Firmicutes;c\_\_Clostridia;o\_\_Clostridiales;f\_\_Ruminococcaceae;g\_\_Butyricicoccus |
| 0.00798676882192 | 0.80% | k\_\_Bacteria;p\_\_Bacteroidetes;c\_\_Bacteroidia;o\_\_Bacteroidales;f\_\_Prevotellaceae;g\_\_Alloprevotella |
| 0.00530095275791 | 0.53% | k\_\_Bacteria;p\_\_Bacteroidetes;c\_\_Bacteroidia;o\_\_Bacteroidales;f\_\_Tannerellaceae;g\_\_Parabacteroides |
| 0.004693110175 | 0.47% | k\_\_Bacteria;p\_\_Firmicutes;c\_\_Clostridia;o\_\_Clostridiales;Other;Other |
| 0.00439625682056 | 0.44% | k\_\_Bacteria;p\_\_Firmicutes;c\_\_Clostridia;o\_\_Clostridiales;f\_\_Lachnospiraceae;g\_\_ |
| 0.00417008283622 | 0.42% | k\_\_Bacteria;p\_\_Proteobacteria;c\_\_Gammaproteobacteria;o\_\_Enterobacteriales;f\_\_Enterobacteriaceae;Other |
| 0.0041559469622 | 0.42% | k\_\_Bacteria;p\_\_Tenericutes;c\_\_Mollicutes;o\_\_;f\_\_;g\_\_ |
| 0.00404285997003 | 0.40% | k\_\_Bacteria;p\_\_Bacteroidetes;c\_\_Bacteroidia;o\_\_Bacteroidales;f\_\_Muribaculaceae;g\_\_Muribaculum |
| 0.00347742500919 | 0.35% | k\_\_Bacteria;p\_\_Proteobacteria;c\_\_Gammaproteobacteria;o\_\_Enterobacteriales;f\_\_Enterobacteriaceae;g\_\_Proteus |
| 0.00333606626898 | 0.33% | k\_\_Bacteria;p\_\_Firmicutes;c\_\_Clostridia;o\_\_Clostridiales;f\_\_Ruminococcaceae;g\_\_Anaerotruncus |
| 0.00323711515083 | 0.32% | k\_\_Bacteria;p\_\_Firmicutes;c\_\_Clostridia;o\_\_Clostridiales;f\_\_Ruminococcaceae;g\_\_Oscillibacter |
| 0.00312402815866 | 0.31% | k\_\_Bacteria;p\_\_Firmicutes;c\_\_Clostridia;o\_\_Clostridiales;f\_\_Ruminococcaceae;g\_\_Intestinimonas |
| 0.00264340844194 | 0.26% | k\_\_Bacteria;p\_\_Firmicutes;c\_\_Clostridia;o\_\_Clostridiales;f\_\_Lachnospiraceae;g\_\_Acetatifactor |
| 0.0022758757174 | 0.23% | k\_\_Bacteria;p\_\_Firmicutes;c\_\_Clostridia;o\_\_Clostridiales;f\_\_Ruminococcaceae;g\_\_Ruminiclostridium |
| 0.00132877215798 | 0.13% | k\_\_Bacteria;p\_\_Firmicutes;c\_\_Clostridia;o\_\_Clostridiales;f\_\_Ruminococcaceae;g\_\_Harryflintia |
| 0.00108846229962 | 0.11% | k\_\_Bacteria;p\_\_Firmicutes;c\_\_Clostridia;o\_\_Clostridiales;f\_\_Lachnospiraceae;g\_\_Tyzzerella |
| 0.000961239433434 | 0.10% | k\_\_Bacteria;p\_\_Firmicutes;c\_\_Clostridia;o\_\_Clostridiales;f\_\_Ruminococcaceae;g\_\_Caproiciproducens |
| 0.000848152441265 | 0.08% | k\_\_Bacteria;p\_\_Firmicutes;c\_\_Clostridia;o\_\_Clostridiales;f\_\_unidentified\_Clostridiales;g\_\_unidentified\_Clostridiales |
| 0.000834016567244 | 0.08% | k\_\_Bacteria;p\_\_Firmicutes;c\_\_Clostridia;o\_\_Clostridiales;f\_\_Ruminococcaceae;g\_\_Faecalibacterium |
| 0.000735065449097 | 0.07% | k\_\_Bacteria;p\_\_Bacteroidetes;c\_\_Bacteroidia;o\_\_Bacteroidales;f\_\_Prevotellaceae;g\_\_unidentified\_Prevotellaceae |
| 0.000720929575076 | 0.07% | k\_\_Bacteria;p\_\_Firmicutes;c\_\_Erysipelotrichia;o\_\_Erysipelotrichales;f\_\_Erysipelotrichaceae;Other |
| 0.000720929575076 | 0.07% | k\_\_Bacteria;p\_\_Bacteroidetes;c\_\_Bacteroidia;o\_\_Bacteroidales;f\_\_Marinifilaceae;g\_\_Butyricimonas |
| 0.00065025020497 | 0.07% | k\_\_Bacteria;p\_\_Firmicutes;c\_\_Clostridia;o\_\_Clostridiales;f\_\_unidentified\_Clostridiales;g\_\_Anaerovorax |
| 0.000607842582907 | 0.06% | k\_\_Bacteria;p\_\_Firmicutes;c\_\_Clostridia;o\_\_Clostridiales;f\_\_unidentified\_Clostridiales;Other |
| 0.000593706708886 | 0.06% | k\_\_Bacteria;p\_\_Firmicutes;Other;Other;Other;Other |
| 0.000452347968675 | 0.05% | k\_\_Bacteria;p\_\_unidentified\_Bacteria;c\_\_unidentified\_Bacteria;o\_\_unidentified\_Bacteria;f\_\_unidentified\_Bacteria;g\_\_Candidatus\_Saccharimonas |
| 0.000395804472591 | 0.04% | k\_\_Bacteria;p\_\_Verrucomicrobia;c\_\_Verrucomicrobiae;o\_\_Verrucomicrobiales;f\_\_Akkermansiaceae;g\_\_Akkermansia |
| 0.000381668598569 | 0.04% | k\_\_Bacteria;p\_\_Firmicutes;c\_\_Clostridia;o\_\_Clostridiales;f\_\_Peptococcaceae;g\_\_ |
| 0.000339260976506 | 0.03% | k\_\_Bacteria;p\_\_Firmicutes;c\_\_Negativicutes;o\_\_Selenomonadales;f\_\_Veillonellaceae;g\_\_Dialister |
| 0.000339260976506 | 0.03% | k\_\_Bacteria;p\_\_Firmicutes;c\_\_Clostridia;o\_\_Clostridiales;f\_\_Lachnospiraceae;g\_\_Fusicatenibacter |
| 0.000268581606401 | 0.03% | k\_\_Bacteria;p\_\_Fusobacteria;c\_\_Fusobacteriia;o\_\_Fusobacteriales;f\_\_Fusobacteriaceae;g\_\_Fusobacterium |
| 0.000268581606401 | 0.03% | k\_\_Bacteria;Other;Other;Other;Other;Other |
| 0.000240309858359 | 0.02% | k\_\_Bacteria;p\_\_Firmicutes;c\_\_Erysipelotrichia;o\_\_Erysipelotrichales;f\_\_Erysipelotrichaceae;g\_\_Dubosiella |
| 0.000197902236295 | 0.02% | k\_\_Bacteria;p\_\_Firmicutes;c\_\_Clostridia;o\_\_Clostridiales;f\_\_Ruminococcaceae;g\_\_Subdoligranulum |
| 0.000169630488253 | 0.02% | k\_\_Bacteria;p\_\_Actinobacteria;c\_\_unidentified\_Actinobacteria;o\_\_Bifidobacteriales;f\_\_Bifidobacteriaceae;g\_\_Bifidobacterium |
| 0.000155494614232 | 0.02% | k\_\_Bacteria;p\_\_Firmicutes;c\_\_Clostridia;o\_\_Clostridiales;f\_\_Lachnospiraceae;g\_\_Marvinbryantia |
| 0.000155494614232 | 0.02% | k\_\_Bacteria;p\_\_Firmicutes;c\_\_Bacilli;o\_\_Lactobacillales;f\_\_Streptococcaceae;g\_\_Streptococcus |
| 0.000141358740211 | 0.01% | k\_\_Bacteria;p\_\_Firmicutes;c\_\_Erysipelotrichia;o\_\_Erysipelotrichales;f\_\_Erysipelotrichaceae;g\_\_unidentified\_Erysipelotrichaceae |
| 0.000141358740211 | 0.01% | k\_\_Bacteria;p\_\_Firmicutes;c\_\_Clostridia;o\_\_Clostridiales;f\_\_Lachnospiraceae;g\_\_Anaerostipes |
| 0.00012722286619 | 0.01% | k\_\_Bacteria;p\_\_Firmicutes;c\_\_Negativicutes;o\_\_Selenomonadales;f\_\_Veillonellaceae;g\_\_Megamonas |
| 0.00012722286619 | 0.01% | k\_\_Bacteria;p\_\_Firmicutes;c\_\_Negativicutes;o\_\_Selenomonadales;f\_\_Acidaminococcaceae;g\_\_Phascolarctobacterium |
| 9.89511181476e-05 | 0.01% | k\_\_Bacteria;p\_\_Proteobacteria;c\_\_Gammaproteobacteria;o\_\_unidentified\_Gammaproteobacteria;f\_\_Burkholderiaceae;g\_\_Parasutterella |
| 9.89511181476e-05 | 0.01% | k\_\_Bacteria;p\_\_Bacteroidetes;c\_\_Bacteroidia;o\_\_Bacteroidales;f\_\_Porphyromonadaceae;g\_\_Porphyromonas |
| 8.48152441265e-05 | 0.01% | k\_\_Bacteria;p\_\_Proteobacteria;c\_\_Gammaproteobacteria;o\_\_unidentified\_Gammaproteobacteria;f\_\_Burkholderiaceae;g\_\_Sutterella |
| 8.48152441265e-05 | 0.01% | k\_\_Bacteria;p\_\_Proteobacteria;c\_\_Gammaproteobacteria;o\_\_Xanthomonadales;f\_\_Xanthomonadaceae;g\_\_Stenotrophomonas |
| 8.48152441265e-05 | 0.01% | k\_\_Bacteria;p\_\_Proteobacteria;c\_\_Deltaproteobacteria;o\_\_Desulfovibrionales;f\_\_Desulfovibrionaceae;g\_\_ |
| 8.48152441265e-05 | 0.01% | k\_\_Bacteria;p\_\_Melainabacteria;Other;Other;Other;Other |
| 8.48152441265e-05 | 0.01% | k\_\_Bacteria;p\_\_Firmicutes;c\_\_Negativicutes;o\_\_Selenomonadales;f\_\_Veillonellaceae;g\_\_Megasphaera |
| 8.48152441265e-05 | 0.01% | k\_\_Bacteria;p\_\_Firmicutes;c\_\_Clostridia;o\_\_Clostridiales;f\_\_Ruminococcaceae;g\_\_Flavonifractor |
| 8.48152441265e-05 | 0.01% | k\_\_Bacteria;p\_\_Firmicutes;c\_\_Clostridia;o\_\_Clostridiales;f\_\_Eubacteriaceae;g\_\_Anaerofustis |
| 8.48152441265e-05 | 0.01% | k\_\_Bacteria;p\_\_Bacteroidetes;c\_\_Bacteroidia;o\_\_Bacteroidales;f\_\_Marinifilaceae;g\_\_Odoribacter |
| 7.06793701055e-05 | 0.01% | k\_\_Bacteria;p\_\_Proteobacteria;c\_\_Gammaproteobacteria;o\_\_unidentified\_Gammaproteobacteria;f\_\_Burkholderiaceae;g\_\_Diaphorobacter |
| 7.06793701055e-05 | 0.01% | k\_\_Bacteria;p\_\_Proteobacteria;c\_\_Alphaproteobacteria;o\_\_Sphingomonadales;f\_\_Sphingomonadaceae;g\_\_Sphingomonas |
| 7.06793701055e-05 | 0.01% | k\_\_Bacteria;p\_\_Firmicutes;c\_\_Clostridia;o\_\_Clostridiales;f\_\_Peptostreptococcaceae;g\_\_Romboutsia |
| 7.06793701055e-05 | 0.01% | k\_\_Bacteria;p\_\_Bacteroidetes;c\_\_Bacteroidia;o\_\_Bacteroidales;f\_\_Prevotellaceae;g\_\_Prevotella |
| 5.65434960844e-05 | 0.01% | k\_\_Bacteria;p\_\_unidentified\_Bacteria;c\_\_unidentified\_Bacteria;o\_\_Campylobacterales;f\_\_unidentified\_Campylobacterales;g\_\_Sulfurospirillum |
| 5.65434960844e-05 | 0.01% | k\_\_Bacteria;p\_\_Proteobacteria;c\_\_Gammaproteobacteria;o\_\_unidentified\_Gammaproteobacteria;f\_\_Burkholderiaceae;g\_\_Delftia |
| 5.65434960844e-05 | 0.01% | k\_\_Bacteria;p\_\_Firmicutes;c\_\_Erysipelotrichia;o\_\_Erysipelotrichales;f\_\_Erysipelotrichaceae;g\_\_Erysipelatoclostridium |
| 4.24076220633e-05 | 0.00% | k\_\_Bacteria;p\_\_Proteobacteria;c\_\_Gammaproteobacteria;o\_\_Xanthomonadales;f\_\_Xanthomonadaceae;g\_\_Arenimonas |
| 4.24076220633e-05 | 0.00% | k\_\_Bacteria;p\_\_Proteobacteria;c\_\_Gammaproteobacteria;o\_\_Pseudomonadales;f\_\_Moraxellaceae;g\_\_Acinetobacter |
| 4.24076220633e-05 | 0.00% | k\_\_Bacteria;p\_\_Proteobacteria;Other;Other;Other;Other |
| 4.24076220633e-05 | 0.00% | k\_\_Bacteria;p\_\_Firmicutes;c\_\_Negativicutes;o\_\_Selenomonadales;f\_\_Veillonellaceae;g\_\_Veillonella |
| 4.24076220633e-05 | 0.00% | k\_\_Bacteria;p\_\_Firmicutes;c\_\_Clostridia;o\_\_Clostridiales;f\_\_Peptostreptococcaceae;g\_\_Clostridioides |
| 4.24076220633e-05 | 0.00% | k\_\_Bacteria;p\_\_Bacteroidetes;c\_\_Bacteroidia;o\_\_Bacteroidales;f\_\_Barnesiellaceae;g\_\_Coprobacter |
| 2.82717480422e-05 | 0.00% | k\_\_Bacteria;p\_\_Proteobacteria;c\_\_Gammaproteobacteria;o\_\_unidentified\_Gammaproteobacteria;f\_\_Neisseriaceae;g\_\_Neisseria |
| 2.82717480422e-05 | 0.00% | k\_\_Bacteria;p\_\_Proteobacteria;c\_\_Gammaproteobacteria;o\_\_Pseudomonadales;f\_\_Pseudomonadaceae;g\_\_Pseudomonas |
| 2.82717480422e-05 | 0.00% | k\_\_Bacteria;p\_\_Proteobacteria;c\_\_Deltaproteobacteria;o\_\_Desulfovibrionales;f\_\_Desulfovibrionaceae;g\_\_Bilophila |
| 2.82717480422e-05 | 0.00% | k\_\_Bacteria;p\_\_Firmicutes;c\_\_Clostridia;o\_\_Clostridiales;f\_\_unidentified\_Clostridiales;g\_\_Parvimonas |
| 2.82717480422e-05 | 0.00% | k\_\_Bacteria;p\_\_Bacteroidetes;c\_\_Bacteroidia;o\_\_Flavobacteriales;f\_\_Flavobacteriaceae;g\_\_Tenacibaculum |
| 1.41358740211e-05 | 0.00% | k\_\_Bacteria;p\_\_Proteobacteria;c\_\_Gammaproteobacteria;o\_\_unidentified\_Gammaproteobacteria;f\_\_Burkholderiaceae;g\_\_Ralstonia |
| 1.41358740211e-05 | 0.00% | k\_\_Bacteria;p\_\_Proteobacteria;c\_\_Gammaproteobacteria;o\_\_unidentified\_Gammaproteobacteria;f\_\_Burkholderiaceae;g\_\_Achromobacter |
| 1.41358740211e-05 | 0.00% | k\_\_Bacteria;p\_\_Proteobacteria;c\_\_Gammaproteobacteria;o\_\_unidentified\_Gammaproteobacteria;f\_\_Burkholderiaceae;Other |
| 1.41358740211e-05 | 0.00% | k\_\_Bacteria;p\_\_Proteobacteria;c\_\_Gammaproteobacteria;o\_\_Pasteurellales;f\_\_Pasteurellaceae;Other |
| 1.41358740211e-05 | 0.00% | k\_\_Bacteria;p\_\_Proteobacteria;c\_\_Alphaproteobacteria;o\_\_Rhodospirillales;f\_\_unidentified\_Rhodospirillales;g\_\_Telmatospirillum |
| 1.41358740211e-05 | 0.00% | k\_\_Bacteria;p\_\_Proteobacteria;c\_\_Alphaproteobacteria;o\_\_Rhizobiales;f\_\_Xanthobacteraceae;g\_\_Bradyrhizobium |
| 1.41358740211e-05 | 0.00% | k\_\_Bacteria;p\_\_Proteobacteria;c\_\_Alphaproteobacteria;o\_\_Rhizobiales;f\_\_Rhizobiaceae;g\_\_Phyllobacterium |
| 1.41358740211e-05 | 0.00% | k\_\_Bacteria;p\_\_Proteobacteria;c\_\_Alphaproteobacteria;o\_\_Rhizobiales;f\_\_Beijerinckiaceae;g\_\_Methylobacterium |
| 1.41358740211e-05 | 0.00% | k\_\_Bacteria;p\_\_Oxyphotobacteria;c\_\_unidentified\_Oxyphotobacteria;o\_\_unidentified\_Oxyphotobacteria;f\_\_unidentified\_Oxyphotobacteria;g\_\_unidentified\_Oxyphotobacteria |
| 1.41358740211e-05 | 0.00% | k\_\_Bacteria;p\_\_Firmicutes;c\_\_Negativicutes;o\_\_Selenomonadales;f\_\_Veillonellaceae;g\_\_Allisonella |
| 1.41358740211e-05 | 0.00% | k\_\_Bacteria;p\_\_Firmicutes;c\_\_Erysipelotrichia;o\_\_Erysipelotrichales;f\_\_Erysipelotrichaceae;g\_\_Turicibacter |
| 1.41358740211e-05 | 0.00% | k\_\_Bacteria;p\_\_Firmicutes;c\_\_Clostridia;o\_\_Clostridiales;f\_\_Peptococcaceae;g\_\_Peptococcus |
| 1.41358740211e-05 | 0.00% | k\_\_Bacteria;p\_\_Firmicutes;c\_\_Bacilli;o\_\_Bacillales;f\_\_unidentified\_Bacillales;g\_\_Exiguobacterium |
| 1.41358740211e-05 | 0.00% | k\_\_Bacteria;p\_\_Bacteroidetes;c\_\_Bacteroidia;o\_\_Bacteroidales;f\_\_Prevotellaceae;Other |
| 1.41358740211e-05 | 0.00% | k\_\_Bacteria;p\_\_Actinobacteria;c\_\_unidentified\_Actinobacteria;o\_\_Corynebacteriales;f\_\_Corynebacteriaceae;g\_\_unidentified\_Corynebacteriaceae |
| 1.41358740211e-05 | 0.00% | k\_\_Bacteria;p\_\_Actinobacteria;c\_\_Coriobacteriia;o\_\_Coriobacteriales;f\_\_Atopobiaceae;g\_\_Atopobium |

|  |  |
| --- | --- |
|  | |
| Taxonomy Summary. Current Level: \_MC1 | |
| View Figure (.pdf)  View Legend (.pdf) |  |
|  |  |
| View Figure (.pdf)  View Legend (.pdf) |  |
|  |  |
|  |  |

|  |  |  |
| --- | --- | --- |
| Count | Pct | Taxonomy |
| 0.349947697266 | 34.99% | k\_\_Bacteria;p\_\_Bacteroidetes;c\_\_Bacteroidia;o\_\_Bacteroidales;f\_\_Muribaculaceae;g\_\_ |
| 0.155155353255 | 15.52% | k\_\_Bacteria;p\_\_Bacteroidetes;c\_\_Bacteroidia;o\_\_Bacteroidales;f\_\_Bacteroidaceae;g\_\_Bacteroides |
| 0.0867801306155 | 8.68% | k\_\_Bacteria;p\_\_Verrucomicrobia;c\_\_Verrucomicrobiae;o\_\_Verrucomicrobiales;f\_\_Akkermansiaceae;g\_\_Akkermansia |
| 0.0806168895423 | 8.06% | k\_\_Bacteria;p\_\_Firmicutes;c\_\_Clostridia;o\_\_Clostridiales;f\_\_Ruminococcaceae;Other |
| 0.0786802748014 | 7.87% | k\_\_Bacteria;p\_\_Firmicutes;c\_\_Clostridia;o\_\_Clostridiales;f\_\_Lachnospiraceae;Other |
| 0.0446128184106 | 4.46% | k\_\_Bacteria;p\_\_Firmicutes;c\_\_Clostridia;o\_\_Clostridiales;f\_\_Ruminococcaceae;g\_\_unidentified\_Ruminococcaceae |
| 0.0251901275056 | 2.52% | k\_\_Bacteria;p\_\_Tenericutes;c\_\_Mollicutes;o\_\_;f\_\_;g\_\_ |
| 0.024144072828 | 2.41% | k\_\_Bacteria;p\_\_Firmicutes;c\_\_Clostridia;o\_\_Clostridiales;f\_\_Lachnospiraceae;g\_\_unidentified\_Lachnospiraceae |
| 0.0200163976139 | 2.00% | k\_\_Bacteria;p\_\_Bacteroidetes;c\_\_Bacteroidia;o\_\_Bacteroidales;f\_\_Muribaculaceae;g\_\_Muribaculum |
| 0.0177546577705 | 1.78% | k\_\_Bacteria;p\_\_Bacteroidetes;c\_\_Bacteroidia;o\_\_Bacteroidales;f\_\_Rikenellaceae;g\_\_Alistipes |
| 0.0166520595968 | 1.67% | k\_\_Bacteria;p\_\_Proteobacteria;c\_\_Gammaproteobacteria;o\_\_Enterobacteriales;f\_\_Enterobacteriaceae;g\_\_unidentified\_Enterobacteriaceae |
| 0.0117469113115 | 1.17% | k\_\_Bacteria;p\_\_Proteobacteria;c\_\_Gammaproteobacteria;o\_\_Enterobacteriales;f\_\_Enterobacteriaceae;Other |
| 0.00969720957847 | 0.97% | k\_\_Bacteria;p\_\_Firmicutes;c\_\_Clostridia;o\_\_Clostridiales;f\_\_Lachnospiraceae;g\_\_Lachnoclostridium |
| 0.0073082468689 | 0.73% | k\_\_Bacteria;p\_\_Firmicutes;c\_\_Erysipelotrichia;o\_\_Erysipelotrichales;f\_\_Erysipelotrichaceae;g\_\_Dubosiella |
| 0.00702552938848 | 0.70% | k\_\_Bacteria;p\_\_Bacteroidetes;c\_\_Bacteroidia;o\_\_Bacteroidales;f\_\_Tannerellaceae;g\_\_Parabacteroides |
| 0.00588052359277 | 0.59% | k\_\_Bacteria;p\_\_Bacteroidetes;c\_\_Bacteroidia;o\_\_Bacteroidales;f\_\_Prevotellaceae;g\_\_Alloprevotella |
| 0.00539990387606 | 0.54% | k\_\_Bacteria;p\_\_Bacteroidetes;c\_\_Bacteroidia;o\_\_Bacteroidales;f\_\_Prevotellaceae;Other |
| 0.00538576800204 | 0.54% | k\_\_Bacteria;p\_\_Firmicutes;c\_\_Clostridia;o\_\_Clostridiales;f\_\_Lachnospiraceae;g\_\_Blautia |
| 0.00453761556077 | 0.45% | k\_\_Bacteria;p\_\_Proteobacteria;c\_\_Deltaproteobacteria;o\_\_Desulfovibrionales;f\_\_Desulfovibrionaceae;g\_\_Desulfovibrio |
| 0.00432557745045 | 0.43% | k\_\_Bacteria;p\_\_Firmicutes;c\_\_Bacilli;o\_\_Lactobacillales;f\_\_Lactobacillaceae;g\_\_Lactobacillus |
| 0.00405699584405 | 0.41% | k\_\_Bacteria;p\_\_Firmicutes;c\_\_Clostridia;o\_\_Clostridiales;f\_\_Ruminococcaceae;g\_\_Oscillibacter |
| 0.00360464787538 | 0.36% | k\_\_Bacteria;p\_\_Firmicutes;c\_\_Clostridia;o\_\_Clostridiales;f\_\_Lachnospiraceae;g\_\_Roseburia |
| 0.00292612592237 | 0.29% | k\_\_Bacteria;p\_\_Firmicutes;c\_\_Erysipelotrichia;o\_\_Erysipelotrichales;f\_\_Erysipelotrichaceae;Other |
| 0.00286958242628 | 0.29% | k\_\_Bacteria;p\_\_Firmicutes;c\_\_Clostridia;o\_\_Clostridiales;Other;Other |
| 0.00278476718215 | 0.28% | k\_\_Bacteria;p\_\_Firmicutes;c\_\_Bacilli;o\_\_Lactobacillales;f\_\_Enterococcaceae;g\_\_Enterococcus |
| 0.00250204970173 | 0.25% | k\_\_Bacteria;p\_\_Firmicutes;c\_\_Clostridia;o\_\_Clostridiales;f\_\_Ruminococcaceae;g\_\_Ruminiclostridium |
| 0.00192247886687 | 0.19% | k\_\_Bacteria;p\_\_Firmicutes;c\_\_Clostridia;o\_\_Clostridiales;f\_\_Lachnospiraceae;g\_\_ |
| 0.00166803313449 | 0.17% | k\_\_Bacteria;p\_\_Melainabacteria;Other;Other;Other;Other |
| 0.00131463628396 | 0.13% | k\_\_Bacteria;p\_\_Firmicutes;c\_\_Clostridia;o\_\_Clostridiales;f\_\_unidentified\_Clostridiales;g\_\_unidentified\_Clostridiales |
| 0.00128636453592 | 0.13% | k\_\_Bacteria;p\_\_Bacteroidetes;c\_\_Bacteroidia;o\_\_Bacteroidales;f\_\_Marinifilaceae;g\_\_Butyricimonas |
| 0.0012722286619 | 0.13% | k\_\_Bacteria;Other;Other;Other;Other;Other |
| 0.00111673404767 | 0.11% | k\_\_Bacteria;p\_\_Firmicutes;c\_\_Clostridia;o\_\_Clostridiales;f\_\_Ruminococcaceae;g\_\_Intestinimonas |
| 0.00104605467756 | 0.10% | k\_\_Bacteria;p\_\_Oxyphotobacteria;c\_\_unidentified\_Oxyphotobacteria;o\_\_unidentified\_Oxyphotobacteria;f\_\_unidentified\_Oxyphotobacteria;g\_\_unidentified\_Oxyphotobacteria |
| 0.00101778292952 | 0.10% | k\_\_Bacteria;p\_\_Firmicutes;c\_\_Clostridia;o\_\_Clostridiales;f\_\_Ruminococcaceae;g\_\_Butyricicoccus |
| 0.000975375307455 | 0.10% | k\_\_Bacteria;p\_\_Firmicutes;c\_\_Clostridia;o\_\_Clostridiales;f\_\_Ruminococcaceae;g\_\_Anaerotruncus |
| 0.000819880693223 | 0.08% | k\_\_Bacteria;p\_\_Firmicutes;c\_\_Clostridia;o\_\_Clostridiales;f\_\_Ruminococcaceae;g\_\_Faecalibacterium |
| 0.000678521953012 | 0.07% | k\_\_Bacteria;p\_\_Proteobacteria;c\_\_Gammaproteobacteria;o\_\_Enterobacteriales;f\_\_Enterobacteriaceae;g\_\_Proteus |
| 0.000593706708886 | 0.06% | k\_\_Bacteria;p\_\_unidentified\_Bacteria;c\_\_unidentified\_Bacteria;o\_\_unidentified\_Bacteria;f\_\_unidentified\_Bacteria;g\_\_Candidatus\_Saccharimonas |
| 0.000579570834865 | 0.06% | k\_\_Bacteria;p\_\_Bacteroidetes;c\_\_Bacteroidia;o\_\_Bacteroidales;f\_\_Prevotellaceae;g\_\_unidentified\_Prevotellaceae |
| 0.000537163212801 | 0.05% | k\_\_Bacteria;p\_\_Firmicutes;c\_\_Clostridia;o\_\_Clostridiales;f\_\_Lachnospiraceae;g\_\_Acetatifactor |
| 0.000480619716717 | 0.05% | k\_\_Bacteria;p\_\_Actinobacteria;c\_\_unidentified\_Actinobacteria;o\_\_Bifidobacteriales;f\_\_Bifidobacteriaceae;g\_\_Bifidobacterium |
| 0.000395804472591 | 0.04% | k\_\_Bacteria;p\_\_Fusobacteria;c\_\_Fusobacteriia;o\_\_Fusobacteriales;f\_\_Fusobacteriaceae;g\_\_Fusobacterium |
| 0.000282717480422 | 0.03% | k\_\_Bacteria;p\_\_Firmicutes;c\_\_Clostridia;o\_\_Clostridiales;f\_\_Lachnospiraceae;g\_\_Fusicatenibacter |
| 0.00025444573238 | 0.03% | k\_\_Bacteria;p\_\_Firmicutes;c\_\_Clostridia;o\_\_Clostridiales;f\_\_Ruminococcaceae;g\_\_Harryflintia |
| 0.000240309858359 | 0.02% | k\_\_Bacteria;p\_\_Firmicutes;c\_\_Clostridia;o\_\_Clostridiales;f\_\_Lachnospiraceae;g\_\_Anaerostipes |
| 0.000226173984337 | 0.02% | k\_\_Bacteria;p\_\_Firmicutes;c\_\_Negativicutes;o\_\_Selenomonadales;f\_\_Veillonellaceae;g\_\_Dialister |
| 0.000197902236295 | 0.02% | k\_\_Bacteria;p\_\_Proteobacteria;c\_\_Gammaproteobacteria;o\_\_Pseudomonadales;f\_\_Moraxellaceae;g\_\_Acinetobacter |
| 0.000197902236295 | 0.02% | k\_\_Bacteria;p\_\_Firmicutes;c\_\_Clostridia;o\_\_Clostridiales;f\_\_Peptococcaceae;g\_\_ |
| 0.000183766362274 | 0.02% | k\_\_Bacteria;p\_\_Firmicutes;Other;Other;Other;Other |
| 0.000169630488253 | 0.02% | k\_\_Bacteria;p\_\_Firmicutes;c\_\_Clostridia;o\_\_Clostridiales;f\_\_Ruminococcaceae;g\_\_Subdoligranulum |
| 0.000169630488253 | 0.02% | k\_\_Bacteria;p\_\_Firmicutes;c\_\_Clostridia;o\_\_Clostridiales;f\_\_Lachnospiraceae;g\_\_Tyzzerella |
| 0.000155494614232 | 0.02% | k\_\_Bacteria;p\_\_Firmicutes;c\_\_Clostridia;o\_\_Clostridiales;f\_\_Ruminococcaceae;g\_\_Caproiciproducens |
| 0.000141358740211 | 0.01% | k\_\_Bacteria;p\_\_Proteobacteria;c\_\_Gammaproteobacteria;o\_\_unidentified\_Gammaproteobacteria;f\_\_Burkholderiaceae;g\_\_Sutterella |
| 0.000113086992169 | 0.01% | k\_\_Bacteria;p\_\_Proteobacteria;c\_\_Gammaproteobacteria;o\_\_Pseudomonadales;f\_\_Pseudomonadaceae;g\_\_Pseudomonas |
| 0.000113086992169 | 0.01% | k\_\_Bacteria;p\_\_Bacteroidetes;c\_\_Bacteroidia;o\_\_Bacteroidales;f\_\_Barnesiellaceae;g\_\_Barnesiella |
| 9.89511181476e-05 | 0.01% | k\_\_Bacteria;p\_\_Firmicutes;c\_\_Clostridia;o\_\_Clostridiales;f\_\_Ruminococcaceae;g\_\_Flavonifractor |
| 9.89511181476e-05 | 0.01% | k\_\_Bacteria;p\_\_Firmicutes;c\_\_Clostridia;o\_\_Clostridiales;f\_\_Peptostreptococcaceae;g\_\_Clostridioides |
| 9.89511181476e-05 | 0.01% | k\_\_Bacteria;p\_\_Bacteroidetes;c\_\_Bacteroidia;o\_\_Bacteroidales;f\_\_Prevotellaceae;g\_\_Prevotella |
| 9.89511181476e-05 | 0.01% | k\_\_Bacteria;p\_\_Bacteroidetes;c\_\_Bacteroidia;o\_\_Bacteroidales;f\_\_Porphyromonadaceae;g\_\_Porphyromonas |
| 9.89511181476e-05 | 0.01% | k\_\_Bacteria;p\_\_Bacteroidetes;c\_\_Bacteroidia;o\_\_Bacteroidales;f\_\_Marinifilaceae;g\_\_Odoribacter |
| 8.48152441265e-05 | 0.01% | k\_\_Bacteria;p\_\_Proteobacteria;c\_\_Gammaproteobacteria;o\_\_unidentified\_Gammaproteobacteria;f\_\_Burkholderiaceae;g\_\_Parasutterella |
| 8.48152441265e-05 | 0.01% | k\_\_Bacteria;p\_\_Firmicutes;c\_\_Erysipelotrichia;o\_\_Erysipelotrichales;f\_\_Erysipelotrichaceae;g\_\_unidentified\_Erysipelotrichaceae |
| 8.48152441265e-05 | 0.01% | k\_\_Bacteria;p\_\_Firmicutes;c\_\_Clostridia;o\_\_Clostridiales;f\_\_unidentified\_Clostridiales;Other |
| 8.48152441265e-05 | 0.01% | k\_\_Bacteria;p\_\_Firmicutes;c\_\_Clostridia;o\_\_Clostridiales;f\_\_Peptostreptococcaceae;g\_\_Romboutsia |
| 8.48152441265e-05 | 0.01% | k\_\_Bacteria;p\_\_Firmicutes;c\_\_Clostridia;o\_\_Clostridiales;f\_\_Lachnospiraceae;g\_\_Marvinbryantia |
| 8.48152441265e-05 | 0.01% | k\_\_Bacteria;p\_\_Firmicutes;c\_\_Clostridia;o\_\_Clostridiales;f\_\_Lachnospiraceae;g\_\_Lachnospira |
| 7.06793701055e-05 | 0.01% | k\_\_Bacteria;p\_\_Proteobacteria;c\_\_Gammaproteobacteria;o\_\_unidentified\_Gammaproteobacteria;f\_\_Burkholderiaceae;g\_\_Diaphorobacter |
| 7.06793701055e-05 | 0.01% | k\_\_Bacteria;p\_\_Firmicutes;c\_\_Bacilli;o\_\_Lactobacillales;f\_\_Streptococcaceae;g\_\_Streptococcus |
| 7.06793701055e-05 | 0.01% | k\_\_Bacteria;p\_\_Bacteroidetes;c\_\_Bacteroidia;o\_\_Flavobacteriales;f\_\_unidentified\_Flavobacteriales;g\_\_Chryseobacterium |
| 5.65434960844e-05 | 0.01% | k\_\_Bacteria;p\_\_unidentified\_Bacteria;c\_\_unidentified\_Bacteria;o\_\_Campylobacterales;f\_\_unidentified\_Campylobacterales;g\_\_Sulfurospirillum |
| 5.65434960844e-05 | 0.01% | k\_\_Bacteria;p\_\_Proteobacteria;c\_\_Alphaproteobacteria;o\_\_Sphingomonadales;f\_\_Sphingomonadaceae;g\_\_Sphingomonas |
| 5.65434960844e-05 | 0.01% | k\_\_Bacteria;p\_\_Firmicutes;c\_\_Negativicutes;o\_\_Selenomonadales;f\_\_Acidaminococcaceae;g\_\_Phascolarctobacterium |
| 5.65434960844e-05 | 0.01% | k\_\_Bacteria;p\_\_Firmicutes;c\_\_Negativicutes;o\_\_Selenomonadales;f\_\_Acidaminococcaceae;g\_\_Acidaminococcus |
| 5.65434960844e-05 | 0.01% | k\_\_Bacteria;p\_\_Firmicutes;c\_\_Clostridia;o\_\_Clostridiales;f\_\_unidentified\_Clostridiales;g\_\_Anaerovorax |
| 5.65434960844e-05 | 0.01% | k\_\_Bacteria;p\_\_Firmicutes;c\_\_Clostridia;o\_\_Clostridiales;f\_\_Peptococcaceae;g\_\_Peptococcus |
| 5.65434960844e-05 | 0.01% | k\_\_Bacteria;p\_\_Firmicutes;c\_\_Clostridia;o\_\_Clostridiales;f\_\_Eubacteriaceae;g\_\_Anaerofustis |
| 4.24076220633e-05 | 0.00% | k\_\_Bacteria;p\_\_Proteobacteria;c\_\_Gammaproteobacteria;o\_\_unidentified\_Gammaproteobacteria;f\_\_Burkholderiaceae;g\_\_Delftia |
| 4.24076220633e-05 | 0.00% | k\_\_Bacteria;p\_\_Proteobacteria;c\_\_Gammaproteobacteria;o\_\_unidentified\_Gammaproteobacteria;f\_\_Burkholderiaceae;g\_\_Achromobacter |
| 4.24076220633e-05 | 0.00% | k\_\_Bacteria;p\_\_Proteobacteria;c\_\_Gammaproteobacteria;o\_\_Pasteurellales;f\_\_Pasteurellaceae;Other |
| 4.24076220633e-05 | 0.00% | k\_\_Bacteria;p\_\_Proteobacteria;c\_\_Alphaproteobacteria;o\_\_Rhizobiales;f\_\_Rhizobiaceae;g\_\_Phyllobacterium |
| 4.24076220633e-05 | 0.00% | k\_\_Bacteria;p\_\_Firmicutes;c\_\_Negativicutes;o\_\_Selenomonadales;f\_\_Veillonellaceae;g\_\_Veillonella |
| 4.24076220633e-05 | 0.00% | k\_\_Bacteria;p\_\_Firmicutes;c\_\_Bacilli;o\_\_Bacillales;f\_\_unidentified\_Bacillales;g\_\_Exiguobacterium |
| 4.24076220633e-05 | 0.00% | k\_\_Bacteria;p\_\_Actinobacteria;c\_\_unidentified\_Actinobacteria;o\_\_Corynebacteriales;f\_\_Nocardiaceae;g\_\_Rhodococcus |
| 2.82717480422e-05 | 0.00% | k\_\_Bacteria;p\_\_Verrucomicrobia;c\_\_Verrucomicrobiae;o\_\_Chthoniobacterales;f\_\_Chthoniobacteraceae;Other |
| 2.82717480422e-05 | 0.00% | k\_\_Bacteria;p\_\_Proteobacteria;c\_\_Gammaproteobacteria;o\_\_unidentified\_Gammaproteobacteria;f\_\_Burkholderiaceae;Other |
| 2.82717480422e-05 | 0.00% | k\_\_Bacteria;p\_\_Proteobacteria;c\_\_Gammaproteobacteria;o\_\_Alteromonadales;f\_\_Shewanellaceae;g\_\_Shewanella |
| 2.82717480422e-05 | 0.00% | k\_\_Bacteria;p\_\_Proteobacteria;c\_\_Alphaproteobacteria;o\_\_Rhodospirillales;f\_\_unidentified\_Rhodospirillales;g\_\_Telmatospirillum |
| 2.82717480422e-05 | 0.00% | k\_\_Bacteria;p\_\_Proteobacteria;c\_\_Alphaproteobacteria;o\_\_Rhizobiales;f\_\_Xanthobacteraceae;g\_\_Bradyrhizobium |
| 2.82717480422e-05 | 0.00% | k\_\_Bacteria;p\_\_Firmicutes;c\_\_Erysipelotrichia;o\_\_Erysipelotrichales;f\_\_Erysipelotrichaceae;g\_\_Holdemania |
| 2.82717480422e-05 | 0.00% | k\_\_Bacteria;p\_\_Firmicutes;c\_\_Clostridia;o\_\_Clostridiales;f\_\_Lachnospiraceae;g\_\_Agathobacter |
| 2.82717480422e-05 | 0.00% | k\_\_Bacteria;p\_\_Bacteroidetes;c\_\_Bacteroidia;o\_\_Bacteroidales;f\_\_Prevotellaceae;g\_\_ |
| 2.82717480422e-05 | 0.00% | k\_\_Bacteria;p\_\_Armatimonadetes;c\_\_Fimbriimonadia;o\_\_Fimbriimonadales;f\_\_Fimbriimonadaceae;g\_\_ |
| 1.41358740211e-05 | 0.00% | k\_\_Bacteria;p\_\_Proteobacteria;c\_\_Alphaproteobacteria;o\_\_Rhizobiales;f\_\_Beijerinckiaceae;g\_\_Methylobacterium |
| 1.41358740211e-05 | 0.00% | k\_\_Bacteria;p\_\_Firmicutes;c\_\_Negativicutes;o\_\_Selenomonadales;f\_\_Veillonellaceae;g\_\_Megasphaera |
| 1.41358740211e-05 | 0.00% | k\_\_Bacteria;p\_\_Firmicutes;c\_\_Negativicutes;o\_\_Selenomonadales;f\_\_Veillonellaceae;g\_\_Megamonas |
| 1.41358740211e-05 | 0.00% | k\_\_Bacteria;p\_\_Firmicutes;c\_\_Bacilli;o\_\_Bacillales;f\_\_Staphylococcaceae;g\_\_Staphylococcus |
| 1.41358740211e-05 | 0.00% | k\_\_Bacteria;p\_\_Bacteroidetes;c\_\_Bacteroidia;o\_\_Flavobacteriales;f\_\_Flavobacteriaceae;g\_\_Tenacibaculum |
| 1.41358740211e-05 | 0.00% | k\_\_Bacteria;p\_\_Actinobacteria;c\_\_unidentified\_Actinobacteria;o\_\_Actinomycetales;f\_\_Actinomycetaceae;g\_\_Actinomyces |

|  |  |
| --- | --- |
|  | |
| Taxonomy Summary. Current Level: \_MC2 | |
| View Figure (.pdf)  View Legend (.pdf) |  |
|  |  |
| View Figure (.pdf)  View Legend (.pdf) |  |
|  |  |
|  |  |

|  |  |  |
| --- | --- | --- |
| Count | Pct | Taxonomy |
| 0.296895762065 | 29.69% | k\_\_Bacteria;p\_\_Bacteroidetes;c\_\_Bacteroidia;o\_\_Bacteroidales;f\_\_Muribaculaceae;g\_\_ |
| 0.270037601425 | 27.00% | k\_\_Bacteria;p\_\_Bacteroidetes;c\_\_Bacteroidia;o\_\_Bacteroidales;f\_\_Bacteroidaceae;g\_\_Bacteroides |
| 0.0709196799638 | 7.09% | k\_\_Bacteria;p\_\_Firmicutes;c\_\_Clostridia;o\_\_Clostridiales;f\_\_Lachnospiraceae;Other |
| 0.0633569873625 | 6.34% | k\_\_Bacteria;p\_\_Proteobacteria;c\_\_Gammaproteobacteria;o\_\_Enterobacteriales;f\_\_Enterobacteriaceae;g\_\_unidentified\_Enterobacteriaceae |
| 0.0569110288089 | 5.69% | k\_\_Bacteria;p\_\_Firmicutes;c\_\_Clostridia;o\_\_Clostridiales;f\_\_Ruminococcaceae;Other |
| 0.0401600180939 | 4.02% | k\_\_Bacteria;p\_\_Firmicutes;c\_\_Clostridia;o\_\_Clostridiales;f\_\_Ruminococcaceae;g\_\_unidentified\_Ruminococcaceae |
| 0.0250770405134 | 2.51% | k\_\_Bacteria;p\_\_Bacteroidetes;c\_\_Bacteroidia;o\_\_Bacteroidales;f\_\_Rikenellaceae;g\_\_Alistipes |
| 0.024144072828 | 2.41% | k\_\_Bacteria;p\_\_Firmicutes;c\_\_Clostridia;o\_\_Clostridiales;f\_\_Lachnospiraceae;g\_\_unidentified\_Lachnospiraceae |
| 0.0203273868423 | 2.03% | k\_\_Bacteria;p\_\_Bacteroidetes;c\_\_Bacteroidia;o\_\_Bacteroidales;f\_\_Prevotellaceae;g\_\_Alloprevotella |
| 0.0176415707783 | 1.76% | k\_\_Bacteria;p\_\_Bacteroidetes;c\_\_Bacteroidia;o\_\_Bacteroidales;f\_\_Prevotellaceae;Other |
| 0.0173588532979 | 1.74% | k\_\_Bacteria;p\_\_Proteobacteria;c\_\_Gammaproteobacteria;o\_\_Enterobacteriales;f\_\_Enterobacteriaceae;Other |
| 0.012708150745 | 1.27% | k\_\_Bacteria;p\_\_Proteobacteria;c\_\_Deltaproteobacteria;o\_\_Desulfovibrionales;f\_\_Desulfovibrionaceae;g\_\_Desulfovibrio |
| 0.0124678408866 | 1.25% | k\_\_Bacteria;p\_\_Verrucomicrobia;c\_\_Verrucomicrobiae;o\_\_Verrucomicrobiales;f\_\_Akkermansiaceae;g\_\_Akkermansia |
| 0.00941449209805 | 0.94% | k\_\_Bacteria;p\_\_Bacteroidetes;c\_\_Bacteroidia;o\_\_Bacteroidales;f\_\_Tannerellaceae;g\_\_Parabacteroides |
| 0.00934381272794 | 0.93% | k\_\_Bacteria;p\_\_Firmicutes;c\_\_Bacilli;o\_\_Lactobacillales;f\_\_Enterococcaceae;g\_\_Enterococcus |
| 0.00655904554579 | 0.66% | k\_\_Bacteria;p\_\_Firmicutes;c\_\_Clostridia;o\_\_Clostridiales;f\_\_Lachnospiraceae;g\_\_Lachnoclostridium |
| 0.00422662633231 | 0.42% | k\_\_Bacteria;p\_\_Firmicutes;c\_\_Bacilli;o\_\_Lactobacillales;f\_\_Lactobacillaceae;g\_\_Lactobacillus |
| 0.00407113171807 | 0.41% | k\_\_Bacteria;p\_\_Bacteroidetes;c\_\_Bacteroidia;o\_\_Bacteroidales;f\_\_Muribaculaceae;g\_\_Muribaculum |
| 0.00385909360776 | 0.39% | k\_\_Bacteria;p\_\_Tenericutes;c\_\_Mollicutes;o\_\_;f\_\_;g\_\_ |
| 0.00363291962342 | 0.36% | k\_\_Bacteria;p\_\_Firmicutes;c\_\_Erysipelotrichia;o\_\_Erysipelotrichales;f\_\_Erysipelotrichaceae;g\_\_Dubosiella |
| 0.0034208815131 | 0.34% | k\_\_Bacteria;p\_\_Firmicutes;c\_\_Clostridia;o\_\_Clostridiales;Other;Other |
| 0.00320884340279 | 0.32% | k\_\_Bacteria;p\_\_Firmicutes;c\_\_Clostridia;o\_\_Clostridiales;f\_\_Ruminococcaceae;g\_\_Oscillibacter |
| 0.00226173984337 | 0.23% | k\_\_Bacteria;p\_\_Firmicutes;c\_\_Clostridia;o\_\_Clostridiales;f\_\_Lachnospiraceae;g\_\_Blautia |
| 0.00221933222131 | 0.22% | k\_\_Bacteria;p\_\_Proteobacteria;c\_\_Gammaproteobacteria;o\_\_Enterobacteriales;f\_\_Enterobacteriaceae;g\_\_Proteus |
| 0.00203556585904 | 0.20% | k\_\_Bacteria;p\_\_Firmicutes;c\_\_Clostridia;o\_\_Clostridiales;f\_\_Lachnospiraceae;g\_\_ |
| 0.00183766362274 | 0.18% | k\_\_Bacteria;p\_\_Firmicutes;c\_\_Erysipelotrichia;o\_\_Erysipelotrichales;f\_\_Erysipelotrichaceae;Other |
| 0.00169630488253 | 0.17% | k\_\_Bacteria;p\_\_Firmicutes;c\_\_Clostridia;o\_\_Clostridiales;f\_\_Lachnospiraceae;g\_\_Roseburia |
| 0.0015408102683 | 0.15% | k\_\_Bacteria;p\_\_Firmicutes;c\_\_Clostridia;o\_\_Clostridiales;f\_\_Ruminococcaceae;g\_\_Intestinimonas |
| 0.00135704390602 | 0.14% | k\_\_Bacteria;p\_\_Firmicutes;c\_\_Clostridia;o\_\_Clostridiales;f\_\_Ruminococcaceae;g\_\_Butyricicoccus |
| 0.00111673404767 | 0.11% | k\_\_Bacteria;p\_\_unidentified\_Bacteria;c\_\_unidentified\_Bacteria;o\_\_unidentified\_Bacteria;f\_\_unidentified\_Bacteria;g\_\_Candidatus\_Saccharimonas |
| 0.000989511181476 | 0.10% | k\_\_Bacteria;p\_\_Firmicutes;c\_\_Clostridia;o\_\_Clostridiales;f\_\_Ruminococcaceae;g\_\_Anaerotruncus |
| 0.00090469593735 | 0.09% | k\_\_Bacteria;p\_\_Firmicutes;c\_\_Clostridia;o\_\_Clostridiales;f\_\_unidentified\_Clostridiales;g\_\_unidentified\_Clostridiales |
| 0.00090469593735 | 0.09% | k\_\_Bacteria;p\_\_Firmicutes;c\_\_Clostridia;o\_\_Clostridiales;f\_\_Ruminococcaceae;g\_\_Ruminiclostridium |
| 0.000664386078991 | 0.07% | k\_\_Bacteria;p\_\_Firmicutes;c\_\_Clostridia;o\_\_Clostridiales;f\_\_Ruminococcaceae;g\_\_Faecalibacterium |
| 0.000565434960844 | 0.06% | k\_\_Bacteria;p\_\_Bacteroidetes;c\_\_Bacteroidia;o\_\_Bacteroidales;f\_\_Marinifilaceae;g\_\_Butyricimonas |
| 0.000395804472591 | 0.04% | k\_\_Bacteria;p\_\_Actinobacteria;c\_\_unidentified\_Actinobacteria;o\_\_Bifidobacteriales;f\_\_Bifidobacteriaceae;g\_\_Bifidobacterium |
| 0.000381668598569 | 0.04% | k\_\_Bacteria;p\_\_Firmicutes;c\_\_Clostridia;o\_\_Clostridiales;f\_\_Peptococcaceae;g\_\_ |
| 0.000367532724548 | 0.04% | k\_\_Bacteria;p\_\_Bacteroidetes;c\_\_Bacteroidia;o\_\_Bacteroidales;f\_\_Prevotellaceae;g\_\_unidentified\_Prevotellaceae |
| 0.000339260976506 | 0.03% | k\_\_Bacteria;p\_\_Firmicutes;c\_\_Clostridia;o\_\_Clostridiales;f\_\_Ruminococcaceae;g\_\_Harryflintia |
| 0.000310989228464 | 0.03% | k\_\_Bacteria;p\_\_Melainabacteria;Other;Other;Other;Other |
| 0.000282717480422 | 0.03% | k\_\_Bacteria;p\_\_Fusobacteria;c\_\_Fusobacteriia;o\_\_Fusobacteriales;f\_\_Fusobacteriaceae;g\_\_Fusobacterium |
| 0.000268581606401 | 0.03% | k\_\_Bacteria;p\_\_Firmicutes;c\_\_Clostridia;o\_\_Clostridiales;f\_\_Lachnospiraceae;g\_\_Tyzzerella |
| 0.00025444573238 | 0.03% | k\_\_Bacteria;Other;Other;Other;Other;Other |
| 0.000240309858359 | 0.02% | k\_\_Bacteria;p\_\_Firmicutes;c\_\_Clostridia;o\_\_Clostridiales;f\_\_unidentified\_Clostridiales;Other |
| 0.000240309858359 | 0.02% | k\_\_Bacteria;p\_\_Firmicutes;c\_\_Clostridia;o\_\_Clostridiales;f\_\_Lachnospiraceae;g\_\_Acetatifactor |
| 0.000240309858359 | 0.02% | k\_\_Bacteria;p\_\_Firmicutes;Other;Other;Other;Other |
| 0.000212038110316 | 0.02% | k\_\_Bacteria;p\_\_Firmicutes;c\_\_Bacilli;o\_\_Lactobacillales;f\_\_Streptococcaceae;g\_\_Streptococcus |
| 0.000197902236295 | 0.02% | k\_\_Bacteria;p\_\_Firmicutes;c\_\_Clostridia;o\_\_Clostridiales;f\_\_Ruminococcaceae;g\_\_Flavonifractor |
| 0.000183766362274 | 0.02% | k\_\_Bacteria;p\_\_Firmicutes;c\_\_Clostridia;o\_\_Clostridiales;f\_\_Ruminococcaceae;g\_\_Subdoligranulum |
| 0.000169630488253 | 0.02% | k\_\_Bacteria;p\_\_Firmicutes;c\_\_Negativicutes;o\_\_Selenomonadales;f\_\_Veillonellaceae;g\_\_Dialister |
| 0.000141358740211 | 0.01% | k\_\_Bacteria;p\_\_Firmicutes;c\_\_Clostridia;o\_\_Clostridiales;f\_\_Ruminococcaceae;g\_\_Caproiciproducens |
| 0.00012722286619 | 0.01% | k\_\_Bacteria;p\_\_Proteobacteria;c\_\_Deltaproteobacteria;o\_\_Desulfovibrionales;f\_\_Desulfovibrionaceae;g\_\_ |
| 9.89511181476e-05 | 0.01% | k\_\_Bacteria;p\_\_Proteobacteria;c\_\_Gammaproteobacteria;o\_\_unidentified\_Gammaproteobacteria;f\_\_Burkholderiaceae;g\_\_Sutterella |
| 9.89511181476e-05 | 0.01% | k\_\_Bacteria;p\_\_Proteobacteria;c\_\_Gammaproteobacteria;o\_\_unidentified\_Gammaproteobacteria;f\_\_Burkholderiaceae;g\_\_Parasutterella |
| 9.89511181476e-05 | 0.01% | k\_\_Bacteria;p\_\_Firmicutes;c\_\_Negativicutes;o\_\_Selenomonadales;f\_\_Acidaminococcaceae;g\_\_Phascolarctobacterium |
| 9.89511181476e-05 | 0.01% | k\_\_Bacteria;p\_\_Firmicutes;c\_\_Clostridia;o\_\_Clostridiales;f\_\_Lachnospiraceae;g\_\_Anaerostipes |
| 8.48152441265e-05 | 0.01% | k\_\_Bacteria;p\_\_Proteobacteria;c\_\_Gammaproteobacteria;o\_\_unidentified\_Gammaproteobacteria;f\_\_Burkholderiaceae;g\_\_Delftia |
| 8.48152441265e-05 | 0.01% | k\_\_Bacteria;p\_\_Firmicutes;c\_\_Erysipelotrichia;o\_\_Erysipelotrichales;f\_\_Erysipelotrichaceae;g\_\_Faecalitalea |
| 8.48152441265e-05 | 0.01% | k\_\_Bacteria;p\_\_Bacteroidetes;c\_\_Bacteroidia;o\_\_Bacteroidales;f\_\_Prevotellaceae;g\_\_Prevotella |
| 7.06793701055e-05 | 0.01% | k\_\_Bacteria;p\_\_Proteobacteria;c\_\_Gammaproteobacteria;o\_\_Pseudomonadales;f\_\_Pseudomonadaceae;g\_\_Pseudomonas |
| 7.06793701055e-05 | 0.01% | k\_\_Bacteria;p\_\_Proteobacteria;c\_\_Gammaproteobacteria;o\_\_Pseudomonadales;f\_\_Moraxellaceae;g\_\_Acinetobacter |
| 7.06793701055e-05 | 0.01% | k\_\_Bacteria;p\_\_Bacteroidetes;c\_\_Bacteroidia;o\_\_Bacteroidales;f\_\_Marinifilaceae;g\_\_Odoribacter |
| 7.06793701055e-05 | 0.01% | k\_\_Bacteria;p\_\_Actinobacteria;c\_\_unidentified\_Actinobacteria;o\_\_Micrococcales;f\_\_Microbacteriaceae;Other |
| 5.65434960844e-05 | 0.01% | k\_\_Bacteria;p\_\_Proteobacteria;c\_\_Gammaproteobacteria;o\_\_unidentified\_Gammaproteobacteria;f\_\_Burkholderiaceae;Other |
| 5.65434960844e-05 | 0.01% | k\_\_Bacteria;p\_\_Firmicutes;c\_\_Clostridia;o\_\_Clostridiales;f\_\_Peptococcaceae;g\_\_Peptococcus |
| 5.65434960844e-05 | 0.01% | k\_\_Bacteria;p\_\_Firmicutes;c\_\_Clostridia;o\_\_Clostridiales;f\_\_Peptococcaceae;Other |
| 5.65434960844e-05 | 0.01% | k\_\_Bacteria;p\_\_Firmicutes;c\_\_Clostridia;o\_\_Clostridiales;f\_\_Lachnospiraceae;g\_\_Fusicatenibacter |
| 5.65434960844e-05 | 0.01% | k\_\_Bacteria;p\_\_Bacteroidetes;c\_\_Bacteroidia;o\_\_Flavobacteriales;f\_\_Flavobacteriaceae;g\_\_Tenacibaculum |
| 5.65434960844e-05 | 0.01% | k\_\_Bacteria;p\_\_Bacteroidetes;c\_\_Bacteroidia;o\_\_Bacteroidales;f\_\_Porphyromonadaceae;g\_\_Porphyromonas |
| 5.65434960844e-05 | 0.01% | k\_\_Bacteria;p\_\_Actinobacteria;c\_\_unidentified\_Actinobacteria;o\_\_Corynebacteriales;f\_\_Nocardiaceae;g\_\_Rhodococcus |
| 4.24076220633e-05 | 0.00% | k\_\_Bacteria;p\_\_Firmicutes;c\_\_Erysipelotrichia;o\_\_Erysipelotrichales;f\_\_Erysipelotrichaceae;g\_\_Turicibacter |
| 4.24076220633e-05 | 0.00% | k\_\_Bacteria;p\_\_Firmicutes;c\_\_Erysipelotrichia;o\_\_Erysipelotrichales;f\_\_Erysipelotrichaceae;g\_\_Erysipelatoclostridium |
| 4.24076220633e-05 | 0.00% | k\_\_Bacteria;p\_\_Bacteroidetes;c\_\_Bacteroidia;o\_\_Bacteroidales;f\_\_Barnesiellaceae;g\_\_Coprobacter |
| 2.82717480422e-05 | 0.00% | k\_\_Bacteria;p\_\_unidentified\_Bacteria;c\_\_unidentified\_Bacteria;o\_\_Campylobacterales;f\_\_unidentified\_Campylobacterales;g\_\_Sulfurospirillum |
| 2.82717480422e-05 | 0.00% | k\_\_Bacteria;p\_\_Proteobacteria;c\_\_Deltaproteobacteria;o\_\_Desulfovibrionales;f\_\_Desulfovibrionaceae;g\_\_Bilophila |
| 2.82717480422e-05 | 0.00% | k\_\_Bacteria;p\_\_Proteobacteria;c\_\_Alphaproteobacteria;o\_\_Sphingomonadales;f\_\_Sphingomonadaceae;g\_\_Sphingomonas |
| 2.82717480422e-05 | 0.00% | k\_\_Bacteria;p\_\_Firmicutes;c\_\_Negativicutes;o\_\_Selenomonadales;f\_\_Veillonellaceae;g\_\_Megasphaera |
| 2.82717480422e-05 | 0.00% | k\_\_Bacteria;p\_\_Firmicutes;c\_\_Negativicutes;o\_\_Selenomonadales;f\_\_Veillonellaceae;g\_\_Megamonas |
| 2.82717480422e-05 | 0.00% | k\_\_Bacteria;p\_\_Firmicutes;c\_\_Erysipelotrichia;o\_\_Erysipelotrichales;f\_\_Erysipelotrichaceae;g\_\_unidentified\_Erysipelotrichaceae |
| 2.82717480422e-05 | 0.00% | k\_\_Bacteria;p\_\_Firmicutes;c\_\_Bacilli;o\_\_Bacillales;f\_\_Staphylococcaceae;g\_\_Staphylococcus |
| 1.41358740211e-05 | 0.00% | k\_\_Bacteria;p\_\_Proteobacteria;c\_\_Gammaproteobacteria;o\_\_Xanthomonadales;f\_\_Xanthomonadaceae;g\_\_Stenotrophomonas |
| 1.41358740211e-05 | 0.00% | k\_\_Bacteria;p\_\_Proteobacteria;c\_\_Gammaproteobacteria;o\_\_Pasteurellales;f\_\_Pasteurellaceae;Other |
| 1.41358740211e-05 | 0.00% | k\_\_Bacteria;p\_\_Proteobacteria;c\_\_Alphaproteobacteria;o\_\_unidentified\_Alphaproteobacteria;f\_\_unidentified\_Alphaproteobacteria;g\_\_Dongia |
| 1.41358740211e-05 | 0.00% | k\_\_Bacteria;p\_\_Proteobacteria;c\_\_Alphaproteobacteria;o\_\_Rhizobiales;Other;Other |
| 1.41358740211e-05 | 0.00% | k\_\_Bacteria;p\_\_Oxyphotobacteria;c\_\_unidentified\_Oxyphotobacteria;o\_\_unidentified\_Oxyphotobacteria;f\_\_unidentified\_Oxyphotobacteria;g\_\_unidentified\_Oxyphotobacteria |
| 1.41358740211e-05 | 0.00% | k\_\_Bacteria;p\_\_Firmicutes;c\_\_Erysipelotrichia;o\_\_Erysipelotrichales;f\_\_Erysipelotrichaceae;g\_\_Holdemania |
| 1.41358740211e-05 | 0.00% | k\_\_Bacteria;p\_\_Firmicutes;c\_\_Clostridia;o\_\_Clostridiales;f\_\_unidentified\_Clostridiales;g\_\_Parvimonas |
| 1.41358740211e-05 | 0.00% | k\_\_Bacteria;p\_\_Firmicutes;c\_\_Clostridia;o\_\_Clostridiales;f\_\_Eubacteriaceae;g\_\_Anaerofustis |
| 1.41358740211e-05 | 0.00% | k\_\_Bacteria;p\_\_Bacteroidetes;c\_\_Bacteroidia;o\_\_Bacteroidales;f\_\_Barnesiellaceae;g\_\_Barnesiella |
| 1.41358740211e-05 | 0.00% | k\_\_Bacteria;p\_\_Actinobacteria;c\_\_Rubrobacteria;o\_\_Rubrobacterales;f\_\_unidentified\_Rubrobacterales;g\_\_Rubrobacter |
| 1.41358740211e-05 | 0.00% | k\_\_Archaea;p\_\_Euryarchaeota;c\_\_Methanobacteria;o\_\_Methanobacteriales;f\_\_Methanobacteriaceae;g\_\_Methanobrevibacter |

|  |  |
| --- | --- |
|  | |
| Taxonomy Summary. Current Level: \_MC3 | |
| View Figure (.pdf)  View Legend (.pdf) |  |
|  |  |
| View Figure (.pdf)  View Legend (.pdf) |  |
|  |  |
|  |  |

|  |  |  |
| --- | --- | --- |
| Count | Pct | Taxonomy |
| 0.332800882079 | 33.28% | k\_\_Bacteria;p\_\_Bacteroidetes;c\_\_Bacteroidia;o\_\_Bacteroidales;f\_\_Muribaculaceae;g\_\_ |
| 0.213508241215 | 21.35% | k\_\_Bacteria;p\_\_Bacteroidetes;c\_\_Bacteroidia;o\_\_Bacteroidales;f\_\_Bacteroidaceae;g\_\_Bacteroides |
| 0.0861157445365 | 8.61% | k\_\_Bacteria;p\_\_Firmicutes;c\_\_Clostridia;o\_\_Clostridiales;f\_\_Lachnospiraceae;Other |
| 0.0793305250064 | 7.93% | k\_\_Bacteria;p\_\_Firmicutes;c\_\_Clostridia;o\_\_Clostridiales;f\_\_Ruminococcaceae;Other |
| 0.0705097396172 | 7.05% | k\_\_Bacteria;p\_\_Firmicutes;c\_\_Clostridia;o\_\_Clostridiales;f\_\_Ruminococcaceae;g\_\_unidentified\_Ruminococcaceae |
| 0.0307879336179 | 3.08% | k\_\_Bacteria;p\_\_Bacteroidetes;c\_\_Bacteroidia;o\_\_Bacteroidales;f\_\_Rikenellaceae;g\_\_Alistipes |
| 0.0214017132679 | 2.14% | k\_\_Bacteria;p\_\_Firmicutes;c\_\_Clostridia;o\_\_Clostridiales;f\_\_Lachnospiraceae;g\_\_unidentified\_Lachnospiraceae |
| 0.019436826779 | 1.94% | k\_\_Bacteria;p\_\_Bacteroidetes;c\_\_Bacteroidia;o\_\_Bacteroidales;f\_\_Prevotellaceae;g\_\_Alloprevotella |
| 0.0192530604167 | 1.93% | k\_\_Bacteria;p\_\_Proteobacteria;c\_\_Gammaproteobacteria;o\_\_Enterobacteriales;f\_\_Enterobacteriaceae;g\_\_unidentified\_Enterobacteriaceae |
| 0.0186452178338 | 1.86% | k\_\_Bacteria;p\_\_Proteobacteria;c\_\_Deltaproteobacteria;o\_\_Desulfovibrionales;f\_\_Desulfovibrionaceae;g\_\_Desulfovibrio |
| 0.0180232393769 | 1.80% | k\_\_Bacteria;p\_\_Proteobacteria;c\_\_Gammaproteobacteria;o\_\_Enterobacteriales;f\_\_Enterobacteriaceae;g\_\_Proteus |
| 0.00988097594074 | 0.99% | k\_\_Bacteria;p\_\_Firmicutes;c\_\_Clostridia;o\_\_Clostridiales;f\_\_Lachnospiraceae;g\_\_Roseburia |
| 0.00835430154646 | 0.84% | k\_\_Bacteria;p\_\_Firmicutes;c\_\_Bacilli;o\_\_Lactobacillales;f\_\_Lactobacillaceae;g\_\_Lactobacillus |
| 0.00819880693223 | 0.82% | k\_\_Bacteria;p\_\_Bacteroidetes;c\_\_Bacteroidia;o\_\_Bacteroidales;f\_\_Muribaculaceae;g\_\_Muribaculum |
| 0.00634700743547 | 0.63% | k\_\_Bacteria;p\_\_Bacteroidetes;c\_\_Bacteroidia;o\_\_Bacteroidales;f\_\_Tannerellaceae;g\_\_Parabacteroides |
| 0.00620564869526 | 0.62% | k\_\_Bacteria;p\_\_Firmicutes;c\_\_Erysipelotrichia;o\_\_Erysipelotrichales;f\_\_Erysipelotrichaceae;Other |
| 0.00562607786039 | 0.56% | k\_\_Bacteria;p\_\_Firmicutes;c\_\_Clostridia;o\_\_Clostridiales;f\_\_Lachnospiraceae;g\_\_Lachnoclostridium |
| 0.00494755590738 | 0.49% | k\_\_Bacteria;p\_\_Proteobacteria;c\_\_Gammaproteobacteria;o\_\_Enterobacteriales;f\_\_Enterobacteriaceae;Other |
| 0.00414181108818 | 0.41% | k\_\_Bacteria;p\_\_Firmicutes;c\_\_Bacilli;o\_\_Lactobacillales;f\_\_Enterococcaceae;g\_\_Enterococcus |
| 0.00319470752877 | 0.32% | k\_\_Bacteria;p\_\_Firmicutes;c\_\_Clostridia;o\_\_Clostridiales;f\_\_Ruminococcaceae;g\_\_Butyricicoccus |
| 0.0031522999067 | 0.32% | k\_\_Bacteria;p\_\_Tenericutes;c\_\_Mollicutes;o\_\_;f\_\_;g\_\_ |
| 0.00286958242628 | 0.29% | k\_\_Bacteria;p\_\_Firmicutes;c\_\_Clostridia;o\_\_Clostridiales;Other;Other |
| 0.00229001159142 | 0.23% | k\_\_Bacteria;p\_\_Firmicutes;c\_\_Clostridia;o\_\_Clostridiales;f\_\_Lachnospiraceae;g\_\_Blautia |
| 0.00209210935512 | 0.21% | k\_\_Bacteria;p\_\_Firmicutes;c\_\_Clostridia;o\_\_Clostridiales;f\_\_Ruminococcaceae;g\_\_Intestinimonas |
| 0.00202142998502 | 0.20% | k\_\_Bacteria;p\_\_Bacteroidetes;c\_\_Bacteroidia;o\_\_Bacteroidales;f\_\_Prevotellaceae;Other |
| 0.00200729411099 | 0.20% | k\_\_Bacteria;p\_\_Firmicutes;c\_\_Clostridia;o\_\_Clostridiales;f\_\_Ruminococcaceae;g\_\_Oscillibacter |
| 0.00163976138645 | 0.16% | k\_\_Bacteria;p\_\_Firmicutes;c\_\_Clostridia;o\_\_Clostridiales;f\_\_Lachnospiraceae;g\_\_ |
| 0.00114500579571 | 0.11% | k\_\_Bacteria;p\_\_Verrucomicrobia;c\_\_Verrucomicrobiae;o\_\_Verrucomicrobiales;f\_\_Akkermansiaceae;g\_\_Akkermansia |
| 0.00111673404767 | 0.11% | k\_\_Bacteria;p\_\_Firmicutes;c\_\_Clostridia;o\_\_Clostridiales;f\_\_Ruminococcaceae;g\_\_Ruminiclostridium |
| 0.00108846229962 | 0.11% | k\_\_Bacteria;p\_\_Firmicutes;c\_\_Clostridia;o\_\_Clostridiales;f\_\_Ruminococcaceae;g\_\_Anaerotruncus |
| 0.00106019055158 | 0.11% | k\_\_Bacteria;p\_\_Firmicutes;c\_\_Clostridia;o\_\_Clostridiales;f\_\_Ruminococcaceae;g\_\_Faecalibacterium |
| 0.000834016567244 | 0.08% | k\_\_Bacteria;p\_\_Firmicutes;c\_\_Clostridia;o\_\_Clostridiales;f\_\_Lachnospiraceae;g\_\_Marvinbryantia |
| 0.000706793701055 | 0.07% | k\_\_Bacteria;Other;Other;Other;Other;Other |
| 0.000678521953012 | 0.07% | k\_\_Bacteria;p\_\_Firmicutes;c\_\_Clostridia;o\_\_Clostridiales;f\_\_unidentified\_Clostridiales;g\_\_unidentified\_Clostridiales |
| 0.00065025020497 | 0.07% | k\_\_Bacteria;p\_\_Bacteroidetes;c\_\_Bacteroidia;o\_\_Bacteroidales;f\_\_Prevotellaceae;g\_\_unidentified\_Prevotellaceae |
| 0.000593706708886 | 0.06% | k\_\_Bacteria;p\_\_unidentified\_Bacteria;c\_\_unidentified\_Bacteria;o\_\_unidentified\_Bacteria;f\_\_unidentified\_Bacteria;g\_\_Candidatus\_Saccharimonas |
| 0.000593706708886 | 0.06% | k\_\_Bacteria;p\_\_Firmicutes;c\_\_Clostridia;o\_\_Clostridiales;f\_\_Ruminococcaceae;g\_\_Subdoligranulum |
| 0.000508891464759 | 0.05% | k\_\_Bacteria;p\_\_Firmicutes;c\_\_Clostridia;o\_\_Clostridiales;f\_\_Ruminococcaceae;g\_\_Caproiciproducens |
| 0.000466483842696 | 0.05% | k\_\_Bacteria;p\_\_Firmicutes;c\_\_Clostridia;o\_\_Clostridiales;f\_\_unidentified\_Clostridiales;g\_\_Anaerovorax |
| 0.000452347968675 | 0.05% | k\_\_Bacteria;p\_\_Firmicutes;c\_\_Clostridia;o\_\_Clostridiales;f\_\_unidentified\_Clostridiales;Other |
| 0.000452347968675 | 0.05% | k\_\_Bacteria;p\_\_Firmicutes;c\_\_Clostridia;o\_\_Clostridiales;f\_\_Peptococcaceae;g\_\_ |
| 0.000452347968675 | 0.05% | k\_\_Bacteria;p\_\_Firmicutes;Other;Other;Other;Other |
| 0.000438212094654 | 0.04% | k\_\_Bacteria;p\_\_Firmicutes;c\_\_Erysipelotrichia;o\_\_Erysipelotrichales;f\_\_Erysipelotrichaceae;g\_\_Dubosiella |
| 0.000353396850527 | 0.04% | k\_\_Bacteria;p\_\_Actinobacteria;c\_\_unidentified\_Actinobacteria;o\_\_Bifidobacteriales;f\_\_Bifidobacteriaceae;g\_\_Bifidobacterium |
| 0.000282717480422 | 0.03% | k\_\_Bacteria;p\_\_Firmicutes;c\_\_Negativicutes;o\_\_Selenomonadales;f\_\_Veillonellaceae;g\_\_Dialister |
| 0.00025444573238 | 0.03% | k\_\_Bacteria;p\_\_Fusobacteria;c\_\_Fusobacteriia;o\_\_Fusobacteriales;f\_\_Fusobacteriaceae;g\_\_Fusobacterium |
| 0.00025444573238 | 0.03% | k\_\_Bacteria;p\_\_Bacteroidetes;c\_\_Bacteroidia;o\_\_Bacteroidales;f\_\_Marinifilaceae;g\_\_Butyricimonas |
| 0.000240309858359 | 0.02% | k\_\_Bacteria;p\_\_Firmicutes;c\_\_Bacilli;o\_\_Lactobacillales;f\_\_Streptococcaceae;g\_\_Streptococcus |
| 0.000226173984337 | 0.02% | k\_\_Bacteria;p\_\_Proteobacteria;c\_\_Gammaproteobacteria;o\_\_Enterobacteriales;f\_\_Enterobacteriaceae;g\_\_Morganella |
| 0.000197902236295 | 0.02% | k\_\_Bacteria;p\_\_Firmicutes;c\_\_Clostridia;o\_\_Clostridiales;f\_\_Lachnospiraceae;g\_\_Tyzzerella |
| 0.000183766362274 | 0.02% | k\_\_Bacteria;p\_\_Proteobacteria;c\_\_Gammaproteobacteria;o\_\_Pasteurellales;f\_\_Pasteurellaceae;Other |
| 0.000155494614232 | 0.02% | k\_\_Bacteria;p\_\_Melainabacteria;Other;Other;Other;Other |
| 0.000155494614232 | 0.02% | k\_\_Bacteria;p\_\_Firmicutes;c\_\_Clostridia;o\_\_Clostridiales;f\_\_Ruminococcaceae;g\_\_Flavonifractor |
| 0.000155494614232 | 0.02% | k\_\_Bacteria;p\_\_Firmicutes;c\_\_Clostridia;o\_\_Clostridiales;f\_\_Lachnospiraceae;g\_\_Fusicatenibacter |
| 0.000141358740211 | 0.01% | k\_\_Bacteria;p\_\_Proteobacteria;c\_\_Gammaproteobacteria;o\_\_unidentified\_Gammaproteobacteria;f\_\_Burkholderiaceae;g\_\_Sutterella |
| 0.000141358740211 | 0.01% | k\_\_Bacteria;p\_\_Proteobacteria;c\_\_Gammaproteobacteria;o\_\_Pseudomonadales;f\_\_Pseudomonadaceae;g\_\_Pseudomonas |
| 0.000141358740211 | 0.01% | k\_\_Bacteria;p\_\_Firmicutes;c\_\_Erysipelotrichia;o\_\_Erysipelotrichales;f\_\_Erysipelotrichaceae;g\_\_unidentified\_Erysipelotrichaceae |
| 0.00012722286619 | 0.01% | k\_\_Bacteria;p\_\_Proteobacteria;c\_\_Gammaproteobacteria;o\_\_Pseudomonadales;f\_\_Moraxellaceae;g\_\_Acinetobacter |
| 0.00012722286619 | 0.01% | k\_\_Bacteria;p\_\_Firmicutes;c\_\_Clostridia;o\_\_Clostridiales;f\_\_Ruminococcaceae;g\_\_Harryflintia |
| 0.00012722286619 | 0.01% | k\_\_Bacteria;p\_\_Actinobacteria;c\_\_Thermoleophilia;o\_\_Solirubrobacterales;f\_\_;g\_\_ |
| 0.000113086992169 | 0.01% | k\_\_Bacteria;p\_\_unidentified\_Bacteria;c\_\_unidentified\_Bacteria;o\_\_Campylobacterales;f\_\_unidentified\_Campylobacterales;g\_\_Sulfurospirillum |
| 0.000113086992169 | 0.01% | k\_\_Bacteria;p\_\_Firmicutes;c\_\_Bacilli;o\_\_Bacillales;f\_\_Staphylococcaceae;g\_\_Staphylococcus |
| 0.000113086992169 | 0.01% | k\_\_Bacteria;p\_\_Bacteroidetes;c\_\_Bacteroidia;o\_\_Bacteroidales;f\_\_Porphyromonadaceae;g\_\_Porphyromonas |
| 0.000113086992169 | 0.01% | k\_\_Bacteria;p\_\_Bacteroidetes;c\_\_Bacteroidia;o\_\_Bacteroidales;f\_\_Marinifilaceae;g\_\_Odoribacter |
| 9.89511181476e-05 | 0.01% | k\_\_Bacteria;p\_\_Proteobacteria;c\_\_Gammaproteobacteria;o\_\_unidentified\_Gammaproteobacteria;f\_\_Burkholderiaceae;g\_\_Diaphorobacter |
| 9.89511181476e-05 | 0.01% | k\_\_Bacteria;p\_\_Proteobacteria;c\_\_Deltaproteobacteria;o\_\_Desulfovibrionales;f\_\_Desulfovibrionaceae;g\_\_ |
| 8.48152441265e-05 | 0.01% | k\_\_Bacteria;p\_\_Bacteroidetes;c\_\_Bacteroidia;o\_\_Bacteroidales;f\_\_Prevotellaceae;g\_\_Prevotella |
| 7.06793701055e-05 | 0.01% | k\_\_Bacteria;p\_\_Proteobacteria;c\_\_Gammaproteobacteria;o\_\_unidentified\_Gammaproteobacteria;f\_\_Burkholderiaceae;g\_\_Parasutterella |
| 7.06793701055e-05 | 0.01% | k\_\_Bacteria;p\_\_Proteobacteria;c\_\_Gammaproteobacteria;o\_\_unidentified\_Gammaproteobacteria;f\_\_Burkholderiaceae;g\_\_Delftia |
| 7.06793701055e-05 | 0.01% | k\_\_Bacteria;p\_\_Firmicutes;c\_\_Negativicutes;o\_\_Selenomonadales;f\_\_Acidaminococcaceae;g\_\_Phascolarctobacterium |
| 7.06793701055e-05 | 0.01% | k\_\_Bacteria;p\_\_Firmicutes;c\_\_Clostridia;o\_\_Clostridiales;f\_\_Eubacteriaceae;g\_\_Anaerofustis |
| 7.06793701055e-05 | 0.01% | k\_\_Bacteria;p\_\_Bacteroidetes;c\_\_Bacteroidia;o\_\_Chitinophagales;f\_\_Chitinophagaceae;g\_\_Segetibacter |
| 5.65434960844e-05 | 0.01% | k\_\_Bacteria;p\_\_Proteobacteria;c\_\_Gammaproteobacteria;o\_\_unidentified\_Gammaproteobacteria;f\_\_Burkholderiaceae;Other |
| 5.65434960844e-05 | 0.01% | k\_\_Bacteria;p\_\_Proteobacteria;c\_\_Alphaproteobacteria;o\_\_Sphingomonadales;f\_\_Sphingomonadaceae;g\_\_Sphingomonas |
| 5.65434960844e-05 | 0.01% | k\_\_Bacteria;p\_\_Proteobacteria;c\_\_Alphaproteobacteria;o\_\_Rhizobiales;f\_\_Rhizobiaceae;g\_\_Phyllobacterium |
| 5.65434960844e-05 | 0.01% | k\_\_Bacteria;p\_\_Oxyphotobacteria;c\_\_unidentified\_Oxyphotobacteria;o\_\_unidentified\_Oxyphotobacteria;f\_\_unidentified\_Oxyphotobacteria;g\_\_unidentified\_Oxyphotobacteria |
| 5.65434960844e-05 | 0.01% | k\_\_Bacteria;p\_\_Firmicutes;c\_\_Negativicutes;o\_\_Selenomonadales;f\_\_Veillonellaceae;g\_\_Megasphaera |
| 5.65434960844e-05 | 0.01% | k\_\_Bacteria;p\_\_Firmicutes;c\_\_Negativicutes;o\_\_Selenomonadales;f\_\_Veillonellaceae;g\_\_Megamonas |
| 5.65434960844e-05 | 0.01% | k\_\_Bacteria;p\_\_Firmicutes;c\_\_Erysipelotrichia;o\_\_Erysipelotrichales;f\_\_Erysipelotrichaceae;g\_\_Erysipelatoclostridium |
| 5.65434960844e-05 | 0.01% | k\_\_Bacteria;p\_\_Bacteroidetes;c\_\_Bacteroidia;o\_\_Bacteroidales;f\_\_Barnesiellaceae;g\_\_Barnesiella |
| 5.65434960844e-05 | 0.01% | k\_\_Archaea;p\_\_Euryarchaeota;c\_\_Methanobacteria;o\_\_Methanobacteriales;f\_\_Methanobacteriaceae;g\_\_Methanobrevibacter |
| 4.24076220633e-05 | 0.00% | k\_\_Bacteria;p\_\_Proteobacteria;c\_\_Gammaproteobacteria;o\_\_unidentified\_Gammaproteobacteria;f\_\_Neisseriaceae;g\_\_Neisseria |
| 4.24076220633e-05 | 0.00% | k\_\_Bacteria;p\_\_Proteobacteria;c\_\_Gammaproteobacteria;o\_\_Xanthomonadales;f\_\_Xanthomonadaceae;g\_\_Stenotrophomonas |
| 4.24076220633e-05 | 0.00% | k\_\_Bacteria;p\_\_Proteobacteria;c\_\_Deltaproteobacteria;o\_\_Desulfovibrionales;f\_\_Desulfovibrionaceae;g\_\_Bilophila |
| 4.24076220633e-05 | 0.00% | k\_\_Bacteria;p\_\_Proteobacteria;c\_\_Alphaproteobacteria;o\_\_Rhodospirillales;f\_\_unidentified\_Rhodospirillales;g\_\_Telmatospirillum |
| 4.24076220633e-05 | 0.00% | k\_\_Bacteria;p\_\_Firmicutes;c\_\_Erysipelotrichia;o\_\_Erysipelotrichales;f\_\_Erysipelotrichaceae;g\_\_Faecalitalea |
| 4.24076220633e-05 | 0.00% | k\_\_Bacteria;p\_\_Firmicutes;c\_\_Clostridia;o\_\_Clostridiales;f\_\_Peptostreptococcaceae;g\_\_Romboutsia |
| 4.24076220633e-05 | 0.00% | k\_\_Bacteria;p\_\_Firmicutes;c\_\_Clostridia;o\_\_Clostridiales;f\_\_Peptostreptococcaceae;g\_\_Peptostreptococcus |
| 4.24076220633e-05 | 0.00% | k\_\_Bacteria;p\_\_Firmicutes;c\_\_Clostridia;o\_\_Clostridiales;f\_\_Lachnospiraceae;g\_\_Anaerostipes |
| 4.24076220633e-05 | 0.00% | k\_\_Bacteria;p\_\_Firmicutes;c\_\_Clostridia;o\_\_Clostridiales;f\_\_Christensenellaceae;g\_\_ |
| 4.24076220633e-05 | 0.00% | k\_\_Bacteria;p\_\_Bacteroidetes;c\_\_Bacteroidia;o\_\_Bacteroidales;f\_\_Prevotellaceae;g\_\_ |
| 4.24076220633e-05 | 0.00% | k\_\_Bacteria;p\_\_Actinobacteria;c\_\_unidentified\_Actinobacteria;o\_\_Corynebacteriales;f\_\_Nocardiaceae;g\_\_Rhodococcus |
| 4.24076220633e-05 | 0.00% | k\_\_Bacteria;p\_\_Actinobacteria;c\_\_Rubrobacteria;o\_\_Rubrobacterales;f\_\_unidentified\_Rubrobacterales;g\_\_Rubrobacter |
| 2.82717480422e-05 | 0.00% | k\_\_Bacteria;p\_\_unidentified\_Bacteria;c\_\_unidentified\_Bacteria;o\_\_Campylobacterales;f\_\_Campylobacteraceae;g\_\_Campylobacter |
| 2.82717480422e-05 | 0.00% | k\_\_Bacteria;p\_\_Tenericutes;c\_\_Mollicutes;o\_\_Anaeroplasmatales;f\_\_Anaeroplasmataceae;g\_\_Anaeroplasma |
| 2.82717480422e-05 | 0.00% | k\_\_Bacteria;p\_\_Proteobacteria;c\_\_Gammaproteobacteria;o\_\_Xanthomonadales;f\_\_Xanthomonadaceae;g\_\_Arenimonas |
| 2.82717480422e-05 | 0.00% | k\_\_Bacteria;p\_\_Proteobacteria;c\_\_Alphaproteobacteria;o\_\_Sphingomonadales;f\_\_Sphingomonadaceae;g\_\_Novosphingobium |
| 2.82717480422e-05 | 0.00% | k\_\_Bacteria;p\_\_Proteobacteria;c\_\_Alphaproteobacteria;o\_\_Rhizobiales;f\_\_Rhizobiaceae;g\_\_Brucella |
| 2.82717480422e-05 | 0.00% | k\_\_Bacteria;p\_\_Firmicutes;c\_\_Negativicutes;o\_\_Selenomonadales;f\_\_Veillonellaceae;g\_\_Veillonella |
| 2.82717480422e-05 | 0.00% | k\_\_Bacteria;p\_\_Firmicutes;c\_\_Clostridia;o\_\_Clostridiales;f\_\_unidentified\_Clostridiales;g\_\_Parvimonas |
| 2.82717480422e-05 | 0.00% | k\_\_Bacteria;p\_\_Firmicutes;c\_\_Clostridia;o\_\_Clostridiales;f\_\_Ruminococcaceae;g\_\_Hydrogenoanaerobacterium |
| 2.82717480422e-05 | 0.00% | k\_\_Bacteria;p\_\_Firmicutes;c\_\_Clostridia;o\_\_Clostridiales;f\_\_Peptostreptococcaceae;g\_\_Clostridioides |
| 2.82717480422e-05 | 0.00% | k\_\_Bacteria;p\_\_Firmicutes;c\_\_Clostridia;o\_\_Clostridiales;f\_\_Peptococcaceae;Other |
| 2.82717480422e-05 | 0.00% | k\_\_Bacteria;p\_\_Firmicutes;c\_\_Clostridia;o\_\_Clostridiales;f\_\_Lachnospiraceae;g\_\_Acetatifactor |
| 2.82717480422e-05 | 0.00% | k\_\_Bacteria;p\_\_Firmicutes;c\_\_Bacilli;o\_\_Lactobacillales;f\_\_Leuconostocaceae;g\_\_Leuconostoc |
| 2.82717480422e-05 | 0.00% | k\_\_Bacteria;p\_\_Bacteroidetes;c\_\_Bacteroidia;o\_\_Sphingobacteriales;f\_\_;g\_\_ |
| 2.82717480422e-05 | 0.00% | k\_\_Bacteria;p\_\_Bacteroidetes;c\_\_Bacteroidia;o\_\_Bacteroidales;f\_\_Barnesiellaceae;g\_\_Coprobacter |
| 2.82717480422e-05 | 0.00% | k\_\_Bacteria;p\_\_Acidobacteria;c\_\_unidentified\_Acidobacteria;o\_\_unidentified\_Acidobacteria;f\_\_unidentified\_Acidobacteria;g\_\_Blastocatella |
| 1.41358740211e-05 | 0.00% | k\_\_Bacteria;p\_\_Proteobacteria;c\_\_Gammaproteobacteria;o\_\_unidentified\_Gammaproteobacteria;f\_\_Burkholderiaceae;g\_\_Ralstonia |
| 1.41358740211e-05 | 0.00% | k\_\_Bacteria;p\_\_Proteobacteria;c\_\_Gammaproteobacteria;o\_\_unidentified\_Gammaproteobacteria;f\_\_Burkholderiaceae;g\_\_Achromobacter |
| 1.41358740211e-05 | 0.00% | k\_\_Bacteria;p\_\_Proteobacteria;c\_\_Gammaproteobacteria;o\_\_Xanthomonadales;f\_\_Rhodanobacteraceae;g\_\_Rhodanobacter |
| 1.41358740211e-05 | 0.00% | k\_\_Bacteria;p\_\_Proteobacteria;c\_\_Alphaproteobacteria;o\_\_Rhodospirillales;f\_\_unidentified\_Rhodospirillales;g\_\_Defluviicoccus |
| 1.41358740211e-05 | 0.00% | k\_\_Bacteria;p\_\_Fusobacteria;c\_\_Fusobacteriia;o\_\_Fusobacteriales;f\_\_Fusobacteriaceae;Other |
| 1.41358740211e-05 | 0.00% | k\_\_Bacteria;p\_\_Firmicutes;c\_\_Negativicutes;o\_\_Selenomonadales;f\_\_Acidaminococcaceae;g\_\_Acidaminococcus |
| 1.41358740211e-05 | 0.00% | k\_\_Bacteria;p\_\_Firmicutes;c\_\_Erysipelotrichia;o\_\_Erysipelotrichales;f\_\_Erysipelotrichaceae;g\_\_Turicibacter |
| 1.41358740211e-05 | 0.00% | k\_\_Bacteria;p\_\_Firmicutes;c\_\_Clostridia;o\_\_Clostridiales;f\_\_Lachnospiraceae;g\_\_Agathobacter |
| 1.41358740211e-05 | 0.00% | k\_\_Bacteria;p\_\_Firmicutes;c\_\_Bacilli;o\_\_Bacillales;f\_\_unidentified\_Bacillales;g\_\_Exiguobacterium |
| 1.41358740211e-05 | 0.00% | k\_\_Bacteria;p\_\_Deinococcus-Thermus;c\_\_Deinococci;o\_\_Deinococcales;f\_\_Deinococcaceae;g\_\_Deinococcus |
| 1.41358740211e-05 | 0.00% | k\_\_Bacteria;p\_\_Bacteroidetes;c\_\_Bacteroidia;o\_\_Flavobacteriales;f\_\_Flavobacteriaceae;Other |
| 1.41358740211e-05 | 0.00% | k\_\_Bacteria;p\_\_Actinobacteria;c\_\_Thermoleophilia;o\_\_Gaiellales;f\_\_;g\_\_ |
| 1.41358740211e-05 | 0.00% | k\_\_Bacteria;p\_\_Actinobacteria;c\_\_Coriobacteriia;o\_\_Coriobacteriales;f\_\_Atopobiaceae;g\_\_Atopobium |

|  |  |
| --- | --- |
|  | |
| Taxonomy Summary. Current Level: \_MC4 | |
| View Figure (.pdf)  View Legend (.pdf) |  |
|  |  |
| View Figure (.pdf)  View Legend (.pdf) |  |
|  |  |
|  |  |

|  |  |  |
| --- | --- | --- |
| Count | Pct | Taxonomy |
| 0.23451415001 | 23.45% | k\_\_Bacteria;p\_\_Bacteroidetes;c\_\_Bacteroidia;o\_\_Bacteroidales;f\_\_Muribaculaceae;g\_\_ |
| 0.154278929066 | 15.43% | k\_\_Bacteria;p\_\_Firmicutes;c\_\_Clostridia;o\_\_Clostridiales;f\_\_Lachnospiraceae;Other |
| 0.131180910916 | 13.12% | k\_\_Bacteria;p\_\_Firmicutes;c\_\_Clostridia;o\_\_Clostridiales;f\_\_Ruminococcaceae;Other |
| 0.103941081677 | 10.39% | k\_\_Bacteria;p\_\_Bacteroidetes;c\_\_Bacteroidia;o\_\_Bacteroidales;f\_\_Bacteroidaceae;g\_\_Bacteroides |
| 0.0832744338582 | 8.33% | k\_\_Bacteria;p\_\_Firmicutes;c\_\_Clostridia;o\_\_Clostridiales;f\_\_Lachnospiraceae;g\_\_unidentified\_Lachnospiraceae |
| 0.0626077860394 | 6.26% | k\_\_Bacteria;p\_\_Firmicutes;c\_\_Clostridia;o\_\_Clostridiales;f\_\_Ruminococcaceae;g\_\_unidentified\_Ruminococcaceae |
| 0.0282717480422 | 2.83% | k\_\_Bacteria;p\_\_Bacteroidetes;c\_\_Bacteroidia;o\_\_Bacteroidales;f\_\_Rikenellaceae;g\_\_Alistipes |
| 0.0268440247661 | 2.68% | k\_\_Bacteria;p\_\_Firmicutes;c\_\_Clostridia;o\_\_Clostridiales;f\_\_Ruminococcaceae;g\_\_Oscillibacter |
| 0.0244974696786 | 2.45% | k\_\_Bacteria;p\_\_Firmicutes;c\_\_Clostridia;o\_\_Clostridiales;f\_\_Lachnospiraceae;g\_\_Lachnoclostridium |
| 0.0193944191569 | 1.94% | k\_\_Bacteria;p\_\_Firmicutes;c\_\_Clostridia;o\_\_Clostridiales;f\_\_Lachnospiraceae;g\_\_Roseburia |
| 0.0163127986203 | 1.63% | k\_\_Bacteria;p\_\_Firmicutes;c\_\_Clostridia;o\_\_Clostridiales;f\_\_Lachnospiraceae;g\_\_Blautia |
| 0.0122982103983 | 1.23% | k\_\_Bacteria;p\_\_Firmicutes;c\_\_Clostridia;o\_\_Clostridiales;f\_\_Ruminococcaceae;g\_\_Butyricicoccus |
| 0.0109694382404 | 1.10% | k\_\_Bacteria;p\_\_Firmicutes;c\_\_Clostridia;o\_\_Clostridiales;f\_\_Lachnospiraceae;g\_\_ |
| 0.0101354216731 | 1.01% | k\_\_Bacteria;p\_\_Firmicutes;c\_\_Clostridia;o\_\_Clostridiales;f\_\_Ruminococcaceae;g\_\_Ruminiclostridium |
| 0.00923072573577 | 0.92% | k\_\_Bacteria;p\_\_Bacteroidetes;c\_\_Bacteroidia;o\_\_Bacteroidales;f\_\_Prevotellaceae;g\_\_Alloprevotella |
| 0.00829775805038 | 0.83% | k\_\_Bacteria;p\_\_Proteobacteria;c\_\_Deltaproteobacteria;o\_\_Desulfovibrionales;f\_\_Desulfovibrionaceae;g\_\_Desulfovibrio |
| 0.00595120296288 | 0.60% | k\_\_Bacteria;p\_\_Firmicutes;c\_\_Clostridia;o\_\_Clostridiales;f\_\_Ruminococcaceae;g\_\_Intestinimonas |
| 0.00554126261627 | 0.55% | k\_\_Bacteria;p\_\_Firmicutes;c\_\_Clostridia;o\_\_Clostridiales;Other;Other |
| 0.00541403975008 | 0.54% | k\_\_Bacteria;p\_\_Proteobacteria;c\_\_Gammaproteobacteria;o\_\_Enterobacteriales;f\_\_Enterobacteriaceae;g\_\_unidentified\_Enterobacteriaceae |
| 0.00537163212801 | 0.54% | k\_\_Bacteria;p\_\_Bacteroidetes;c\_\_Bacteroidia;o\_\_Bacteroidales;f\_\_Tannerellaceae;g\_\_Parabacteroides |
| 0.00467897430098 | 0.47% | k\_\_Bacteria;p\_\_Firmicutes;c\_\_Clostridia;o\_\_Clostridiales;f\_\_Ruminococcaceae;g\_\_Anaerotruncus |
| 0.00449520793871 | 0.45% | k\_\_Bacteria;p\_\_Bacteroidetes;c\_\_Bacteroidia;o\_\_Bacteroidales;f\_\_Muribaculaceae;g\_\_Muribaculum |
| 0.00371773486755 | 0.37% | k\_\_Bacteria;p\_\_Proteobacteria;c\_\_Gammaproteobacteria;o\_\_Enterobacteriales;f\_\_Enterobacteriaceae;g\_\_Proteus |
| 0.00371773486755 | 0.37% | k\_\_Bacteria;p\_\_Firmicutes;c\_\_Bacilli;o\_\_Lactobacillales;f\_\_Lactobacillaceae;g\_\_Lactobacillus |
| 0.0023465550875 | 0.23% | k\_\_Bacteria;p\_\_Firmicutes;c\_\_Clostridia;o\_\_Clostridiales;f\_\_Peptococcaceae;g\_\_ |
| 0.00214865285121 | 0.21% | k\_\_Bacteria;p\_\_Firmicutes;c\_\_Erysipelotrichia;o\_\_Erysipelotrichales;f\_\_Erysipelotrichaceae;Other |
| 0.00188007124481 | 0.19% | k\_\_Bacteria;Other;Other;Other;Other;Other |
| 0.00183766362274 | 0.18% | k\_\_Bacteria;p\_\_Proteobacteria;c\_\_Gammaproteobacteria;o\_\_Enterobacteriales;f\_\_Enterobacteriaceae;Other |
| 0.00147013089819 | 0.15% | k\_\_Bacteria;p\_\_Firmicutes;c\_\_Clostridia;o\_\_Clostridiales;f\_\_Ruminococcaceae;g\_\_Harryflintia |
| 0.00130050040994 | 0.13% | k\_\_Bacteria;p\_\_Verrucomicrobia;c\_\_Verrucomicrobiae;o\_\_Verrucomicrobiales;f\_\_Akkermansiaceae;g\_\_Akkermansia |
| 0.00125809278788 | 0.13% | k\_\_Bacteria;p\_\_Firmicutes;c\_\_Bacilli;o\_\_Lactobacillales;f\_\_Enterococcaceae;g\_\_Enterococcus |
| 0.00101778292952 | 0.10% | k\_\_Bacteria;p\_\_Firmicutes;c\_\_Clostridia;o\_\_Clostridiales;f\_\_Lachnospiraceae;g\_\_Acetatifactor |
| 0.000918831811371 | 0.09% | k\_\_Bacteria;p\_\_Firmicutes;c\_\_Clostridia;o\_\_Clostridiales;f\_\_Ruminococcaceae;g\_\_Faecalibacterium |
| 0.000876424189308 | 0.09% | k\_\_Bacteria;p\_\_Firmicutes;c\_\_Clostridia;o\_\_Clostridiales;f\_\_unidentified\_Clostridiales;g\_\_unidentified\_Clostridiales |
| 0.000862288315287 | 0.09% | k\_\_Bacteria;p\_\_Firmicutes;Other;Other;Other;Other |
| 0.000819880693223 | 0.08% | k\_\_Bacteria;p\_\_Tenericutes;c\_\_Mollicutes;o\_\_;f\_\_;g\_\_ |
| 0.000579570834865 | 0.06% | k\_\_Bacteria;p\_\_Firmicutes;c\_\_Clostridia;o\_\_Clostridiales;f\_\_Lachnospiraceae;g\_\_Tyzzerella |
| 0.000438212094654 | 0.04% | k\_\_Bacteria;p\_\_Bacteroidetes;c\_\_Bacteroidia;o\_\_Bacteroidales;f\_\_Prevotellaceae;g\_\_unidentified\_Prevotellaceae |
| 0.000424076220633 | 0.04% | k\_\_Bacteria;p\_\_Firmicutes;c\_\_Negativicutes;o\_\_Selenomonadales;f\_\_Veillonellaceae;g\_\_Dialister |
| 0.000395804472591 | 0.04% | k\_\_Bacteria;p\_\_Firmicutes;c\_\_Clostridia;o\_\_Clostridiales;f\_\_Ruminococcaceae;g\_\_Subdoligranulum |
| 0.000381668598569 | 0.04% | k\_\_Bacteria;p\_\_Firmicutes;c\_\_Clostridia;o\_\_Clostridiales;f\_\_Peptococcaceae;g\_\_Peptococcus |
| 0.000367532724548 | 0.04% | k\_\_Bacteria;p\_\_unidentified\_Bacteria;c\_\_unidentified\_Bacteria;o\_\_Campylobacterales;f\_\_unidentified\_Campylobacterales;g\_\_Sulfurospirillum |
| 0.000367532724548 | 0.04% | k\_\_Bacteria;p\_\_Firmicutes;c\_\_Clostridia;o\_\_Clostridiales;f\_\_unidentified\_Clostridiales;g\_\_Anaerovorax |
| 0.000325125102485 | 0.03% | k\_\_Bacteria;p\_\_Melainabacteria;Other;Other;Other;Other |
| 0.000310989228464 | 0.03% | k\_\_Bacteria;p\_\_Fusobacteria;c\_\_Fusobacteriia;o\_\_Fusobacteriales;f\_\_Fusobacteriaceae;g\_\_Fusobacterium |
| 0.000310989228464 | 0.03% | k\_\_Bacteria;p\_\_Firmicutes;c\_\_Clostridia;o\_\_Clostridiales;f\_\_Lachnospiraceae;g\_\_Fusicatenibacter |
| 0.000282717480422 | 0.03% | k\_\_Bacteria;p\_\_Proteobacteria;c\_\_Gammaproteobacteria;o\_\_unidentified\_Gammaproteobacteria;f\_\_Burkholderiaceae;g\_\_Diaphorobacter |
| 0.000268581606401 | 0.03% | k\_\_Bacteria;p\_\_Firmicutes;c\_\_Clostridia;o\_\_Clostridiales;f\_\_unidentified\_Clostridiales;Other |
| 0.000268581606401 | 0.03% | k\_\_Bacteria;p\_\_Bacteroidetes;c\_\_Bacteroidia;o\_\_Bacteroidales;f\_\_Prevotellaceae;Other |
| 0.000226173984337 | 0.02% | k\_\_Bacteria;p\_\_Bacteroidetes;c\_\_Bacteroidia;o\_\_Bacteroidales;f\_\_Marinifilaceae;g\_\_Butyricimonas |
| 0.000212038110316 | 0.02% | k\_\_Bacteria;p\_\_Actinobacteria;c\_\_unidentified\_Actinobacteria;o\_\_Bifidobacteriales;f\_\_Bifidobacteriaceae;g\_\_Bifidobacterium |
| 0.000197902236295 | 0.02% | k\_\_Bacteria;p\_\_unidentified\_Bacteria;c\_\_unidentified\_Bacteria;o\_\_unidentified\_Bacteria;f\_\_unidentified\_Bacteria;g\_\_Candidatus\_Saccharimonas |
| 0.000155494614232 | 0.02% | k\_\_Bacteria;p\_\_Firmicutes;c\_\_Erysipelotrichia;o\_\_Erysipelotrichales;f\_\_Erysipelotrichaceae;g\_\_Dubosiella |
| 0.000155494614232 | 0.02% | k\_\_Bacteria;p\_\_Firmicutes;c\_\_Clostridia;o\_\_Clostridiales;f\_\_Ruminococcaceae;g\_\_Caproiciproducens |
| 0.000141358740211 | 0.01% | k\_\_Bacteria;p\_\_Proteobacteria;c\_\_Gammaproteobacteria;o\_\_unidentified\_Gammaproteobacteria;f\_\_Burkholderiaceae;g\_\_Sutterella |
| 0.00012722286619 | 0.01% | k\_\_Bacteria;p\_\_Firmicutes;c\_\_Negativicutes;o\_\_Selenomonadales;f\_\_Acidaminococcaceae;g\_\_Phascolarctobacterium |
| 0.00012722286619 | 0.01% | k\_\_Bacteria;p\_\_Firmicutes;c\_\_Clostridia;o\_\_Clostridiales;f\_\_Ruminococcaceae;g\_\_Flavonifractor |
| 0.00012722286619 | 0.01% | k\_\_Bacteria;p\_\_Firmicutes;c\_\_Clostridia;o\_\_Clostridiales;f\_\_Lachnospiraceae;g\_\_Anaerostipes |
| 0.00012722286619 | 0.01% | k\_\_Bacteria;p\_\_Bacteroidetes;c\_\_Bacteroidia;o\_\_Bacteroidales;f\_\_Porphyromonadaceae;g\_\_Porphyromonas |
| 0.00012722286619 | 0.01% | k\_\_Bacteria;p\_\_Bacteroidetes;c\_\_Bacteroidia;o\_\_Bacteroidales;f\_\_Marinifilaceae;g\_\_Odoribacter |
| 0.000113086992169 | 0.01% | k\_\_Bacteria;p\_\_Firmicutes;c\_\_Clostridia;o\_\_Clostridiales;f\_\_Peptostreptococcaceae;g\_\_Romboutsia |
| 0.000113086992169 | 0.01% | k\_\_Bacteria;p\_\_Firmicutes;c\_\_Clostridia;o\_\_Clostridiales;f\_\_Lachnospiraceae;g\_\_Marvinbryantia |
| 0.000113086992169 | 0.01% | k\_\_Bacteria;p\_\_Bacteroidetes;c\_\_Bacteroidia;o\_\_Bacteroidales;f\_\_Prevotellaceae;g\_\_Prevotella |
| 8.48152441265e-05 | 0.01% | k\_\_Bacteria;p\_\_Proteobacteria;c\_\_Deltaproteobacteria;o\_\_Desulfovibrionales;f\_\_Desulfovibrionaceae;g\_\_ |
| 8.48152441265e-05 | 0.01% | k\_\_Bacteria;p\_\_Firmicutes;c\_\_Negativicutes;o\_\_Selenomonadales;f\_\_Veillonellaceae;g\_\_Veillonella |
| 8.48152441265e-05 | 0.01% | k\_\_Bacteria;p\_\_Actinobacteria;c\_\_unidentified\_Actinobacteria;o\_\_Actinomycetales;f\_\_Actinomycetaceae;g\_\_Actinomyces |
| 7.06793701055e-05 | 0.01% | k\_\_Bacteria;p\_\_Proteobacteria;c\_\_Gammaproteobacteria;o\_\_Pseudomonadales;f\_\_Moraxellaceae;g\_\_Acinetobacter |
| 5.65434960844e-05 | 0.01% | k\_\_Bacteria;p\_\_Proteobacteria;c\_\_Gammaproteobacteria;o\_\_unidentified\_Gammaproteobacteria;f\_\_Burkholderiaceae;g\_\_Parasutterella |
| 5.65434960844e-05 | 0.01% | k\_\_Bacteria;p\_\_Proteobacteria;c\_\_Gammaproteobacteria;o\_\_unidentified\_Gammaproteobacteria;f\_\_Burkholderiaceae;Other |
| 5.65434960844e-05 | 0.01% | k\_\_Bacteria;p\_\_Proteobacteria;c\_\_Alphaproteobacteria;o\_\_Rhodospirillales;f\_\_unidentified\_Rhodospirillales;g\_\_Telmatospirillum |
| 5.65434960844e-05 | 0.01% | k\_\_Bacteria;p\_\_Firmicutes;c\_\_Negativicutes;o\_\_Selenomonadales;f\_\_Veillonellaceae;g\_\_Megamonas |
| 5.65434960844e-05 | 0.01% | k\_\_Bacteria;p\_\_Firmicutes;c\_\_Erysipelotrichia;o\_\_Erysipelotrichales;f\_\_Erysipelotrichaceae;g\_\_unidentified\_Erysipelotrichaceae |
| 5.65434960844e-05 | 0.01% | k\_\_Bacteria;p\_\_Bacteroidetes;c\_\_Bacteroidia;o\_\_Bacteroidales;f\_\_Prevotellaceae;g\_\_ |
| 5.65434960844e-05 | 0.01% | k\_\_Bacteria;p\_\_Bacteroidetes;c\_\_Bacteroidia;o\_\_Bacteroidales;f\_\_Barnesiellaceae;g\_\_Barnesiella |
| 4.24076220633e-05 | 0.00% | k\_\_Bacteria;p\_\_Proteobacteria;c\_\_Gammaproteobacteria;o\_\_unidentified\_Gammaproteobacteria;f\_\_Burkholderiaceae;g\_\_Delftia |
| 4.24076220633e-05 | 0.00% | k\_\_Bacteria;p\_\_Proteobacteria;c\_\_Gammaproteobacteria;o\_\_Xanthomonadales;f\_\_Xanthomonadaceae;g\_\_Stenotrophomonas |
| 4.24076220633e-05 | 0.00% | k\_\_Bacteria;p\_\_Proteobacteria;c\_\_Gammaproteobacteria;o\_\_Pseudomonadales;f\_\_Pseudomonadaceae;g\_\_Pseudomonas |
| 4.24076220633e-05 | 0.00% | k\_\_Bacteria;p\_\_Proteobacteria;c\_\_Gammaproteobacteria;o\_\_Pasteurellales;f\_\_Pasteurellaceae;Other |
| 4.24076220633e-05 | 0.00% | k\_\_Bacteria;p\_\_Proteobacteria;c\_\_Alphaproteobacteria;o\_\_Sphingomonadales;f\_\_Sphingomonadaceae;g\_\_Novosphingobium |
| 4.24076220633e-05 | 0.00% | k\_\_Bacteria;p\_\_Oxyphotobacteria;c\_\_unidentified\_Oxyphotobacteria;o\_\_unidentified\_Oxyphotobacteria;f\_\_unidentified\_Oxyphotobacteria;g\_\_unidentified\_Oxyphotobacteria |
| 4.24076220633e-05 | 0.00% | k\_\_Bacteria;p\_\_Firmicutes;c\_\_Negativicutes;o\_\_Selenomonadales;f\_\_Veillonellaceae;g\_\_Allisonella |
| 4.24076220633e-05 | 0.00% | k\_\_Bacteria;p\_\_Firmicutes;c\_\_Negativicutes;o\_\_Selenomonadales;f\_\_Acidaminococcaceae;g\_\_Acidaminococcus |
| 4.24076220633e-05 | 0.00% | k\_\_Bacteria;p\_\_Firmicutes;c\_\_Erysipelotrichia;o\_\_Erysipelotrichales;f\_\_Erysipelotrichaceae;g\_\_Holdemania |
| 4.24076220633e-05 | 0.00% | k\_\_Bacteria;p\_\_Firmicutes;c\_\_Erysipelotrichia;o\_\_Erysipelotrichales;f\_\_Erysipelotrichaceae;g\_\_Erysipelatoclostridium |
| 4.24076220633e-05 | 0.00% | k\_\_Bacteria;p\_\_Firmicutes;c\_\_Clostridia;o\_\_Clostridiales;f\_\_unidentified\_Clostridiales;g\_\_Parvimonas |
| 4.24076220633e-05 | 0.00% | k\_\_Bacteria;p\_\_Firmicutes;c\_\_Clostridia;o\_\_Clostridiales;f\_\_Ruminococcaceae;g\_\_Hydrogenoanaerobacterium |
| 4.24076220633e-05 | 0.00% | k\_\_Bacteria;p\_\_Firmicutes;c\_\_Clostridia;o\_\_Clostridiales;f\_\_Peptostreptococcaceae;g\_\_Peptostreptococcus |
| 4.24076220633e-05 | 0.00% | k\_\_Bacteria;p\_\_Firmicutes;c\_\_Clostridia;o\_\_Clostridiales;f\_\_Eubacteriaceae;g\_\_Anaerofustis |
| 4.24076220633e-05 | 0.00% | k\_\_Bacteria;p\_\_Firmicutes;c\_\_Bacilli;o\_\_Bacillales;f\_\_unidentified\_Bacillales;g\_\_Exiguobacterium |
| 2.82717480422e-05 | 0.00% | k\_\_Bacteria;p\_\_Firmicutes;c\_\_Erysipelotrichia;o\_\_Erysipelotrichales;f\_\_Erysipelotrichaceae;g\_\_Turicibacter |
| 2.82717480422e-05 | 0.00% | k\_\_Bacteria;p\_\_Firmicutes;c\_\_Clostridia;o\_\_Clostridiales;f\_\_Christensenellaceae;g\_\_ |
| 2.82717480422e-05 | 0.00% | k\_\_Bacteria;p\_\_Firmicutes;c\_\_Bacilli;o\_\_Lactobacillales;f\_\_Streptococcaceae;g\_\_Streptococcus |
| 2.82717480422e-05 | 0.00% | k\_\_Bacteria;p\_\_Actinobacteria;c\_\_unidentified\_Actinobacteria;o\_\_Corynebacteriales;f\_\_Corynebacteriaceae;g\_\_unidentified\_Corynebacteriaceae |
| 1.41358740211e-05 | 0.00% | k\_\_Bacteria;p\_\_Proteobacteria;c\_\_Alphaproteobacteria;o\_\_Rhizobiales;f\_\_Rhizobiaceae;g\_\_Phyllobacterium |
| 1.41358740211e-05 | 0.00% | k\_\_Bacteria;p\_\_Proteobacteria;c\_\_Alphaproteobacteria;o\_\_Rhizobiales;f\_\_Rhizobiaceae;g\_\_Brucella |
| 1.41358740211e-05 | 0.00% | k\_\_Bacteria;p\_\_Firmicutes;c\_\_Erysipelotrichia;o\_\_Erysipelotrichales;f\_\_Erysipelotrichaceae;g\_\_Faecalitalea |
| 1.41358740211e-05 | 0.00% | k\_\_Bacteria;p\_\_Firmicutes;c\_\_Bacilli;o\_\_Bacillales;f\_\_Staphylococcaceae;g\_\_Staphylococcus |
| 1.41358740211e-05 | 0.00% | k\_\_Bacteria;p\_\_Actinobacteria;c\_\_unidentified\_Actinobacteria;o\_\_Corynebacteriales;f\_\_Nocardiaceae;g\_\_Rhodococcus |

|  |  |
| --- | --- |
|  | |
| Taxonomy Summary. Current Level: \_MC5 | |
| View Figure (.pdf)  View Legend (.pdf) |  |
|  |  |
| View Figure (.pdf)  View Legend (.pdf) |  |
|  |  |
|  |  |

|  |  |  |
| --- | --- | --- |
| Count | Pct | Taxonomy |
| 0.25862995109 | 25.86% | k\_\_Bacteria;p\_\_Bacteroidetes;c\_\_Bacteroidia;o\_\_Bacteroidales;f\_\_Muribaculaceae;g\_\_ |
| 0.195018517995 | 19.50% | k\_\_Bacteria;p\_\_Bacteroidetes;c\_\_Bacteroidia;o\_\_Bacteroidales;f\_\_Bacteroidaceae;g\_\_Bacteroides |
| 0.13242486783 | 13.24% | k\_\_Bacteria;p\_\_Firmicutes;c\_\_Clostridia;o\_\_Clostridiales;f\_\_Lachnospiraceae;Other |
| 0.116437194312 | 11.64% | k\_\_Bacteria;p\_\_Firmicutes;c\_\_Clostridia;o\_\_Clostridiales;f\_\_Ruminococcaceae;Other |
| 0.0428316982839 | 4.28% | k\_\_Bacteria;p\_\_Firmicutes;c\_\_Clostridia;o\_\_Clostridiales;f\_\_Ruminococcaceae;g\_\_unidentified\_Ruminococcaceae |
| 0.0365412343445 | 3.65% | k\_\_Bacteria;p\_\_Firmicutes;c\_\_Clostridia;o\_\_Clostridiales;f\_\_Lachnospiraceae;g\_\_unidentified\_Lachnospiraceae |
| 0.0250063611433 | 2.50% | k\_\_Bacteria;p\_\_Bacteroidetes;c\_\_Bacteroidia;o\_\_Bacteroidales;f\_\_Prevotellaceae;g\_\_Alloprevotella |
| 0.0229001159142 | 2.29% | k\_\_Bacteria;p\_\_Proteobacteria;c\_\_Gammaproteobacteria;o\_\_Enterobacteriales;f\_\_Enterobacteriaceae;g\_\_unidentified\_Enterobacteriaceae |
| 0.0222357298352 | 2.22% | k\_\_Bacteria;p\_\_Proteobacteria;c\_\_Gammaproteobacteria;o\_\_Enterobacteriales;f\_\_Enterobacteriaceae;Other |
| 0.0193944191569 | 1.94% | k\_\_Bacteria;p\_\_Bacteroidetes;c\_\_Bacteroidia;o\_\_Bacteroidales;f\_\_Rikenellaceae;g\_\_Alistipes |
| 0.0141782816432 | 1.42% | k\_\_Bacteria;p\_\_Verrucomicrobia;c\_\_Verrucomicrobiae;o\_\_Verrucomicrobiales;f\_\_Akkermansiaceae;g\_\_Akkermansia |
| 0.0104605467756 | 1.05% | k\_\_Bacteria;p\_\_Firmicutes;c\_\_Clostridia;o\_\_Clostridiales;f\_\_Lachnospiraceae;g\_\_Blautia |
| 0.0101636934212 | 1.02% | k\_\_Bacteria;p\_\_Proteobacteria;c\_\_Deltaproteobacteria;o\_\_Desulfovibrionales;f\_\_Desulfovibrionaceae;g\_\_Desulfovibrio |
| 0.00907523112154 | 0.91% | k\_\_Bacteria;p\_\_Firmicutes;c\_\_Clostridia;o\_\_Clostridiales;f\_\_Ruminococcaceae;g\_\_Oscillibacter |
| 0.00883492126318 | 0.88% | k\_\_Bacteria;p\_\_Firmicutes;c\_\_Clostridia;o\_\_Clostridiales;f\_\_Lachnospiraceae;g\_\_Lachnoclostridium |
| 0.00736479036499 | 0.74% | k\_\_Bacteria;p\_\_Bacteroidetes;c\_\_Bacteroidia;o\_\_Bacteroidales;f\_\_Tannerellaceae;g\_\_Parabacteroides |
| 0.00718102400271 | 0.72% | k\_\_Bacteria;p\_\_Firmicutes;c\_\_Clostridia;o\_\_Clostridiales;f\_\_Lachnospiraceae;g\_\_Roseburia |
| 0.00718102400271 | 0.72% | k\_\_Bacteria;p\_\_Firmicutes;c\_\_Clostridia;o\_\_Clostridiales;Other;Other |
| 0.00460829493088 | 0.46% | k\_\_Bacteria;p\_\_Tenericutes;c\_\_Mollicutes;o\_\_;f\_\_;g\_\_ |
| 0.00460829493088 | 0.46% | k\_\_Bacteria;p\_\_Firmicutes;c\_\_Bacilli;o\_\_Lactobacillales;f\_\_Lactobacillaceae;g\_\_Lactobacillus |
| 0.00418421871024 | 0.42% | k\_\_Bacteria;p\_\_Firmicutes;c\_\_Clostridia;o\_\_Clostridiales;f\_\_Ruminococcaceae;g\_\_Ruminiclostridium |
| 0.00337847389104 | 0.34% | k\_\_Bacteria;p\_\_Bacteroidetes;c\_\_Bacteroidia;o\_\_Bacteroidales;f\_\_Muribaculaceae;g\_\_Muribaculum |
| 0.00329365864691 | 0.33% | k\_\_Bacteria;p\_\_Firmicutes;c\_\_Clostridia;o\_\_Clostridiales;f\_\_Ruminococcaceae;g\_\_Intestinimonas |
| 0.00326538689887 | 0.33% | k\_\_Bacteria;p\_\_Firmicutes;c\_\_Clostridia;o\_\_Clostridiales;f\_\_Ruminococcaceae;g\_\_Anaerotruncus |
| 0.00310989228464 | 0.31% | k\_\_Bacteria;p\_\_Bacteroidetes;c\_\_Bacteroidia;o\_\_Bacteroidales;f\_\_Prevotellaceae;Other |
| 0.00302507704051 | 0.30% | k\_\_Bacteria;p\_\_Firmicutes;c\_\_Bacilli;o\_\_Lactobacillales;f\_\_Enterococcaceae;g\_\_Enterococcus |
| 0.00275649543411 | 0.28% | k\_\_Bacteria;p\_\_Proteobacteria;c\_\_Gammaproteobacteria;o\_\_Enterobacteriales;f\_\_Enterobacteriaceae;g\_\_Proteus |
| 0.00265754431597 | 0.27% | k\_\_Bacteria;p\_\_Firmicutes;c\_\_Clostridia;o\_\_Clostridiales;f\_\_Lachnospiraceae;g\_\_ |
| 0.00240309858359 | 0.24% | k\_\_Bacteria;p\_\_Firmicutes;c\_\_Clostridia;o\_\_Clostridiales;f\_\_Ruminococcaceae;g\_\_Butyricicoccus |
| 0.00192247886687 | 0.19% | k\_\_Bacteria;p\_\_Firmicutes;c\_\_Erysipelotrichia;o\_\_Erysipelotrichales;f\_\_Erysipelotrichaceae;Other |
| 0.00152667439428 | 0.15% | k\_\_Bacteria;p\_\_Firmicutes;c\_\_Clostridia;o\_\_Clostridiales;f\_\_unidentified\_Clostridiales;g\_\_unidentified\_Clostridiales |
| 0.00144185915015 | 0.14% | k\_\_Bacteria;p\_\_Melainabacteria;Other;Other;Other;Other |
| 0.0010743264256 | 0.11% | k\_\_Bacteria;Other;Other;Other;Other;Other |
| 0.0010036470555 | 0.10% | k\_\_Bacteria;p\_\_Bacteroidetes;c\_\_Bacteroidia;o\_\_Bacteroidales;f\_\_Marinifilaceae;g\_\_Butyricimonas |
| 0.000975375307455 | 0.10% | k\_\_Bacteria;p\_\_Firmicutes;c\_\_Clostridia;o\_\_Clostridiales;f\_\_Lachnospiraceae;g\_\_Acetatifactor |
| 0.000819880693223 | 0.08% | k\_\_Bacteria;p\_\_unidentified\_Bacteria;c\_\_unidentified\_Bacteria;o\_\_unidentified\_Bacteria;f\_\_unidentified\_Bacteria;g\_\_Candidatus\_Saccharimonas |
| 0.000735065449097 | 0.07% | k\_\_Bacteria;p\_\_Firmicutes;c\_\_Clostridia;o\_\_Clostridiales;f\_\_Ruminococcaceae;g\_\_Faecalibacterium |
| 0.000735065449097 | 0.07% | k\_\_Bacteria;p\_\_Firmicutes;c\_\_Clostridia;o\_\_Clostridiales;f\_\_Peptococcaceae;g\_\_ |
| 0.000537163212801 | 0.05% | k\_\_Bacteria;p\_\_Firmicutes;c\_\_Clostridia;o\_\_Clostridiales;f\_\_Lachnospiraceae;g\_\_Tyzzerella |
| 0.000452347968675 | 0.05% | k\_\_Bacteria;p\_\_Firmicutes;c\_\_Clostridia;o\_\_Clostridiales;f\_\_Ruminococcaceae;g\_\_Harryflintia |
| 0.000438212094654 | 0.04% | k\_\_Bacteria;p\_\_Firmicutes;c\_\_Clostridia;o\_\_Clostridiales;f\_\_Ruminococcaceae;g\_\_Subdoligranulum |
| 0.000409940346612 | 0.04% | k\_\_Bacteria;p\_\_Actinobacteria;c\_\_unidentified\_Actinobacteria;o\_\_Corynebacteriales;f\_\_Nocardiaceae;g\_\_Rhodococcus |
| 0.000367532724548 | 0.04% | k\_\_Bacteria;p\_\_Bacteroidetes;c\_\_Bacteroidia;o\_\_Bacteroidales;f\_\_Prevotellaceae;g\_\_unidentified\_Prevotellaceae |
| 0.000339260976506 | 0.03% | k\_\_Bacteria;p\_\_Fusobacteria;c\_\_Fusobacteriia;o\_\_Fusobacteriales;f\_\_Fusobacteriaceae;g\_\_Fusobacterium |
| 0.000310989228464 | 0.03% | k\_\_Bacteria;p\_\_Firmicutes;Other;Other;Other;Other |
| 0.000268581606401 | 0.03% | k\_\_Bacteria;p\_\_Firmicutes;c\_\_Negativicutes;o\_\_Selenomonadales;f\_\_Veillonellaceae;g\_\_Dialister |
| 0.00025444573238 | 0.03% | k\_\_Bacteria;p\_\_Firmicutes;c\_\_Clostridia;o\_\_Clostridiales;f\_\_Ruminococcaceae;g\_\_Caproiciproducens |
| 0.00025444573238 | 0.03% | k\_\_Bacteria;p\_\_Actinobacteria;c\_\_unidentified\_Actinobacteria;o\_\_Bifidobacteriales;f\_\_Bifidobacteriaceae;g\_\_Bifidobacterium |
| 0.000240309858359 | 0.02% | k\_\_Bacteria;p\_\_Firmicutes;c\_\_Clostridia;o\_\_Clostridiales;f\_\_unidentified\_Clostridiales;Other |
| 0.000183766362274 | 0.02% | k\_\_Bacteria;p\_\_Proteobacteria;c\_\_Gammaproteobacteria;o\_\_unidentified\_Gammaproteobacteria;f\_\_Burkholderiaceae;g\_\_Sutterella |
| 0.000169630488253 | 0.02% | k\_\_Bacteria;p\_\_Bacteroidetes;c\_\_Bacteroidia;o\_\_Bacteroidales;f\_\_Prevotellaceae;g\_\_Prevotella |
| 0.000141358740211 | 0.01% | k\_\_Bacteria;p\_\_Proteobacteria;c\_\_Gammaproteobacteria;o\_\_Pseudomonadales;f\_\_Moraxellaceae;g\_\_Acinetobacter |
| 0.000141358740211 | 0.01% | k\_\_Bacteria;p\_\_Firmicutes;c\_\_Clostridia;o\_\_Clostridiales;f\_\_Lachnospiraceae;g\_\_Fusicatenibacter |
| 0.00012722286619 | 0.01% | k\_\_Bacteria;p\_\_Proteobacteria;c\_\_Gammaproteobacteria;o\_\_Pasteurellales;f\_\_Pasteurellaceae;Other |
| 0.00012722286619 | 0.01% | k\_\_Bacteria;p\_\_Firmicutes;c\_\_Erysipelotrichia;o\_\_Erysipelotrichales;f\_\_Erysipelotrichaceae;g\_\_Dubosiella |
| 0.000113086992169 | 0.01% | k\_\_Bacteria;p\_\_Proteobacteria;c\_\_Gammaproteobacteria;o\_\_unidentified\_Gammaproteobacteria;f\_\_Burkholderiaceae;g\_\_Parasutterella |
| 0.000113086992169 | 0.01% | k\_\_Bacteria;p\_\_Firmicutes;c\_\_Erysipelotrichia;o\_\_Erysipelotrichales;f\_\_Erysipelotrichaceae;g\_\_unidentified\_Erysipelotrichaceae |
| 9.89511181476e-05 | 0.01% | k\_\_Bacteria;p\_\_Proteobacteria;c\_\_Gammaproteobacteria;o\_\_Pseudomonadales;f\_\_Pseudomonadaceae;g\_\_Pseudomonas |
| 9.89511181476e-05 | 0.01% | k\_\_Bacteria;p\_\_Firmicutes;c\_\_Negativicutes;o\_\_Selenomonadales;f\_\_Acidaminococcaceae;g\_\_Acidaminococcus |
| 9.89511181476e-05 | 0.01% | k\_\_Bacteria;p\_\_Firmicutes;c\_\_Clostridia;o\_\_Clostridiales;f\_\_Ruminococcaceae;g\_\_Flavonifractor |
| 9.89511181476e-05 | 0.01% | k\_\_Bacteria;p\_\_Firmicutes;c\_\_Clostridia;o\_\_Clostridiales;f\_\_Lachnospiraceae;g\_\_Marvinbryantia |
| 9.89511181476e-05 | 0.01% | k\_\_Bacteria;p\_\_Firmicutes;c\_\_Bacilli;o\_\_Lactobacillales;f\_\_Streptococcaceae;g\_\_Streptococcus |
| 8.48152441265e-05 | 0.01% | k\_\_Bacteria;p\_\_Firmicutes;c\_\_Clostridia;o\_\_Clostridiales;f\_\_Lachnospiraceae;g\_\_Lachnospira |
| 8.48152441265e-05 | 0.01% | k\_\_Bacteria;p\_\_Firmicutes;c\_\_Clostridia;o\_\_Clostridiales;f\_\_Lachnospiraceae;g\_\_Anaerostipes |
| 8.48152441265e-05 | 0.01% | k\_\_Bacteria;p\_\_Bacteroidetes;c\_\_Bacteroidia;o\_\_Bacteroidales;f\_\_Porphyromonadaceae;g\_\_Porphyromonas |
| 7.06793701055e-05 | 0.01% | k\_\_Bacteria;p\_\_Firmicutes;c\_\_Negativicutes;o\_\_Selenomonadales;f\_\_Veillonellaceae;g\_\_Megasphaera |
| 5.65434960844e-05 | 0.01% | k\_\_Bacteria;p\_\_Firmicutes;c\_\_Negativicutes;o\_\_Selenomonadales;f\_\_Veillonellaceae;g\_\_Veillonella |
| 5.65434960844e-05 | 0.01% | k\_\_Bacteria;p\_\_Firmicutes;c\_\_Negativicutes;o\_\_Selenomonadales;f\_\_Veillonellaceae;g\_\_Megamonas |
| 5.65434960844e-05 | 0.01% | k\_\_Bacteria;p\_\_Firmicutes;c\_\_Negativicutes;o\_\_Selenomonadales;f\_\_Acidaminococcaceae;g\_\_Phascolarctobacterium |
| 5.65434960844e-05 | 0.01% | k\_\_Bacteria;p\_\_Bacteroidetes;c\_\_Bacteroidia;o\_\_Bacteroidales;f\_\_Barnesiellaceae;g\_\_Coprobacter |
| 4.24076220633e-05 | 0.00% | k\_\_Bacteria;p\_\_unidentified\_Bacteria;c\_\_unidentified\_Bacteria;o\_\_Campylobacterales;f\_\_unidentified\_Campylobacterales;g\_\_Sulfurospirillum |
| 4.24076220633e-05 | 0.00% | k\_\_Bacteria;p\_\_Proteobacteria;c\_\_Gammaproteobacteria;o\_\_unidentified\_Gammaproteobacteria;f\_\_Burkholderiaceae;g\_\_Achromobacter |
| 4.24076220633e-05 | 0.00% | k\_\_Bacteria;p\_\_Proteobacteria;c\_\_Gammaproteobacteria;o\_\_unidentified\_Gammaproteobacteria;f\_\_Burkholderiaceae;Other |
| 4.24076220633e-05 | 0.00% | k\_\_Bacteria;p\_\_Firmicutes;c\_\_Clostridia;o\_\_Clostridiales;f\_\_Peptostreptococcaceae;g\_\_Romboutsia |
| 4.24076220633e-05 | 0.00% | k\_\_Bacteria;p\_\_Firmicutes;c\_\_Clostridia;o\_\_Clostridiales;f\_\_Peptostreptococcaceae;g\_\_Clostridioides |
| 4.24076220633e-05 | 0.00% | k\_\_Bacteria;p\_\_Firmicutes;c\_\_Clostridia;o\_\_Clostridiales;f\_\_Peptococcaceae;g\_\_Peptococcus |
| 4.24076220633e-05 | 0.00% | k\_\_Bacteria;p\_\_Bacteroidetes;c\_\_Bacteroidia;o\_\_Bacteroidales;f\_\_Barnesiellaceae;g\_\_Barnesiella |
| 2.82717480422e-05 | 0.00% | k\_\_Bacteria;p\_\_Proteobacteria;c\_\_Gammaproteobacteria;o\_\_unidentified\_Gammaproteobacteria;f\_\_Burkholderiaceae;g\_\_Delftia |
| 2.82717480422e-05 | 0.00% | k\_\_Bacteria;p\_\_Proteobacteria;c\_\_Deltaproteobacteria;o\_\_Desulfovibrionales;f\_\_Desulfovibrionaceae;g\_\_Bilophila |
| 2.82717480422e-05 | 0.00% | k\_\_Bacteria;p\_\_Proteobacteria;c\_\_Alphaproteobacteria;o\_\_Caulobacterales;f\_\_Caulobacteraceae;g\_\_Brevundimonas |
| 2.82717480422e-05 | 0.00% | k\_\_Bacteria;p\_\_Firmicutes;c\_\_Erysipelotrichia;o\_\_Erysipelotrichales;f\_\_Erysipelotrichaceae;g\_\_Holdemania |
| 2.82717480422e-05 | 0.00% | k\_\_Bacteria;p\_\_Acidobacteria;c\_\_unidentified\_Acidobacteria;o\_\_unidentified\_Acidobacteria;f\_\_unidentified\_Acidobacteria;g\_\_Blastocatella |
| 2.82717480422e-05 | 0.00% | k\_\_Bacteria;p\_\_Acidobacteria;c\_\_Holophagae;o\_\_;f\_\_;g\_\_ |
| 1.41358740211e-05 | 0.00% | k\_\_Bacteria;p\_\_Proteobacteria;c\_\_Gammaproteobacteria;o\_\_Xanthomonadales;f\_\_Xanthomonadaceae;g\_\_Stenotrophomonas |
| 1.41358740211e-05 | 0.00% | k\_\_Bacteria;p\_\_Proteobacteria;c\_\_Alphaproteobacteria;o\_\_Rhodospirillales;f\_\_unidentified\_Rhodospirillales;g\_\_Telmatospirillum |
| 1.41358740211e-05 | 0.00% | k\_\_Bacteria;p\_\_Proteobacteria;c\_\_Alphaproteobacteria;o\_\_Rhizobiales;f\_\_Rhizobiaceae;g\_\_Phyllobacterium |
| 1.41358740211e-05 | 0.00% | k\_\_Bacteria;p\_\_Firmicutes;c\_\_Erysipelotrichia;o\_\_Erysipelotrichales;f\_\_Erysipelotrichaceae;g\_\_Faecalitalea |
| 1.41358740211e-05 | 0.00% | k\_\_Bacteria;p\_\_Firmicutes;c\_\_Erysipelotrichia;o\_\_Erysipelotrichales;f\_\_Erysipelotrichaceae;g\_\_Erysipelatoclostridium |
| 1.41358740211e-05 | 0.00% | k\_\_Bacteria;p\_\_Firmicutes;c\_\_Clostridia;o\_\_Clostridiales;f\_\_unidentified\_Clostridiales;g\_\_Anaerovorax |
| 1.41358740211e-05 | 0.00% | k\_\_Bacteria;p\_\_Deinococcus-Thermus;c\_\_Deinococci;o\_\_Deinococcales;f\_\_Deinococcaceae;g\_\_Deinococcus |
| 1.41358740211e-05 | 0.00% | k\_\_Bacteria;p\_\_Bacteroidetes;c\_\_Bacteroidia;o\_\_Bacteroidales;f\_\_Marinifilaceae;g\_\_Odoribacter |
